# Trends in the incidence of common sexually transmitted infections at the global, regional and national levels, 1990–2021: results of the Global Burden of Disease 2021 study

**DOI:** 10.1186/s41182-025-00744-2

**Published:** 2025-05-16

**Authors:** Miao Deng, Jiaxi Chen, Zhi Wang, Rubin Zheng, Wenyi Pang, Rui Sun, Zhixun Bai

**Affiliations:** 1 Department of Nephrology, People’s Hospital of Qianxinan Prefecture, Xingyi, 562400 Guizhou China; 2 Graduate School, Zunyi Medical University, Zunyi, 563000 Guizhou China; 3 First Clinical College, Zunyi Medical University, Zunyi, 563000 Guizhou China; 4 Clinical College, Guizhou Medical University, Guiyang, 550000 Guizhou China

**Keywords:** Sexually transmitted infections, Incidence, DALYs, Global burden of disease

## Abstract

**Background:**

Sexually transmitted diseases (STDs) are prevalent globally and represent a significant public health challenge. This study aims to evaluate the most recent estimates of the burden of common sexually transmitted infections (STIs) at global, regional, and national levels, which will aid in the development of more effective prevention strategies.

**Methods:**

Data for this study were obtained from the Global Burden of Disease (GBD) 2021 study via the Global Health Data Exchange (GHDx) query tool (https://vizhub.healthdata.org/gbd-results/). We extracted the data in accordance with GBD operational guidelines, selecting the most recent results from the 2021 GBD study. The latest GBD study results provided data on incidence, prevalence, and disability-adjusted life years (DALYs) for 21 regions and 204 countries. We extracted the number of cases, incidence, and age-standardized incidence of sexually transmitted diseases (STDs) by sex, age group, and location, following GBD usage guidelines. The data were categorized into five groups based on sociological demographic indices (SDIs).

**Results:**

In 2021, the total number of STDs was ~ 289.17 million, reflecting an increase of about 58.38% compared to 1990. While the total number of cases was higher in males, the increase was more pronounced in females. Syphilis exhibited the highest age-standardized rate (ASR) in Equatorial Guinea (EAPC 0.57, 95% CI [− 2.97, 4.24]). Trichomoniasis had the greatest ASR in Tanzania (EAPC − 1.24, 95% CI [− 4.97, 2.64]). Gonococcal (EAPC − 0.52, 95% CI [− 4.33, 3.44]) and chlamydial infections (EAPC − 0.52, 95% CI [− 4.33, 3.44]) showed the highest ASR in South Africa, while genital herpes (EAPC − 1.3, 95% CI [− 4.89, 2.44]) had the greatest ASR in Zimbabwe. HIV/AIDS had the highest ASR in Lesotho (EAPC − 0.33, 95% CI [− 3.99, 3.46]), and the combined ASR for HIV/AIDS and STIs was highest in South Africa (EAPC − 0.47, 95% CI [− 0.58, 0.37]).

**Conclusion:**

The burden of STDs remains high and has been steadily increasing; the burden of STIs is more severe in low SDI areas and among young and middle-aged people; the prevalence, incidence, and disability-adjusted years of STIs during the period 1990–2021 are attributable to three main factors: population, disease epidemiology, and aging.

**Supplementary Information:**

The online version contains supplementary material available at 10.1186/s41182-025-00744-2.

## Introduction

Sexually transmitted infections (STIs) encompass over 30 distinct pathogenic microorganisms including bacteria, viruses, and parasites that are primarily transmitted through sexual contact (vaginal, anal, and oral intercourse). Vertical transmission mechanism during pregnancy, childbirth, and breastfeeding constitute additional routes of infection for certain STIs [1].

The Global Burden of Disease (GBD) study constitutes a comprehensive systematic analysis evaluating disease burden across genders and geographic regions from 1990 to 2021. Sexually transmitted infections (STIs) emerge as one of the most prevalent infectious disease groups worldwide, presenting formidable challenges to global public health systems. Current GBD 2021 classifications stratify STIs into two principal categories: human immunodeficiency virus (HIV) infections and non-HIV STIs. The latter category encompasses six distinct entities: syphilis (*Treponema pallidum*), chlamydia (*Chlamydia trachomatis*), gonorrhea (*Neisseria gonorrhoeae*), trichomoniasis (*Trichomonas vaginalis*), genital herpes (Herpes simplex virus), and other sexually transmitted diseases.

World Health Organization (WHO) [1] surveillance data reveal a staggering incidence of over one million treatable STI cases daily. Notably, 2020 WHO estimates documented 374 million new infections attributable to four major pathogens: chlamydia (129 million), gonorrhea (82 million), syphilis (7.1 million), and trichomoniasis (156 million). While most bacterial STIs are amenable to antimicrobial therapy, their clinical management is frequently complicated by prolonged asymptomatic latency periods, leading to underdiagnosis and substantial threats to individual and public health [2, 3].

As highlighted by the World Health Organization (WHO) [1], individuals infected with sexually transmitted infections (STIs) frequently experience prolonged asymptomatic periods, during which they remain capable of transmitting pathogens while developing severe long-term sequelae. These include but are not limited to: secondary opportunistic infections due to immunocompromised status, neoplastic transformations, chronic pelvic inflammatory syndrome, ectopic gestation, and reproductive system failure. Particularly in tropical regions with limited healthcare resources, the vertical transmission during perinatal period poses substantial risks of congenital abnormalities and neonatal morbidity.

Current epidemiological surveillance systems face substantial challenges in case ascertainment, particularly in resource-constrained settings where underreporting and diagnostic limitations contribute to significant underestimation of disease burden.

While contemporary therapeutic regimens can achieve virological suppression in most cases, the persistent transmission reservoirs and psychosocial impacts continue to compromise global health equity. This perpetuates a vicious cycle of health disparities, particularly affecting vulnerable populations in low- and middle-income countries.

Utilizing the Global Burden of Disease (GBD) 2021 dataset, we conducted a multilevel spatiotemporal regression analysis to delineate the epidemiological trajectories of major STIs (HIV/AIDS, syphilis, chlamydia, gonorrhea, trichomoniasis, genital herpes, and other STIS) across 204 countries and territories from 1990 to 2021. Our age-stratified, Bayesian meta-regression approach provides granular estimates at global, regional, and national levels, with particular focus on regions bearing disproportionate burden.

## Materials and methods

### Study data

Data on the annual incidence, age-standardized incidence rate (ASIR), disability-adjusted life years (DALYs), and age-standardized DALYs rate (DALYs rate) for sexually transmitted diseases (STDs) from 1990 to 2021 were collected using the Global Health Data Exchange (GHDx) query tool (http://ghdx.healthdata.org/gbd-results-tool). The data were categorized by sex, region, country, and STD type (HIV/AIDS, syphilis, chlamydial infection, gonococcal infection, trichomoniasis, genital herpes, and other sexually transmitted infections). Data for 204 countries and regions were available, which were then grouped into five categories based on the socio-demographic index (SDI): low, low-middle, middle, middle-high, and high. Additionally, the world is divided into 21 geographical regions. Detailed descriptions of the GBD data can be found in previous studies [4, 5], so they are not reiterated here.

### Statistical analysis

This study uses age-standardized incidence rate (ASIR), age-standardized DALYs rate (DALYs rate), and estimated annual percentage change (EAPC) to quantify the burden caused by different types of sexually transmitted diseases. Standardization is essential when comparing populations with different age structures or observing changes in the age structure of the same population over time. The ASR is calculated using the following formula:

ASR is defined as the rate per 100,000 persons, where *α*_*i*_ and *ω*_*i*_ represent the age-specific rate and the number of individuals in the *i*th age group, respectively, and *N* denotes the total number of age groups [6, 7].

EAPC refers to the average annual change in ASR over a specified time period, with the regression line fitting the natural logarithm of the ratio. The calculation of EAPC is estimated using the linear model: EAPC = *c* + *bX* − 1, where *y* = ln(ASR), *X* represents the calendar year, and *b* is the regression coefficient. The 95% confidence interval (CI) can also be derived from the regression model [8]. The trend of ASR is considered increasing (or decreasing) when both the EAPC estimate and its 95% CI are greater than 0 (or both are less than 0) [6, 9]. Additionally, 95% uncertainty intervals (95% UI) were computed for all indicators. In this study, the association between EAPC and ASR was assessed at the national level, and decomposition analysis was employed to visualize the factors influencing changes in the incidence of STI diseases and deaths from 1990 to 2021. The analysis focused on three factors (aging, demographic, and epidemiologic) that drive changes in the number of STIs, with epidemiologic changes referring to the underlying age- and population-adjusted mortality and morbidity rates [10].

The autoregressive integrated moving average model (ARIMA) incorporates both the autoregressive (AR) model and the moving average (MA) model. This model forecasts future values based on time and past data, expressed by the equation: *Y*_*t*_ = φ1*Y*_*t*−1_ + φ2*Y*_*t*−2_ + … + φ*pY*_*t*−*p*_ + *e*_*t−*θ_1*e*_t−1_…θ*qe*_*t*−*q*_. In this equation, (φ1*Y*_*t*−1_ + φ2*Y*_*t*−2_ + … + φ*pY*_*t*−*p*_ + *e*_*t*_) represents the AR model component, while (*e*_*t*−θ_1*e*_t−1_…θ*qe*_*t*−*q*_) signifies the MA model component. Here, *Y*_*t*−1_ denotes the observation from the (*t*−*p*)-cycle, *p* and *q* indicate the orders of the AR and MA models, respectively, and et represents the random error at cycle *t* [11]. The time series utilized in the ARIMA model should exhibit characteristics of a smooth random series with a zero mean. In the ARIMA model, φ_1_, φ_2_,…,φ*ₚ* are the parameters of the autoregressive (AR) component, which represent the linear relationship between the current data point and the past p data points. Similarly, θ₁, θ₂,…,θ*ₖ* are the parameters of the moving average (MA) component, indicating the influence of the error term on the current forecast. The integers *p* and *q* denote the orders of the AR and MA models, respectively, while *eₜ* represents the random error at time *t* [11]. The time series utilized in the ARIMA model should exhibit characteristics of a smooth random series with a mean of zero. The orders of the AR and MA components are determined using the autocorrelation function (ACF) and the partial autocorrelation function (PACF). Model evaluation is conducted through residual analysis and the Akaike information criterion (AIC), with the model yielding the smallest AIC value being selected to ensure optimal model fit and high forecasting accuracy.

The age–period–cohort model investigates the effects of age, period, and cohort on health outcomes. The age effect pertains to the risk of outcomes at various ages; the period effect denotes the impact of temporal changes on outcomes at each age; and the cohort effect reflects changes in outcomes among participants within the same birth cohort. This relationship is articulated through a log-linear regression model: log(*Y*_*i*_) = μ + α*agei + β*periodi + γ*cohorti + ε, where *Y*_*i*_ represents the standardized morbidity or mortality rate, α, β, and γ denote the coefficients for age, period, and cohort, respectively, μ signifies the intercept, and ε indicates the model residual. To derive the net effect of these three dimensions, the intrinsic estimator (IE) approach was integrated with the age–period–cohort model [12].

### Cross-country inequalities analysis

The slope index of inequality and the concentration index serve as standardized measures of absolute and relative gradient imbalances, respectively. The slope index of inequality is derived from regression analysis that correlates a country's age-standardized incidence rates (ASIRs) or disability-adjusted life years (DALYs) with its relative position on the socio-demographic index (SDI), defined by the midpoint of the population in the cumulative distribution ordered by the SDI [13]. Heteroscedasticity was assessed using weighted regression models. Concentration indices were computed by numerically integrating the area beneath the Lorenz curve, aligning the cumulative proportion of ASIRs or age-standardized mortality rates (ASMRs) with the cumulative distribution of the population sorted by SDI [14].

Statistical significance was considered when the *P* value was ≤ 0.05 (two-sided test); to assess trends in STI disease burden, a joint-rater regression analysis was performed using R software version 4.3.0.

## Results

### Global STD burden

**Fig. 1:**
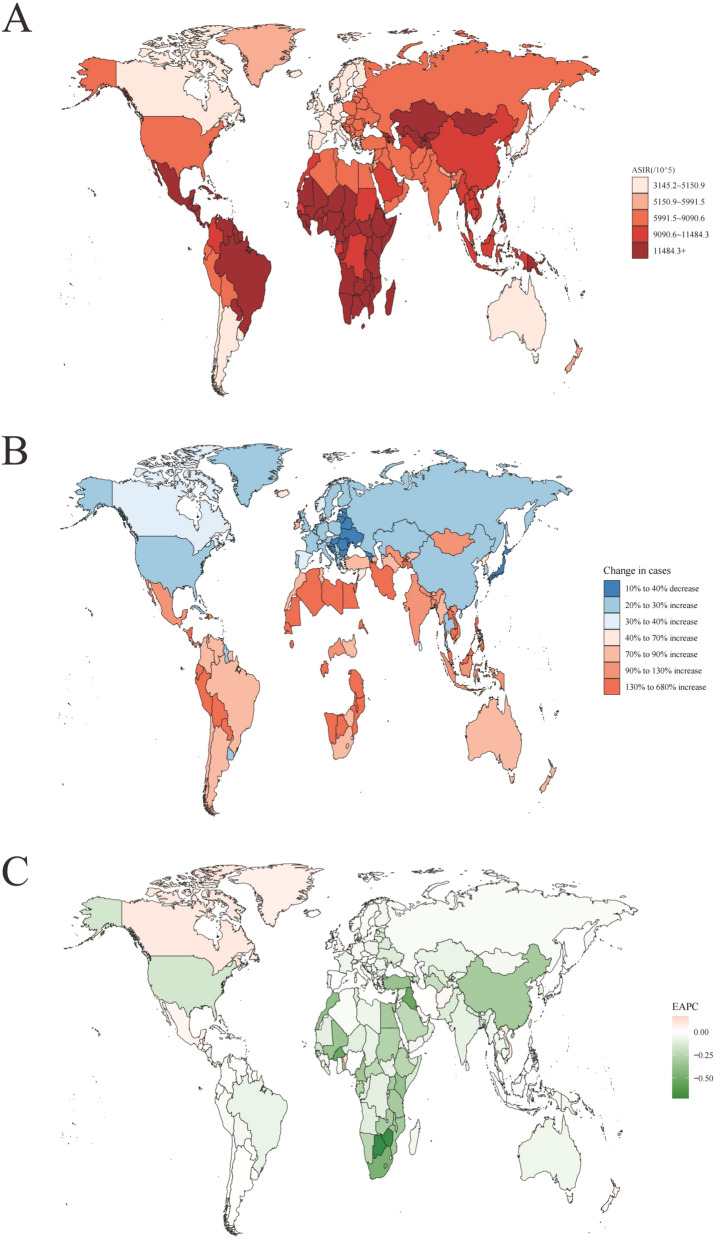
The global disease burden of sexually transmitted diseases for both sexes in 204 countries and territories. **A** The ASIR of sexually transmitted diseases in 2021; **B** the relative change in incident cases of sexually transmitted diseases between 1990 and 2021; **C** The EAPC of sexually transmitted diseases. The global disease burden of sexually transmitted diseases for both sexes in 204 countries and territories. Transmitted diseases ASIR from 1990 to 2021. EAPC estimated annual percentage change. The global disease burden of sexually transmitted diseases for the global disease burden of sexually transmitted diseases for both sexes in 204 countries and territories

**Fig. 2:**
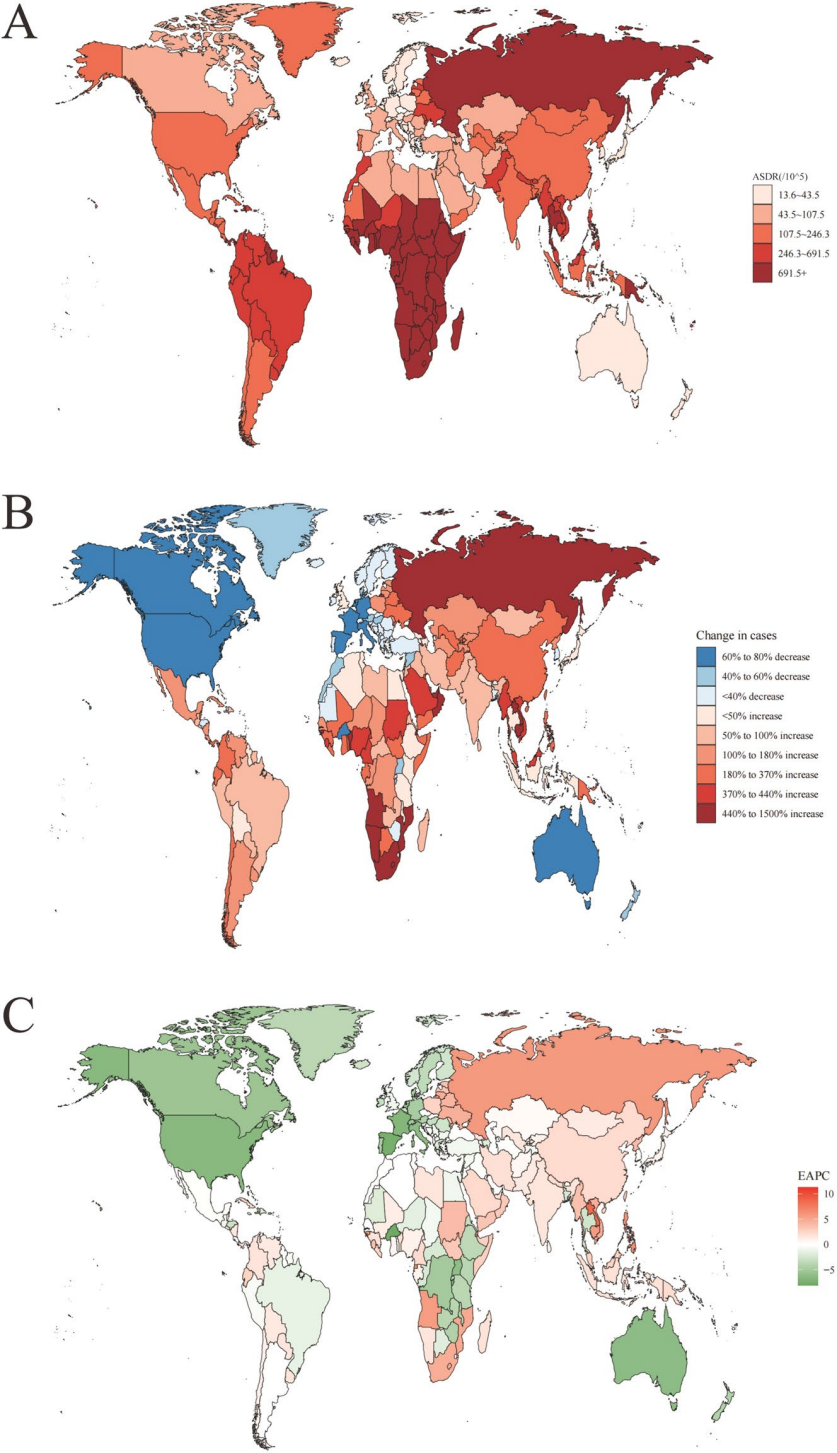
The global disease burden of sexually transmitted diseases for both sexes in 204 countries and territories. Transmitted diseases in 2021; **A** The ASDR of sexually transmitted diseases in 2021; **B** the relative change in DALYs cases of sexually transmitted diseases between 1990 and 2021; **C** The EAPC of sexually transmitted diseases DALYs from 1990 to 2021. Transmitted diseases DALYs from 1990 to 2021. EAPC estimated annual percentage change. The global disease burden of sexually transmitted diseases for the global disease burden of sexually transmitted diseases for both sexes in 204 countries and territories

The age-standardized incidence rate (ASIR) and disability-adjusted life years (DALYs) for sexually transmitted disease (STDs) exhibited significant global variation (Figs. 1 and 2). In 2021, South Africa recorded the highest ASIR at 21,590.1 per 100,000 people, followed by the United Republic of Tanzania at 18,358.1 per 100,000 people, and Mozambique at 18,289.6 per 100,000 people. Conversely, Belgium reported the lowest ASIR at 3145.2 per 100,000 people. In terms of absolute incidence, China had the highest number of STI cases in 2021, with an estimated incidence of 148,305,369.2, followed by India with 94,425,747.2, and Indonesia with 34,123,344.1 cases. Niue registered the lowest incidence with 192.8 cases (Table S1). Regarding DALYs in 2021, Lesotho had the highest rate at 22,820.2 per 100,000 people, followed by Eswatini at 16,447.2 per 100,000 people, and Botswana at 11,576.3 per 100,000 people. Finland reported the lowest DALYs rate at 13.6 per 100,000 people. In absolute terms, South Africa recorded the highest number of STI-related DALYs in 2021, totaling 59,374,490.4 years, followed by Nigeria at 4,602,152.3 years and India at 3,250,671.5 years (Table S2).

**Fig. 3:**
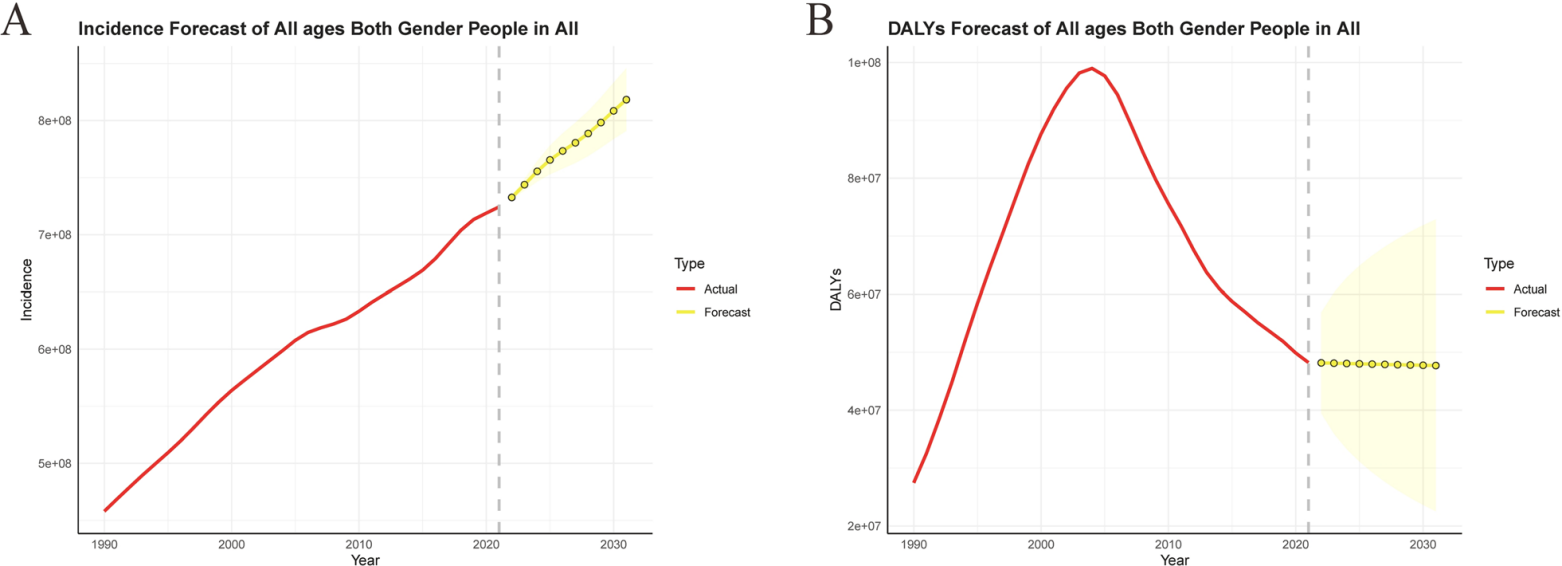
Predicted trends of sexually transmitted diseases incidence (**A**) and DALYs (**B**) in the next decade (2021–2031). Red lines represent the true trend of sexually transmitted diseases incidence and DALYs during 1990–2021; yellow dot lines and shaded regions represent the predicted trend and its 95% CI. Red lines represent the true trend of sexually transmitted diseases incidence and DALYs during 1990–2021; yellow dot lines and shaded regions represent the predicted trend and its 95% CI

The results of the ARIMA model indicate a persistent increase in the incidence of sexually transmitted diseases (STDs), with projections suggesting that the number of STDs will escalate from 725 million in 2021 to 820 million by 2031 (Fig. 3 and Table S3). Conversely, after reaching a peak in 2004 at 98,969,000, the disability-adjusted life years (DALYs) attributable to STDs have exhibited a downward trajectory and are anticipated to continue this decline, decreasing from 48.22 million in 2021 to 47.704 million by 2031 (Fig. 3 and Table S3).

**Fig. 4:**
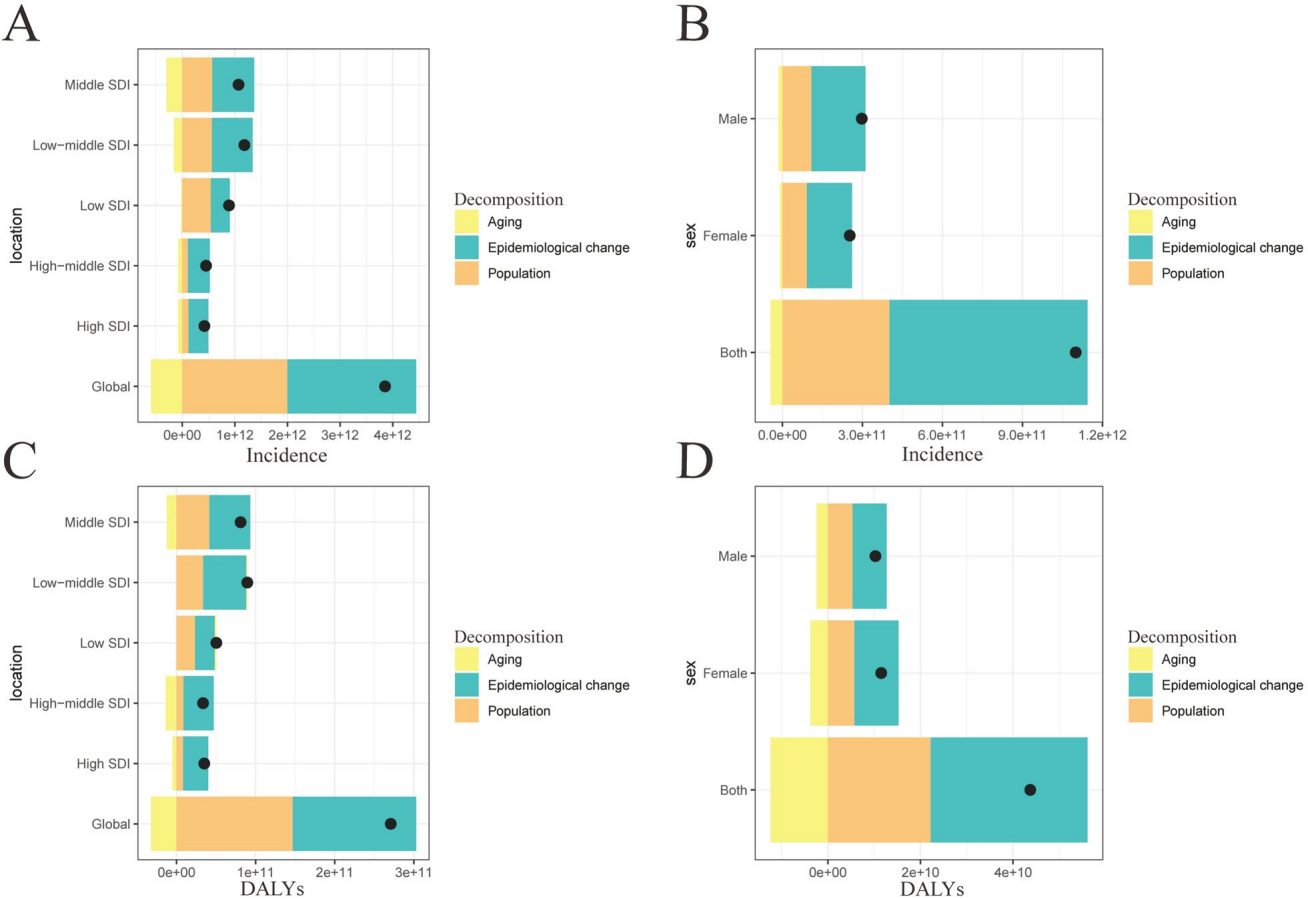
Changes in STD incidence and DALYs from 1990 to 2021 according to population-level determinants of population growth, aging, and epidemiological change across different socio-demographic index quintiles and by sex. **A** Decomposition of STD incidence by SDI quintile; **B** Decomposition of STD incidence by sex; **C** Decomposition of DALYs by SDI quintile; **D** Decomposition DALYs by sex. The black dot represents the overall value of change contributed by all three components. Epidemiologic changes refer to the evolving patterns of STD incidence and outcomes, independent of demographic shifts, as reflected in age- and population-standardized incidence and mortality. SDI, socio-demographic index

From 1990 to 2021, the increase in global incidence was primarily influenced by epidemiological changes (63.42%), followed by population growth (51.94%). The most significant rise in sexually transmitted diseases (STDs) occurred in the low-middle socio-demographic index (SDI) region, where epidemiological changes contributed the most (65.26%), followed by population growth (48.02%) (Fig. 4A; Table S4). A similar trend was observed at the gender level, with a greater increase in STDs incidence among males (Fig. 4B; Table S4). Additionally, the global increase in disability-adjusted life years (DALYs) was also more significantly influenced by epidemiological changes (57.65%) compared to population growth (54.22%). In the low-middle SDI region, DALYs saw the largest increase, with epidemiological changes being the predominant factor (60.62%) (Fig. 4C; Table S4). Likewise, at the gender level, the increase in DALYs was more pronounced for female STDs (Fig. 4D; Table S4).

**Fig. 5:**
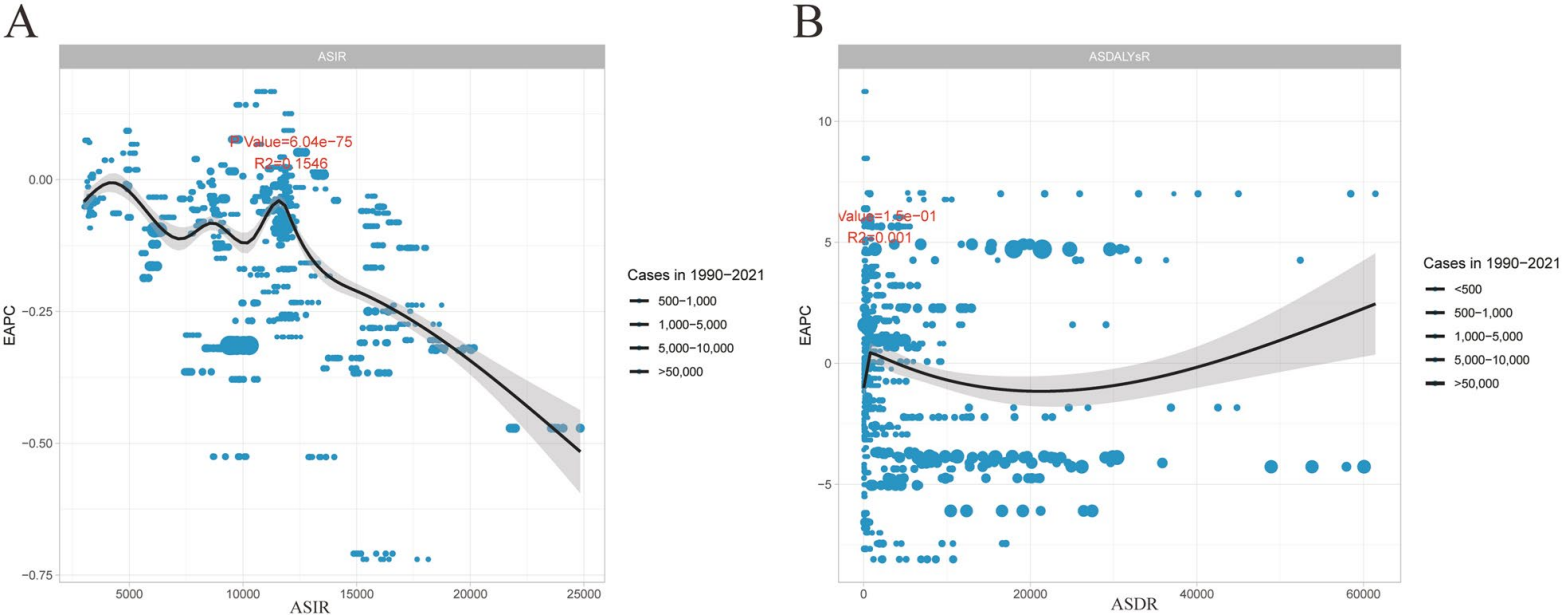
The correlation between EAPC and (**A**) ASIR and (**B**) DALYs rate. Circles represent cases, with larger circles indicating higher cases. The *R*^2^ and the *R*(2) and *P* values were derived from Pearson correlation analysis

A significant correlation was observed between the estimated annual percentage change (EAPC) and both the age-standardized incidence rate (ASIR) and disability-adjusted life years (DALYs) rate (*P* < 0.05; 2021). When ASIR is less than 12,500, EAPC exhibits considerable fluctuations, demonstrating dynamic variability; however, it generally trends downward (EAPC < 0). In contrast, when ASIR exceeds 12,500, EAPC consistently shows a decreasing trend (*P* = 6.04e−75, *R*^2^ = 0.1546). No correlation was found between EAPC and the DALYs rate (*P* = 1.5e−01, *R*^2^ = 0.001) (Fig. 5).

**Fig. 6:**
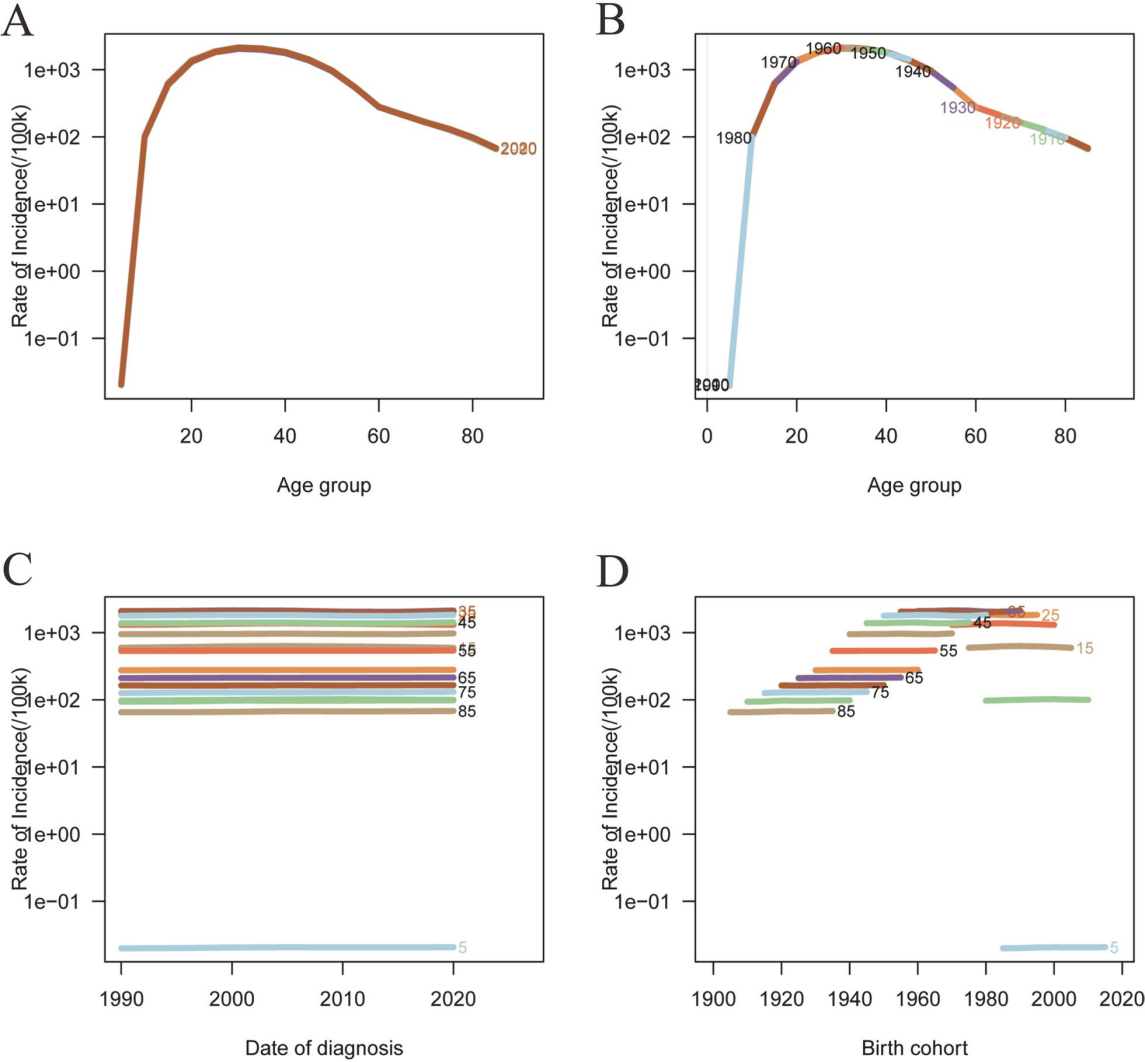
Trends in age-specific, period-specific, and cohort-specific STD incidence rates in global. **A**, **B** Age-specific incidence rates of STD by year and birth cohort; **C** Period-specific STD incidence rates across age groups; **D** Cohort-specific STD incidence rates by birth cohort. **A**, **B** Age-specific incidence rates of STD by year and birth cohort; **C** period-specific STD incidence rates across age groups; **D** cohort-specific STD incidence rates by birth cohort

**Fig. 7:**
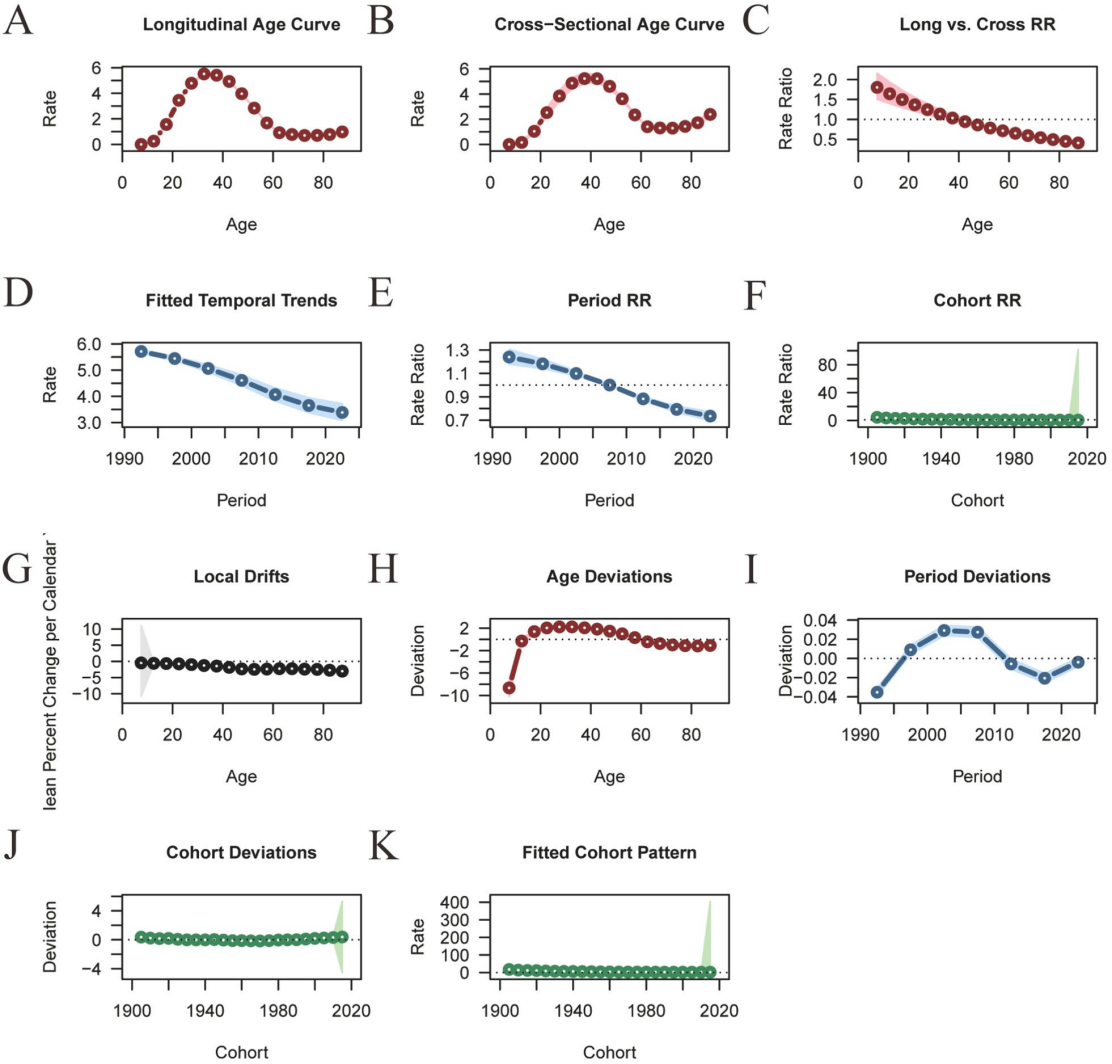
Age-period-cohort analysis of global STD incidence from 1990 to 2021. **A**, **B** Longitudinal and cross-sectional age curves of STD incidence (**C**); (**A**, **B**) longitudinal and cross-sectional age curves of STD incidence; **C** comparison of longitudinal vs. cross-sectional rate ratios; **D**, **E** fitted temporal trends and period rate ratios; **F** cohort rate ratios; **G**, **H** local drifts and age deviations; **I**, **J** period deviations and cohort deviations; **K** fitted cohort pattern

Figures 6 and 7 illustrate the age–period–cohort effects on the incidence of sexually transmitted diseases (STDs). The incidence of STDs peaks between the ages of 20 and 40 years. Overall, the age–period–cohort analysis of STD incidence demonstrates an initial increase followed by a decrease, with the highest incidence observed in the 30–40 age group (Fig. 6; Table S5). The relative risk (RR) value decreased from 1.2397 (95% CI: 1.1773–1.3055) in mid-1992 to 0.7344 (95% CI: 0.6973–0.7734) in mid-2022. The birth cohort analysis reveals a significantly higher risk of early STDs onset for older cohorts (RR cohort (1905) = 4.4912, 95% CI: 4.0862–4.9364), while the most recent cohort exhibits a lower risk (RR cohort (2015) = 0.5975, 95% CI: 0.0035–101.994) (Fig. 6; Table S5).

**Fig. 8:**
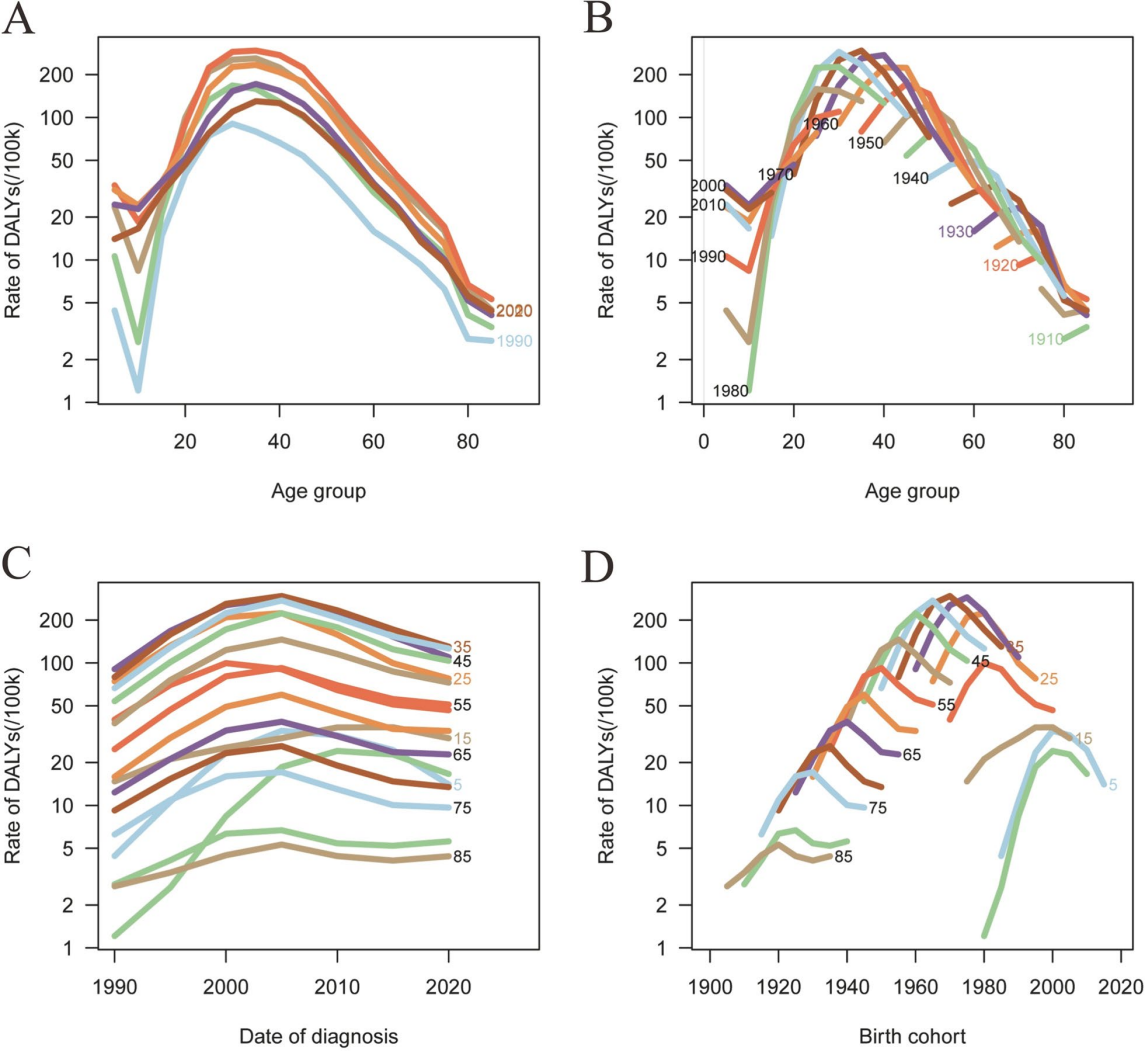
Trends in age-specific, period-specific, and cohort-specific STD DALYs rates in global. **A**, **B** Age-specific incidence rates of STD by year and birth cohort; **C** period-specific STD incidence rates across age groups; **D** cohort-specific STD incidence rates by birth cohort. **A**, **B** Age-specific incidence rates of STD by year and birth cohort; **C** period-specific STD incidence rates across age groups; **D** cohort-specific STD incidence rates by birth cohort

**Fig. 9:**
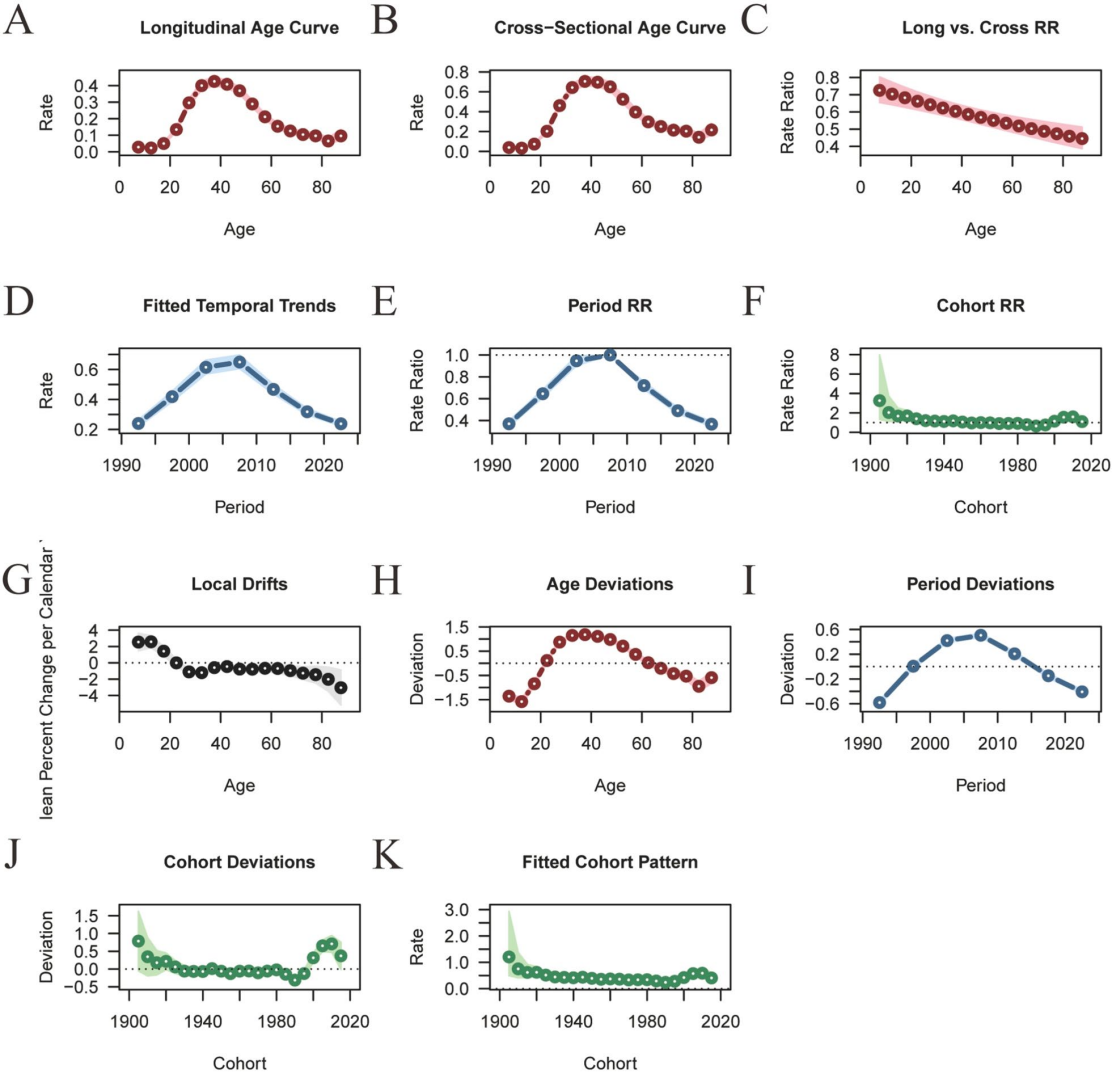
Age–period–cohort analysis of global STD DALYs rate from 1990 to 2021. **A**, **B** Longitudinal and cross-sectional age curves of STD DALYs rate; **C** comparison of longitudinal vs. cross-sectional rate ratios; **D**, **E** fitted temporal trends and period rate ratios; **F** cohort rate ratios; **G**, **H** local drifts and age deviations; **I**, **J** period deviations and cohort deviations; **K** fitted cohort pattern

Figures 8 and 9 illustrate the age–period–cohort effect of disability-adjusted life years (DALYs) for sexually transmitted diseases (STDs). The rate of DALYs for STIs peaks between the ages of 30 and 40 years, exhibiting an overall trend of increase followed by a decrease (Fig. 8; Table S5). The relative risk (RR) increased from 0.3696 (95% CI: 0.3444–0.3967) in mid-1992 to 1 (95% CI: 1–1) in mid-2007, before decreasing to 0.3662 (95% CI: 0.3436–0.3904) in mid-2022 (Fig. 9; Table S5).

**Fig. 10:**
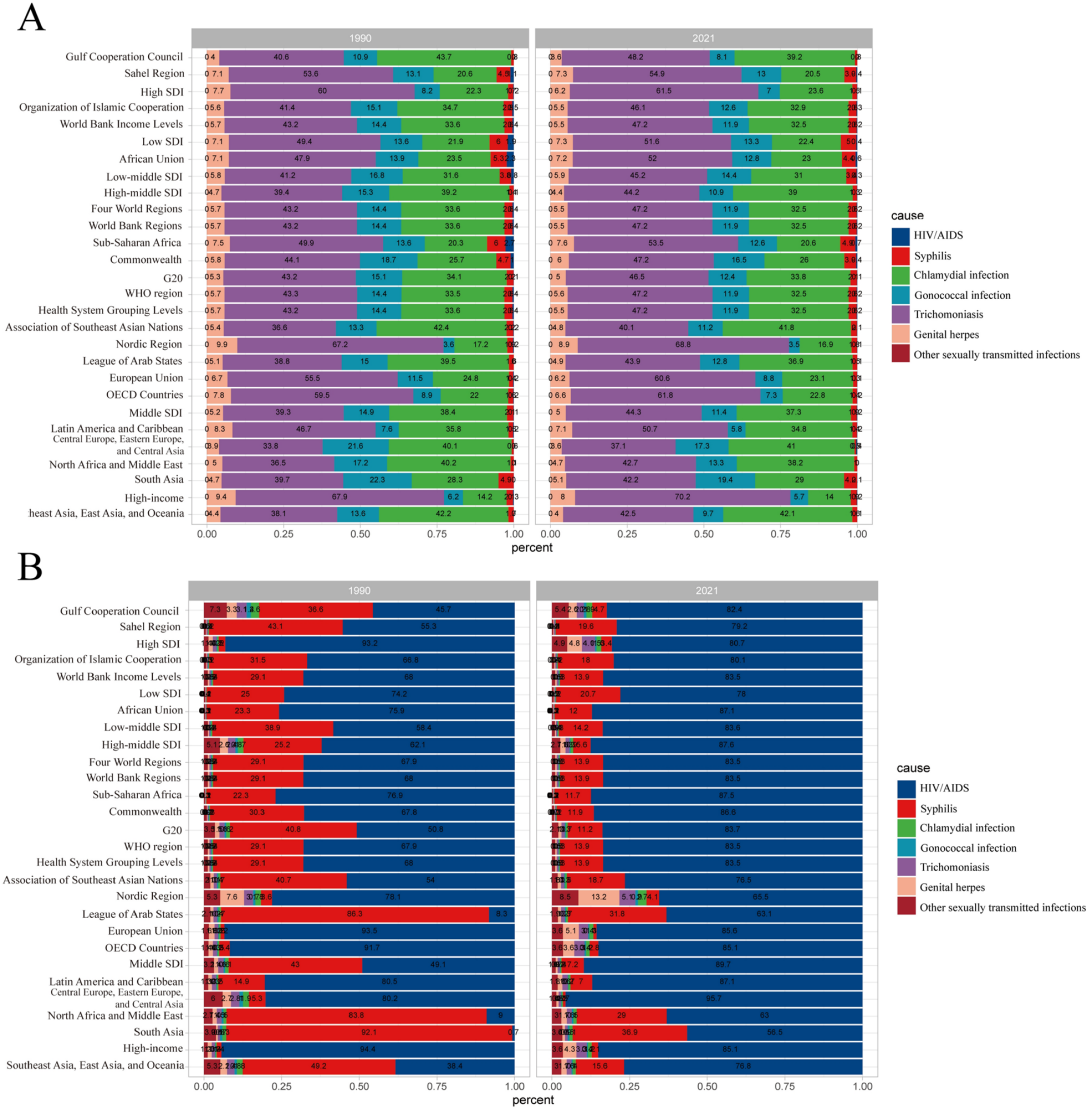
Contribution of STD incidence (**A**), and DALYs (**B**), both sexes and by region, in 1990 and 2021

The global burden of sexually transmitted diseases (STDs) in 1990 and 2021 is illustrated as follows: across all regions, trichomoniasis has consistently shown the highest incidence rates, followed by chlamydial infections, gonococcal infections, and genital herpes. Notably, the incidence of trichomoniasis has increased in all regions since 1990 (Fig. 10A). In terms of disability-adjusted life years (DALYs), HIV/AIDS remains the predominant cause, followed by syphilis. Compared to 1990, the DALYs attributed to HIV/AIDS have also risen; however, in 1990, only a few countries reported syphilis as the leading cause of DALYs (Fig. 10B).

**Fig. 11:**
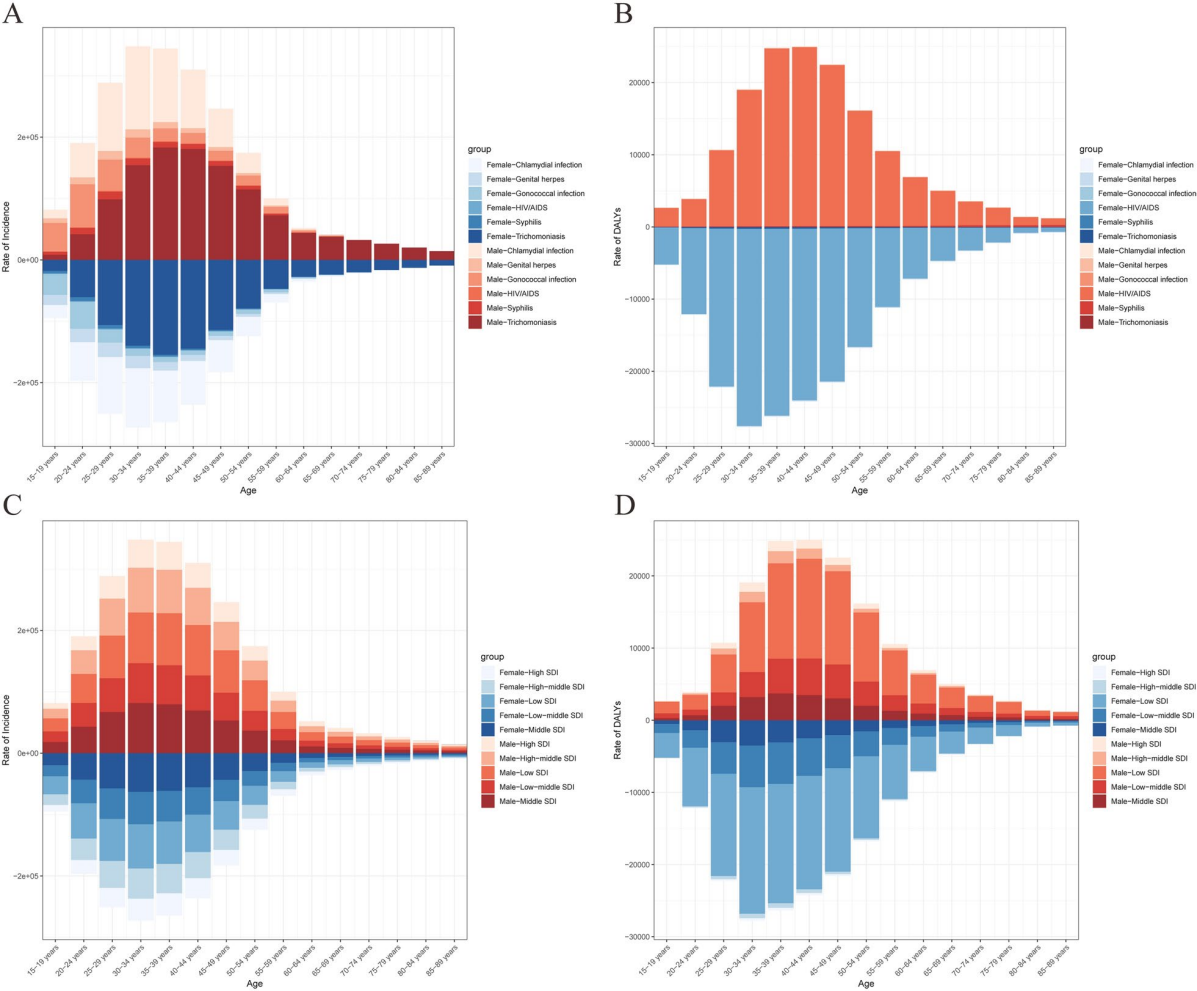
Age-specific incidence rates of STD by cause and sex (**A**) and age-specific incidence rates of STD by location and sex (**B**). Age-specific DALYs rates of STD by cause and sex (**C**) and age-specific DALYs rates of STD by location and sex (**D**)

Stratified analysis by gender and age cohort revealed distinct epidemiological patterns in sexually transmitted diseases (STDs). In individuals aged ≥ 25 years, trichomoniasis predominated as the primary STDs diagnosis in both genders, followed by chlamydial infection as the secondary etiology. Notably divergent patterns emerged in younger male populations, where gonococcal infections demonstrated significantly higher prevalence rates. Female adolescents exhibited comparable incidence rates across trichomoniasis, gonococcal infections, and chlamydial infections (Fig. 11A). Comprehensive disability-adjusted life year (DALY) analysis demonstrated HIV/AIDS as the principal contributor to STD-related disease burden across all demographic strata, regardless of gender or age group (Fig. 11B). Stratified analysis by gender, age group, and socio-demographic index (SDI) reveals that sexually transmitted diseases (STDs) are predominantly concentrated in regions with low to middle SDI levels (including middle, low-middle, and low SDI regions) across all genders and age groups (Fig. 11C). The disability-adjusted life years (DALYs) associated with sexually transmitted infections (STIs) are primarily observed in low SDI regions, showing no significant gender or age group differences, followed by low-middle SDI regions (Fig. 11D).

**Fig. 12:**
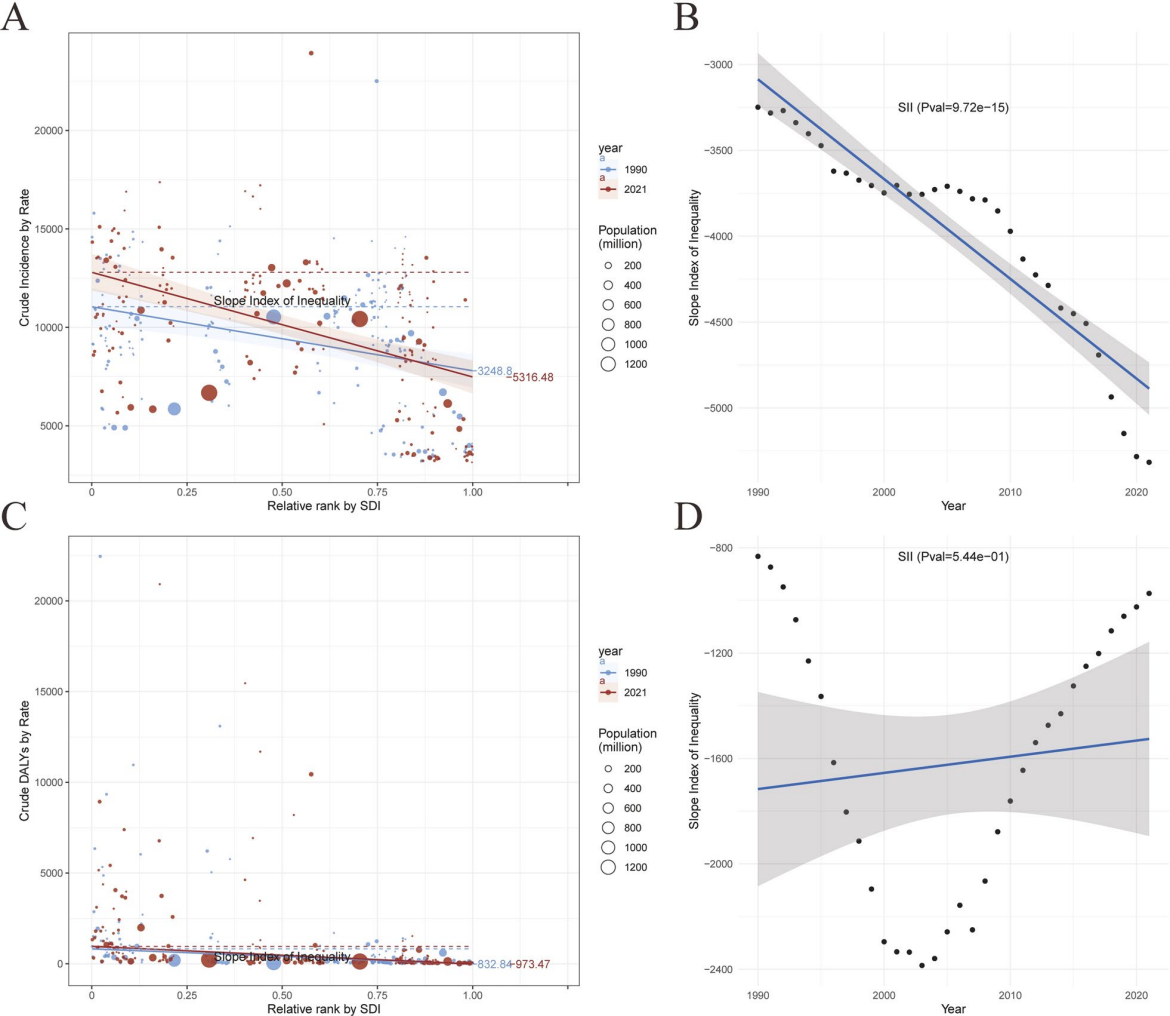
Trends in crude STD incidence and STD-related DALYs by SDI and SII from 1990 to 2021. **A** Crude STD incidence rate by relative SDI rank, with the A crude STD incidence rate by relative SDI rank, with the slope index of inequality (SII) for 1990 and 2021; **B** Change in SII over time for crude STD incidence; C crude STD-related DALYs rate by relative SDI rank, with the SII for 1990 and 2021. **C** Crude STD-related DALYs rate by relative SDI rank; **D** Change in SII over time for crude STD-related DALYs. Circle sizes represent population size for different countries and territories. Circle sizes represent population size for different countries and territories

Compared to 1990, the inequality in the incidence of sexually transmitted diseases (STDs) across countries and regions with varying socio-demographic index (SDI) levels decreased by 2021. The overall trend indicates a decline in incidence rates as SDI levels increase. Notably, the absolute value of the slope index of inequality (SII) in 2021 (SII = − 5316.48) was significantly lower than that in 1990 (SII = − −3248.8). This suggests a year-on-year decrease in SII, with the regression fitting results being statistically significant (*P* = 9.72e−15 < 0.05) (Fig. 12A, Table S6). This indicates that the burden of STDs incidence is gradually decreasing in terms of inequality among countries and regions with different levels of social development. In a similar vein, the overall inequality in disability-adjusted life years (DALYs) also exhibited a decreasing trend; however, the magnitude of this decrease was not statistically significant (Fig. 12C, Table S6). Overall, the inequality in the burden of DALYs due to STDs among countries and regions with varying levels of social development is gradually diminishing.

## Discussion

The purpose of this study is to assess the incidence and disability-adjusted life years (DALYs) of sexually transmitted diseases (STDs), including syphilis, HIV, chlamydia, trichomoniasis, genital herpes, gonorrhea, and others, by globe, region, and country using data from the Global Burden of Disease (GBD) of 2021. This paper highlights the focus populations for the incidence of STDs and DALYs from 1990 to 2021, examining epidemiological changes and differences in the burden of incidence in countries with varying socio-demographic indices. It emphasizes the need to develop preventive measures tailored to different age groups and socio-demographic indices.

Epidemiological changes and global population growth are critical factors influencing the prevalence of STDs, alongside socio-demographic indices. Consistent with previous studies, we found that the age-standardized rate (ASR) for trichomoniasis remained stable and high from 1990 to 2021, particularly in low-income areas [15–17], with the highest ASR recorded in South Africa. In 2021, syphilis prevalence was highest in South Africa, while Brazil exhibited the largest increase in syphilis ASR, followed by Cape Verde and Greece. This finding corroborates previous research [15], including a study by Juliana et al., which reported [18] that syphilis in pregnancy—whether asymptomatic or symptomatic during prenatal, labor, or puerperium stages—continues to pose significant public health challenges in Brazil, particularly in cases of congenital syphilis, which includes stillbirths, neonates, or miscarriages in women with untreated or inadequately treated syphilis.

We found that the highest number of morbidities and deaths globally are attributable to HIV/AIDS, and that the increase in morbidity may be related to its high-risk sexual behaviors, including: unprotected sex [19], such as failure to use contraception (condom use); alcohol- or drug-influenced sex; or commercialized sex [20]. At the same time, people who consume alcohol show lower willingness to use condoms [21]; and the high mortality rate of HIV/AIDS may be due to the inability of combination antiretroviral therapy (ART) to eradicate the HIV virus, resulting in multiple infections due to host immunodeficiency [22].

Our analysis indicates that globally, from 1990 to 2021, the age-standardized rates (ASRs) for Trichomonas vaginalis, genital herpes, and gonococcus exhibited a decreasing trend. In contrast, the ASRs for syphilis and chlamydia demonstrated an increasing trend, with the most significant rise in chlamydia ASRs observed in the Maldives, followed by the Cook Islands and Colombia. Notably, despite the downward trend, the ASRs for trichomoniasis and genital herpes remain particularly high in the lower quintiles of the socio-demographic index (SDI) countries. This phenomenon may be attributed to their reliance on integrated management strategies, which tend to be less specific and result in over-treatment with antibiotics [3, 23]. Consistent with previous research [16], Africa continues to be a critical region for the prevention and control of trichomoniasis and genital herpes.

Furthermore, we observed a trend towards younger populations being affected by sexually transmitted diseases (STDs). The incidence of STDs peaked around the age of 27.5 years, particularly among individuals born between 1950 and 1970, with a gradual increase noted in those born in subsequent years. This trend may correlate with high-risk sexual behaviors prevalent in these cohorts. To effectively control STDs, it is imperative to address and reduce risky sexual behaviors, as highlighted in previous studies [24]. Adolescent boys, in particular, tend to have more sexual partners than adults, leading to a significantly increased risk of STDs among adolescents [25, 26]. This underscores the importance of school-based education, especially in sexual health, in fostering and protecting healthy sexual behaviors among young people. Encouraging a reduction in the number of sexual partners and promoting the use of contraception can significantly mitigate the incidence of sexually transmitted infections [27–31].

Our epidemiological surveillance reveals persistently elevated STD prevalence rates in Africa, standing out as the highest global burden. Current evidence strongly associates this phenomenon with Africa's unique sociocultural matrix [32]^.^ The continent's cultural pluralism and religious diversity significantly influence reproductive health paradigms through multiple pathways: 1. behavioral determinants (a. high prevalence of extramarital/commercial sexual activities b. persistent practice of polygynous unions, c. cultural adherence to menstrual-phase sexual practices and initiation rituals) 2. structural drivers (a. limited contraceptive accessibility (contraception prevalence rate < 30% in 18 countries), b. migration patterns disrupting family structures, c. educational deficits in sexual health literacy). Empirical studies identify many synergistic factors amplifying STI incidence [33].

Implementation challenges and precision public health solutions, while our research corroborates previous findings [34] advocating targeted antenatal interventions, operational barriers persist: current STI screening coverage for pregnant women in South Africa remains suboptimal (CT: 58%, NG: 51%, TV: 37%) due to: molecular diagnostic capacity gaps and decentralized healthcare resource allocation. Then, proposed mitigation strategies include: 1. development of novel risk stratification instruments combining: geospatial mapping of high-transmission zones and Behavioral risk scoring algorithms. 2. Implementation of multiplex PCR testing platforms to optimize: diagnostic yield (sensitivity > 95%) and cost-effectiveness (USD 2.8/test). This precision public health approach could increase screening efficiency by 40–60% while maintaining 90% sensitivity thresholds [33]. 3. Enhancing sexual education and awareness plays a pivotal role in reducing the incidence of sexually transmitted diseases.

Our study has several limitations. The accuracy and robustness of the Global Burden of Disease (GBD) estimates are heavily reliant on the quality and quantity of data utilized in the modeling process. For instance, unavoidable data loss can adversely impact the accuracy of the findings. Additionally, the sensitivity of sexually transmitted diseases (STDs) diagnosis varies across countries, complicating the assessment of whether the estimates truly reflect local prevalence levels. Furthermore, the GBD database comprises data from various countries, which can inevitably introduce measurement bias.

## Supplementary Information

Supplementary Material 1

## Data Availability

No datasets were generated or analysed during the current study.
