# Supplementary material for: Trends in the incidence of common sexually transmitted infections at the global, regional and national levels, 1990–2021: results of the Global Burden of Disease 2021 study
Source: Trop Med Health. 2025 May 16;53:70. doi: 10.1186/s41182-025-00744-2 (PMC12084928; doi:10.1186/s41182-025-00744-2)
Supplement: Supplementary file 1 — Supplementary Material 1 [file 41182_2025_744_MOESM1_ESM.docx]

Table S1. The STD Incident cases and age standardized incidence of STD caused by different location from 1990 to 2021.

| **Cause** | **Location** | **1990** | | **2021** | | **1990-2021** |
| --- | --- | --- | --- | --- | --- | --- |
|  |  | Incident cases  No. ×10^3^ (95% UI) | ASIR per 100,000  No. (95% UI) | Incident cases  No. ×10^3^ (95% UI) | ASIR per 100,000  No. (95% UI) | EAPC  No. (95% CI) |
| Chlamydial infection | Afghanistan | 207179.8 (148906.7-275104.2) | 2973.4 (2140.6-4124.4) | 816519.4 (567835.4-1133155) | 2964.9 (2115.3-4235.8) | 0.27 (-3.89-4.6) |
| Chlamydial infection | Albania | 114857.8 (78039.8-164328.7) | 3355.4 (2360.1-4704.8) | 88169.9 (63114.7-125568.1) | 3302.6 (2335.8-4706.6) | -0.11 (-4.53-4.51) |
| Chlamydial infection | Algeria | 724928.5 (513471.9-994999.6) | 3196 (2340.5-4385.8) | 1297669.3 (932144.7-1825649.2) | 2770.3 (1980.6-3835.4) | 0.49 (-3.58-4.74) |
| Chlamydial infection | American Samoa | 1419.3 (996-1963.9) | 2943.9 (2121.6-4086.2) | 1365.4 (1003.2-1837) | 2901 (2101.8-3978.4) | 0.96 (-3.19-5.29) |
| Chlamydial infection | Andorra | 239.6 (169.4-337.6) | 362 (257.4-495.3) | 330.1 (244.7-448.9) | 358.6 (258.2-489.1) | -0.13 (-3.69-3.57) |
| Chlamydial infection | Angola | 193630.7 (134991.7-277006) | 2213.1 (1584-3172.9) | 585586.8 (416376-820876.9) | 2146.3 (1552.5-2987.1) | 0.43 (-3.19-4.19) |
| Chlamydial infection | Antigua and Barbuda | 2919.9 (2047.2-4171.8) | 4673 (3386.1-6516.3) | 4606.2 (3405.1-6434.6) | 4659.7 (3413.8-6489) | -0.21 (-4.12-3.85) |
| Chlamydial infection | Argentina | 273954.2 (202060.8-372362.1) | 863.1 (637.8-1176.9) | 428672.4 (315307.1-603658.3) | 886.9 (648.5-1245) | -0.06 (-3.49-3.5) |
| Chlamydial infection | Armenia | 186274.5 (131546.2-267236.6) | 5179.7 (3799-7259.6) | 162893.9 (118970.1-229251.1) | 5147.8 (3796.7-7113.8) | 0.92 (-3.39-5.42) |
| Chlamydial infection | Australia | 164634.4 (119452.2-228126.2) | 902.4 (657.7-1246.6) | 229637.9 (168849.6-318068.7) | 892.5 (642.3-1233.5) | -0.13 (-3.7-3.57) |
| Chlamydial infection | Austria | 30132.4 (21994-40730) | 358.6 (259.8-484.6) | 32344.6 (24224.9-44019.9) | 359.8 (261.9-488.5) | -0.37 (-3.76-3.13) |
| Chlamydial infection | Azerbaijan | 398254.5 (278530-549935.2) | 5321.9 (3847.5-7289.2) | 627861.3 (450903-877973.6) | 5199.5 (3742.1-7205.8) | 0 (-4.41-4.62) |
| Chlamydial infection | Bahrain | 18912.5 (12774-27759.3) | 2922.7 (2103.4-4077.4) | 58326.3 (41753.7-84669.5) | 2841.1 (2041.2-4053.1) | -0.25 (-4.4-4.07) |
| Chlamydial infection | Bangladesh | 1779219.9 (1242052.1-2494635.2) | 1892.2 (1364.3-2649.2) | 3312820.5 (2367817.7-4619856.7) | 1882.1 (1356.5-2621.3) | 0.02 (-4.03-4.25) |
| Chlamydial infection | Barbados | 16032.2 (11363.4-22314.2) | 5876.8 (4264.1-8095.3) | 17212.9 (12665.6-23898.6) | 5862 (4240.7-8195.5) | -0.19 (-4.02-3.8) |
| Chlamydial infection | Belarus | 405585.1 (293339.1-580705) | 3733.5 (2708.9-5296.8) | 352220.5 (259360.2-502980.4) | 3729.1 (2697.4-5327.9) | 0.97 (-3.01-5.1) |
| Chlamydial infection | Belgium | 47932.5 (34939.6-64708.4) | 458.4 (332.1-619.6) | 50044.5 (37007.2-68168.9) | 459.1 (330.3-623.5) | -0.14 (-3.44-3.28) |
| Chlamydial infection | Belize | 7500.6 (5198.3-10362.1) | 4726.7 (3396.4-6620) | 21838.4 (15713.1-29918.8) | 4717.4 (3401.9-6503.4) | -0.21 (-3.99-3.72) |
| Chlamydial infection | Benin | 65041.9 (45268.6-90908) | 1678.9 (1204.3-2337.2) | 201774.2 (140432.8-278338.7) | 1688.8 (1211.4-2308) | -0.5 (-4.08-3.21) |
| Chlamydial infection | Bermuda | 3323.8 (2393.6-4745.8) | 4604.8 (3368.8-6466.1) | 2663.4 (1998.4-3732.2) | 4577.9 (3327.5-6413.5) | -0.48 (-4.39-3.6) |
| Chlamydial infection | Bhutan | 10530.1 (7455.6-14633.7) | 1850.3 (1367.9-2608.8) | 16293.1 (11499.4-23083.4) | 1830.3 (1316.9-2572.2) | 0.16 (-3.49-3.96) |
| Chlamydial infection | Bolivia | 150789.3 (108230.7-208523.5) | 2651.3 (1915.5-3691.8) | 327944 (233875.8-466711.4) | 2613.1 (1881.5-3697.6) | -0.33 (-4.02-3.51) |
| Chlamydial infection | Bosnia and Herzegovina | 164918.3 (118159.2-236234.3) | 3313.5 (2392.1-4737.8) | 103300 (75032.1-144274.8) | 3279.2 (2339.7-4639.1) | 0.06 (-4.35-4.67) |
| Chlamydial infection | Botswana | 36720.8 (25679.9-50309.8) | 3173.5 (2292-4292.8) | 84017.8 (59894.9-116015.4) | 3077 (2220.4-4196.3) | -1.25 (-4.85-2.49) |
| Chlamydial infection | Brazil | 6273237.8 (4466755.6-8910609.2) | 4248.1 (3113.9-6018) | 10348996.7 (7584090.7-14593083) | 4255.5 (3088.7-6034.2) | 0.06 (-3.86-4.14) |
| Chlamydial infection | Brunei | 3238.9 (2260-4645.1) | 1111.2 (814.9-1546.6) | 6223.6 (4448.7-8714.9) | 1108.3 (796.1-1534.5) | 0.32 (-3.39-4.18) |
| Chlamydial infection | Bulgaria | 310956.2 (225085.2-439216.1) | 3631.6 (2603.3-5155.4) | 227588.3 (165127.5-320110.4) | 3632.8 (2590-5092.7) | 0.4 (-3.8-4.78) |
| Chlamydial infection | Burkina Faso | 138992.4 (98970.8-192797.6) | 1902.9 (1373.7-2597.8) | 366096.2 (255586.1-510691.4) | 1861.8 (1322-2630) | -1.47 (-5.19-2.41) |
| Chlamydial infection | Burundi | 151477.7 (105977.9-209360.7) | 3212 (2320.8-4454.8) | 385009.3 (270263.4-536059.9) | 3215 (2285.3-4464) | -1.89 (-5.64-2.01) |
| Chlamydial infection | Cambodia | 399496.6 (283115.1-564664.1) | 4607.4 (3311.3-6480.4) | 848780.6 (597844.7-1213934.3) | 4618.8 (3297.4-6558.8) | -0.55 (-4.4-3.45) |
| Chlamydial infection | Cameroon | 178292.8 (125298.1-243184.1) | 2047.1 (1462.7-2822.7) | 598244.3 (416732.5-824630.1) | 2014.4 (1442.8-2787.9) | -0.82 (-4.42-2.91) |
| Chlamydial infection | Canada | 97994.4 (70823.5-134764.8) | 326.8 (236.1-437.9) | 116466.7 (84693.3-158404.8) | 324.9 (230.9-440) | 0.1 (-3.42-3.74) |
| Chlamydial infection | Cape Verde | 5275 (3556-7388.7) | 1840 (1310.1-2558.5) | 11589.6 (8241.7-16248) | 1807 (1311.4-2498.7) | -0.72 (-4.4-3.11) |
| Chlamydial infection | Central African Republic | 54475 (38269.8-76107.6) | 2323.2 (1661.8-3293.1) | 115375.7 (82104.8-162256.1) | 2325.5 (1682-3283.4) | -0.86 (-4.39-2.79) |
| Chlamydial infection | Chad | 91180 (63847.7-126180.8) | 1917.2 (1380-2667.9) | 259153.9 (180918-357776.6) | 1869.6 (1354.9-2594.6) | -0.59 (-4.2-3.15) |
| Chlamydial infection | Chile | 122459.7 (87174-172518.6) | 882.4 (636.2-1230.8) | 178712.8 (132552-250736.2) | 881.4 (642.3-1231.8) | 0.38 (-3.11-4.01) |
| Chlamydial infection | China | 51841033.7 (37450723.8-74183243.6) | 4068.1 (2988.4-5831.6) | 62674305.7 (46082548.4-90052843.7) | 4163.3 (3043.2-5995.6) | -0.08 (-4.2-4.23) |
| Chlamydial infection | Colombia | 998206.5 (723039.3-1382104.5) | 3027.2 (2230.5-4184.7) | 1714387.3 (1225934.2-2415099.8) | 3186.9 (2288-4481.4) | 0.24 (-3.66-4.29) |
| Chlamydial infection | Comoros | 12148.8 (8496.2-16761.3) | 3161.4 (2269-4369.8) | 24572.8 (17004.4-34174.8) | 3176 (2227.9-4438.6) | -0.17 (-4.66-4.53) |
| Chlamydial infection | Congo | 43835 (30761.8-61721.3) | 2159.2 (1554.7-3079.6) | 117750 (84164.4-167670.5) | 2163.4 (1553.5-3089.8) | -0.53 (-4.06-3.13) |
| Chlamydial infection | Cook Islands | 530 (378.9-715.5) | 2860.8 (2066.9-3890.1) | 511.9 (376.2-684.7) | 3090.3 (2227.9-4165.2) | 1.05 (-3.11-5.39) |
| Chlamydial infection | Costa Rica | 113032.1 (79776.8-157129.9) | 3705.1 (2714.6-5117.3) | 195115.3 (141462.5-274958.5) | 3734.7 (2713.4-5249.7) | 0.07 (-3.98-4.28) |
| Chlamydial infection | Cote d'Ivoire | 235544.7 (163507.5-328867) | 2175 (1550.9-3038.6) | 565065.9 (395936.6-784393.7) | 2121.4 (1518.9-2940.2) | -1.18 (-4.71-2.49) |
| Chlamydial infection | Croatia | 187848.2 (136091-267154.2) | 3649.5 (2625.9-5163) | 142241.8 (104439.7-200069) | 3648.4 (2624.4-5170.6) | 0.08 (-4.16-4.5) |
| Chlamydial infection | Cuba | 557620.9 (397612.2-774758.7) | 4631.3 (3349.9-6538.3) | 508405.8 (378674.5-706484) | 4616.3 (3332.2-6441.2) | 0.92 (-3.19-5.22) |
| Chlamydial infection | Cyprus | 2990.6 (2151.2-4043.3) | 366.1 (263.3-492.5) | 5423.7 (4056-7481.2) | 361.3 (264.3-492.3) | 0.04 (-3.63-3.85) |
| Chlamydial infection | Czech Republic | 340809.3 (249961.6-479631.1) | 3262.3 (2342.6-4571.1) | 320581.8 (234991.4-455877.3) | 3244.6 (2302.5-4575.4) | 0.39 (-3.87-4.83) |
| Chlamydial infection | Democratic Republic of the Congo | 648677.1 (453612.3-924486.2) | 2069 (1514.4-2963.5) | 1696554.2 (1208654.3-2441116.3) | 2097.1 (1513.9-3004.9) | -1.3 (-4.94-2.49) |
| Chlamydial infection | Denmark | 28817.5 (20906.2-39294.1) | 523.6 (377.7-715.2) | 29379.6 (21484.4-39539.2) | 523.2 (378.4-715.1) | -0.26 (-3.73-3.34) |
| Chlamydial infection | Djibouti | 12784.7 (8976.5-17645.4) | 3273.1 (2364.2-4617.8) | 45011.9 (31769.7-63399.8) | 3247 (2313.6-4555.6) | -0.19 (-3.94-3.7) |
| Chlamydial infection | Dominica | 3128.2 (2224.3-4372.6) | 4671.7 (3371.7-6586.8) | 3247.1 (2377.9-4564.9) | 4673.7 (3388.2-6594.7) | -0.21 (-4.16-3.9) |
| Chlamydial infection | Dominican Republic | 325630.3 (230284.9-455257.8) | 4720.6 (3407.2-6666.5) | 556916.5 (395904.2-779518.8) | 4690.8 (3363-6552.1) | -0.71 (-4.46-3.19) |
| Chlamydial infection | Ecuador | 210403.1 (147785.8-293337.8) | 2231.9 (1607-3115.4) | 415293 (299666.3-580702.4) | 2195.2 (1585.5-3060.3) | 0.54 (-3.22-4.44) |
| Chlamydial infection | Egypt | 1641919.8 (1203816.6-2232325.3) | 3157.3 (2339.5-4337.2) | 3119042.6 (2250617.7-4336630.8) | 2930.4 (2124.9-4074.1) | 0 (-4.33-4.53) |
| Chlamydial infection | El Salvador | 180857.9 (130241.9-252399.9) | 3839.8 (2774-5408.6) | 251682.6 (181086.7-348620) | 3786.5 (2733.9-5256.9) | -0.23 (-4.16-3.86) |
| Chlamydial infection | Equatorial Guinea | 7148.9 (5079.9-10025.8) | 2132.8 (1531.7-2996.3) | 33232 (22826.2-47119.6) | 2189.1 (1575.3-3132.1) | 0.57 (-2.97-4.24) |
| Chlamydial infection | Eritrea | 93375 (65999.3-129409.5) | 3212.1 (2315-4502.5) | 213858 (152464.6-298998.9) | 3203.5 (2341.9-4448.7) | -1.3 (-5.1-2.65) |
| Chlamydial infection | Estonia | 59476.9 (42914.2-84577.9) | 3696.3 (2614.3-5243) | 46508.3 (33986.1-66530.3) | 3693.5 (2680.9-5264) | 0.65 (-3.26-4.73) |
| Chlamydial infection | Ethiopia | 1337773.2 (944957-1857085.1) | 3326.2 (2416.7-4664.1) | 3253374.5 (2278508.3-4604211.8) | 3235 (2338.7-4643.8) | -1.38 (-5.11-2.5) |
| Chlamydial infection | Federated States of Micronesia | 2666.5 (1877.9-3615) | 2952.2 (2137.2-3979.3) | 3086.5 (2226.9-4179.6) | 2902.6 (2103.2-3928.4) | 0.99 (-3.14-5.3) |
| Chlamydial infection | Fiji | 44720.3 (33914.2-57394.6) | 5675.8 (4352.4-7299) | 44137.8 (32913.2-58604.2) | 4658.8 (3469.5-6169.7) | 0.08 (-4.15-4.5) |
| Chlamydial infection | Finland | 22244.7 (16421.5-30237.5) | 415.3 (301.5-563) | 21166.6 (15681.8-28370.9) | 416.4 (305.2-563.2) | -0.17 (-3.68-3.47) |
| Chlamydial infection | France | 211372.2 (153199.2-286578.1) | 352.1 (253.8-475.4) | 214559.7 (160471.9-286742.4) | 350.9 (255-469.2) | 0.07 (-3.21-3.47) |
| Chlamydial infection | Gabon | 18513.4 (12794.5-26093.5) | 2181.2 (1577.7-3102.3) | 38075 (26923.3-53635.6) | 2128 (1520-3006.3) | -0.52 (-4.04-3.14) |
| Chlamydial infection | Georgia | 293256.7 (213414.1-412930.7) | 5189.1 (3782.2-7231.6) | 175666.3 (128725.1-244278.5) | 5101 (3694.4-7006.1) | 0.67 (-3.52-5.04) |
| Chlamydial infection | Germany | 488908.9 (358955.9-672136.6) | 559.2 (407.8-770.7) | 456879.2 (339151.9-618651.8) | 563.6 (409.1-769.8) | -0.12 (-3.64-3.52) |
| Chlamydial infection | Ghana | 436679.9 (305589.4-600175.1) | 3323 (2385.1-4625.8) | 1108179.4 (782365.4-1531783.5) | 3221.3 (2334.1-4438.2) | -0.48 (-4.16-3.34) |
| Chlamydial infection | Greece | 92257.3 (67059.7-125777.5) | 868 (623.3-1190.6) | 80957.1 (59741.6-109671.1) | 840.1 (618.9-1149.3) | 0.58 (-2.98-4.27) |
| Chlamydial infection | Greenland | 343.5 (244.4-480.5) | 507.4 (367.9-690.4) | 283.1 (204.7-379.3) | 490.3 (351-664.7) | 0.04 (-3.4-3.61) |
| Chlamydial infection | Grenada | 3540.6 (2490.1-4892.5) | 4702.6 (3404.3-6538.9) | 5139.2 (3711.8-7084) | 4689.1 (3411.1-6538.7) | -0.4 (-4.39-3.75) |
| Chlamydial infection | Guam | 4221 (2917.3-5839) | 2696.5 (1910.3-3675.5) | 4336.8 (3153.7-5856) | 2805.4 (2007.4-3791.8) | 0.1 (-4.06-4.44) |
| Chlamydial infection | Guatemala | 254441.3 (193647.2-335859.4) | 3738.6 (2875.3-4955) | 603835.6 (431790.9-822451.3) | 3694.8 (2688.6-5076.2) | -0.51 (-4.52-3.67) |
| Chlamydial infection | Guinea | 93878 (66810.9-131492.6) | 1911.3 (1377.9-2679.4) | 218775.4 (153088.4-299855.3) | 1858.4 (1340-2572.7) | -0.32 (-3.93-3.42) |
| Chlamydial infection | Guinea-Bissau | 15767.6 (10999.5-22054) | 1892.5 (1373.1-2624) | 35837.4 (25124-49073.2) | 1853.9 (1341.9-2516.7) | 0.28 (-3.3-3.99) |
| Chlamydial infection | Guyana | 36778.2 (25616.5-51584.2) | 4789.6 (3399.9-6696.8) | 38104 (27389.2-53489.2) | 4760.1 (3424.1-6702.6) | -0.3 (-4.05-3.59) |
| Chlamydial infection | Haiti | 231858.3 (163436.7-322662.3) | 4103.3 (2978.6-5765.5) | 550749.5 (386611.1-779856.9) | 4054.6 (2897.1-5683.4) | -0.6 (-4.18-3.11) |
| Chlamydial infection | Honduras | 149181.5 (105895-204322.2) | 3884.9 (2796.4-5338.9) | 404201.1 (285313.2-556542.4) | 3833.6 (2746.4-5318.8) | -0.35 (-4.55-4.04) |
| Chlamydial infection | Hungary | 336229.7 (246170.2-471515.9) | 3228.4 (2336.2-4581.3) | 293013.7 (215321.1-416412.4) | 3234.2 (2307.4-4590) | 0.36 (-3.86-4.77) |
| Chlamydial infection | Iceland | 948.5 (679.1-1298.5) | 357 (258.7-482.4) | 1257.5 (921.6-1680.8) | 358.1 (257.7-480.2) | 0.38 (-3.03-3.91) |
| Chlamydial infection | India | 13015499.5 (9338258.9-18759276.4) | 1632.2 (1189.7-2349.5) | 24934355.3 (18111633.1-35972883) | 1608.4 (1172.9-2310.2) | -0.44 (-4.05-3.31) |
| Chlamydial infection | Indonesia | 8002039 (5731977.9-11530113.8) | 4491.1 (3290.7-6418.8) | 13893723.7 (10272170.4-19996201.1) | 4398.1 (3230.5-6332) | 0.56 (-3.67-4.97) |
| Chlamydial infection | Iran | 1657093.3 (1167767.3-2312401.4) | 3512 (2559.1-4954.1) | 3395317.3 (2477597.2-4874903.9) | 3331.3 (2422.7-4701.6) | 0.47 (-3.83-4.96) |
| Chlamydial infection | Iraq | 633139.4 (476452.6-823376.6) | 3820.5 (2925.2-5011.7) | 1418518.7 (1003470.4-1978495.4) | 3251.4 (2327.9-4541.3) | -0.11 (-4.46-4.44) |
| Chlamydial infection | Ireland | 14737.2 (10653.1-19929) | 418 (302-568.2) | 20291.2 (15032.8-27557.8) | 418 (299.5-564) | -0.07 (-3.39-3.35) |
| Chlamydial infection | Israel | 25694 (18706.6-35096.5) | 536.1 (389.4-724) | 43594.7 (31741.8-58023.9) | 478.4 (346.8-642.7) | 0.11 (-3.29-3.62) |
| Chlamydial infection | Italy | 262202.3 (193813.8-359239.4) | 434.6 (318.8-604.2) | 243769.1 (182186.8-332952.4) | 431 (318.6-599.8) | -0.26 (-3.6-3.19) |
| Chlamydial infection | Jamaica | 104348.1 (73005.4-145380.9) | 4699.9 (3368.6-6624) | 147474.2 (103977.1-206060.6) | 4678.8 (3330-6568.4) | -0.1 (-3.89-3.85) |
| Chlamydial infection | Japan | 1374429 (1025547.4-1927858.5) | 1031.9 (750.5-1472) | 1164425.1 (861730.2-1629855.7) | 1042.7 (756.2-1484) | 0.26 (-3.62-4.31) |
| Chlamydial infection | Jordan | 43428.6 (30251.3-60835.3) | 1315.9 (939.3-1884.6) | 174103.5 (125052.6-243459.6) | 1293 (939.6-1826.5) | -0.02 (-4.11-4.25) |
| Chlamydial infection | Kazakhstan | 876573.1 (627168.2-1241070.9) | 5165.7 (3757.2-7178.2) | 1018925.6 (745914-1432852.2) | 5167.1 (3763.4-7157.2) | 0.39 (-3.64-4.58) |
| Chlamydial infection | Kenya | 548903.3 (381822.2-770964.7) | 3043.7 (2205.2-4335.2) | 1480072.9 (1044728.9-2094202.4) | 3053.8 (2209.3-4336.6) | -1.43 (-5.05-2.32) |
| Chlamydial infection | Kiribati | 2362 (1680.9-3259.7) | 3255.1 (2361.5-4477.4) | 4009 (2922.1-5420.9) | 3242 (2396.1-4344.7) | -0.14 (-4.48-4.4) |
| Chlamydial infection | Kuwait | 64471.3 (44868-95375.7) | 2987.2 (2157.3-4224.5) | 178691.2 (128416.4-257228.3) | 2719 (1964.5-3810.2) | -0.27 (-4.48-4.13) |
| Chlamydial infection | Kyrgyzstan | 216733.8 (153022.1-308340.9) | 5182.2 (3743.3-7299) | 366154.5 (260949.8-522664.3) | 5178 (3727.8-7283.9) | 0.86 (-3.29-5.19) |
| Chlamydial infection | Laos | 166869.1 (119303-236379.3) | 4760.7 (3460.7-6743.2) | 374956.5 (266971.9-539276.3) | 4676.9 (3386.7-6673) | 0.67 (-3.38-4.9) |
| Chlamydial infection | Latvia | 101269.2 (74680.4-142511.1) | 3726.5 (2732.6-5264.3) | 64732.4 (47655-91862.9) | 3722.3 (2694.8-5324.6) | 0.27 (-3.61-4.31) |
| Chlamydial infection | Lebanon | 60578.3 (42937.3-85100.4) | 2152 (1526.2-3064.2) | 135104.4 (94805.9-193184) | 2105.1 (1505.1-2959.6) | 0.32 (-3.65-4.46) |
| Chlamydial infection | Lesotho | 44100.2 (31472.2-60393.6) | 3456.7 (2495.4-4780.1) | 66320.5 (47024.5-90712.3) | 3367.1 (2435.8-4624.8) | -0.33 (-3.99-3.46) |
| Chlamydial infection | Liberia | 39650.9 (27602.5-55276.5) | 1877.1 (1352.9-2598.9) | 97692.8 (69696-134277.4) | 1833.5 (1324.5-2519.2) | -0.48 (-4.09-3.26) |
| Chlamydial infection | Libya | 107221.7 (75584-149160) | 2891.1 (2065.4-4031.5) | 232229.2 (169007.7-323101.8) | 2779.7 (2021-3845) | 0.15 (-3.89-4.36) |
| Chlamydial infection | Lithuania | 141072.4 (101438.5-200939.6) | 3694.7 (2667.7-5231.5) | 94484.8 (68755.5-135054.9) | 3715.5 (2645-5343.2) | 0.45 (-3.61-4.68) |
| Chlamydial infection | Luxembourg | 1137.1 (819.4-1548.8) | 270 (192.1-366.3) | 1889.4 (1407.5-2582.9) | 273.4 (198.6-371.6) | -0.24 (-3.47-3.11) |
| Chlamydial infection | Macedonia | 69072 (49368.5-98386.2) | 3280.9 (2350.2-4667.7) | 77311 (55812-109645) | 3277.2 (2346.1-4664.4) | 0.06 (-4.35-4.68) |
| Chlamydial infection | Madagascar | 323548.1 (228589.3-448128.7) | 3198 (2301-4453.1) | 855034.6 (605979.9-1200219) | 3170.3 (2275.5-4493.3) | 0.59 (-3.5-4.85) |
| Chlamydial infection | Malawi | 289304.4 (209822.9-396523.3) | 3417.8 (2527.8-4692.7) | 601002.5 (417875.3-817675) | 3340.4 (2382.5-4639.5) | -0.93 (-4.53-2.81) |
| Chlamydial infection | Malaysia | 802588.4 (569441.2-1142147.4) | 4557.2 (3312.1-6423) | 1664400.8 (1193778.7-2402960.5) | 4536.9 (3289.1-6477.8) | -0.14 (-4.07-3.94) |
| Chlamydial infection | Maldives | 8077.7 (5678.4-11363) | 4613.4 (3277.9-6470.9) | 37758.4 (26450.8-56502.8) | 4946.5 (3562.9-7128.7) | 0.08 (-4.31-4.67) |
| Chlamydial infection | Mali | 142106 (99978.6-196358.8) | 2030.6 (1471.8-2820.9) | 391452.6 (272226.6-540943.9) | 1977.3 (1421.4-2712.6) | -0.76 (-4.46-3.08) |
| Chlamydial infection | Malta | 1412.1 (1040.5-1927.5) | 361.4 (261.3-490.2) | 1552.3 (1145-2107.1) | 366.4 (264.5-491.4) | 0.67 (-2.67-4.13) |
| Chlamydial infection | Marshall Islands | 330.1 (231.9-457.5) | 865.7 (615.6-1188.9) | 515.7 (370-716.4) | 858 (617.6-1189.1) | 0.87 (-3.11-5.02) |
| Chlamydial infection | Mauritania | 33323 (23258.4-45619.5) | 1896.4 (1350.9-2628.2) | 72338.8 (50954.2-101126.3) | 1837.4 (1341.8-2540.6) | -0.14 (-4.38-4.29) |
| Chlamydial infection | Mauritius | 55723.4 (38889.7-79998) | 4546.3 (3263.6-6385.2) | 59942.4 (44568.5-84402.5) | 4465.1 (3272.7-6363.1) | 0.69 (-3.22-4.77) |
| Chlamydial infection | Mexico | 3471359.6 (2471419.3-4853904.1) | 4466.4 (3276.7-6285.8) | 6100382.2 (4491310.5-8586331.5) | 4402.8 (3234.1-6213.5) | 0.09 (-3.94-4.29) |
| Chlamydial infection | Moldova | 174831.8 (122912.7-251732.8) | 3772.2 (2703.4-5358.1) | 149728.9 (108277.4-213293.1) | 3768.8 (2677.4-5382.7) | 0.22 (-3.64-4.23) |
| Chlamydial infection | Monaco | 107.7 (79.9-144.4) | 356.9 (262.1-474.9) | 117.4 (87.4-155.8) | 358.2 (259-483.9) | -0.03 (-3.59-3.66) |
| Chlamydial infection | Mongolia | 102415.6 (71749.1-144674.2) | 5245.3 (3775.7-7355.9) | 184834.2 (133529.4-261516.1) | 5224.3 (3804.1-7248.2) | 0.37 (-4.08-5.03) |
| Chlamydial infection | Montenegro | 21512 (15230.1-30328.5) | 3269.1 (2323.6-4584.6) | 19644.6 (14042.1-27660.7) | 3227.7 (2276.1-4564.2) | 0 (-4.31-4.51) |
| Chlamydial infection | Morocco | 1272836.1 (919527.7-1720089) | 5261.9 (3898.3-7094.7) | 1722070.3 (1251033.2-2436643.2) | 4364.9 (3162.5-6167.4) | -0.27 (-4.32-3.94) |
| Chlamydial infection | Mozambique | 366714.7 (264298.3-509536.6) | 3333.7 (2432.4-4660) | 895549.9 (632807.6-1222057) | 3339.3 (2403.1-4588) | -0.21 (-3.9-3.63) |
| Chlamydial infection | Myanmar | 1833757.1 (1267228.1-2619870.5) | 4680.8 (3350.6-6667.6) | 2687058.7 (1955504.8-3729544) | 4584.2 (3326.3-6405.1) | -0.3 (-4.05-3.6) |
| Chlamydial infection | Namibia | 42676.4 (29672.4-59848.8) | 3388.9 (2416-4722.6) | 85478.2 (59918.6-117656.6) | 3337.8 (2383.3-4565.8) | -0.75 (-4.39-3.03) |
| Chlamydial infection | Nauru | 285.9 (202.5-394.1) | 2931.2 (2110.1-4020.2) | 331.4 (235.5-453.1) | 2954.2 (2117.6-3980.1) | 1.02 (-3.14-5.35) |
| Chlamydial infection | Nepal | 323466.1 (226777.9-452639.3) | 1900.2 (1356.6-2672.8) | 608016.4 (427089.4-849551) | 1869.8 (1335.2-2638.5) | 0.14 (-3.56-3.98) |
| Chlamydial infection | Netherlands | 80235.5 (58918.1-108280) | 485.5 (356.5-651.6) | 67897.4 (50123.9-90541) | 415.6 (299.3-559.7) | -0.06 (-3.48-3.47) |
| Chlamydial infection | New Zealand | 61687.5 (44912.2-86025.2) | 1698.7 (1242.9-2359.9) | 89000.4 (65999.7-123071.9) | 1696.7 (1244.2-2338.8) | 0.13 (-3.66-4.07) |
| Chlamydial infection | Nicaragua | 130928.6 (96114.2-178190.3) | 4002.7 (2984.5-5507.2) | 277714.7 (200224.1-391100.3) | 3855.6 (2815.5-5422) | 0.45 (-3.69-4.78) |
| Chlamydial infection | Niger | 121777.4 (84581.6-169445.8) | 1914.7 (1370.7-2678.2) | 355243.3 (247586.4-494292.5) | 1847.9 (1339.2-2549.7) | -1.21 (-4.97-2.7) |
| Chlamydial infection | Nigeria | 1857237.1 (1310102.4-2594053.6) | 2425.5 (1759.9-3441.8) | 5000540.2 (3557417.5-6988791.9) | 2545.3 (1842.4-3600.6) | -0.41 (-4.07-3.4) |
| Chlamydial infection | Niue | 58.5 (41.8-78.1) | 2898.3 (2086-3893.8) | 46.2 (34.2-62.1) | 2914 (2129.5-3977.3) | 0.96 (-3.19-5.28) |
| Chlamydial infection | North Korea | 1043842.1 (745291.8-1458578.3) | 4839.4 (3477.5-6830.1) | 1410244.3 (1047097.9-2004546.8) | 4778.9 (3486.1-6825.6) | 0.61 (-3.52-4.92) |
| Chlamydial infection | Northern Mariana Islands | 1651.9 (1145.4-2273.9) | 2764.4 (1980.7-3737.3) | 1332 (977.1-1809.2) | 2738.2 (1973.2-3762.8) | 0.11 (-4.12-4.52) |
| Chlamydial infection | Norway | 31252.6 (23378.6-43502.7) | 703.6 (522.8-982.5) | 38673.9 (28922.3-53604.6) | 704.2 (520-982) | -0.12 (-3.57-3.45) |
| Chlamydial infection | Oman | 59354.2 (41331.1-85971.5) | 2904.4 (2088.8-4105.3) | 185304.9 (127312.4-273715.1) | 2838 (2032.2-4013) | 0.21 (-3.9-4.5) |
| Chlamydial infection | Pakistan | 2328533.7 (1651010-3266483.1) | 2578.8 (1868.7-3636.4) | 5851642.1 (4146347.3-8283898.3) | 2496.4 (1803.4-3534.8) | 1.1 (-2.86-5.23) |
| Chlamydial infection | Palau | 481.6 (340.4-655.9) | 2857.4 (2047.6-3844.1) | 473.8 (354.1-634) | 2487.3 (1806.5-3376.7) | 0.87 (-3.28-5.19) |
| Chlamydial infection | Palestine | 47358.8 (33039.5-66082.2) | 2840.5 (2029.6-4020) | 145178.5 (102364.5-201737.7) | 2787.6 (2011.8-3888.7) | 0.16 (-4.12-4.63) |
| Chlamydial infection | Panama | 89440.3 (63531.3-122856.6) | 3743.6 (2730-5210.6) | 162560.3 (116149.3-223053.2) | 3714.6 (2641.5-5133.3) | 0.21 (-3.6-4.17) |
| Chlamydial infection | Papua New Guinea | 167223.9 (123083.7-220928) | 4242.4 (3154.3-5590.7) | 395767.8 (285895.7-543361.7) | 3739.2 (2749.9-5123.4) | 0.53 (-3.47-4.7) |
| Chlamydial infection | Paraguay | 166056.7 (116677.5-231189.5) | 4518 (3231.3-6285) | 349086.6 (248213.4-486376.2) | 4488.2 (3234.1-6215.5) | 0.46 (-3.59-4.68) |
| Chlamydial infection | Peru | 643685 (461771.1-897289.5) | 3148.8 (2301.1-4406.9) | 1203217.3 (860404-1708664.3) | 3054.3 (2199.3-4324.8) | 0.01 (-3.81-3.99) |
| Chlamydial infection | Philippines | 2798757.2 (2004859-4057785) | 4876.6 (3549.8-6990.9) | 5641324.3 (4085612-8116903.7) | 4773.8 (3481-6842.5) | 1.16 (-2.86-5.36) |
| Chlamydial infection | Poland | 1316865.5 (949812.1-1895336.8) | 3256.5 (2336.4-4676.4) | 1277397.4 (936617.5-1843039.3) | 3223.2 (2335.9-4660.2) | 0.18 (-4.01-4.55) |
| Chlamydial infection | Portugal | 48101 (35138.5-64810.8) | 465.8 (339.1-635.6) | 46627 (34673.7-62343.3) | 465.9 (335.1-628.4) | -0.73 (-3.96-2.61) |
| Chlamydial infection | Puerto Rico | 167606.6 (122495.2-234984.3) | 4589.2 (3360.8-6429.4) | 140788.9 (103952.7-194532.1) | 4602 (3314.6-6444.9) | -0.9 (-4.73-3.08) |
| Chlamydial infection | Qatar | 19132.9 (13115.8-28050.7) | 3019.9 (2181.6-4249) | 147324.6 (100627-220625.8) | 2976.7 (2124.1-4192.6) | -0.23 (-4.5-4.22) |
| Chlamydial infection | Romania | 758419.2 (548682.9-1072588.3) | 3293.6 (2350.4-4677.3) | 570686.3 (421241.4-799033.1) | 3275.2 (2359.8-4645) | 0.28 (-3.82-4.56) |
| Chlamydial infection | Russian Federation | 5886622.1 (4286328-8545151.2) | 3592.4 (2631.5-5173) | 5443055.8 (4001963-7794173.3) | 3581.5 (2616.7-5145.9) | 1.19 (-2.71-5.26) |
| Chlamydial infection | Rwanda | 185584.9 (130887.2-260420.2) | 3053 (2185.2-4257.4) | 398399.8 (278664.8-555249.9) | 3007.5 (2145.5-4217.2) | -0.97 (-4.64-2.84) |
| Chlamydial infection | Saint Kitts and Nevis | 1818.4 (1245.4-2572.5) | 4684.7 (3364.4-6612.1) | 3106 (2262.4-4324.8) | 4673.9 (3366.3-6500.1) | -0.03 (-3.79-3.88) |
| Chlamydial infection | Saint Lucia | 5915.1 (4141.6-8261.7) | 4712.1 (3422.7-6615.9) | 9106.4 (6707.4-12813.5) | 4678.7 (3397.5-6631.5) | -0.41 (-4.16-3.49) |
| Chlamydial infection | Saint Vincent and the Grenadines | 4739.9 (3355.7-6607.4) | 4711.4 (3418.1-6544.1) | 5403.8 (3996.3-7469.7) | 4671.6 (3404.1-6552) | -0.51 (-4.33-3.47) |
| Chlamydial infection | Samoa | 6297.6 (4488.6-8536.2) | 4283.5 (3164.8-5797.8) | 8476 (6178.9-11331.4) | 4297.9 (3151.6-5728.1) | 0.97 (-3.28-5.41) |
| Chlamydial infection | San Marino | 92.2 (67.7-122.5) | 358.9 (264.2-477.5) | 109.7 (81-146.9) | 355.6 (255.9-485) | -0.07 (-3.62-3.61) |
| Chlamydial infection | Sao Tome and Principe | 1758.3 (1229.1-2439.2) | 1853.1 (1306.1-2564.9) | 3967.1 (2781.1-5527.1) | 1819.3 (1294.8-2530.4) | -0.49 (-4.64-3.84) |
| Chlamydial infection | Saudi Arabia | 721682.7 (527649.8-977308.6) | 4561.6 (3383.4-6165.1) | 2079461.6 (1500235.1-2904535.2) | 3977.5 (2897.7-5400) | -0.03 (-4.22-4.34) |
| Chlamydial infection | Senegal | 85675.3 (59689.2-116567.1) | 1338.7 (971-1805.1) | 159981.6 (112094.6-222857.8) | 1073.7 (771.8-1485.2) | -0.7 (-4.3-3.04) |
| Chlamydial infection | Serbia | 324912.1 (234265.8-464369.5) | 3278.3 (2341.9-4709.5) | 290385.6 (209231.4-401808.2) | 3271.4 (2326.6-4583.1) | 0 (-4.35-4.55) |
| Chlamydial infection | Seychelles | 3202.1 (2247.4-4562.7) | 4432 (3181.9-6220.9) | 5085 (3713.6-7275.5) | 4448.3 (3224-6372.2) | -0.26 (-4.28-3.94) |
| Chlamydial infection | Sierra Leone | 70342.4 (49169-96887.5) | 1884.7 (1362.7-2592.1) | 158251.2 (112579.1-216383) | 1840.7 (1337.4-2509.4) | -0.21 (-3.84-3.55) |
| Chlamydial infection | Singapore | 41340.4 (29131.3-58492.6) | 1069.9 (774.5-1488.6) | 70182.8 (51173.1-99448.3) | 1071.8 (770.2-1493.3) | -0.46 (-3.96-3.18) |
| Chlamydial infection | Slovakia | 177847.4 (125934.8-252102.8) | 3244.1 (2297.4-4573.1) | 178102.9 (128572.7-254091.5) | 3235.1 (2291.7-4578.8) | 0.05 (-4.36-4.66) |
| Chlamydial infection | Slovenia | 53718.5 (38636.3-76807.2) | 2535.6 (1824.7-3613.8) | 47653.1 (34739.9-68198.3) | 2542.2 (1804.4-3663.3) | -0.2 (-4.32-4.1) |
| Chlamydial infection | Solomon Islands | 7091.9 (4922.1-9747.5) | 2467.8 (1762.6-3383.3) | 16351.2 (11509.2-22900.1) | 2444.8 (1735.6-3462.7) | 0.94 (-3.15-5.19) |
| Chlamydial infection | Somalia | 299706.2 (214278.6-414890.7) | 4505 (3249.5-6187.3) | 747862.6 (529175-1042240.6) | 4063.8 (2913.5-5753.5) | -0.55 (-4.47-3.53) |
| Chlamydial infection | South Africa | 2329544 (1674444.1-3234600) | 6451.6 (4721.4-8942) | 4105626 (3001424-5819166.9) | 6354.4 (4672.6-8899.6) | -0.52 (-4.33-3.44) |
| Chlamydial infection | South Korea | 530390.5 (380411.6-729646.1) | 1027.4 (752.8-1390.3) | 584662.1 (434903.2-811220.8) | 1087.6 (787-1535) | 0.02 (-3.88-4.08) |
| Chlamydial infection | South Sudan | 165978.2 (117133.8-228614.9) | 3261.3 (2352.5-4477.7) | 256798.7 (183968.8-348802) | 3168.1 (2262.9-4381.4) | -0.09 (-3.83-3.8) |
| Chlamydial infection | Spain | 191452.1 (139348.2-258718.4) | 481.5 (349.5-651.6) | 217696.9 (159609.5-296205.7) | 487.8 (352.8-670.3) | -0.6 (-3.89-2.82) |
| Chlamydial infection | Sri Lanka | 822865.7 (577946.2-1165152.1) | 4549.5 (3224.9-6464.8) | 987839.5 (727211.3-1402617.8) | 4398 (3193.7-6279.8) | 0.01 (-4.41-4.63) |
| Chlamydial infection | Sudan | 646831.4 (459452.1-885720.3) | 3726.5 (2687-5111.7) | 1632710.3 (1184930.6-2183444.8) | 3741.4 (2780.5-5046.3) | 0.13 (-3.67-4.08) |
| Chlamydial infection | Suriname | 18274.2 (13000.6-25269) | 4797.3 (3478.2-6689.4) | 27982.7 (20224.2-39135.2) | 4770.1 (3412.5-6733.7) | -0.09 (-3.89-3.85) |
| Chlamydial infection | Swaziland | 22878.7 (16189.9-31219) | 3398.5 (2450-4661.1) | 40558.1 (28596.9-56836) | 3337.3 (2424.5-4647.8) | -0.24 (-3.91-3.57) |
| Chlamydial infection | Sweden | 74175.5 (54918.5-103029.1) | 844.1 (614.7-1176.3) | 82445.3 (61212.2-113338.1) | 827.2 (607.8-1135.2) | -0.15 (-3.64-3.47) |
| Chlamydial infection | Switzerland | 26319.2 (19321.6-35818.5) | 346.6 (251.9-467.7) | 30803.9 (22855.3-41931.3) | 346.1 (251-470.8) | -0.79 (-4.08-2.62) |
| Chlamydial infection | Syria | 303451.6 (215200.2-420736.1) | 2889.4 (2096.9-4055.1) | 359877.6 (266694.3-483876.4) | 2779.2 (1983.4-3882.2) | 0 (-4.28-4.47) |
| Chlamydial infection | Taiwan | 1291480.6 (940229-1807428.1) | 5595.7 (4154.9-7723.8) | 1316331.1 (975106.1-1826255.2) | 5260.2 (3780.6-7299.8) | 0.43 (-3.63-4.67) |
| Chlamydial infection | Tajikistan | 246063.3 (169937.4-347121.6) | 5284.1 (3801.2-7375.4) | 545664.8 (385155.1-769197.6) | 5218.1 (3779.9-7256.2) | 0.14 (-4.11-4.59) |
| Chlamydial infection | Tanzania | 823795.5 (592778.5-1093998.7) | 3808.8 (2828.8-5181.6) | 1942238.9 (1377785.8-2689236.1) | 3615.8 (2625.1-5009.4) | -1.24 (-4.97-2.64) |
| Chlamydial infection | Thailand | 3264318.1 (2395550.8-4420192.2) | 5211.5 (3869.9-7057.9) | 3358199.7 (2476265-4651107.5) | 4921.2 (3523.5-6844.5) | -0.72 (-4.45-3.15) |
| Chlamydial infection | The Bahamas | 13194.2 (9167.5-18604.1) | 4704.5 (3384.4-6551.1) | 19917.7 (14247.5-27705.4) | 4673.1 (3323-6539.4) | -0.3 (-4.02-3.56) |
| Chlamydial infection | The Gambia | 11230.8 (7660.9-15915.8) | 1309.2 (931.9-1835.6) | 28377.8 (19734.9-39531.5) | 1248.3 (900.6-1738.7) | 0.14 (-3.42-3.83) |
| Chlamydial infection | Timor-Leste | 35780.1 (25048.5-51692.1) | 4813.7 (3464.3-6854.2) | 57859.2 (41449.3-80324.9) | 4584.5 (3333.1-6429.7) | -0.2 (-4.01-3.76) |
| Chlamydial infection | Togo | 57340.2 (40253.5-78574.8) | 1874.2 (1350.1-2564.8) | 148777.7 (105121.3-204065.3) | 1843.4 (1324.5-2524.1) | -0.96 (-4.47-2.68) |
| Chlamydial infection | Tokelau | 41.1 (29.1-56) | 3031 (2187.8-4094.5) | 38 (27.5-51.9) | 2902.9 (2080.9-3997) | 0.84 (-3.31-5.17) |
| Chlamydial infection | Tonga | 2921.5 (2098.3-3943) | 3522.8 (2550.9-4735.9) | 3388.2 (2433.7-4538) | 3537.3 (2556-4780.5) | 1.02 (-3.16-5.37) |
| Chlamydial infection | Trinidad and Tobago | 58116.4 (41103.6-81807) | 4711.1 (3430.5-6556.6) | 68142.8 (48871.9-95721.1) | 4667.7 (3335.7-6525) | -0.02 (-3.72-3.83) |
| Chlamydial infection | Tunisia | 229221.3 (162049.4-319712.3) | 2866.2 (2055.6-4022.5) | 346451.3 (250243.2-488990.6) | 2767.4 (1980.3-3890.7) | 0.28 (-3.98-4.72) |
| Chlamydial infection | Turkey | 1886558.9 (1378773.3-2521512.9) | 3288.3 (2436.3-4420.7) | 2558163.4 (1850889.9-3606847.5) | 2844.7 (2031.3-4006.5) | -0.04 (-4.32-4.44) |
| Chlamydial infection | Turkmenistan | 178708.1 (124880.1-251300) | 5211.8 (3782.4-7312.7) | 278300.2 (201463.9-390176.2) | 5106.4 (3724.7-7089.1) | -0.39 (-4.59-4) |
| Chlamydial infection | Tuvalu | 287.2 (206.4-383.8) | 3176.6 (2303.3-4240.2) | 347.4 (249.8-475.3) | 2780.6 (2021.1-3795.1) | 0.84 (-3.32-5.17) |
| Chlamydial infection | Uganda | 461202.2 (328017.3-632334.9) | 3230.4 (2346-4443) | 1107962 (791855.5-1524960.2) | 2943.1 (2140.5-4023.7) | -0.66 (-4.3-3.11) |
| Chlamydial infection | Ukraine | 1938790.7 (1406265.2-2758978.9) | 3590.1 (2598.9-5098.8) | 1659948.9 (1211576.2-2373576.3) | 3595.2 (2622.6-5124.9) | 0.33 (-3.48-4.29) |
| Chlamydial infection | United Arab Emirates | 70882 (48508.9-105741.6) | 2770.3 (1981.7-3876.4) | 404814.9 (281020.7-585400.5) | 2730.3 (1963.1-3869.3) | 0.15 (-4.02-4.5) |
| Chlamydial infection | United Kingdom | 179604.1 (131890-248433.9) | 301.8 (219.6-420) | 204721.7 (152290.7-280706.9) | 304.2 (221.5-423.4) | 0 (-3.29-3.4) |
| Chlamydial infection | United States | 1969259.7 (1414836.8-2779448.7) | 709.7 (515-987.5) | 2495068.4 (1858900.6-3435753.4) | 766 (558.2-1074.1) | -0.15 (-3.67-3.5) |
| Chlamydial infection | Uruguay | 26842.6 (19715.3-37229.7) | 886.8 (648.7-1238) | 30303.3 (22525.6-41795.8) | 882 (648.3-1228.2) | 0.16 (-3.27-3.71) |
| Chlamydial infection | Uzbekistan | 990203.1 (694485.6-1367093) | 5176.6 (3734.5-7167.4) | 1920508.8 (1392435.5-2679567.5) | 5150.7 (3761.9-7097.1) | 0.53 (-3.58-4.81) |
| Chlamydial infection | Vanuatu | 5810.1 (4250-7594.2) | 4170.7 (3115-5408.7) | 11293.8 (8055.5-15154.8) | 3685.1 (2660.2-4962.1) | 0.92 (-3.27-5.3) |
| Chlamydial infection | Venezuela | 702289.4 (498957.4-982224.3) | 3763.6 (2743.1-5245.3) | 1010757.6 (741535.8-1403871.9) | 3776.5 (2750.6-5211) | 0.25 (-3.64-4.3) |
| Chlamydial infection | Vietnam | 2560726 (1834517.9-3626855.6) | 4042.2 (2977.5-5648.6) | 4647293 (3372188.4-6715461.3) | 4190.2 (3023.8-5959.6) | 0.32 (-3.54-4.34) |
| Chlamydial infection | Yemen | 307577.2 (214927.5-431623.7) | 2974.3 (2122.6-4148.9) | 924110.9 (652073.4-1296916.3) | 2880 (2083.4-4049.3) | 0.51 (-3.57-4.77) |
| Chlamydial infection | Zambia | 156109.8 (108699.4-216885.8) | 2390.8 (1694.2-3350.5) | 444391.8 (309438.1-626114.5) | 2406.5 (1711.6-3384.8) | -0.88 (-4.51-2.9) |
| Chlamydial infection | Zimbabwe | 192884.9 (134813.5-268262.5) | 2206.4 (1557.9-3089) | 328684.6 (232070-451127.3) | 2198.5 (1563.5-3013.8) | -1.3 (-4.89-2.44) |
| Genital herpes | Afghanistan | 31932 (26399.6-38298.2) | 383.7 (317.4-451.2) | 109599.1 (90599.3-129207.3) | 363.3 (304.7-425.7) | 0.27 (-3.89-4.6) |
| Genital herpes | Albania | 8716 (7216.1-10333.2) | 255.4 (212.3-301) | 6862.3 (5700-8070.6) | 254.8 (211.4-301.6) | -0.11 (-4.53-4.51) |
| Genital herpes | Algeria | 95758.9 (78430.6-114384.6) | 390.1 (324.1-454.2) | 176185.5 (146856.9-206790.4) | 390.6 (327.3-456.9) | 0.49 (-3.58-4.74) |
| Genital herpes | American Samoa | 331.4 (274.8-389) | 647.6 (541.4-752.4) | 310.9 (262.4-360.7) | 651.4 (549.7-757) | 0.96 (-3.19-5.29) |
| Genital herpes | Andorra | 211.9 (172.7-252.3) | 330.7 (275.2-388.7) | 276.1 (226.8-327.2) | 333.3 (280-390.8) | -0.13 (-3.69-3.57) |
| Genital herpes | Angola | 116199.2 (99159.2-131547.2) | 1093.5 (952.4-1222.2) | 376597.8 (323165.8-428212.3) | 1118.5 (972.6-1254.3) | 0.43 (-3.19-4.19) |
| Genital herpes | Antigua and Barbuda | 532.1 (445.7-624.1) | 808.4 (683.9-933.7) | 752.8 (637.3-863) | 805.9 (680.9-925.1) | -0.21 (-4.12-3.85) |
| Genital herpes | Argentina | 201452.2 (187732.5-214511.9) | 618.6 (575.8-660.2) | 280264.8 (236504.2-320690.9) | 599.7 (505.8-687.1) | -0.06 (-3.49-3.5) |
| Genital herpes | Armenia | 12263.8 (9982.5-14481.8) | 339.8 (279.3-397.3) | 10520.3 (8699.8-12431.7) | 339.1 (281.6-397.3) | 0.92 (-3.39-5.42) |
| Genital herpes | Australia | 89125.7 (73881.8-108414) | 510 (421.8-620) | 94280.9 (78544.3-110528.1) | 399.9 (335.1-470.6) | -0.13 (-3.7-3.57) |
| Genital herpes | Austria | 24537.4 (20379.4-28629.2) | 301.4 (251-352.3) | 25551.4 (21200.8-30280.5) | 302.1 (250.5-353.7) | -0.37 (-3.76-3.13) |
| Genital herpes | Azerbaijan | 25895.9 (21073.2-30648.1) | 340.4 (282.2-403.1) | 39956.2 (32874.4-47233.5) | 337.5 (278.8-396.6) | 0 (-4.41-4.62) |
| Genital herpes | Bahrain | 2199.7 (1780-2648.8) | 370.9 (310.5-439.7) | 6730.8 (5593.6-7989.5) | 360.7 (302.4-423.5) | -0.25 (-4.4-4.07) |
| Genital herpes | Bangladesh | 269642.9 (222443-318011.3) | 279.2 (232.1-329.6) | 493262 (410913-578331.6) | 281.3 (235.3-329.5) | 0.02 (-4.03-4.25) |
| Genital herpes | Barbados | 2232.2 (1868.2-2594.8) | 802 (677.1-923.5) | 2210.2 (1875.9-2519.4) | 807.9 (679.2-931.2) | -0.19 (-4.02-3.8) |
| Genital herpes | Belarus | 49225.4 (40893.8-57778.7) | 470.3 (391.7-547.6) | 38798.4 (32350.2-45580.1) | 467.6 (392.6-545.4) | 0.97 (-3.01-5.1) |
| Genital herpes | Belgium | 33785.7 (28296.2-39474.7) | 334 (280.1-390.7) | 34721.6 (28910.6-40597.6) | 335.6 (277.9-394.2) | -0.14 (-3.44-3.28) |
| Genital herpes | Belize | 1474.1 (1223.7-1731.3) | 801.2 (675.9-926) | 3926.9 (3314.8-4513.8) | 809.8 (687.5-924) | -0.21 (-3.99-3.72) |
| Genital herpes | Benin | 33215.5 (27546.2-39260) | 755.1 (632.9-872.7) | 100800.7 (83360.2-118249.6) | 746.4 (629.1-859.8) | -0.5 (-4.08-3.21) |
| Genital herpes | Bermuda | 545.3 (454.1-640.5) | 802.7 (678.3-929.9) | 411.3 (350.4-471.4) | 809 (683.4-932) | -0.48 (-4.39-3.6) |
| Genital herpes | Bhutan | 1630.2 (1334.8-1922.8) | 275.4 (228-323.1) | 2397.8 (1967-2827) | 276.5 (230.7-325.1) | 0.16 (-3.49-3.96) |
| Genital herpes | Bolivia | 55865.1 (46802.3-64938.9) | 868.5 (737.7-995) | 110480.6 (93181-126464.5) | 859.8 (732.1-977.2) | -0.33 (-4.02-3.51) |
| Genital herpes | Bosnia and Herzegovina | 12597.6 (10383.4-14821.7) | 255.2 (212.2-300.8) | 8151.1 (6734.9-9695.3) | 255.7 (212-302.7) | 0.06 (-4.35-4.67) |
| Genital herpes | Botswana | 14587.1 (12148.7-16865.3) | 1068.8 (905-1216) | 30972.5 (26502.2-35105) | 1125.2 (964.7-1275.9) | -1.25 (-4.85-2.49) |
| Genital herpes | Brazil | 1530671.1 (1304829.1-1767026.6) | 949.4 (812.3-1083.4) | 2165397.8 (1841840.2-2476196.7) | 938.2 (799-1073.9) | 0.06 (-3.86-4.14) |
| Genital herpes | Brunei | 1011 (820.6-1195) | 345.2 (285.7-404.7) | 1863.4 (1549.2-2191.2) | 345.8 (288.7-404.3) | 0.32 (-3.39-4.18) |
| Genital herpes | Bulgaria | 28815.6 (24022.8-33718.7) | 337.3 (282.5-394.9) | 20525.9 (16996-24194.6) | 335.6 (278.1-393.8) | 0.4 (-3.8-4.78) |
| Genital herpes | Burkina Faso | 65403.7 (53672.4-76783.8) | 782.4 (652.5-908.6) | 171313.1 (142410.1-199859.5) | 772.7 (646.5-893) | -1.47 (-5.19-2.41) |
| Genital herpes | Burundi | 55080.8 (45926.6-63885.8) | 995.8 (844.3-1144.4) | 138918.7 (115380.2-160796.6) | 993 (843.2-1131.3) | -1.89 (-5.64-2.01) |
| Genital herpes | Cambodia | 55110.9 (45446.5-64800) | 567.2 (475.6-657.5) | 104311.2 (87644.9-123375.5) | 562.1 (473.8-660.1) | -0.55 (-4.4-3.45) |
| Genital herpes | Cameroon | 94320.6 (78785.6-109850.3) | 935.8 (796.3-1070.9) | 316220.9 (268487.7-362780.5) | 935.5 (803-1062.9) | -0.82 (-4.42-2.91) |
| Genital herpes | Canada | 99040.9 (81513.1-116478.5) | 341.4 (284.4-400.5) | 115772.8 (97278.6-135405.6) | 339.6 (284.5-400.3) | 0.1 (-3.42-3.74) |
| Genital herpes | Cape Verde | 2603.5 (2139.4-3050.5) | 774 (649.8-891.5) | 4845.9 (4045-5580) | 754.1 (628.7-864.5) | -0.72 (-4.4-3.11) |
| Genital herpes | Central African Republic | 31818.2 (27192.9-36016.9) | 1115.1 (968.7-1244.2) | 66750.1 (57357.4-75695.9) | 1120.1 (976.2-1257.8) | -0.86 (-4.39-2.79) |
| Genital herpes | Chad | 36419.2 (29815.8-42733.7) | 680.4 (563.9-788.1) | 109232.2 (89987.6-128866.9) | 681.8 (573.4-787.6) | -0.59 (-4.2-3.15) |
| Genital herpes | Chile | 87700.7 (72957.4-101817.6) | 598.6 (504.3-686.9) | 112860.9 (95155.6-130150.7) | 591.3 (499.9-678.6) | 0.38 (-3.11-4.01) |
| Genital herpes | China | 4937219.5 (4076505.5-5813009.9) | 376.9 (315.2-444) | 5385309.8 (4478206.7-6312319.8) | 375.1 (311.1-436.5) | -0.08 (-4.2-4.23) |
| Genital herpes | Colombia | 351051.3 (320815.3-382334.5) | 958.7 (880.7-1041.4) | 442523.5 (373222-506222.1) | 839.2 (711.3-956.3) | 0.24 (-3.66-4.29) |
| Genital herpes | Comoros | 4604.8 (3833.7-5383.6) | 979.6 (830.3-1122.3) | 8060.8 (6814.5-9268.7) | 983.4 (836.8-1127.4) | -0.17 (-4.66-4.53) |
| Genital herpes | Congo | 28908.3 (24720.3-32873.3) | 1108.8 (972.5-1235.4) | 66210.3 (57406.8-74176) | 1114.9 (972.4-1248.1) | -0.53 (-4.06-3.13) |
| Genital herpes | Cook Islands | 127.7 (106-149) | 648.1 (540.2-752.7) | 106.2 (89.7-122.8) | 651.3 (546.9-760.5) | 1.05 (-3.11-5.39) |
| Genital herpes | Costa Rica | 27344.9 (24742.7-30079.4) | 831.9 (751.6-913.3) | 40299.5 (34284.5-46206.2) | 803.2 (684.4-916.9) | 0.07 (-3.98-4.28) |
| Genital herpes | Cote d'Ivoire | 94217.8 (77358.3-109659.1) | 770.4 (648.2-879.6) | 221095.2 (184841.8-257330.3) | 764.9 (645.7-885.5) | -1.18 (-4.71-2.49) |
| Genital herpes | Croatia | 14544.1 (12031.4-16989.1) | 284.4 (236.2-332) | 11073.6 (9137.6-13018.9) | 283.3 (236.6-331.3) | 0.08 (-4.16-4.5) |
| Genital herpes | Cuba | 100113.1 (83854.2-116205.2) | 799.8 (674.1-918.1) | 80667 (68704.4-91978.9) | 799.3 (677.4-913.3) | 0.92 (-3.19-5.22) |
| Genital herpes | Cyprus | 2701.8 (2241.3-3156.4) | 333.7 (277.5-390) | 4683.9 (3829.9-5564.4) | 335.2 (276.7-392.4) | 0.04 (-3.63-3.85) |
| Genital herpes | Czech Republic | 19798.4 (18513.5-20984.2) | 192.9 (182.1-203.7) | 22718.4 (18730.7-27060.9) | 227.1 (187.7-266.1) | 0.39 (-3.87-4.83) |
| Genital herpes | Democratic Republic of the Congo | 430411.2 (369376.3-488879.1) | 1098 (962-1229) | 1073753.3 (926943.3-1221699.4) | 1092.1 (955.1-1228.6) | -1.3 (-4.94-2.49) |
| Genital herpes | Denmark | 20845.8 (17160.2-24464.3) | 401.1 (331.1-476.3) | 19621.9 (16283.7-22876.4) | 370.2 (310.6-433.7) | -0.26 (-3.73-3.34) |
| Genital herpes | Djibouti | 4132.6 (3443.4-4823.6) | 889.5 (754.4-1023) | 12430.7 (10392.2-14406.8) | 886.1 (744.2-1024.2) | -0.19 (-3.94-3.7) |
| Genital herpes | Dominica | 588.4 (492.1-691.3) | 794.4 (672-918.1) | 550.1 (465.7-632.4) | 800.7 (676.9-922.5) | -0.21 (-4.16-3.9) |
| Genital herpes | Dominican Republic | 62644.4 (51740.2-73908.2) | 810.4 (679.5-935.1) | 95855 (80665.3-110195.7) | 805.9 (680-923.7) | -0.71 (-4.46-3.19) |
| Genital herpes | Ecuador | 92921.6 (77457.8-107952.5) | 867.9 (731.5-992.4) | 165609.1 (140911.5-190870.8) | 860.6 (732-984.2) | 0.54 (-3.22-4.44) |
| Genital herpes | Egypt | 212885.6 (176251.3-252430.5) | 388.8 (322.8-452.6) | 420622.9 (352257.4-494239.8) | 389.8 (326.6-457) | 0 (-4.33-4.53) |
| Genital herpes | El Salvador | 44096.8 (36779.5-51176.9) | 817.5 (694-934.3) | 55801.5 (47300.9-64034.4) | 818.7 (698.5-933.7) | -0.23 (-4.16-3.86) |
| Genital herpes | Equatorial Guinea | 4664.8 (4004-5262.3) | 1125.6 (983.9-1247.7) | 19417 (16661.9-22129.1) | 1044.9 (911.6-1172.8) | 0.57 (-2.97-4.24) |
| Genital herpes | Eritrea | 28377.9 (24570.1-32688.2) | 829.6 (717.1-941.8) | 61677.9 (51253-72274.9) | 846.1 (713.6-982) | -1.3 (-5.1-2.65) |
| Genital herpes | Estonia | 7178 (6043.6-8419.2) | 468.1 (393.3-546.9) | 5248.5 (4408.7-6146.1) | 467.7 (390.7-549.2) | 0.65 (-3.26-4.73) |
| Genital herpes | Ethiopia | 362190.4 (299111.9-424122.8) | 776.8 (650-894.7) | 898526.8 (743440.4-1057605.6) | 772.1 (647.6-893.3) | -1.38 (-5.11-2.5) |
| Genital herpes | Federated States of Micronesia | 654.6 (541.5-763.6) | 649.4 (546.8-751.4) | 721.5 (605.3-846.1) | 653.2 (547.1-759.4) | 0.99 (-3.14-5.3) |
| Genital herpes | Fiji | 5329.1 (4431.2-6233.3) | 647.3 (538.2-750.3) | 6170.9 (5178.4-7135.9) | 652.7 (546.2-754.3) | 0.08 (-4.15-4.5) |
| Genital herpes | Finland | 20640.8 (18452.3-23116.5) | 414.2 (372.9-458.3) | 15966.9 (13340.3-18548.2) | 329.1 (274.1-383.1) | -0.17 (-3.68-3.47) |
| Genital herpes | France | 198629.7 (164431.9-233651.9) | 338.5 (280.1-397.4) | 197764 (164244.4-232932.1) | 338.6 (282.8-398.8) | 0.07 (-3.21-3.47) |
| Genital herpes | Gabon | 11540.2 (9856.3-13180.5) | 1108.4 (963.2-1247.6) | 23096 (20043.8-26106.1) | 1141.1 (1001.7-1274.3) | -0.52 (-4.04-3.14) |
| Genital herpes | Georgia | 19298.1 (15849.1-22597.9) | 340.4 (279.8-397.9) | 11501.1 (9574.8-13458.4) | 337.5 (280.6-394.2) | 0.67 (-3.52-5.04) |
| Genital herpes | Germany | 388437.3 (319010.8-462762) | 482.7 (397-582.7) | 295791.5 (245740.5-345091.1) | 400.1 (334-470.7) | -0.12 (-3.64-3.52) |
| Genital herpes | Ghana | 114176.4 (93708.8-133323) | 770.2 (641.8-887.1) | 285856.6 (236826.1-333737.2) | 773.8 (648.4-894.8) | -0.48 (-4.16-3.34) |
| Genital herpes | Greece | 29955.7 (24992.5-35170.8) | 286.2 (238.7-336.4) | 25690.9 (21408.9-30284.4) | 286.7 (238.1-336.9) | 0.58 (-2.98-4.27) |
| Genital herpes | Greenland | 282.4 (232.2-336) | 423.5 (353-497.9) | 236.3 (196.4-275.2) | 427 (355.3-503) | 0.04 (-3.4-3.61) |
| Genital herpes | Grenada | 672.3 (555.5-786.5) | 799.8 (671-921.2) | 864.1 (727.7-990.9) | 794.5 (671.4-908.6) | -0.4 (-4.39-3.75) |
| Genital herpes | Guam | 1042.7 (862.6-1229.9) | 650.3 (543.3-754.2) | 989.3 (829.7-1146.1) | 654.1 (544.6-761.3) | 0.1 (-4.06-4.44) |
| Genital herpes | Guatemala | 63036 (52616-72982.5) | 816.7 (693.6-929.6) | 143061.1 (119953.7-165561.4) | 816.6 (693.2-936.6) | -0.51 (-4.52-3.67) |
| Genital herpes | Guinea | 41938.6 (34729.4-48634.2) | 779.2 (652.8-896) | 104609.5 (87210.7-122731.4) | 783.3 (663.4-903.5) | -0.32 (-3.93-3.42) |
| Genital herpes | Guinea-Bissau | 7421.8 (6149.1-8665.9) | 773.9 (657.2-890.1) | 16882.2 (14095.5-19692.9) | 780.1 (662.1-894.9) | 0.28 (-3.3-3.99) |
| Genital herpes | Guyana | 6877.4 (5706.1-8065.2) | 804.8 (678.8-927.9) | 6694.8 (5657.8-7726.5) | 814.5 (689.9-930.1) | -0.3 (-4.05-3.59) |
| Genital herpes | Haiti | 45158.2 (37466.6-52670.4) | 735.3 (614.4-847.8) | 101573.4 (84572.1-118398.2) | 726.2 (606.8-841.4) | -0.6 (-4.18-3.11) |
| Genital herpes | Honduras | 44337.6 (37499.4-51255.7) | 971.7 (837.6-1099.4) | 111759.5 (94364.3-127939.5) | 975.8 (836-1106.7) | -0.35 (-4.55-4.04) |
| Genital herpes | Hungary | 21482.5 (17734.7-25271) | 202.4 (166.9-237.2) | 18751 (15490.5-22369.5) | 202 (167-240) | 0.36 (-3.86-4.77) |
| Genital herpes | Iceland | 960.6 (797.9-1132.7) | 360.7 (301-425.6) | 1206.4 (1011.4-1405.7) | 359.4 (300.9-422.6) | 0.38 (-3.03-3.91) |
| Genital herpes | India | 2381627.4 (1977499.5-2797048.9) | 294.2 (244.7-345) | 4908576.2 (4080434.8-5734846) | 316.8 (263.9-369) | -0.44 (-4.05-3.31) |
| Genital herpes | Indonesia | 980132.3 (795531-1146777.3) | 509.3 (421.1-593.5) | 1557445.7 (1299466-1807799.7) | 507 (423.6-588.1) | 0.56 (-3.67-4.97) |
| Genital herpes | Iran | 250055.4 (207305.8-294618.2) | 475.7 (398.2-555.8) | 449404.4 (371377-530095) | 475.7 (397.8-554.4) | 0.47 (-3.83-4.96) |
| Genital herpes | Iraq | 67558.1 (55358.8-80871.1) | 387 (321.3-450.4) | 172203.4 (143489.3-201932.8) | 387.1 (323.3-452.9) | -0.11 (-4.46-4.44) |
| Genital herpes | Ireland | 12041.4 (10029.2-14192.1) | 335.6 (279.4-394.1) | 15529.9 (12977.7-18422.4) | 335.8 (279.6-394.5) | -0.07 (-3.39-3.35) |
| Genital herpes | Israel | 15864.2 (13337.7-18625.6) | 323.5 (271-378.9) | 28596.5 (23847.8-33773) | 316 (262.6-372.8) | 0.11 (-3.29-3.62) |
| Genital herpes | Italy | 176999.9 (147801.5-206946.3) | 299.3 (250.5-349.6) | 157726.6 (130614.5-185294.5) | 300.6 (250.1-349.4) | -0.26 (-3.6-3.19) |
| Genital herpes | Jamaica | 25756.7 (21775.7-28976.6) | 995.4 (855.6-1108.7) | 27937.8 (23831.5-32120.6) | 892.2 (765-1016.4) | -0.1 (-3.89-3.85) |
| Genital herpes | Japan | 353791.6 (290640.8-420856.5) | 267.8 (219.8-315.3) | 299034.6 (245483.9-357180.1) | 274.2 (225.5-323.5) | 0.26 (-3.62-4.31) |
| Genital herpes | Jordan | 16221.4 (13288.6-19416.3) | 434.3 (364.4-506.5) | 59695.5 (49389.1-70355.5) | 433.1 (360-510.1) | -0.02 (-4.11-4.25) |
| Genital herpes | Kazakhstan | 57681.6 (47326.8-68369.4) | 338.7 (281-400.7) | 65970.4 (54656.7-77505.9) | 339.1 (280.3-396) | 0.39 (-3.64-4.58) |
| Genital herpes | Kenya | 234184 (193783.8-272632) | 1016.5 (862.5-1161.5) | 573557.5 (479457.9-664582.3) | 1021 (867.9-1165.8) | -1.43 (-5.05-2.32) |
| Genital herpes | Kiribati | 499.9 (410.8-590.4) | 646.7 (542.6-752.3) | 837 (694-976.5) | 651 (543.9-755) | -0.14 (-4.48-4.4) |
| Genital herpes | Kuwait | 7522.2 (6125.3-8949.2) | 374 (310.7-438.8) | 22271.3 (18176.1-26522.2) | 389.2 (326.6-455.1) | -0.27 (-4.48-4.13) |
| Genital herpes | Kyrgyzstan | 14536.2 (11896.4-17196.7) | 339.1 (280.2-401.2) | 23944.4 (19562-28229.9) | 338.9 (281-396.6) | 0.86 (-3.29-5.19) |
| Genital herpes | Laos | 22116.7 (18253.7-25875.1) | 564.9 (474.4-655.2) | 45847.2 (37493.9-54217.5) | 560.8 (466.9-654.4) | 0.67 (-3.38-4.9) |
| Genital herpes | Latvia | 12159.3 (10202.1-14153.7) | 469.4 (392.7-546.6) | 7228 (6033.9-8445.8) | 467.5 (392.5-545.2) | 0.27 (-3.61-4.31) |
| Genital herpes | Lebanon | 11698 (9715.9-13880.1) | 396.1 (329.7-468.3) | 23278.2 (19126.4-27808.2) | 389 (323.6-455.5) | 0.32 (-3.65-4.46) |
| Genital herpes | Lesotho | 15434.5 (12961.6-17803) | 1067.7 (905.9-1223.9) | 25215.3 (21407-28918.9) | 1153.5 (990.2-1314.7) | -0.33 (-3.99-3.46) |
| Genital herpes | Liberia | 18166.2 (14988.1-21205.8) | 769 (646.2-886.5) | 44441 (37140.2-51702.9) | 763.6 (639.9-882.6) | -0.48 (-4.09-3.26) |
| Genital herpes | Libya | 15790.6 (12955.4-18932.9) | 382.7 (318.5-453.5) | 31217.9 (26072.1-36603.7) | 389.2 (325.6-454.5) | 0.15 (-3.89-4.36) |
| Genital herpes | Lithuania | 17473.5 (14537-20426.6) | 469 (390.5-546.3) | 10856.3 (9051.2-12675) | 468.1 (391.3-545.7) | 0.45 (-3.61-4.68) |
| Genital herpes | Luxembourg | 1341.2 (1109.3-1573.1) | 334.7 (276.4-392.2) | 2146.2 (1770.1-2537.2) | 334.6 (278.8-391.1) | -0.24 (-3.47-3.11) |
| Genital herpes | Macedonia | 5368.6 (4451-6316.6) | 255.9 (212.5-300.9) | 5913.4 (4848.1-7006.6) | 254.8 (211.2-301.6) | 0.06 (-4.35-4.68) |
| Genital herpes | Madagascar | 119030.8 (99359.7-138550.2) | 980.4 (830.6-1123.1) | 309856.1 (258996.7-358913.1) | 986.8 (838.3-1131.6) | 0.59 (-3.5-4.85) |
| Genital herpes | Malawi | 124775.7 (109763.3-138495.1) | 1183.1 (1059.4-1301.7) | 257978.5 (217709.7-298921.7) | 1149.3 (988-1308.7) | -0.93 (-4.53-2.81) |
| Genital herpes | Malaysia | 104745 (87469.3-123323.4) | 560.5 (467.3-652.7) | 200918.8 (165984.7-236094.1) | 558.7 (466.7-654.3) | -0.14 (-4.07-3.94) |
| Genital herpes | Maldives | 1149.2 (945.3-1369.3) | 561.9 (469.8-648.5) | 3615.6 (2962.9-4318.5) | 543.5 (458-631.9) | 0.08 (-4.31-4.67) |
| Genital herpes | Mali | 79496.2 (73516.5-85547.9) | 986.1 (912.3-1056.5) | 201266.9 (168975.3-231320.7) | 855.4 (733.1-971.3) | -0.76 (-4.46-3.08) |
| Genital herpes | Malta | 1268 (1049.5-1488.6) | 335.1 (279-393.6) | 1310.2 (1084.1-1551.8) | 333.2 (276.6-392.3) | 0.67 (-2.67-4.13) |
| Genital herpes | Marshall Islands | 280.9 (231.9-328) | 649.3 (546.8-751.4) | 401.2 (335.8-466.7) | 652.8 (548.6-756.9) | 0.87 (-3.11-5.02) |
| Genital herpes | Mauritania | 15193.4 (12609.1-17688.1) | 767 (649.9-882.4) | 34215.8 (28537.2-39738.1) | 772.1 (654.4-891) | -0.14 (-4.38-4.29) |
| Genital herpes | Mauritius | 7037.7 (5832.1-8252) | 560.1 (468.6-646.9) | 7139.8 (5934-8319.8) | 561.4 (468.5-655.2) | 0.69 (-3.22-4.77) |
| Genital herpes | Mexico | 552041.6 (460903.1-642757) | 627.1 (528.9-723) | 862277.9 (724472.7-993892.2) | 628.2 (527.5-722.6) | 0.09 (-3.94-4.29) |
| Genital herpes | Moldova | 21324.4 (17748.5-25038.9) | 472 (394.1-554.5) | 16121.1 (13394.2-18972.1) | 467.7 (392.6-542.4) | 0.22 (-3.64-4.23) |
| Genital herpes | Monaco | 93.2 (76.8-109.5) | 336.2 (277.5-394) | 101.6 (84.4-119.2) | 335.7 (280.7-393.2) | -0.03 (-3.59-3.66) |
| Genital herpes | Mongolia | 6850.3 (5615.4-8159.7) | 338.1 (280.3-398.2) | 11750.4 (9719.3-13880.9) | 338.3 (281.3-394.7) | 0.37 (-4.08-5.03) |
| Genital herpes | Montenegro | 1677.6 (1388.5-1973) | 255.4 (212.4-301.1) | 1571.7 (1311-1849.1) | 256.1 (213.4-301.1) | 0 (-4.31-4.51) |
| Genital herpes | Morocco | 143969.1 (133198.5-154718.8) | 534.7 (490.7-580.8) | 166469.1 (139527.9-193948.4) | 427.1 (357.8-498.1) | -0.27 (-4.32-3.94) |
| Genital herpes | Mozambique | 140657.2 (118962.7-162710.6) | 1074.4 (926-1220) | 371488.4 (315646.5-428345.6) | 1110.6 (960.5-1258.3) | -0.21 (-3.9-3.63) |
| Genital herpes | Myanmar | 241100.4 (198650-283843.3) | 561.7 (468.2-652.6) | 333252.8 (279214-389275.4) | 562.5 (471.8-657) | -0.3 (-4.05-3.6) |
| Genital herpes | Namibia | 15448.4 (12861.8-17987.6) | 1047.4 (884.9-1191.7) | 30436.5 (25802.6-34882.5) | 1103.5 (945.1-1252.6) | -0.75 (-4.39-3.03) |
| Genital herpes | Nauru | 66.7 (55.3-78.1) | 647.6 (539.8-752.1) | 77.4 (64.5-91.5) | 652.5 (546.4-766.1) | 1.02 (-3.14-5.35) |
| Genital herpes | Nepal | 49035.7 (40703.7-57517.4) | 281.4 (233.8-332.6) | 92807.4 (76866.6-109180.3) | 283.6 (236.4-333.7) | 0.14 (-3.56-3.98) |
| Genital herpes | Netherlands | 65243 (54471.7-76834.3) | 407.4 (340.5-479.2) | 62441.5 (52590.5-72977) | 408 (341-476.6) | -0.06 (-3.48-3.47) |
| Genital herpes | New Zealand | 10265.3 (8398.1-12090.9) | 284.7 (233.6-335.3) | 14411.9 (11992.4-16812) | 286.8 (238.7-334.7) | 0.13 (-3.66-4.07) |
| Genital herpes | Nicaragua | 31016.8 (25775.3-36367.2) | 817.3 (688.3-936.8) | 59329.1 (50191-69047.5) | 806 (689.5-931.2) | 0.45 (-3.69-4.78) |
| Genital herpes | Niger | 56315.7 (46535.7-66194) | 771.9 (651.3-891.6) | 176990 (145193.2-208216.3) | 765.7 (650.6-879.8) | -1.21 (-4.97-2.7) |
| Genital herpes | Nigeria | 704245.7 (587189.1-820551.2) | 802.5 (678.5-919.1) | 1865648.7 (1564160.3-2167467.7) | 813.3 (693.7-925.2) | -0.41 (-4.07-3.4) |
| Genital herpes | Niue | 13.7 (11.5-15.9) | 647.9 (540.2-752.6) | 10.2 (8.6-11.8) | 652.5 (550.3-760.9) | 0.96 (-3.19-5.28) |
| Genital herpes | North Korea | 83346.8 (69596.1-97053.1) | 384.1 (319.7-447.2) | 106781.2 (88934.1-124500.9) | 373.2 (310.8-435) | 0.61 (-3.52-4.92) |
| Genital herpes | Northern Mariana Islands | 380.8 (313.2-453.6) | 648.6 (542.7-752.4) | 303.6 (256.6-350.1) | 654.2 (549.3-753.7) | 0.11 (-4.12-4.52) |
| Genital herpes | Norway | 15027.5 (12453.6-17609.2) | 349.4 (289.3-409.2) | 17066.7 (14265.1-19990.8) | 328.9 (274-385.5) | -0.12 (-3.57-3.45) |
| Genital herpes | Oman | 7151.1 (5773.2-8547) | 359.7 (301.4-425.4) | 20441.8 (16648.4-24734.5) | 364.1 (304.8-426.7) | 0.21 (-3.9-4.5) |
| Genital herpes | Pakistan | 221617.9 (179352.1-265943.1) | 237.7 (192.7-284.8) | 567627.8 (457983.3-680308.8) | 241.3 (193.9-290.7) | 1.1 (-2.86-5.23) |
| Genital herpes | Palau | 114.1 (94.6-134) | 648.8 (540.7-753.6) | 113 (95.6-130.1) | 658.9 (551.1-768.4) | 0.87 (-3.28-5.19) |
| Genital herpes | Palestine | 7251.4 (5937.8-8707) | 391.3 (325.5-455.8) | 21180.5 (17525.8-25103) | 390.4 (327-459.2) | 0.16 (-4.12-4.63) |
| Genital herpes | Panama | 20853.7 (17468.6-24233.3) | 803.4 (684.3-922.1) | 35330.3 (29820-40594.9) | 806 (680.5-925.7) | 0.21 (-3.6-4.17) |
| Genital herpes | Papua New Guinea | 29931.3 (24856-35413.6) | 706.9 (593.9-817.4) | 78691.1 (65108-91939.3) | 710.7 (594.9-823.5) | 0.53 (-3.47-4.7) |
| Genital herpes | Paraguay | 37559.2 (31408.6-43650.3) | 935.4 (791.5-1074.3) | 74114.8 (62672.5-85437.4) | 935.6 (793.3-1070.8) | 0.46 (-3.59-4.68) |
| Genital herpes | Peru | 202499.2 (180808.9-225255.1) | 861.4 (769.9-955.5) | 334254.2 (283810-384145) | 856 (728.8-979) | 0.01 (-3.81-3.99) |
| Genital herpes | Philippines | 326299.4 (265436.7-384208.3) | 516.6 (429.8-602.8) | 633432.3 (524206.5-737813) | 521 (434.1-603.3) | 1.16 (-2.86-5.36) |
| Genital herpes | Poland | 87914.5 (72224.2-105254.8) | 222 (183.3-264.7) | 86991.8 (70345.6-104547.5) | 223.7 (183.5-265.8) | 0.18 (-4.01-4.55) |
| Genital herpes | Portugal | 34517.4 (28762.4-40514.7) | 336.3 (280.8-395.4) | 31298.7 (25989.8-36745.5) | 337.1 (283.2-395) | -0.73 (-3.96-2.61) |
| Genital herpes | Puerto Rico | 30024.5 (25281.4-34457.7) | 807.9 (679.4-928.9) | 23240.4 (19647.9-26524.6) | 808.7 (680.4-927.6) | -0.9 (-4.73-3.08) |
| Genital herpes | Qatar | 1901.1 (1535.4-2293.2) | 346.9 (290-410.4) | 13866.4 (11095.4-16917.5) | 347.5 (291-408.7) | -0.23 (-4.5-4.22) |
| Genital herpes | Romania | 60364.3 (50286.9-71268.3) | 256.3 (213-301.9) | 45270.6 (37625.8-54327) | 255.5 (212.3-299.2) | 0.28 (-3.82-4.56) |
| Genital herpes | Russian Federation | 712209.4 (591377.1-836680.8) | 456.6 (382.9-531.2) | 611631.9 (511611.1-714998.9) | 456.3 (379.6-528) | 1.19 (-2.71-5.26) |
| Genital herpes | Rwanda | 68676 (57391-80150.7) | 950.5 (810.5-1087.1) | 142779.3 (119263.3-165439.4) | 962 (814.3-1101.3) | -0.97 (-4.64-2.84) |
| Genital herpes | Saint Kitts and Nevis | 345.2 (286.6-406.3) | 802.4 (678.1-929.7) | 498.4 (419.3-567.6) | 808 (675.5-922.9) | -0.03 (-3.79-3.88) |
| Genital herpes | Saint Lucia | 1146.3 (948.1-1349.9) | 807.1 (679.6-926.1) | 1455.2 (1235.6-1664.1) | 802 (676.9-915.4) | -0.41 (-4.16-3.49) |
| Genital herpes | Saint Vincent and the Grenadines | 920.3 (760.9-1092) | 798 (672.6-919.9) | 900.9 (767.2-1034.8) | 802.1 (679.6-928.9) | -0.51 (-4.33-3.47) |
| Genital herpes | Samoa | 1094.8 (907.2-1279.8) | 650.1 (547.6-752.1) | 1361.1 (1134.1-1587) | 652.1 (547.8-756.5) | 0.97 (-3.28-5.41) |
| Genital herpes | San Marino | 84.8 (70-99.4) | 336.7 (278.2-394.6) | 96.6 (80.5-113.2) | 336.8 (281.5-394.3) | -0.07 (-3.62-3.61) |
| Genital herpes | Sao Tome and Principe | 857.8 (712.2-1007.3) | 764.5 (647.7-880.7) | 1798.2 (1502.4-2087.6) | 763.3 (641.1-886.1) | -0.49 (-4.64-3.84) |
| Genital herpes | Saudi Arabia | 60228 (48857.4-71555.1) | 371.8 (311.2-439.8) | 177629 (146506.1-211154.5) | 373.2 (313.2-435.6) | -0.03 (-4.22-4.34) |
| Genital herpes | Senegal | 48765.7 (39864.6-57518.1) | 690.7 (577.3-797.1) | 110911.6 (91554.6-131592) | 677.4 (562.2-786.8) | -0.7 (-4.3-3.04) |
| Genital herpes | Serbia | 25532.2 (21136.7-30170.5) | 255.7 (212.5-301.2) | 22614.3 (18827-26712) | 254.3 (212-299.1) | 0 (-4.35-4.55) |
| Genital herpes | Seychelles | 438 (364.5-522.6) | 560.1 (465.2-657.8) | 593.8 (497.9-686.2) | 556.4 (470-644.8) | -0.26 (-4.28-3.94) |
| Genital herpes | Sierra Leone | 31957.2 (26577.6-37546.2) | 777.6 (655.1-898.8) | 73854 (61071.2-86900.1) | 771.6 (649.4-887.2) | -0.21 (-3.84-3.55) |
| Genital herpes | Singapore | 13364.2 (10945.5-15760.3) | 354 (293.4-414.5) | 21237.2 (17279-25296.6) | 356.1 (296.3-415.8) | -0.46 (-3.96-3.18) |
| Genital herpes | Slovakia | 13945.7 (11542.8-16467) | 256.2 (213.1-301.9) | 13871.9 (11320.3-16491.7) | 255.4 (211.8-302.4) | 0.05 (-4.36-4.66) |
| Genital herpes | Slovenia | 5369.4 (4456-6356.7) | 255.9 (212.8-301.6) | 4832.7 (3985.5-5756.6) | 254.8 (212.5-299.7) | -0.2 (-4.32-4.1) |
| Genital herpes | Solomon Islands | 2104.5 (1741.6-2454.4) | 649 (546.5-751.1) | 4599.4 (3828.8-5396.5) | 652.1 (545.9-758.9) | 0.94 (-3.15-5.19) |
| Genital herpes | Somalia | 75676.6 (63104.2-87696.4) | 970.4 (822.2-1110.5) | 218776.3 (182128.4-254450.8) | 977.9 (830.1-1122.3) | -0.55 (-4.47-3.53) |
| Genital herpes | South Africa | 426585.3 (361155.9-490669.7) | 1043.5 (892.9-1180.2) | 690655.4 (593571.6-781983.6) | 1090.7 (939.5-1229.2) | -0.52 (-4.33-3.44) |
| Genital herpes | South Korea | 344626.4 (326274.2-368020.1) | 643.3 (604.9-686.4) | 213181.3 (178168.7-248506.5) | 433.3 (365.4-505.2) | 0.02 (-3.88-4.08) |
| Genital herpes | South Sudan | 60493 (50626.3-70299.9) | 974.5 (832.8-1114.8) | 97848.6 (81153.4-114507.5) | 991.1 (834-1133) | -0.09 (-3.83-3.8) |
| Genital herpes | Spain | 114675.5 (98221.4-132368.7) | 287 (245.5-332.7) | 123826.1 (101403.2-146494) | 302.9 (251.4-359.2) | -0.6 (-3.89-2.82) |
| Genital herpes | Sri Lanka | 105679.3 (87335.5-123241.4) | 561.3 (469.3-648.2) | 123621.7 (103570.2-143087.5) | 562.5 (469.3-656.8) | 0.01 (-4.41-4.63) |
| Genital herpes | Sudan | 74138 (60949.1-87904.2) | 394.9 (327.2-463.8) | 178406.2 (147215.2-210182.4) | 393 (328.7-461.5) | 0.13 (-3.67-4.08) |
| Genital herpes | Suriname | 3312.4 (2760.6-3889.8) | 795.4 (671.9-921.4) | 4660.9 (3962.7-5349.5) | 808.6 (684.6-931.9) | -0.09 (-3.89-3.85) |
| Genital herpes | Swaziland | 8487.5 (7140.1-9886) | 1057.1 (904.1-1197.5) | 15408.1 (13037.5-17663.6) | 1151.8 (985.9-1310.9) | -0.24 (-3.91-3.57) |
| Genital herpes | Sweden | 32702.5 (27172.9-38066.1) | 406.2 (339.5-471.5) | 36790.2 (30521.7-43255.1) | 395 (327.5-464) | -0.15 (-3.64-3.47) |
| Genital herpes | Switzerland | 29556.4 (27605.9-31520.2) | 407.3 (382.4-432.7) | 29995.2 (24734.8-35270.6) | 367.1 (304.9-430.6) | -0.79 (-4.08-2.62) |
| Genital herpes | Syria | 37647.9 (30905.5-44736.2) | 326.4 (271.2-384.3) | 49243.1 (41395.7-58573.1) | 345 (287.5-405.2) | 0 (-4.28-4.47) |
| Genital herpes | Taiwan | 86497.1 (71765.8-101831.4) | 376.4 (313-438.1) | 89012.1 (73626.3-105390.5) | 374.8 (310.8-441.4) | 0.43 (-3.63-4.67) |
| Genital herpes | Tajikistan | 16387.2 (13376.1-19370.7) | 338.7 (280.9-400.8) | 35456.5 (29121.3-41861.1) | 338.6 (281.4-395.4) | 0.14 (-4.11-4.59) |
| Genital herpes | Tanzania | 260248.3 (217528.7-305881.9) | 980.8 (827.3-1133.9) | 584942.8 (489911.3-684094.7) | 939.7 (797.9-1080.1) | -1.24 (-4.97-2.64) |
| Genital herpes | Thailand | 425412.5 (389426.6-461900.1) | 641.6 (584.8-696.7) | 366496.8 (309744.2-421057.8) | 598.8 (505.7-691.2) | -0.72 (-4.45-3.15) |
| Genital herpes | The Bahamas | 2411.5 (1998.2-2825.6) | 805.4 (674.5-930) | 3383.4 (2856.1-3868.7) | 813.3 (683.8-933.7) | -0.3 (-4.02-3.56) |
| Genital herpes | The Gambia | 6688.9 (5488-7837.5) | 688.9 (578-797.2) | 17747.7 (14655.5-20672.7) | 695.7 (588.6-801) | 0.14 (-3.42-3.83) |
| Genital herpes | Timor-Leste | 4925.2 (4085.9-5833.5) | 614.6 (517.1-716.9) | 8883.2 (7364.4-10401.7) | 617.2 (518.1-716.9) | -0.2 (-4.01-3.76) |
| Genital herpes | Togo | 27349.5 (22370.9-32043.4) | 772.9 (646.7-891.8) | 67454.9 (56334.9-78040.2) | 772.3 (650.9-891.8) | -0.96 (-4.47-2.68) |
| Genital herpes | Tokelau | 9.5 (8-11.1) | 648.9 (546.4-751) | 8.7 (7.3-10.1) | 652.6 (548.5-762.5) | 0.84 (-3.31-5.17) |
| Genital herpes | Tonga | 608.4 (502.2-716.8) | 646.3 (537.8-749.7) | 660.6 (555.5-778.1) | 651.5 (547-752.9) | 1.02 (-3.16-5.37) |
| Genital herpes | Trinidad and Tobago | 10358 (8688.8-12140.3) | 802 (678.3-926.8) | 10841.6 (9190-12441.9) | 806.1 (682.2-929.5) | -0.02 (-3.72-3.83) |
| Genital herpes | Tunisia | 33139.3 (27235.7-39283.1) | 391.2 (324.8-455.5) | 47450.3 (39702-55859) | 393.5 (329.4-461.5) | 0.28 (-3.98-4.72) |
| Genital herpes | Turkey | 202211.9 (166730.1-237489.5) | 341.2 (283.2-403.3) | 296461.7 (246919.4-348808.5) | 337.1 (279.4-396.8) | -0.04 (-4.32-4.44) |
| Genital herpes | Turkmenistan | 12017 (9902.3-14238.7) | 339.8 (281.8-397.9) | 18182.2 (15057.4-21298.7) | 334.6 (277.9-391.7) | -0.39 (-4.59-4) |
| Genital herpes | Tuvalu | 60.1 (50.1-70.4) | 645.6 (538.9-749.7) | 84.3 (70.7-99.1) | 654.6 (552.4-763.5) | 0.84 (-3.32-5.17) |
| Genital herpes | Uganda | 281791.8 (260236.9-298054.3) | 1502 (1392.1-1596.3) | 530095 (450517.1-614376.4) | 1116 (960-1265.6) | -0.66 (-4.3-3.11) |
| Genital herpes | Ukraine | 237156.9 (197980.6-275001.2) | 457.8 (381.6-530.9) | 184738.7 (154845.2-216464.5) | 456.9 (378.8-534) | 0.33 (-3.48-4.29) |
| Genital herpes | United Arab Emirates | 7858.1 (6322.2-9509.9) | 352.9 (292.3-414.8) | 41541 (32439.8-52121.2) | 362.5 (305.1-424.8) | 0.15 (-4.02-4.5) |
| Genital herpes | United Kingdom | 171364 (142882.6-201164.5) | 296.3 (247.5-345.4) | 192298.9 (160118.6-225479.5) | 300.4 (250.5-350.1) | 0 (-3.29-3.4) |
| Genital herpes | United States | 1683058 (1405338.1-1965901.2) | 630.5 (532.4-730.2) | 1678104.8 (1413506.6-1936732.9) | 546.5 (458.8-632.7) | -0.15 (-3.67-3.5) |
| Genital herpes | Uruguay | 18024.9 (15207.3-20725.4) | 595.9 (501.9-685.3) | 19453.6 (16362.9-22263.1) | 599.5 (502.2-689.4) | 0.16 (-3.27-3.71) |
| Genital herpes | Uzbekistan | 66827.4 (54542.1-79317) | 338.9 (281.9-401) | 124455.1 (102889.3-147312.3) | 338.8 (280.9-397.5) | 0.53 (-3.58-4.81) |
| Genital herpes | Vanuatu | 877 (727.4-1027.1) | 593.9 (498.6-685) | 1923.5 (1606.9-2266.7) | 599.3 (504.4-698.4) | 0.92 (-3.27-5.3) |
| Genital herpes | Venezuela | 164572.6 (137116.6-189894) | 807.9 (683.2-927.6) | 209093.7 (176897.5-239000.1) | 823.2 (693.7-944.6) | 0.25 (-3.64-4.3) |
| Genital herpes | Vietnam | 378164.4 (316464.1-440348.6) | 539.4 (457.7-622.4) | 562839.7 (467491.9-655435.4) | 541 (453.7-629.4) | 0.32 (-3.54-4.34) |
| Genital herpes | Yemen | 44799 (36716.7-53832.3) | 392.5 (325.9-462.4) | 132852 (109732.2-156882.4) | 391.9 (328.4-458.4) | 0.51 (-3.57-4.77) |
| Genital herpes | Zambia | 93812.8 (79296.1-109100.6) | 1113.2 (959.9-1261.9) | 250377.4 (211054.5-288218.4) | 1140 (978-1288.3) | -0.88 (-4.51-2.9) |
| Genital herpes | Zimbabwe | 117064.1 (97744.1-136495.2) | 1114.8 (953.3-1280.6) | 195921.4 (166544.4-225559.6) | 1167.5 (1002.4-1332.4) | -1.3 (-4.89-2.44) |
| Gonococcal infection | Afghanistan | 108353.5 (73795.8-158960.5) | 1098 (786.7-1557.9) | 385254.4 (262289.5-565879.1) | 1114.3 (790.7-1604.5) | 0.27 (-3.89-4.6) |
| Gonococcal infection | Albania | 74313.9 (50835.3-109823.2) | 1976.1 (1388.5-2891.3) | 47183.3 (32520.5-67378.5) | 1859.9 (1292.3-2656.4) | -0.11 (-4.53-4.51) |
| Gonococcal infection | Algeria | 203295.8 (141067-292032.5) | 743 (529.5-1046.2) | 308093.2 (223217.9-429349.2) | 715.4 (513.5-1012.1) | 0.49 (-3.58-4.74) |
| Gonococcal infection | American Samoa | 566.9 (398.5-814.8) | 1046 (762.1-1471) | 483.5 (353.9-685.9) | 976.5 (709.3-1382.8) | 0.96 (-3.19-5.29) |
| Gonococcal infection | Andorra | 80.5 (60.6-105.8) | 134.5 (101.8-179.4) | 114.8 (85.5-150.9) | 133.4 (101.6-177.2) | -0.13 (-3.69-3.57) |
| Gonococcal infection | Angola | 141534.3 (100140.9-198823.9) | 1396 (997.7-1904.3) | 412847.9 (289109.1-586651.9) | 1282.3 (920.1-1791.5) | 0.43 (-3.19-4.19) |
| Gonococcal infection | Antigua and Barbuda | 704.1 (487.9-1030.1) | 1051.3 (754.7-1518.4) | 885.4 (640.5-1268.7) | 973.2 (687.8-1419.2) | -0.21 (-4.12-3.85) |
| Gonococcal infection | Argentina | 137449.8 (103407-188335.2) | 418.7 (317.6-571.8) | 193978.5 (143117.2-264833.9) | 419.2 (306.3-574.1) | -0.06 (-3.49-3.5) |
| Gonococcal infection | Armenia | 88553.9 (60923.3-125502.1) | 2442.1 (1702.5-3491.9) | 53679.3 (38093.6-75771.4) | 2083.8 (1485.6-3023.8) | 0.92 (-3.39-5.42) |
| Gonococcal infection | Australia | 49401.8 (36580.1-66657.7) | 284.8 (210.7-386.2) | 64857.9 (49247.3-87028.1) | 276.1 (206-378.4) | -0.13 (-3.7-3.57) |
| Gonococcal infection | Austria | 10571.7 (8072.5-14029.6) | 132.9 (101.7-176.9) | 10784.9 (8089.5-14067.2) | 126.5 (94.5-167.7) | -0.37 (-3.76-3.13) |
| Gonococcal infection | Azerbaijan | 208093 (141616.1-304984.5) | 2481 (1736.9-3568.6) | 229365.9 (161408-324835.7) | 2216.2 (1525-3180.5) | 0 (-4.41-4.62) |
| Gonococcal infection | Bahrain | 6501.4 (4527.5-9254.7) | 1091.9 (772.9-1536.3) | 18839.2 (13500.6-26118.5) | 1077.7 (771-1510.5) | -0.25 (-4.4-4.07) |
| Gonococcal infection | Bangladesh | 836472 (581544.6-1189230.4) | 752.4 (540.9-1048.8) | 1122644.6 (777799.8-1578727.1) | 623.6 (435.5-870.2) | 0.02 (-4.03-4.25) |
| Gonococcal infection | Barbados | 3235.5 (2261.4-4666.1) | 1174.2 (835.6-1679.3) | 3074.1 (2237.3-4363.8) | 1142.8 (805.6-1658.5) | -0.19 (-4.02-3.8) |
| Gonococcal infection | Belarus | 201834.8 (143441.3-288478.4) | 2019.7 (1436.7-2933) | 126755.6 (91066.3-174779.9) | 1745.4 (1233.5-2502.9) | 0.97 (-3.01-5.1) |
| Gonococcal infection | Belgium | 13542.6 (10403-17608.9) | 135.8 (104.6-179.9) | 13212.2 (10053.5-17182.7) | 124.5 (94.3-166.9) | -0.14 (-3.44-3.28) |
| Gonococcal infection | Belize | 2016.2 (1370.6-3081.2) | 992.1 (715.5-1441.5) | 4680.3 (3275.2-6923.7) | 927 (662.4-1342.5) | -0.21 (-3.99-3.72) |
| Gonococcal infection | Benin | 61367.7 (43706.3-86864.7) | 1411.8 (1045.5-1957.9) | 192653.3 (138248.5-271363.3) | 1429.9 (1035.4-1959.6) | -0.5 (-4.08-3.21) |
| Gonococcal infection | Bermuda | 613.6 (444.6-894.7) | 998.3 (720-1471.1) | 433.6 (321.2-604.8) | 903.6 (642-1304.1) | -0.48 (-4.39-3.6) |
| Gonococcal infection | Bhutan | 4822.1 (3221.7-7031.7) | 673.7 (477.8-938.8) | 5319.6 (3758.3-7493.4) | 604.8 (430.7-847.4) | 0.16 (-3.49-3.96) |
| Gonococcal infection | Bolivia | 15885 (11289.4-22601.2) | 244.7 (179.4-341) | 30329.1 (22004.5-42806) | 239 (174.7-336.5) | -0.33 (-4.02-3.51) |
| Gonococcal infection | Bosnia and Herzegovina | 93519 (64885.6-132888.1) | 1899.3 (1323.3-2703.8) | 48765.7 (34672.2-69299.7) | 1817.1 (1261.1-2646.8) | 0.06 (-4.35-4.67) |
| Gonococcal infection | Botswana | 32838.1 (22951.9-48471.8) | 2322.1 (1690-3247.5) | 59380.3 (41724.9-83736.7) | 2230.8 (1571.1-3132.1) | -1.25 (-4.85-2.49) |
| Gonococcal infection | Brazil | 1406437.9 (934935.6-2075059.3) | 846 (573.5-1219.1) | 1812598.2 (1226671.4-2620705.7) | 818.6 (558.9-1185) | 0.06 (-3.86-4.14) |
| Gonococcal infection | Brunei | 1531.5 (1093.1-2122.7) | 537.6 (403.4-722.4) | 2771.8 (2074.3-3757.2) | 543.6 (408.2-735.4) | 0.32 (-3.39-4.18) |
| Gonococcal infection | Bulgaria | 146984.3 (104341.3-208755.7) | 1857.3 (1289.6-2667.8) | 86613.6 (63888.5-119434.7) | 1773.7 (1245.2-2531) | 0.4 (-3.8-4.78) |
| Gonococcal infection | Burkina Faso | 119234 (84028-171505.8) | 1379.1 (1000.7-1933.5) | 294231.6 (201766.6-432094.8) | 1298.6 (931.2-1857.7) | -1.47 (-5.19-2.41) |
| Gonococcal infection | Burundi | 75526.3 (52966.1-108164.1) | 1380 (982.9-1959.1) | 183493.2 (128909.2-266982.9) | 1323.3 (961.8-1897.6) | -1.89 (-5.64-2.01) |
| Gonococcal infection | Cambodia | 125820.3 (86996.5-177056.9) | 1218.1 (873.2-1676.7) | 212524.7 (149580.7-302415.3) | 1149.9 (815.3-1615.8) | -0.55 (-4.4-3.45) |
| Gonococcal infection | Cameroon | 200829.7 (142866.2-280003.9) | 2017.8 (1470.2-2762.8) | 652412.7 (461847.3-936979.8) | 1978.7 (1433.8-2751.3) | -0.82 (-4.42-2.91) |
| Gonococcal infection | Canada | 72744.7 (54446.2-97223) | 262.4 (195.8-356.6) | 86666.5 (66204-115432.8) | 261.1 (195.4-358.4) | 0.1 (-3.42-3.74) |
| Gonococcal infection | Cape Verde | 3926.8 (2685.5-5677.4) | 1108.6 (792.3-1567) | 7162.6 (5194.2-9922.7) | 1144.4 (845.5-1579.4) | -0.72 (-4.4-3.11) |
| Gonococcal infection | Central African Republic | 42066.2 (28778.5-61676.2) | 1530.4 (1078.8-2161.9) | 88315.3 (60987.6-129421.1) | 1521.3 (1081.8-2177.6) | -0.86 (-4.39-2.79) |
| Gonococcal infection | Chad | 72456.5 (51581.7-104010.9) | 1305.9 (949.3-1799.4) | 209836.1 (144324.5-310881.9) | 1249.3 (891.6-1786.6) | -0.59 (-4.2-3.15) |
| Gonococcal infection | Chile | 60307 (43842.9-83074.2) | 420.8 (310.7-570.1) | 76223 (57110.5-104038.9) | 404.3 (299.1-553.4) | 0.38 (-3.11-4.01) |
| Gonococcal infection | China | 17237250.3 (11373648.3-25456044.1) | 1208 (819.3-1729.3) | 13078657.9 (9382794.5-18049473.6) | 1065.7 (725.7-1523.5) | -0.08 (-4.2-4.23) |
| Gonococcal infection | Colombia | 171174.8 (120308.6-250243.8) | 464.8 (337.7-657.9) | 242261.1 (172829.2-344534.9) | 459.3 (328.6-650.3) | 0.24 (-3.66-4.29) |
| Gonococcal infection | Comoros | 6451.8 (4442.8-9370.8) | 1352.2 (974.6-1891.7) | 10662 (7608.3-15356) | 1298.1 (940-1855.5) | -0.17 (-4.66-4.53) |
| Gonococcal infection | Congo | 33369.1 (23275.1-47154.2) | 1334 (962.7-1850.3) | 74259.4 (52901.7-107163.2) | 1283.8 (921.5-1821.5) | -0.53 (-4.06-3.13) |
| Gonococcal infection | Cook Islands | 205.9 (140.2-288.8) | 983 (683.8-1376.8) | 161.1 (115.8-230.5) | 963.3 (679.8-1384.3) | 1.05 (-3.11-5.39) |
| Gonococcal infection | Costa Rica | 22001 (15227.7-31879.3) | 656.6 (465.5-931.9) | 30866 (22379.8-42945.7) | 631.7 (456.5-884.8) | 0.07 (-3.98-4.28) |
| Gonococcal infection | Cote d'Ivoire | 175525.2 (121911.8-251139.8) | 1428.5 (1015.6-1998.6) | 383665.5 (273505-551746.3) | 1336.5 (971.9-1879.5) | -1.18 (-4.71-2.49) |
| Gonococcal infection | Croatia | 85527.3 (61470.2-123071.8) | 1835.4 (1308.7-2691) | 57322.6 (41679.3-81332.7) | 1743.5 (1210.1-2518) | 0.08 (-4.16-4.5) |
| Gonococcal infection | Cuba | 125042.6 (86575.2-184642.7) | 951.4 (678.4-1378) | 87654.1 (65465.2-122584.9) | 894.1 (642.5-1303.6) | 0.92 (-3.19-5.22) |
| Gonococcal infection | Cyprus | 1065.4 (810.1-1397.2) | 134.5 (101.6-177.1) | 1762.2 (1331.6-2310.4) | 133.4 (100.4-178.9) | 0.04 (-3.63-3.85) |
| Gonococcal infection | Czech Republic | 182703.7 (133308.3-261865.9) | 1889.2 (1344.8-2744.7) | 135027.4 (98985.9-186929) | 1759.3 (1215.7-2514.8) | 0.39 (-3.87-4.83) |
| Gonococcal infection | Democratic Republic of the Congo | 461120.7 (326662.4-658752.3) | 1230.8 (890.3-1710.3) | 1149484.7 (799721.5-1616486.6) | 1219.7 (891.1-1693) | -1.3 (-4.94-2.49) |
| Gonococcal infection | Denmark | 6870.3 (5242-9159.2) | 131.9 (100.6-177.8) | 7003.6 (5313.8-9263) | 127.4 (97.3-171.8) | -0.26 (-3.73-3.34) |
| Gonococcal infection | Djibouti | 6409.6 (4349.4-9450.7) | 1354.5 (967.6-1931.3) | 17839.4 (12796.7-25403.5) | 1295.2 (937.9-1837.1) | -0.19 (-3.94-3.7) |
| Gonococcal infection | Dominica | 863.8 (594-1294.2) | 1069.9 (746.3-1557.2) | 693.8 (500.3-1014.5) | 1001.1 (723.3-1481.5) | -0.21 (-4.16-3.9) |
| Gonococcal infection | Dominican Republic | 92162.4 (62355.5-137316.8) | 1098.8 (777.5-1582.6) | 119030.3 (85108.9-171631.9) | 1003.9 (724.8-1439.2) | -0.71 (-4.46-3.19) |
| Gonococcal infection | Ecuador | 24435.1 (17300.3-35742.9) | 227.2 (165-318.2) | 43015 (31048.6-59581.5) | 224.6 (163.8-309.5) | 0.54 (-3.22-4.44) |
| Gonococcal infection | Egypt | 763688.5 (526770.1-1116926) | 1289.2 (912.9-1829.6) | 1342089.4 (964497.6-1927218) | 1210.3 (877.3-1722.4) | 0 (-4.33-4.53) |
| Gonococcal infection | El Salvador | 38283.1 (26421.8-56382.2) | 662 (474.1-945.5) | 43796.1 (30566.4-62916.4) | 627.5 (447.1-896.2) | -0.23 (-4.16-3.86) |
| Gonococcal infection | Equatorial Guinea | 5368.9 (3746.9-7584.6) | 1332.7 (941-1817) | 23752.3 (16193.6-34428.1) | 1319.5 (936.4-1844.4) | 0.57 (-2.97-4.24) |
| Gonococcal infection | Eritrea | 49882.4 (34410-72677.2) | 1410 (1018.1-1970.2) | 97455.3 (69038.5-141102.7) | 1324.2 (949.3-1889.3) | -1.3 (-5.1-2.65) |
| Gonococcal infection | Estonia | 30726.4 (21787.3-43135.7) | 2097 (1480.8-2974.9) | 17115.7 (12566.3-23569.2) | 1716 (1218.6-2446.7) | 0.65 (-3.26-4.73) |
| Gonococcal infection | Ethiopia | 484152.3 (323943.5-709737) | 999.3 (694.9-1426.8) | 1104683.3 (725316.1-1634648.6) | 911.3 (626.2-1315.7) | -1.38 (-5.11-2.5) |
| Gonococcal infection | Federated States of Micronesia | 1183.9 (826.4-1720.7) | 1102.6 (783.9-1555.8) | 1206.7 (843-1772.5) | 1038.7 (734.8-1496.8) | 0.99 (-3.14-5.3) |
| Gonococcal infection | Fiji | 10533.5 (7637.5-14752.4) | 1228.5 (911.1-1689.1) | 10581.1 (7595.6-15016.9) | 1120.7 (801-1594.7) | 0.08 (-4.15-4.5) |
| Gonococcal infection | Finland | 6679.5 (5146.1-8694.5) | 135.2 (102.5-180.8) | 6083 (4673.5-7864.6) | 123.6 (94.1-164.1) | -0.17 (-3.68-3.47) |
| Gonococcal infection | France | 77603.7 (59690.3-102402.2) | 134.4 (103.9-178.8) | 77529.7 (58483.4-102142) | 127.6 (96.5-169.5) | 0.07 (-3.21-3.47) |
| Gonococcal infection | Gabon | 13388.9 (9338.4-19045.5) | 1339.3 (961.6-1848.5) | 24367.2 (17069.5-34223.2) | 1235.5 (882.3-1712.4) | -0.52 (-4.04-3.14) |
| Gonococcal infection | Georgia | 125303.6 (87909.7-176830.4) | 2271.1 (1623.2-3220.3) | 61219.6 (43942-86831.9) | 2118.7 (1487-3072.2) | 0.67 (-3.52-5.04) |
| Gonococcal infection | Germany | 111474.5 (85759.6-144618.6) | 137 (104.5-181.1) | 100576 (76772.8-132382.4) | 130.7 (99.3-176.3) | -0.12 (-3.64-3.52) |
| Gonococcal infection | Ghana | 300333.4 (210059.5-427994.9) | 1965.9 (1433.7-2713.1) | 716511.1 (503986.9-1025996.5) | 1923 (1382.5-2703.6) | -0.48 (-4.16-3.34) |
| Gonococcal infection | Greece | 13661.4 (10450.6-17936) | 128.6 (97.2-170.4) | 11324.1 (8470.7-14813.2) | 123.8 (95-165) | 0.58 (-2.98-4.27) |
| Gonococcal infection | Greenland | 182.2 (131.1-246.7) | 286.8 (214.2-388.7) | 154.1 (116.1-207.5) | 276.8 (205.9-380) | 0.04 (-3.4-3.61) |
| Gonococcal infection | Grenada | 979.8 (673.9-1455.5) | 1089.3 (775.9-1593.7) | 1114.6 (809.5-1625.2) | 1003.1 (720.4-1457.4) | -0.4 (-4.39-3.75) |
| Gonococcal infection | Guam | 1569.5 (1080.3-2270.4) | 969.1 (680.4-1376) | 1417.8 (1028.1-2028) | 929.2 (670.5-1333.7) | 0.1 (-4.06-4.44) |
| Gonococcal infection | Guatemala | 76593.9 (52406.8-113111.2) | 930.7 (663.1-1336) | 148449.9 (100884.8-216799.8) | 807.8 (563.5-1166) | -0.51 (-4.52-3.67) |
| Gonococcal infection | Guinea | 69064.4 (48556.3-99067.3) | 1278.9 (914.7-1801.8) | 163903.6 (115837.2-232734.8) | 1217.4 (877.4-1712.7) | -0.32 (-3.93-3.42) |
| Gonococcal infection | Guinea-Bissau | 12769.7 (8985.7-18263.5) | 1309.1 (949.6-1827.2) | 26672.5 (18903.5-38246.1) | 1235.5 (905.7-1731.3) | 0.28 (-3.3-3.99) |
| Gonococcal infection | Guyana | 10151 (6897.6-15087.3) | 1108.2 (777.1-1600.7) | 8581.5 (6037.6-12595.6) | 1010.5 (715.1-1469.8) | -0.3 (-4.05-3.59) |
| Gonococcal infection | Haiti | 70434 (48398.5-104602.9) | 1067.8 (740-1544.1) | 132019.6 (92778.7-191886.4) | 931.4 (667-1329.5) | -0.6 (-4.18-3.11) |
| Gonococcal infection | Honduras | 33530.1 (23270.3-49437.9) | 706.5 (503.3-1008.5) | 80111.5 (55430.5-117085.7) | 681.9 (483-983.9) | -0.35 (-4.55-4.04) |
| Gonococcal infection | Hungary | 184815.6 (134717.2-261399) | 1951.3 (1392.4-2796) | 131720.2 (96059.5-187262.7) | 1785.5 (1263.3-2613.4) | 0.36 (-3.86-4.77) |
| Gonococcal infection | Iceland | 350 (265.8-468.5) | 135.6 (103.2-181.6) | 427.5 (324.5-548.5) | 126.4 (95.5-167.4) | 0.38 (-3.03-3.91) |
| Gonococcal infection | India | 12427483.8 (8378555.6-18092865.3) | 1396.1 (978.5-1997) | 21041216.1 (14450902.2-30730697.7) | 1325 (920.1-1917.5) | -0.44 (-4.05-3.31) |
| Gonococcal infection | Indonesia | 2803902.8 (1921860.8-4043516.8) | 1385.3 (967.9-1932.7) | 4136753 (2870120.8-5836957.1) | 1371.5 (941.4-1937.2) | 0.56 (-3.67-4.97) |
| Gonococcal infection | Iran | 676978.3 (442650.6-995021) | 1159.6 (779.5-1669.3) | 970301 (683785.5-1371821.1) | 1150.6 (778.8-1663.5) | 0.47 (-3.83-4.96) |
| Gonococcal infection | Iraq | 212595.7 (143955.4-311061) | 1082 (775.8-1534.8) | 467913.6 (330457.3-662615.5) | 1005.7 (721.8-1405.9) | -0.11 (-4.46-4.44) |
| Gonococcal infection | Ireland | 4776.5 (3634.2-6419.6) | 131.7 (101.1-175.5) | 5910.8 (4485.4-7862.7) | 124.6 (93.9-169.5) | -0.07 (-3.39-3.35) |
| Gonococcal infection | Israel | 6592.5 (4973.6-9029.3) | 134.9 (102.8-182.1) | 11774 (8974.7-15490.7) | 128.7 (97.6-170.5) | 0.11 (-3.29-3.62) |
| Gonococcal infection | Italy | 86787.9 (65916.5-116141.4) | 146.1 (109.8-191.9) | 77648.5 (59346.5-102070.5) | 144.8 (107.3-192.6) | -0.26 (-3.6-3.19) |
| Gonococcal infection | Jamaica | 27565.9 (18885.4-41440) | 1025.8 (734.9-1496.9) | 30042.6 (21397.6-44410.6) | 970.9 (693.2-1412) | -0.1 (-3.89-3.85) |
| Gonococcal infection | Japan | 718844 (536933.2-955906) | 555.6 (404.1-737.4) | 580444.7 (445367.3-777082.2) | 572.4 (414.7-765.4) | 0.26 (-3.62-4.31) |
| Gonococcal infection | Jordan | 42086.7 (28565.1-62292.6) | 975.4 (691.4-1390.1) | 135606.9 (96181.4-192079.6) | 948.6 (680.1-1328) | -0.02 (-4.11-4.25) |
| Gonococcal infection | Kazakhstan | 405118.3 (284510.2-584768.8) | 2333 (1639.7-3369.5) | 368348.5 (263626.1-524367.3) | 2130.1 (1502-3134.5) | 0.39 (-3.64-4.58) |
| Gonococcal infection | Kenya | 245777.4 (156169.3-362902.9) | 1025.2 (689.9-1478.5) | 571596.3 (379455.4-841397.3) | 991.1 (670.1-1415.1) | -1.43 (-5.05-2.32) |
| Gonococcal infection | Kiribati | 509.5 (347.8-763) | 629.8 (445.2-910.7) | 798.9 (560.4-1184.4) | 612.8 (436.3-894.8) | -0.14 (-4.48-4.4) |
| Gonococcal infection | Kuwait | 24170.7 (16830-34387.1) | 1202.6 (859.7-1701.4) | 51918.1 (38422.7-71159.8) | 1078.7 (773.9-1535.4) | -0.27 (-4.48-4.13) |
| Gonococcal infection | Kyrgyzstan | 108509 (74727.8-158941.3) | 2286.1 (1623.6-3322) | 152280.9 (106017.3-218474.1) | 2170.9 (1530.6-3150.5) | 0.86 (-3.29-5.19) |
| Gonococcal infection | Laos | 52875 (37532.2-76333.3) | 1284.5 (930-1795) | 98578.4 (69935-135584.3) | 1199.4 (859.4-1635.8) | 0.67 (-3.38-4.9) |
| Gonococcal infection | Latvia | 51530.2 (36374.2-72714.5) | 2064.6 (1449.1-2935.3) | 24276.4 (17682.2-33709.8) | 1771.3 (1240.7-2544.9) | 0.27 (-3.61-4.31) |
| Gonococcal infection | Lebanon | 31356.1 (22358.8-45614.8) | 995.2 (722-1406) | 54616 (39304.3-75816.4) | 975.9 (699.6-1374.2) | 0.32 (-3.65-4.46) |
| Gonococcal infection | Lesotho | 38853.2 (27768.5-55778.2) | 2622.1 (1880.9-3632) | 57213 (39462-81432.4) | 2607 (1843-3643) | -0.33 (-3.99-3.46) |
| Gonococcal infection | Liberia | 30186.8 (21490.5-43718) | 1287.2 (936.4-1812.2) | 71524.4 (50665.7-103064.6) | 1230.1 (893.4-1723.6) | -0.48 (-4.09-3.26) |
| Gonococcal infection | Libya | 48027.4 (32914.4-71463.9) | 1043.5 (738.9-1512.2) | 77127.5 (55484.7-108854.3) | 1002.3 (717.8-1424) | 0.15 (-3.89-4.36) |
| Gonococcal infection | Lithuania | 73542.3 (51189.3-104140.6) | 2010.4 (1394.9-2888.6) | 38710.8 (27707.9-54239.1) | 1800.4 (1261.6-2569.4) | 0.45 (-3.61-4.68) |
| Gonococcal infection | Luxembourg | 539.5 (413.7-703.4) | 139.5 (106.5-182.9) | 849.8 (648-1105.7) | 133.3 (101.3-179.6) | -0.24 (-3.47-3.11) |
| Gonococcal infection | Macedonia | 38782.9 (27585.3-55783.5) | 1858.1 (1324.5-2680.4) | 35257.2 (25392.7-48576.4) | 1820.2 (1282.1-2585.2) | 0.06 (-4.35-4.68) |
| Gonococcal infection | Madagascar | 165217 (114289.7-233443.9) | 1358 (975.2-1900.6) | 419262.1 (288430.4-607405.2) | 1324.3 (968.8-1876.5) | 0.59 (-3.5-4.85) |
| Gonococcal infection | Malawi | 166610.5 (113964.3-242258) | 1604.3 (1152.7-2273.3) | 361373.1 (246630.4-523231.8) | 1586.3 (1119.3-2215) | -0.93 (-4.53-2.81) |
| Gonococcal infection | Malaysia | 226166.4 (158122.5-313566.6) | 1183.9 (844.4-1631.6) | 403627.4 (288369.6-564571.7) | 1142.1 (819.1-1598) | -0.14 (-4.07-3.94) |
| Gonococcal infection | Maldives | 2810.4 (1980.8-4055.2) | 1271.7 (920.3-1763.3) | 7855.2 (5560.7-11118.4) | 1312 (956.6-1830) | 0.08 (-4.31-4.67) |
| Gonococcal infection | Mali | 98069.4 (69997-139711.1) | 1237.8 (913.3-1740.2) | 280904.4 (197470.9-408736.1) | 1187 (873.5-1666.3) | -0.76 (-4.46-3.08) |
| Gonococcal infection | Malta | 505.1 (389.7-665.9) | 136.1 (104.4-183.9) | 508.5 (387.1-663.6) | 131 (99.5-176.1) | 0.67 (-2.67-4.13) |
| Gonococcal infection | Marshall Islands | 496.5 (334.9-728.5) | 1069.1 (754.5-1522.1) | 643.2 (446-930.8) | 1029 (721.2-1483.7) | 0.87 (-3.11-5.02) |
| Gonococcal infection | Mauritania | 26126.5 (18356.2-37201.5) | 1300.9 (932.5-1819.6) | 54444.6 (38843.8-78061.4) | 1207.2 (872.6-1682.2) | -0.14 (-4.38-4.29) |
| Gonococcal infection | Mauritius | 14890.6 (10476.2-21336.4) | 1184.3 (845.6-1675.3) | 13149 (9664.9-18037) | 1048.1 (766.5-1451.1) | 0.69 (-3.22-4.77) |
| Gonococcal infection | Mexico | 721615.8 (471764.3-1065510.8) | 754.6 (507.2-1087.2) | 981692 (666720.6-1423640.7) | 716.4 (488.3-1037.1) | 0.09 (-3.94-4.29) |
| Gonococcal infection | Moldova | 88495.9 (63316.1-126303) | 2050.2 (1479.4-2967.3) | 55227.4 (40310.2-76376.3) | 1843.6 (1306.4-2642.6) | 0.22 (-3.64-4.23) |
| Gonococcal infection | Monaco | 35.7 (27.1-47.4) | 129.5 (98.3-172.3) | 41.9 (31.2-56.4) | 128.5 (98-173.1) | -0.03 (-3.59-3.66) |
| Gonococcal infection | Mongolia | 56460.8 (39093.8-83060.8) | 2318.9 (1649.1-3333.6) | 70089.7 (49063.7-100447.1) | 2224.4 (1544.2-3229.9) | 0.37 (-4.08-5.03) |
| Gonococcal infection | Montenegro | 12651.6 (8734.2-18276.9) | 1930.6 (1342.8-2809.4) | 10185.3 (7245.9-14392.3) | 1872.6 (1293-2715.7) | 0 (-4.31-4.51) |
| Gonococcal infection | Morocco | 317574.2 (222375.3-463672.1) | 1136.9 (808.6-1619.9) | 413679.7 (293699.9-592689.4) | 1073.4 (756.6-1543.2) | -0.27 (-4.32-3.94) |
| Gonococcal infection | Mozambique | 216032.6 (152529.7-307879.1) | 1661.7 (1205.9-2332.8) | 525213.4 (360312.7-765822.7) | 1574.4 (1136.3-2235) | -0.21 (-3.9-3.63) |
| Gonococcal infection | Myanmar | 575641.3 (405210-813387.7) | 1283 (917.4-1775.7) | 707205.2 (510803.7-1021892.2) | 1176.5 (850.7-1688.1) | -0.3 (-4.05-3.6) |
| Gonococcal infection | Namibia | 41443.9 (28481.8-59551.9) | 2713.6 (1937-3746.9) | 68248.9 (47105.4-99131.7) | 2486 (1749.7-3550) | -0.75 (-4.39-3.03) |
| Gonococcal infection | Nauru | 106.5 (74.5-155.5) | 998.9 (712.2-1443.9) | 119.1 (83.6-172.9) | 967.4 (691-1371.8) | 1.02 (-3.14-5.35) |
| Gonococcal infection | Nepal | 128100.4 (90687.2-181019.9) | 660.4 (472.7-911.8) | 204218.7 (141880.5-289912.7) | 576.2 (411.6-800.5) | 0.14 (-3.56-3.98) |
| Gonococcal infection | Netherlands | 19991.3 (15278.1-26200.8) | 128.5 (97.5-167.4) | 20005.1 (15141.1-26532) | 123.9 (94.1-167.3) | -0.06 (-3.48-3.47) |
| Gonococcal infection | New Zealand | 11848.4 (8409.6-16111.6) | 332.8 (237.1-450.9) | 16155.7 (11722.1-21761.8) | 329.3 (234.3-448.2) | 0.13 (-3.66-4.07) |
| Gonococcal infection | Nicaragua | 38242.5 (26000.9-57330.8) | 935.3 (671.9-1360.8) | 61271 (42728.1-89818.7) | 829.1 (590.9-1206.5) | 0.45 (-3.69-4.78) |
| Gonococcal infection | Niger | 113478.6 (79972.1-161661.8) | 1527.1 (1113.8-2128.3) | 333215 (228085.5-477955.4) | 1378.1 (993.8-1915.9) | -1.21 (-4.97-2.7) |
| Gonococcal infection | Nigeria | 1161383.4 (803512.2-1670244.4) | 1322.3 (939.2-1829.9) | 2886014.9 (1992119-4113746.3) | 1226.1 (866.1-1691.6) | -0.41 (-4.07-3.4) |
| Gonococcal infection | Niue | 21.7 (15.2-31.4) | 976.2 (683.3-1408.8) | 15.1 (10.8-21.6) | 949 (666.5-1359.9) | 0.96 (-3.19-5.28) |
| Gonococcal infection | North Korea | 200460 (139238.2-288337.3) | 886.9 (617.9-1257.3) | 235451.3 (170800.3-326940.5) | 884.3 (637.4-1250.3) | 0.61 (-3.52-4.92) |
| Gonococcal infection | Northern Mariana Islands | 583 (402.9-835.6) | 1028.1 (731.4-1466.5) | 444 (325.6-631.9) | 954 (687.4-1373.7) | 0.11 (-4.12-4.52) |
| Gonococcal infection | Norway | 6233.9 (4638.7-8248.1) | 147.4 (109.7-196.2) | 8128.7 (6214.9-10771.5) | 156.2 (116-208.4) | -0.12 (-3.57-3.45) |
| Gonococcal infection | Oman | 22397.7 (15895.5-31795.6) | 1103.2 (797.7-1569.2) | 53432.5 (37472.5-74034.5) | 1044.9 (739.6-1470.2) | 0.21 (-3.9-4.5) |
| Gonococcal infection | Pakistan | 390521 (267692.3-555574.3) | 361.4 (255.7-492.2) | 863392.9 (591108.4-1208477) | 337.9 (238.9-467) | 1.1 (-2.86-5.23) |
| Gonococcal infection | Palau | 177.4 (122.1-257) | 987.3 (693.3-1402.3) | 157.4 (115.1-219.3) | 951.6 (659.7-1367.7) | 0.87 (-3.28-5.19) |
| Gonococcal infection | Palestine | 22489.8 (15341-32705.1) | 1049.7 (760.3-1488.9) | 59873.5 (41963.8-86617.7) | 1020.4 (734.8-1451.7) | 0.16 (-4.12-4.63) |
| Gonococcal infection | Panama | 16658.8 (11729-24063.7) | 615.5 (447.7-871.6) | 27686.6 (19885.3-39078.6) | 623.6 (449.2-877) | 0.21 (-3.6-4.17) |
| Gonococcal infection | Papua New Guinea | 140714.2 (99442.5-194613.2) | 3129.4 (2279.4-4232.2) | 348675.6 (249044.2-486430.1) | 3078.3 (2249.7-4244.8) | 0.53 (-3.47-4.7) |
| Gonococcal infection | Paraguay | 32883 (22773.5-47645.5) | 775.9 (553.6-1110) | 59009.9 (41591.5-85853.5) | 744.3 (531.7-1079.5) | 0.46 (-3.59-4.68) |
| Gonococcal infection | Peru | 54371.5 (38695.4-76056.8) | 233.2 (169.6-315.1) | 89181.4 (64365-124056.4) | 231.7 (168-321) | 0.01 (-3.81-3.99) |
| Gonococcal infection | Philippines | 843324.3 (577032.1-1207866.5) | 1252 (879.9-1728.3) | 1588165.8 (1110026.5-2201365.2) | 1277.8 (902.3-1750.1) | 1.16 (-2.86-5.36) |
| Gonococcal infection | Poland | 703504.9 (488079-991997.1) | 1966.3 (1330.6-2795.7) | 542104.1 (382508.3-734389) | 1780 (1215.2-2510.5) | 0.18 (-4.01-4.55) |
| Gonococcal infection | Portugal | 14461.6 (11103.5-19133.7) | 137.9 (105-183.8) | 12814.3 (9655.1-16923.9) | 132.5 (99.1-176.9) | -0.73 (-3.96-2.61) |
| Gonococcal infection | Puerto Rico | 37705.4 (26627.5-55360.4) | 993.8 (706.1-1445.2) | 26681.3 (19279.9-38247.1) | 934.5 (658.8-1360.6) | -0.9 (-4.73-3.08) |
| Gonococcal infection | Qatar | 5848 (4059.5-8208.1) | 1122.6 (810.6-1581.3) | 37445.4 (26640.3-52752.8) | 1084.8 (781.5-1552) | -0.23 (-4.5-4.22) |
| Gonococcal infection | Romania | 445649.4 (312466.1-650004.5) | 1922.4 (1345.5-2817.3) | 262673 (194126.8-369167.9) | 1807.5 (1262.1-2621.6) | 0.28 (-3.82-4.56) |
| Gonococcal infection | Russian Federation | 3185843.7 (2171878-4485753.4) | 2194.3 (1463.6-3161.5) | 2437051.1 (1727863.3-3414025.7) | 2094.4 (1392.2-3032.3) | 1.19 (-2.71-5.26) |
| Gonococcal infection | Rwanda | 99958.1 (69508.3-144691.3) | 1382.6 (991.7-1965.4) | 190869.2 (132236.3-278499.2) | 1280.5 (919.5-1816.3) | -0.97 (-4.64-2.84) |
| Gonococcal infection | Saint Kitts and Nevis | 457 (310.3-686.7) | 1012.3 (712.5-1488.7) | 561.1 (405.1-808.5) | 944.4 (664.6-1394.6) | -0.03 (-3.79-3.88) |
| Gonococcal infection | Saint Lucia | 1696.9 (1149.6-2581.8) | 1090.3 (777.6-1590.2) | 1741.5 (1265-2465.2) | 992.8 (716-1420.9) | -0.41 (-4.16-3.49) |
| Gonococcal infection | Saint Vincent and the Grenadines | 1404 (943.9-2107.3) | 1111.9 (784.3-1599) | 1143.4 (821.2-1671.1) | 1011.4 (714.4-1497.6) | -0.51 (-4.33-3.47) |
| Gonococcal infection | Samoa | 2321 (1594-3382.8) | 1238 (876.9-1725.8) | 2685.8 (1896.4-3796.6) | 1222.2 (876.9-1721.3) | 0.97 (-3.28-5.41) |
| Gonococcal infection | San Marino | 32.4 (24.8-42.6) | 129.5 (98.7-171.6) | 39.3 (29.6-52.9) | 128 (96-170.8) | -0.07 (-3.62-3.61) |
| Gonococcal infection | Sao Tome and Principe | 1426.8 (983.1-2045.5) | 1218.6 (880.2-1700.6) | 2850.8 (2039.8-4049.3) | 1204.6 (881.2-1675.5) | -0.49 (-4.64-3.84) |
| Gonococcal infection | Saudi Arabia | 154814.6 (108486.4-229501.2) | 893.9 (636.4-1291.6) | 375582.1 (266197.9-524749.4) | 855.1 (617.9-1226.1) | -0.03 (-4.22-4.34) |
| Gonococcal infection | Senegal | 38186.9 (27298.6-53615.7) | 518.2 (380.8-707.1) | 77761.3 (56319.2-110735.1) | 462.2 (344.7-633.6) | -0.7 (-4.3-3.04) |
| Gonococcal infection | Serbia | 179766.4 (126692.6-254849.8) | 1921.3 (1350.6-2738.8) | 142104.5 (101840.6-202081.1) | 1856.8 (1311.8-2696.4) | 0 (-4.35-4.55) |
| Gonococcal infection | Seychelles | 1002.3 (696.5-1405.9) | 1238.1 (882.4-1706.4) | 1284.2 (928.6-1737.9) | 1244.9 (893.1-1727.7) | -0.26 (-4.28-3.94) |
| Gonococcal infection | Sierra Leone | 51073 (36057.8-72622.6) | 1249.2 (914.7-1734.7) | 116715.3 (82648.7-168220.6) | 1212.4 (895.1-1692.9) | -0.21 (-3.84-3.55) |
| Gonococcal infection | Singapore | 17478.7 (12832.3-23537.5) | 482.7 (354.8-640.9) | 24004.1 (18208.9-31438.7) | 448.9 (334.4-608.1) | -0.46 (-3.96-3.18) |
| Gonococcal infection | Slovakia | 97365.7 (69161.4-141423.5) | 1886.9 (1333.2-2772.9) | 80029.9 (57301.3-110604.5) | 1862.4 (1264-2678) | 0.05 (-4.36-4.66) |
| Gonococcal infection | Slovenia | 35752.5 (25306.3-51357.1) | 1828.7 (1284.5-2639.5) | 24588.4 (18112.4-34594.7) | 1658.7 (1164.6-2393.2) | -0.2 (-4.32-4.1) |
| Gonococcal infection | Solomon Islands | 2678.5 (1841.7-3888.1) | 755 (540.3-1062.9) | 5177 (3619.4-7457) | 705.2 (498.1-1000) | 0.94 (-3.15-5.19) |
| Gonococcal infection | Somalia | 156687.3 (113406.8-216819.3) | 2077.6 (1507.2-2799.6) | 466785.5 (326590-660099.1) | 2113.4 (1522.2-2887.1) | -0.55 (-4.47-3.53) |
| Gonococcal infection | South Africa | 1986404.6 (1371268.4-2777238.1) | 4785.7 (3414.2-6560.2) | 2802719.5 (1946416.6-3914845.5) | 4603.9 (3194-6431.4) | -0.52 (-4.33-3.44) |
| Gonococcal infection | South Korea | 254311.7 (187850.4-343623.9) | 492.5 (373.1-660.5) | 233896.3 (175897.9-309546.7) | 481.6 (354.1-654.1) | 0.02 (-3.88-4.08) |
| Gonococcal infection | South Sudan | 90286.7 (61677.9-129713.8) | 1443.9 (1026.8-2028.4) | 139491.6 (97458.4-203488.5) | 1371.5 (989.3-1911.5) | -0.09 (-3.83-3.8) |
| Gonococcal infection | Spain | 77801.3 (59762.9-101949) | 191.7 (145.4-253.5) | 77232.5 (57925.2-102342.3) | 180.3 (136.7-241.2) | -0.6 (-3.89-2.82) |
| Gonococcal infection | Sri Lanka | 251496.4 (180150.5-343631.5) | 1318.8 (957.3-1777.7) | 281505 (206075.5-387404.6) | 1283.8 (930.6-1786.2) | 0.01 (-4.41-4.63) |
| Gonococcal infection | Sudan | 286475.6 (195000.4-429927.2) | 1367.4 (960.1-1995) | 639854.1 (452072.2-929193.7) | 1291 (932.9-1850.4) | 0.13 (-3.67-4.08) |
| Gonococcal infection | Suriname | 4944.4 (3432.5-7276.5) | 1095.2 (778.7-1577) | 5730.7 (4109.7-8167.5) | 983.2 (694.5-1412.6) | -0.09 (-3.89-3.85) |
| Gonococcal infection | Swaziland | 22215.9 (15411.1-33293.3) | 2660 (1897.3-3798.6) | 33722.7 (23220.7-49157.7) | 2546.3 (1788.4-3657.2) | -0.24 (-3.91-3.57) |
| Gonococcal infection | Sweden | 12610.4 (9397.2-16656.6) | 151.9 (111.9-199.8) | 14099.2 (10641.3-18914.6) | 149.7 (110.3-202) | -0.15 (-3.64-3.47) |
| Gonococcal infection | Switzerland | 9200.7 (7044.4-11993.8) | 132 (101.2-176.3) | 10735.9 (8110.8-13944.4) | 129.1 (98.6-173.6) | -0.79 (-4.08-2.62) |
| Gonococcal infection | Syria | 140885.6 (95915.7-212868.6) | 1056 (744.9-1521.4) | 147092.1 (102534-219017.7) | 953.7 (684.3-1383.8) | 0 (-4.28-4.47) |
| Gonococcal infection | Taiwan | 201093 (142013.9-286260.9) | 866.8 (620.4-1214.1) | 170410.9 (125589.3-233096.7) | 836.9 (602.6-1188.8) | 0.43 (-3.63-4.67) |
| Gonococcal infection | Tajikistan | 146417.7 (98883.1-215251.1) | 2535.9 (1771.5-3638.2) | 264813.1 (181899.6-379580) | 2451.5 (1700.4-3466.9) | 0.14 (-4.11-4.59) |
| Gonococcal infection | Tanzania | 435591.2 (290737.1-657166.9) | 1684.3 (1156.4-2429.1) | 948963.3 (631468.5-1403251.5) | 1535.3 (1035.9-2228.6) | -1.24 (-4.97-2.64) |
| Gonococcal infection | Thailand | 796027.3 (555975.9-1112682.4) | 1186.1 (845.2-1624.9) | 678835.7 (503328.1-904665.2) | 1121.2 (803.3-1542.3) | -0.72 (-4.45-3.15) |
| Gonococcal infection | The Bahamas | 3188.7 (2220.1-4737.1) | 1033.3 (741.1-1500.4) | 3943.5 (2850.7-5642.3) | 948.6 (679.3-1377.6) | -0.3 (-4.02-3.56) |
| Gonococcal infection | The Gambia | 12183.2 (8459-17669.2) | 1248.9 (905.5-1733.3) | 31078.1 (21820.5-44532) | 1199.7 (874.6-1693.1) | 0.14 (-3.42-3.83) |
| Gonococcal infection | Timor-Leste | 10829 (7642.8-15501.9) | 1316.2 (940-1822.8) | 18297.1 (12612.7-26729.4) | 1178.9 (854.5-1656.6) | -0.2 (-4.01-3.76) |
| Gonococcal infection | Togo | 45216.2 (30919.3-65321.5) | 1252.7 (896.2-1763) | 105386.1 (74971.9-152413.6) | 1199 (870.7-1696.3) | -0.96 (-4.47-2.68) |
| Gonococcal infection | Tokelau | 15.9 (10.9-23.6) | 1015.5 (715.9-1442.2) | 13.2 (9.4-19) | 967.6 (676.9-1384.4) | 0.84 (-3.31-5.17) |
| Gonococcal infection | Tonga | 1266.4 (868.8-1818.1) | 1202.9 (847.3-1664.5) | 1244.9 (872.3-1815.5) | 1150.3 (819-1649.4) | 1.02 (-3.16-5.37) |
| Gonococcal infection | Trinidad and Tobago | 12723.7 (8898.2-18329.2) | 981.3 (694.4-1409.6) | 11625.8 (8541.6-16414.5) | 924.8 (662.4-1347.8) | -0.02 (-3.72-3.83) |
| Gonococcal infection | Tunisia | 136321.7 (97339.1-186977.8) | 1445.4 (1070-1938.2) | 136324.8 (99097.7-189090.7) | 1220.9 (870.7-1722.4) | 0.28 (-3.98-4.72) |
| Gonococcal infection | Turkey | 1220833.4 (876294.2-1698852) | 1871.9 (1391.6-2549.3) | 1261717.4 (924824.6-1808558.7) | 1497.2 (1080.3-2145.4) | -0.04 (-4.32-4.44) |
| Gonococcal infection | Turkmenistan | 98943.3 (67491-145604.6) | 2415.5 (1713-3492.4) | 109923 (77681.8-155955.5) | 2021.2 (1422.3-2873) | -0.39 (-4.59-4) |
| Gonococcal infection | Tuvalu | 97.1 (67.2-141.1) | 1026.5 (721.7-1481.4) | 129.9 (90.4-186.5) | 975.5 (690.5-1393.8) | 0.84 (-3.32-5.17) |
| Gonococcal infection | Uganda | 435911.5 (306555.6-611490.8) | 2513.9 (1852.5-3450.8) | 1173906.2 (813807.4-1689903.1) | 2507.4 (1812-3442.2) | -0.66 (-4.3-3.11) |
| Gonococcal infection | Ukraine | 1099414 (760440.5-1565748.2) | 2238.2 (1510.3-3256.7) | 696278.9 (501663.4-966150.7) | 2009.4 (1341.6-2926.4) | 0.33 (-3.48-4.29) |
| Gonococcal infection | United Arab Emirates | 23717.8 (16444-33378.4) | 1109.5 (798.7-1547.5) | 91628.8 (66773.3-122062.1) | 1047.4 (746.8-1488.5) | 0.15 (-4.02-4.5) |
| Gonococcal infection | United Kingdom | 81054 (63104.8-103549) | 142.5 (110.5-180.2) | 90458.7 (70820.5-115983.8) | 141.7 (108.4-180.4) | 0 (-3.29-3.4) |
| Gonococcal infection | United States | 926386.1 (671750-1235043.8) | 361.5 (262.5-485.8) | 1144646.1 (853177.6-1519914.6) | 367.2 (266-495.3) | -0.15 (-3.67-3.5) |
| Gonococcal infection | Uruguay | 13416.9 (10018.4-18062.9) | 435.4 (323.3-587.9) | 13500.3 (10155-18457.4) | 410.4 (306-558.3) | 0.16 (-3.27-3.71) |
| Gonococcal infection | Uzbekistan | 556774.9 (379621-821939) | 2436.3 (1711.6-3547.7) | 732692.2 (505609.2-1055780.4) | 2121.7 (1474.5-3090.3) | 0.53 (-3.58-4.81) |
| Gonococcal infection | Vanuatu | 2031 (1427.3-2920.5) | 1284.8 (914.8-1806.1) | 4172.5 (2923.2-5947.8) | 1236.2 (880.5-1727.1) | 0.92 (-3.27-5.3) |
| Gonococcal infection | Venezuela | 138433.6 (95712.2-202398.5) | 658.1 (471.7-940.5) | 157285.9 (115185.2-216608.2) | 637.8 (454-896.4) | 0.25 (-3.64-4.3) |
| Gonococcal infection | Vietnam | 780131 (532857.4-1105861.5) | 1057.2 (735.5-1464.8) | 1005254.2 (730741.4-1368283.7) | 1021 (737.5-1414.6) | 0.32 (-3.54-4.34) |
| Gonococcal infection | Yemen | 138866 (96830.3-199919.7) | 1110.4 (797.6-1579.1) | 389545.5 (270712.2-564342.3) | 1074.5 (768.7-1525.5) | 0.51 (-3.57-4.77) |
| Gonococcal infection | Zambia | 112763.3 (77860.8-164542.8) | 1328.8 (949.5-1849.9) | 281672.6 (193490.5-408221.7) | 1297.5 (933.4-1832.3) | -0.88 (-4.51-2.9) |
| Gonococcal infection | Zimbabwe | 230653.1 (158285.7-337342.3) | 2149.4 (1537.8-3008.5) | 343878 (241741.4-502143.2) | 2038 (1445.4-2899.8) | -1.3 (-4.89-2.44) |
| HIV/AIDS | Afghanistan | 55.9 (24.1-131.4) | 0.7 (0.3-1.8) | 432.5 (179.4-724.6) | 1.6 (0.6-2.6) | 0.27 (-3.89-4.6) |
| HIV/AIDS | Albania | 4.3 (3.4-5.9) | 0.1 (0.1-0.2) | 2.2 (1.5-3.4) | 0.1 (0.1-0.1) | -0.11 (-4.53-4.51) |
| HIV/AIDS | Algeria | 88.5 (79.4-99.7) | 0.4 (0.3-0.4) | 1682.3 (1530.2-1896.3) | 3.7 (3.3-4.2) | 0.49 (-3.58-4.74) |
| HIV/AIDS | American Samoa | 0.4 (0.2-0.8) | 0.8 (0.5-1.7) | 9.3 (5.6-12.3) | 19.9 (12.2-26.4) | 0.96 (-3.19-5.29) |
| HIV/AIDS | Andorra | 1.1 (1-1.2) | 1.7 (1.5-1.9) | 1.3 (1.1-1.4) | 1.4 (1.3-1.5) | -0.13 (-3.69-3.57) |
| HIV/AIDS | Angola | 2552.1 (479.9-6419.4) | 27.6 (5.2-69.5) | 59213.9 (27439.7-110248) | 208.2 (94.9-391.1) | 0.43 (-3.19-4.19) |
| HIV/AIDS | Antigua and Barbuda | 14.4 (9.5-32) | 24.5 (16.4-54.3) | 11 (7.9-15.7) | 12.1 (8.4-17.8) | -0.21 (-4.12-3.85) |
| HIV/AIDS | Argentina | 6164.2 (5689.4-6887.4) | 19.3 (17.8-21.5) | 7175 (6515.4-7832.6) | 14.8 (13.5-16.2) | -0.06 (-3.49-3.5) |
| HIV/AIDS | Armenia | 6.7 (0-33.3) | 0.2 (0-1) | 307.7 (235.5-418.9) | 9 (6.8-12.3) | 0.92 (-3.39-5.42) |
| HIV/AIDS | Australia | 1169.3 (969.8-1367.3) | 6.5 (5.5-7.6) | 1653.5 (977.7-2441.3) | 6.4 (3.7-9.6) | -0.13 (-3.7-3.57) |
| HIV/AIDS | Austria | 1348.4 (901.7-1867.4) | 15.7 (10.6-21.7) | 284.6 (152.5-451.7) | 3.4 (1.8-5.5) | -0.37 (-3.76-3.13) |
| HIV/AIDS | Azerbaijan | 55.4 (31-147.1) | 0.7 (0.4-2) | 260.1 (147.3-434.5) | 2.1 (1.2-3.5) | 0 (-4.41-4.62) |
| HIV/AIDS | Bahrain | 12.9 (10.2-17.2) | 2.3 (1.8-3) | 17.7 (14.4-23.5) | 1 (0.8-1.3) | -0.25 (-4.4-4.07) |
| HIV/AIDS | Bangladesh | 10.7 (9.3-12.4) | 0 (0-0) | 1565 (1245-1907.7) | 0.9 (0.7-1.1) | 0.02 (-4.03-4.25) |
| HIV/AIDS | Barbados | 150.5 (124.2-183.2) | 54.7 (45.8-66) | 157.2 (117.7-210.1) | 52.4 (40-71.1) | -0.19 (-4.02-3.8) |
| HIV/AIDS | Belarus | 379.6 (0-1231.2) | 3.5 (0-11.1) | 4298.3 (3104.9-5921.7) | 44 (31.2-63.7) | 0.97 (-3.01-5.1) |
| HIV/AIDS | Belgium | 821.8 (525-1218.1) | 7.6 (4.8-11.1) | 948 (411.6-1499.4) | 8.1 (3.9-12.2) | -0.14 (-3.44-3.28) |
| HIV/AIDS | Belize | 45.3 (35.2-58.4) | 30.6 (24.4-39) | 159.8 (109.9-254.1) | 33.7 (23.9-53.2) | -0.21 (-3.99-3.72) |
| HIV/AIDS | Benin | 1706.1 (979.3-2542.1) | 44.4 (25.7-66.3) | 2994.9 (1479.4-5725.5) | 26.2 (12.8-50.6) | -0.5 (-4.08-3.21) |
| HIV/AIDS | Bermuda | 22.5 (14.7-51.6) | 32.2 (21-73.6) | 4.5 (3.8-5.8) | 7.9 (6-11.5) | -0.48 (-4.39-3.6) |
| HIV/AIDS | Bhutan | 15.6 (9.6-23.7) | 2.7 (1.7-4.1) | 67 (31.2-113.2) | 7.8 (3.6-13.1) | 0.16 (-3.49-3.96) |
| HIV/AIDS | Bolivia | 184.3 (44.6-504.7) | 3.3 (0.8-8.9) | 1115.5 (688.3-1638.5) | 9.1 (5.6-13.3) | -0.33 (-4.02-3.51) |
| HIV/AIDS | Bosnia and Herzegovina | 4.9 (4.6-5.4) | 0.1 (0.1-0.1) | 10.5 (5.6-17.5) | 0.3 (0.1-0.5) | 0.06 (-4.35-4.67) |
| HIV/AIDS | Botswana | 16686.7 (14380.8-20174.3) | 1444.3 (1211.9-1783.7) | 6017.2 (3110.1-10838.9) | 226 (115.7-404.8) | -1.25 (-4.85-2.49) |
| HIV/AIDS | Brazil | 24084.9 (20595.4-28724.4) | 15.5 (13.5-18.4) | 52609.2 (34073.4-75206.3) | 22.1 (14.1-31.8) | 0.06 (-3.86-4.14) |
| HIV/AIDS | Brunei | 4.4 (2.7-6.5) | 1.8 (1.1-2.6) | 26.8 (14.3-44.9) | 5 (2.7-8.3) | 0.32 (-3.39-4.18) |
| HIV/AIDS | Bulgaria | 97.9 (0-180.6) | 1.1 (0-2) | 343.7 (198.6-485.3) | 5.7 (3.6-7.5) | 0.4 (-3.8-4.78) |
| HIV/AIDS | Burkina Faso | 30815.7 (21206.9-42412.5) | 359.5 (239.8-509.8) | 1833.9 (698-4076.5) | 8.8 (3.4-18.3) | -1.47 (-5.19-2.41) |
| HIV/AIDS | Burundi | 35082.7 (11482.6-59944.6) | 716.4 (238.8-1214.2) | 752.3 (403.7-1442.1) | 6.1 (3.2-11.6) | -1.89 (-5.64-2.01) |
| HIV/AIDS | Cambodia | 53.5 (48.1-59.5) | 0.6 (0.5-0.7) | 1184.5 (824.1-1667.3) | 6.6 (4.6-9.3) | -0.55 (-4.4-3.45) |
| HIV/AIDS | Cameroon | 18092.8 (11747.6-26391.8) | 202.4 (132.4-294.1) | 29518.9 (17535.6-50269.2) | 102.9 (60.7-175.5) | -0.82 (-4.42-2.91) |
| HIV/AIDS | Canada | 2918.2 (1590.5-4123.8) | 9.7 (5.4-13.8) | 3632.5 (1743.5-5843.6) | 10.7 (5.4-17) | 0.1 (-3.42-3.74) |
| HIV/AIDS | Cape Verde | 245.3 (71.9-477.6) | 84.7 (25.3-166.4) | 90.2 (19.5-389.7) | 14.7 (3.1-63.3) | -0.72 (-4.4-3.11) |
| HIV/AIDS | Central African Republic | 35761.9 (26215.8-49748.8) | 1475.2 (1072.4-2093) | 6070.8 (1927.7-14632.9) | 123.2 (39.6-290) | -0.86 (-4.39-2.79) |
| HIV/AIDS | Chad | 6465.5 (2540.3-12283.6) | 129.6 (51.6-245.4) | 5357.6 (2224.6-10613.8) | 40.1 (16.5-78.2) | -0.59 (-4.2-3.15) |
| HIV/AIDS | Chile | 847 (593.4-1107.3) | 5.8 (4.1-7.5) | 4753.5 (2571.1-7650.7) | 23.8 (12.7-38.5) | 0.38 (-3.11-4.01) |
| HIV/AIDS | China | 7884.9 (4872.7-14791.2) | 0.7 (0.4-1.3) | 31708.7 (16964.8-48726.9) | 2.3 (1.3-3.8) | -0.08 (-4.2-4.23) |
| HIV/AIDS | Colombia | 2598.5 (1177.7-9175.6) | 8.1 (3.7-27.4) | 9551.1 (6073.4-13935.2) | 17.9 (11.4-26.1) | 0.24 (-3.66-4.29) |
| HIV/AIDS | Comoros | 1.2 (0.3-3) | 0.3 (0.1-0.8) | 2 (0.7-3.6) | 0.3 (0.1-0.5) | -0.17 (-4.66-4.53) |
| HIV/AIDS | Congo | 12911.4 (7546.9-19243) | 586.6 (330.9-898.4) | 6814 (2596.7-16039.2) | 127.2 (48.3-298.8) | -0.53 (-4.06-3.13) |
| HIV/AIDS | Cook Islands | 0.2 (0.1-0.3) | 0.8 (0.5-1.7) | 3.4 (2.1-4.5) | 19.8 (12.4-25.7) | 1.05 (-3.11-5.39) |
| HIV/AIDS | Costa Rica | 188.3 (160.6-219.9) | 6.2 (5.5-7.1) | 420.7 (332.1-549.5) | 8.2 (6.4-10.9) | 0.07 (-3.98-4.28) |
| HIV/AIDS | Cote d'Ivoire | 99933.1 (66356.7-140053.6) | 901.8 (593.9-1294.9) | 12765.8 (6478.4-23603.8) | 49.5 (24.6-92.5) | -1.18 (-4.71-2.49) |
| HIV/AIDS | Croatia | 88.7 (67.1-116.5) | 1.7 (1.2-2.2) | 77.7 (32-115.1) | 1.8 (0.8-2.8) | 0.08 (-4.16-4.5) |
| HIV/AIDS | Cuba | 194.7 (157.9-250.1) | 1.5 (1.3-1.9) | 1974.9 (1053.4-3322.2) | 15.7 (8.7-24.8) | 0.92 (-3.19-5.22) |
| HIV/AIDS | Cyprus | 4 (2.3-5.9) | 0.5 (0.3-0.7) | 11.9 (8.1-18.5) | 1 (0.6-1.6) | 0.04 (-3.63-3.85) |
| HIV/AIDS | Czech Republic | 31.5 (22.9-46.2) | 0.3 (0.2-0.4) | 185 (76.3-301.4) | 2.1 (0.9-3.4) | 0.39 (-3.87-4.83) |
| HIV/AIDS | Democratic Republic of the Congo | 76628 (51332.8-104016.3) | 216.7 (141.8-295.7) | 5914.9 (3467.7-9853.8) | 7.2 (4.2-12.2) | -1.3 (-4.94-2.49) |
| HIV/AIDS | Denmark | 347.2 (329.2-366.8) | 6.3 (6-6.6) | 185.6 (173.4-198.8) | 3.1 (3-3.3) | -0.26 (-3.73-3.34) |
| HIV/AIDS | Djibouti | 43.8 (5.8-150.6) | 11.9 (1.6-41.1) | 1036.7 (360.7-2289.3) | 77.3 (26.6-171.3) | -0.19 (-3.94-3.7) |
| HIV/AIDS | Dominica | 13.7 (11-17.8) | 20.4 (16.7-26.1) | 7.1 (5.3-10.4) | 10.8 (7.8-16.4) | -0.21 (-4.16-3.9) |
| HIV/AIDS | Dominican Republic | 8129.4 (5156.3-11784.2) | 118.8 (74.7-173.5) | 1805.4 (577.7-4270.8) | 15.6 (5-36.7) | -0.71 (-4.46-3.19) |
| HIV/AIDS | Ecuador | 322.3 (178.2-794.1) | 3.2 (1.8-7.9) | 3803 (2931.6-5190.7) | 19.9 (15.7-26.6) | 0.54 (-3.22-4.44) |
| HIV/AIDS | Egypt | 108.1 (77.1-159.8) | 0.2 (0.1-0.3) | 810.6 (401.1-1420) | 0.7 (0.4-1.3) | 0 (-4.33-4.53) |
| HIV/AIDS | El Salvador | 440.5 (306.5-622.8) | 9.2 (6.6-12.7) | 667.1 (473.5-969.3) | 9.9 (7.1-14.7) | -0.23 (-4.16-3.86) |
| HIV/AIDS | Equatorial Guinea | 287.2 (83.8-710.6) | 78.8 (23.4-195.6) | 8286.8 (1649.8-22574.6) | 571.8 (114.6-1545.9) | 0.57 (-2.97-4.24) |
| HIV/AIDS | Eritrea | 5453.4 (1633.4-10442) | 184.1 (53.9-347.7) | 880.6 (439.3-1591.1) | 12.6 (6.2-23.2) | -1.3 (-5.1-2.65) |
| HIV/AIDS | Estonia | 11.3 (6.3-19.3) | 0.7 (0.4-1.3) | 217.3 (147.4-351.6) | 20.2 (13.6-33.8) | 0.65 (-3.26-4.73) |
| HIV/AIDS | Ethiopia | 74704.7 (30473.4-128374) | 175.4 (71.3-299.4) | 22256.8 (12984.5-45759.5) | 22.4 (12.9-45.8) | -1.38 (-5.11-2.5) |
| HIV/AIDS | Federated States of Micronesia | 0.8 (0.5-1.5) | 0.9 (0.5-1.7) | 21.9 (13.5-28.2) | 21.1 (12.9-27.1) | 0.99 (-3.14-5.3) |
| HIV/AIDS | Fiji | 10.4 (6.4-25) | 1.4 (0.9-3.5) | 45 (26.3-78.9) | 4.8 (2.8-8.4) | 0.08 (-4.15-4.5) |
| HIV/AIDS | Finland | 148.2 (103.6-194.4) | 2.6 (1.8-3.5) | 105.8 (52.3-168.2) | 1.9 (0.9-3.1) | -0.17 (-3.68-3.47) |
| HIV/AIDS | France | 5984.7 (4946.4-7633) | 9.9 (8.2-12.6) | 9038.9 (8119.2-10083.3) | 14.1 (12.8-15.3) | 0.07 (-3.21-3.47) |
| HIV/AIDS | Gabon | 1719.1 (865.8-3070) | 192 (96.6-345.1) | 2140.4 (764.2-5643.2) | 121 (42.6-316.5) | -0.52 (-4.04-3.14) |
| HIV/AIDS | Georgia | 80.1 (19.1-224.4) | 1.4 (0.3-3.9) | 284.9 (175.6-512.7) | 7.3 (4.4-13.5) | 0.67 (-3.52-5.04) |
| HIV/AIDS | Germany | 6055.5 (4912-7313.7) | 7.1 (5.8-8.6) | 2416 (1556.6-3637.8) | 3 (1.9-4.4) | -0.12 (-3.64-3.52) |
| HIV/AIDS | Ghana | 20858.3 (15281.5-26745.8) | 153.8 (113.2-198.6) | 23678.6 (13369.4-40192.6) | 71.9 (40.6-121.7) | -0.48 (-4.16-3.34) |
| HIV/AIDS | Greece | 463.5 (314.4-650.8) | 4.5 (3.1-6.3) | 492.5 (332.1-857) | 6.4 (4.3-10.8) | 0.58 (-2.98-4.27) |
| HIV/AIDS | Greenland | 12.7 (8.6-17.3) | 19.3 (13.1-26.1) | 9.8 (1.5-22) | 16.4 (2.6-36.6) | 0.04 (-3.4-3.61) |
| HIV/AIDS | Grenada | 12.6 (8.4-27.5) | 17.1 (11.5-37.1) | 4.8 (3.8-6.9) | 4.5 (3.5-6.5) | -0.4 (-4.39-3.75) |
| HIV/AIDS | Guam | 4.6 (3.4-11.4) | 3.2 (2.3-7.8) | 7.7 (4.7-12.7) | 5 (3.1-8.4) | 0.1 (-4.06-4.44) |
| HIV/AIDS | Guatemala | 729.6 (541.4-1026.3) | 11.1 (8.6-15) | 1052.6 (684.1-1760.2) | 6.1 (4.1-10) | -0.51 (-4.52-3.67) |
| HIV/AIDS | Guinea | 3477.7 (1666.3-5990.9) | 67.4 (32.4-114.2) | 6063.4 (2994-10444.1) | 53.4 (26.5-93.6) | -0.32 (-3.93-3.42) |
| HIV/AIDS | Guinea-Bissau | 495.9 (96.4-1003.8) | 58.6 (11.5-121.8) | 4831.9 (301.8-12525) | 238.5 (16.9-630) | 0.28 (-3.3-3.99) |
| HIV/AIDS | Guyana | 319.7 (241.7-428.7) | 42.4 (33-55) | 309.6 (236.7-425.8) | 37.9 (29.5-55.5) | -0.3 (-4.05-3.59) |
| HIV/AIDS | Haiti | 23858.7 (16037.2-34882.7) | 388.8 (248-577.5) | 11796.3 (4529.1-22582.7) | 88.6 (34.5-168) | -0.6 (-4.18-3.11) |
| HIV/AIDS | Honduras | 268.2 (223.1-331.5) | 6.3 (5.5-7.5) | 247.8 (173.3-370.4) | 2.3 (1.6-3.3) | -0.35 (-4.55-4.04) |
| HIV/AIDS | Hungary | 107.3 (78.1-131) | 1 (0.8-1.2) | 358.6 (216.1-500.2) | 3 (1.9-4) | 0.36 (-3.86-4.77) |
| HIV/AIDS | Iceland | 9.9 (7.6-12.4) | 3.7 (2.8-4.6) | 31.2 (15.9-52.3) | 9.1 (4.5-15.2) | 0.38 (-3.03-3.91) |
| HIV/AIDS | India | 20034.8 (11647.7-31919.9) | 2.5 (1.4-4) | 71016.4 (50108.4-97509.6) | 4.8 (3.4-6.6) | -0.44 (-4.05-3.31) |
| HIV/AIDS | Indonesia | 1158.6 (342.1-1775.7) | 0.6 (0.2-1) | 10190.1 (7501.8-15457.9) | 3.4 (2.5-5.2) | 0.56 (-3.67-4.97) |
| HIV/AIDS | Iran | 100.2 (42.1-307.6) | 0.2 (0.1-0.7) | 2231.8 (1682.2-2937.8) | 2.3 (1.8-3) | 0.47 (-3.83-4.96) |
| HIV/AIDS | Iraq | 23.5 (12.1-53.3) | 0.1 (0.1-0.3) | 273.6 (109.1-538.1) | 0.6 (0.2-1.3) | -0.11 (-4.46-4.44) |
| HIV/AIDS | Ireland | 362 (242.2-489.1) | 10.4 (6.9-14) | 398.5 (178.2-672.6) | 9.1 (3.9-15.1) | -0.07 (-3.39-3.35) |
| HIV/AIDS | Israel | 216.1 (128.6-338.2) | 4.5 (2.7-7) | 648.8 (351.4-916.1) | 7 (3.8-10) | 0.11 (-3.29-3.62) |
| HIV/AIDS | Italy | 8274.9 (5684.5-13775.1) | 14.5 (10.3-23.3) | 2867.7 (1966.5-3900.5) | 5.2 (3.4-7.1) | -0.26 (-3.6-3.19) |
| HIV/AIDS | Jamaica | 824.4 (462-1942.2) | 36.8 (21.1-86.5) | 1056.2 (790.7-1466) | 33.6 (25.3-46) | -0.1 (-3.89-3.85) |
| HIV/AIDS | Japan | 644.6 (341.1-1051.6) | 0.5 (0.2-0.8) | 2892.2 (1271.2-4533.7) | 1.8 (0.9-2.7) | 0.26 (-3.62-4.31) |
| HIV/AIDS | Jordan | 4.4 (3-6.9) | 0.1 (0.1-0.2) | 17.9 (13.2-24.4) | 0.1 (0.1-0.2) | -0.02 (-4.11-4.25) |
| HIV/AIDS | Kazakhstan | 1822.5 (1294.8-2431.5) | 10.3 (7.4-13.7) | 8569.3 (5174.6-11877.6) | 41.3 (25.4-57.3) | 0.39 (-3.64-4.58) |
| HIV/AIDS | Kenya | 245161.1 (207662.4-281252.7) | 1277.8 (1071.9-1480.8) | 35509.9 (26344.8-45880.4) | 72.9 (54.2-93.6) | -1.43 (-5.05-2.32) |
| HIV/AIDS | Kiribati | 1 (0.9-1.3) | 1.5 (1.2-1.9) | 0.9 (0.4-2.4) | 0.7 (0.3-1.9) | -0.14 (-4.48-4.4) |
| HIV/AIDS | Kuwait | 17.7 (12.4-23.9) | 1.3 (0.8-1.8) | 19.5 (13.3-29.4) | 0.4 (0.3-0.7) | -0.27 (-4.48-4.13) |
| HIV/AIDS | Kyrgyzstan | 86.5 (56.8-122.5) | 2.1 (1.4-2.9) | 2703.9 (1586.8-4113.9) | 39.8 (23.4-60.1) | 0.86 (-3.29-5.19) |
| HIV/AIDS | Laos | 3.2 (2.5-4.9) | 0.1 (0.1-0.1) | 507.4 (377.4-668) | 6.5 (4.8-8.6) | 0.67 (-3.38-4.9) |
| HIV/AIDS | Latvia | 278.3 (209.2-359.3) | 10.2 (7.6-13.3) | 559.8 (330.2-676.4) | 31.4 (19.8-38.1) | 0.27 (-3.61-4.31) |
| HIV/AIDS | Lebanon | 24 (20.8-28) | 0.8 (0.7-1) | 209.4 (187.4-235.7) | 3.5 (3.1-3.9) | 0.32 (-3.65-4.46) |
| HIV/AIDS | Lesotho | 8931.7 (6865.8-11557.8) | 691.8 (535.5-890.9) | 15963.3 (10396.2-23240) | 818.4 (534.7-1183.3) | -0.33 (-3.99-3.46) |
| HIV/AIDS | Liberia | 1782.8 (478.5-4269.7) | 82.3 (22.4-191.1) | 1999.7 (929.1-4122.6) | 39.4 (18.2-80.1) | -0.48 (-4.09-3.26) |
| HIV/AIDS | Libya | 24.2 (3.7-96.6) | 0.7 (0.1-2.7) | 134.6 (3.7-744) | 1.8 (0.1-10) | 0.15 (-3.89-4.36) |
| HIV/AIDS | Lithuania | 75.2 (61.4-94) | 2 (1.6-2.6) | 305.8 (179.8-498) | 11.2 (6.4-18.9) | 0.45 (-3.61-4.68) |
| HIV/AIDS | Luxembourg | 70.6 (54.9-87.5) | 16.9 (12.9-21.1) | 51 (26.6-83.4) | 7.9 (4-13.2) | -0.24 (-3.47-3.11) |
| HIV/AIDS | Macedonia | 1 (0.8-1.2) | 0 (0-0.1) | 5.9 (3.2-9.9) | 0.2 (0.1-0.4) | 0.06 (-4.35-4.68) |
| HIV/AIDS | Madagascar | 53.4 (14.3-117) | 0.5 (0.1-1.2) | 6070.4 (2299.7-12943.1) | 22.6 (8.4-48.9) | 0.59 (-3.5-4.85) |
| HIV/AIDS | Malawi | 112012.6 (94224.5-129692.4) | 1262.5 (1063.8-1481.9) | 29119.4 (18556.4-43934.1) | 167.3 (106-253.6) | -0.93 (-4.53-2.81) |
| HIV/AIDS | Malaysia | 2238.6 (1500.8-3232.3) | 13.1 (8.9-18.7) | 4824.8 (3420.4-6688.5) | 13.6 (9.7-18.7) | -0.14 (-4.07-3.94) |
| HIV/AIDS | Maldives | 0.8 (0.6-1.2) | 0.4 (0.3-0.6) | 3 (2.5-4) | 0.5 (0.4-0.7) | 0.08 (-4.31-4.67) |
| HIV/AIDS | Mali | 6233.4 (1854.8-13079.9) | 84.5 (25.8-173.9) | 7725.1 (3984.7-13670.7) | 32.3 (15.6-60.5) | -0.76 (-4.46-3.08) |
| HIV/AIDS | Malta | 20.1 (18-22.7) | 5.4 (4.8-6.3) | 94.9 (48.9-155.5) | 23.5 (11.5-39.1) | 0.67 (-2.67-4.13) |
| HIV/AIDS | Marshall Islands | 0.3 (0.2-0.6) | 0.9 (0.5-1.7) | 12 (7.4-15.6) | 20.5 (12.7-26.7) | 0.87 (-3.11-5.02) |
| HIV/AIDS | Mauritania | 20.3 (5.9-57.8) | 0.7 (0.3-1.7) | 30.5 (2.7-158.2) | 0.5 (0.1-2.5) | -0.14 (-4.38-4.29) |
| HIV/AIDS | Mauritius | 20.1 (17.3-29.6) | 1.7 (1.5-2.4) | 209.1 (163.6-276.7) | 15.6 (12.4-20.7) | 0.69 (-3.22-4.77) |
| HIV/AIDS | Mexico | 15606 (11490-21555.3) | 19.3 (14.6-26.2) | 17175.7 (13991.9-21707.8) | 12.6 (10.2-15.9) | 0.09 (-3.94-4.29) |
| HIV/AIDS | Moldova | 282.6 (107.8-1290.3) | 6 (2.3-27.3) | 652 (449.6-919.1) | 16.6 (11.2-23.8) | 0.22 (-3.64-4.23) |
| HIV/AIDS | Monaco | 0.5 (0.4-0.6) | 1.6 (1.4-2) | 0.6 (0.5-0.7) | 1.7 (1.6-1.8) | -0.03 (-3.59-3.66) |
| HIV/AIDS | Mongolia | 0.2 (0-0) | 0 (0-0) | 33.4 (9.8-78.2) | 1 (0.3-2.4) | 0.37 (-4.08-5.03) |
| HIV/AIDS | Montenegro | 2.7 (1.9-3.5) | 0.4 (0.3-0.5) | 4.4 (3.4-5.9) | 0.8 (0.5-1.1) | 0 (-4.31-4.51) |
| HIV/AIDS | Morocco | 585.1 (393.3-859.7) | 2.4 (1.6-3.5) | 675.9 (487.4-909.5) | 1.7 (1.3-2.4) | -0.27 (-4.32-3.94) |
| HIV/AIDS | Mozambique | 25321.9 (13752.5-42260.9) | 210.9 (112.7-354) | 111812.2 (48233.2-234074.8) | 402.4 (165.1-835.1) | -0.21 (-3.9-3.63) |
| HIV/AIDS | Myanmar | 7197.1 (6137.9-8334.5) | 18.3 (15.6-21.3) | 8814.6 (6902.3-11207.1) | 15.1 (11.8-19.2) | -0.3 (-4.05-3.6) |
| HIV/AIDS | Namibia | 4867.1 (3704.4-6348.7) | 397.5 (302.4-521.5) | 4444 (3154.6-6019.5) | 180.5 (126-244) | -0.75 (-4.39-3.03) |
| HIV/AIDS | Nauru | 0.1 (0-0.2) | 0.8 (0.5-1.7) | 2.2 (1.4-2.9) | 20.1 (12.7-26.1) | 1.02 (-3.14-5.35) |
| HIV/AIDS | Nepal | 0 (0-0) | 0 (0-0) | 922.4 (667.7-1253.6) | 2.8 (2.1-3.9) | 0.14 (-3.56-3.98) |
| HIV/AIDS | Netherlands | 537.1 (414.8-662.5) | 3.3 (2.5-4.1) | 352.1 (179.1-583.4) | 2.2 (1.1-3.6) | -0.06 (-3.48-3.47) |
| HIV/AIDS | New Zealand | 86.7 (68-107.8) | 2.4 (1.9-3) | 113.2 (58-183.9) | 2.2 (1.1-3.5) | 0.13 (-3.66-4.07) |
| HIV/AIDS | Nicaragua | 53.2 (36-78.5) | 1.5 (1-2.1) | 877.3 (432.2-1645.2) | 12.2 (6.1-22.4) | 0.45 (-3.69-4.78) |
| HIV/AIDS | Niger | 3427.4 (1209.9-6960.1) | 53.1 (19.1-107.8) | 835.2 (291.9-2085.9) | 4.5 (1.5-11.5) | -1.21 (-4.97-2.7) |
| HIV/AIDS | Nigeria | 94445.8 (71596.7-124059.5) | 119.3 (90.9-157.3) | 140777 (114816.1-175975.1) | 71.6 (58.2-90.1) | -0.41 (-4.07-3.4) |
| HIV/AIDS | Niue | 0 (0-0) | 0.8 (0.5-1.7) | 0.3 (0.2-0.4) | 19.5 (12.1-25.4) | 0.96 (-3.19-5.28) |
| HIV/AIDS | North Korea | 35.7 (12.2-71) | 0.2 (0.1-0.3) | 1445.5 (277.3-3153.7) | 5.1 (1-11.2) | 0.61 (-3.52-4.92) |
| HIV/AIDS | Northern Mariana Islands | 0.7 (0.4-1.7) | 1.3 (0.8-3.2) | 1.5 (0.9-2.5) | 3.5 (2.1-5.8) | 0.11 (-4.12-4.52) |
| HIV/AIDS | Norway | 436.8 (361.4-558.5) | 9.7 (8-12.3) | 293 (153.4-456.9) | 5.9 (3-9.3) | -0.12 (-3.57-3.45) |
| HIV/AIDS | Oman | 13.5 (9.6-19.4) | 0.7 (0.5-1) | 338.9 (129.3-697.5) | 5.7 (2.3-10.9) | 0.21 (-3.9-4.5) |
| HIV/AIDS | Pakistan | 4.4 (0.2-21.2) | 0 (0-0) | 24818.4 (1747-88072.4) | 10.5 (0.7-37.6) | 1.1 (-2.86-5.23) |
| HIV/AIDS | Palau | 0.1 (0.1-0.3) | 0.8 (0.5-1.7) | 3.5 (2.1-4.6) | 18.5 (11.4-24.2) | 0.87 (-3.28-5.19) |
| HIV/AIDS | Palestine | 2.2 (1.4-3.9) | 0.1 (0.1-0.2) | 34 (20-54.4) | 0.7 (0.4-1) | 0.16 (-4.12-4.63) |
| HIV/AIDS | Panama | 779.3 (513.8-1125.2) | 32.7 (22.3-46) | 2062.7 (1553.4-2876.6) | 46 (34.8-64.2) | 0.21 (-3.6-4.17) |
| HIV/AIDS | Papua New Guinea | 41.3 (17.2-82) | 1.1 (0.5-2.3) | 3542.8 (1466.3-7017.6) | 35.2 (14.2-70) | 0.53 (-3.47-4.7) |
| HIV/AIDS | Paraguay | 103.4 (29.5-361.3) | 2.8 (0.8-9.9) | 797.2 (468-1317.7) | 10.3 (6.1-16.9) | 0.46 (-3.59-4.68) |
| HIV/AIDS | Peru | 1860.8 (1010-5892.5) | 8.6 (4.7-27.9) | 4689 (2971.1-6856.9) | 12 (7.6-17.4) | 0.01 (-3.81-3.99) |
| HIV/AIDS | Philippines | 76.3 (39.6-141.5) | 0.1 (0.1-0.2) | 47279.5 (26347.6-76796.8) | 39.6 (22.2-63.2) | 1.16 (-2.86-5.36) |
| HIV/AIDS | Poland | 270.5 (138.8-457.5) | 0.7 (0.4-1.2) | 1028.9 (501.7-1862.8) | 2.7 (1.4-4.8) | 0.18 (-4.01-4.55) |
| HIV/AIDS | Portugal | 2777.4 (2623.1-2943.1) | 26.3 (25.1-27.5) | 732.1 (636-856.2) | 6.9 (6.1-7.9) | -0.73 (-3.96-2.61) |
| HIV/AIDS | Puerto Rico | 5883.5 (5217.2-6866.7) | 161.7 (142.9-188.7) | 461.2 (397.8-542.1) | 14.5 (12.5-18) | -0.9 (-4.73-3.08) |
| HIV/AIDS | Qatar | 5.5 (4.6-7.4) | 1 (0.8-1.3) | 8.4 (6.5-10.2) | 0.2 (0.2-0.3) | -0.23 (-4.5-4.22) |
| HIV/AIDS | Romania | 202.2 (135.4-279.4) | 0.9 (0.6-1.2) | 709.3 (403.2-1113) | 5 (2.8-7.9) | 0.28 (-3.82-4.56) |
| HIV/AIDS | Russian Federation | 6335.9 (4126.8-7711.2) | 4 (2.6-4.9) | 98872.2 (78813.8-126235.4) | 70.2 (55.9-92.3) | 1.19 (-2.71-5.26) |
| HIV/AIDS | Rwanda | 21871.7 (6717.8-44245.2) | 349.8 (108.8-684.3) | 7023.5 (3623.6-12903.8) | 55.7 (28.4-102.4) | -0.97 (-4.64-2.84) |
| HIV/AIDS | Saint Kitts and Nevis | 12.1 (7.8-27.3) | 30.5 (19.5-68.3) | 20 (16-24.6) | 31.4 (25.6-38.3) | -0.03 (-3.79-3.88) |
| HIV/AIDS | Saint Lucia | 112.7 (90.3-144.2) | 93.4 (77.3-117.2) | 65.1 (50.8-90.5) | 36.2 (26.9-52.9) | -0.41 (-4.16-3.49) |
| HIV/AIDS | Saint Vincent and the Grenadines | 46.5 (33.8-64.4) | 46.6 (34.9-63.1) | 21.9 (17.2-30.7) | 19.7 (15.1-28.7) | -0.51 (-4.33-3.47) |
| HIV/AIDS | Samoa | 1.3 (0.8-2.6) | 0.9 (0.5-1.7) | 42.9 (27-55.1) | 21 (13.1-27.1) | 0.97 (-3.28-5.41) |
| HIV/AIDS | San Marino | 0.5 (0.4-0.6) | 1.8 (1.6-2.1) | 0.5 (0.5-0.6) | 1.6 (1.5-1.8) | -0.07 (-3.62-3.61) |
| HIV/AIDS | Sao Tome and Principe | 2.5 (1-9.2) | 2.4 (0.9-10.4) | 1.7 (0.9-2.8) | 0.9 (0.4-1.5) | -0.49 (-4.64-3.84) |
| HIV/AIDS | Saudi Arabia | 217.3 (115.6-478.2) | 1.5 (0.8-3.3) | 1163.4 (428.4-2776.2) | 2.7 (1-6.4) | -0.03 (-4.22-4.34) |
| HIV/AIDS | Senegal | 2429.4 (1348.3-3903.7) | 37.6 (21.2-59.4) | 2189.4 (878.1-4368.9) | 14.9 (6.1-29.1) | -0.7 (-4.3-3.04) |
| HIV/AIDS | Serbia | 36.2 (0-53.6) | 0.4 (0-0.6) | 33.5 (19.5-62.3) | 0.4 (0.2-0.8) | 0 (-4.35-4.55) |
| HIV/AIDS | Seychelles | 7.6 (5-11.2) | 11.1 (7.5-16.3) | 4.2 (3.6-5.3) | 3.8 (3.2-5.1) | -0.26 (-4.28-3.94) |
| HIV/AIDS | Sierra Leone | 1738.2 (428.7-4056.6) | 45.8 (11.6-109.3) | 3436.2 (1550-6798.9) | 42 (18.7-83.9) | -0.21 (-3.84-3.55) |
| HIV/AIDS | Singapore | 840.9 (560.8-1226.8) | 23.4 (15.7-34.1) | 547.2 (342.4-856.5) | 8.8 (5.4-14.1) | -0.46 (-3.96-3.18) |
| HIV/AIDS | Slovakia | 4.6 (3.8-5.5) | 0.1 (0.1-0.1) | 15.5 (8-27) | 0.3 (0.1-0.5) | 0.05 (-4.36-4.66) |
| HIV/AIDS | Slovenia | 36.8 (0-63.6) | 1.7 (0-2.9) | 28.7 (17.5-50.9) | 1.4 (0.8-2.5) | -0.2 (-4.32-4.1) |
| HIV/AIDS | Solomon Islands | 2.7 (1.7-5.1) | 0.9 (0.6-1.7) | 143.1 (89.3-184.2) | 21.2 (13.3-27.4) | 0.94 (-3.15-5.19) |
| HIV/AIDS | Somalia | 991.2 (170.1-3195.2) | 15.1 (2.6-49.4) | 3772.6 (1297.4-8891.8) | 17.9 (5.7-42.9) | -0.55 (-4.47-3.53) |
| HIV/AIDS | South Africa | 54755.4 (40966.9-71097.5) | 149.9 (112.2-193.1) | 233514.4 (168238.2-305931.5) | 373.3 (270.4-488.7) | -0.52 (-4.33-3.44) |
| HIV/AIDS | South Korea | 186.2 (0-447.5) | 0.4 (0-1) | 544.5 (87.3-902.1) | 1.1 (0.2-1.8) | 0.02 (-3.88-4.08) |
| HIV/AIDS | South Sudan | 3118.9 (412-10234) | 61.4 (8.3-200.3) | 7616.3 (1343.2-21509.9) | 99.1 (16.9-284.6) | -0.09 (-3.83-3.8) |
| HIV/AIDS | Spain | 17168.3 (14098.5-21127.8) | 42.2 (34.9-51.5) | 3472.4 (2398.1-4602.7) | 7.6 (5.2-10.1) | -0.6 (-3.89-2.82) |
| HIV/AIDS | Sri Lanka | 111.6 (11.9-563) | 0.6 (0.1-3.2) | 247.8 (124.9-479.3) | 1.1 (0.5-2.2) | 0.01 (-4.41-4.63) |
| HIV/AIDS | Sudan | 2087.4 (190.3-7755.1) | 11.9 (1.1-46.2) | 16455.5 (1663.3-59649) | 39.5 (3.9-145.7) | 0.13 (-3.67-4.08) |
| HIV/AIDS | Suriname | 111.7 (74.2-177.8) | 29.8 (20.2-46.7) | 245.9 (168.6-363.4) | 42.6 (28.8-64.1) | -0.09 (-3.89-3.85) |
| HIV/AIDS | Swaziland | 541.5 (9.5-2856.1) | 78.7 (1.4-403.5) | 4258.4 (2300.5-7150.7) | 349.6 (185.2-592) | -0.24 (-3.91-3.57) |
| HIV/AIDS | Sweden | 1183.5 (830.7-1762.9) | 13.6 (9.4-20.2) | 530.2 (269.9-783.8) | 5.6 (3-8.2) | -0.15 (-3.64-3.47) |
| HIV/AIDS | Switzerland | 1961 (1053-2826) | 26.2 (14.4-38) | 386.5 (170.2-625.7) | 4.2 (1.9-6.9) | -0.79 (-4.08-2.62) |
| HIV/AIDS | Syria | 9.9 (7.5-13.2) | 0.1 (0.1-0.1) | 47.8 (30.9-75) | 0.3 (0.2-0.5) | 0 (-4.28-4.47) |
| HIV/AIDS | Taiwan | 284.4 (182.2-481.3) | 1.3 (0.8-2.2) | 1298.7 (397.8-2377.1) | 5.7 (2-10.9) | 0.43 (-3.63-4.67) |
| HIV/AIDS | Tajikistan | 90.1 (60.7-131.2) | 1.9 (1.3-2.8) | 618.6 (308.9-892.2) | 6.3 (3-9.4) | 0.14 (-4.11-4.59) |
| HIV/AIDS | Tanzania | 184282.2 (143988.8-229520) | 797.7 (600.6-1016.8) | 32091.8 (13047.2-64934.6) | 57.5 (22.5-120.2) | -1.24 (-4.97-2.64) |
| HIV/AIDS | Thailand | 59841.3 (37383.5-86388.2) | 93.7 (59-135.3) | 14791.3 (9767.9-22997.1) | 23.9 (15.2-39.3) | -0.72 (-4.45-3.15) |
| HIV/AIDS | The Bahamas | 305.3 (258.9-359.2) | 111.5 (96.8-129.1) | 243.5 (162.1-373.3) | 59.5 (38.2-93.1) | -0.3 (-4.02-3.56) |
| HIV/AIDS | The Gambia | 311.9 (104.3-623.6) | 36.9 (12.2-73.3) | 2824.8 (1176.6-5682.8) | 133.3 (55.9-259.8) | 0.14 (-3.42-3.83) |
| HIV/AIDS | Timor-Leste | 196.5 (174.2-221.1) | 27.1 (23.8-30.6) | 248.1 (203.2-300.8) | 18.3 (14.8-22.3) | -0.2 (-4.01-3.76) |
| HIV/AIDS | Togo | 6276.4 (3616-9914.3) | 205.6 (117-323.9) | 2958.9 (1529.3-5118.5) | 38.1 (19.3-66.5) | -0.96 (-4.47-2.68) |
| HIV/AIDS | Tokelau | 0 (0-0) | 0.8 (0.5-1.7) | 0.2 (0.1-0.3) | 18.8 (11.5-24.6) | 0.84 (-3.31-5.17) |
| HIV/AIDS | Tonga | 0.8 (0.5-1.5) | 0.9 (0.5-1.7) | 24.2 (15.7-31.2) | 23.9 (15.5-30.9) | 1.02 (-3.16-5.37) |
| HIV/AIDS | Trinidad and Tobago | 689 (495-957.1) | 56 (41-76.9) | 1146.5 (937.8-1392.6) | 87.8 (72.2-112.3) | -0.02 (-3.72-3.83) |
| HIV/AIDS | Tunisia | 9.5 (1.1-22.3) | 0.1 (0-0.3) | 117 (57-188.9) | 0.9 (0.5-1.5) | 0.28 (-3.98-4.72) |
| HIV/AIDS | Turkey | 33.8 (0-84.7) | 0.1 (0-0.2) | 442 (313.1-636.3) | 0.5 (0.4-0.8) | -0.04 (-4.32-4.44) |
| HIV/AIDS | Turkmenistan | 206.7 (129.1-756.1) | 5.5 (3.5-20.5) | 144.5 (107.4-220.2) | 2.6 (2-4) | -0.39 (-4.59-4) |
| HIV/AIDS | Tuvalu | 0.1 (0-0.2) | 0.8 (0.5-1.7) | 2.5 (1.6-3.2) | 20 (12.5-26.1) | 0.84 (-3.32-5.17) |
| HIV/AIDS | Uganda | 123333.4 (111447.9-137922) | 683.7 (613.6-774.7) | 59070.5 (28840.4-109588) | 153.5 (77.1-282.9) | -0.66 (-4.3-3.11) |
| HIV/AIDS | Ukraine | 4131.3 (3204.5-6453.3) | 7.8 (5.9-12.3) | 40655 (29616.7-53440) | 87.4 (63.7-120.4) | 0.33 (-3.48-4.29) |
| HIV/AIDS | United Arab Emirates | 11.4 (7.2-20.8) | 0.5 (0.3-0.9) | 211.3 (131.7-299.5) | 1.7 (1.1-2.4) | 0.15 (-4.02-4.5) |
| HIV/AIDS | United Kingdom | 2956.4 (2102.4-4367.5) | 5 (3.6-7.4) | 5874.4 (3833.7-8133.1) | 8.9 (5.8-12.5) | 0 (-3.29-3.4) |
| HIV/AIDS | United States | 60798.5 (37212.9-83034.1) | 22.2 (13.7-30.5) | 58484.1 (23163.6-93818.1) | 17.7 (7.4-28) | -0.15 (-3.67-3.5) |
| HIV/AIDS | Uruguay | 378 (159.1-1180.9) | 11.6 (4.9-36) | 942.6 (659.4-1264.6) | 28.5 (19.3-38.6) | 0.16 (-3.27-3.71) |
| HIV/AIDS | Uzbekistan | 542.6 (184.3-989.9) | 2.8 (1-5.2) | 8677.8 (2758.8-14742.8) | 23 (7.6-38.6) | 0.53 (-3.58-4.81) |
| HIV/AIDS | Vanuatu | 1.2 (0.8-2.3) | 0.8 (0.5-1.7) | 64.6 (39.9-83.3) | 20.9 (12.8-27) | 0.92 (-3.27-5.3) |
| HIV/AIDS | Venezuela | 1700.7 (1296.7-2207.6) | 9.5 (7.4-12.3) | 6086 (4775.8-8100.1) | 22.9 (17.5-29.1) | 0.25 (-3.64-4.3) |
| HIV/AIDS | Vietnam | 3787.1 (2864.9-4818.5) | 5.4 (4.1-6.9) | 14029.2 (11074.8-18930) | 14.1 (11.2-18.5) | 0.32 (-3.54-4.34) |
| HIV/AIDS | Yemen | 60.4 (26.7-133.5) | 0.6 (0.2-1.3) | 1436.6 (600.1-2491.8) | 4.6 (1.9-8) | 0.51 (-3.57-4.77) |
| HIV/AIDS | Zambia | 103504.3 (87123.5-122946.8) | 1463.9 (1210.7-1771.5) | 37570 (22021.3-60449.3) | 205.7 (119.3-331.4) | -0.88 (-4.51-2.9) |
| HIV/AIDS | Zimbabwe | 220610.7 (195057.9-250664.2) | 2495.6 (2184.3-2859.1) | 20305.2 (14016-28819.4) | 138.7 (95.2-199.7) | -1.3 (-4.89-2.44) |
| Other sexually transmitted infections | Afghanistan | 0 (0-0) | 0 (0-0) | 0 (0-0) | 0 (0-0) | 0.27 (-3.89-4.6) |
| Other sexually transmitted infections | Albania | 0 (0-0) | 0 (0-0) | 0 (0-0) | 0 (0-0) | -0.11 (-4.53-4.51) |
| Other sexually transmitted infections | Algeria | 0 (0-0) | 0 (0-0) | 0 (0-0) | 0 (0-0) | 0.49 (-3.58-4.74) |
| Other sexually transmitted infections | American Samoa | 0 (0-0) | 0 (0-0) | 0 (0-0) | 0 (0-0) | 0.96 (-3.19-5.29) |
| Other sexually transmitted infections | Andorra | 0 (0-0) | 0 (0-0) | 0 (0-0) | 0 (0-0) | -0.13 (-3.69-3.57) |
| Other sexually transmitted infections | Angola | 0 (0-0) | 0 (0-0) | 0 (0-0) | 0 (0-0) | 0.43 (-3.19-4.19) |
| Other sexually transmitted infections | Antigua and Barbuda | 0 (0-0) | 0 (0-0) | 0 (0-0) | 0 (0-0) | -0.21 (-4.12-3.85) |
| Other sexually transmitted infections | Argentina | 0 (0-0) | 0 (0-0) | 0 (0-0) | 0 (0-0) | -0.06 (-3.49-3.5) |
| Other sexually transmitted infections | Armenia | 0 (0-0) | 0 (0-0) | 0 (0-0) | 0 (0-0) | 0.92 (-3.39-5.42) |
| Other sexually transmitted infections | Australia | 0 (0-0) | 0 (0-0) | 0 (0-0) | 0 (0-0) | -0.13 (-3.7-3.57) |
| Other sexually transmitted infections | Austria | 0 (0-0) | 0 (0-0) | 0 (0-0) | 0 (0-0) | -0.37 (-3.76-3.13) |
| Other sexually transmitted infections | Azerbaijan | 0 (0-0) | 0 (0-0) | 0 (0-0) | 0 (0-0) | 0 (-4.41-4.62) |
| Other sexually transmitted infections | Bahrain | 0 (0-0) | 0 (0-0) | 0 (0-0) | 0 (0-0) | -0.25 (-4.4-4.07) |
| Other sexually transmitted infections | Bangladesh | 0 (0-0) | 0 (0-0) | 0 (0-0) | 0 (0-0) | 0.02 (-4.03-4.25) |
| Other sexually transmitted infections | Barbados | 0 (0-0) | 0 (0-0) | 0 (0-0) | 0 (0-0) | -0.19 (-4.02-3.8) |
| Other sexually transmitted infections | Belarus | 0 (0-0) | 0 (0-0) | 0 (0-0) | 0 (0-0) | 0.97 (-3.01-5.1) |
| Other sexually transmitted infections | Belgium | 0 (0-0) | 0 (0-0) | 0 (0-0) | 0 (0-0) | -0.14 (-3.44-3.28) |
| Other sexually transmitted infections | Belize | 0 (0-0) | 0 (0-0) | 0 (0-0) | 0 (0-0) | -0.21 (-3.99-3.72) |
| Other sexually transmitted infections | Benin | 0 (0-0) | 0 (0-0) | 0 (0-0) | 0 (0-0) | -0.5 (-4.08-3.21) |
| Other sexually transmitted infections | Bermuda | 0 (0-0) | 0 (0-0) | 0 (0-0) | 0 (0-0) | -0.48 (-4.39-3.6) |
| Other sexually transmitted infections | Bhutan | 0 (0-0) | 0 (0-0) | 0 (0-0) | 0 (0-0) | 0.16 (-3.49-3.96) |
| Other sexually transmitted infections | Bolivia | 0 (0-0) | 0 (0-0) | 0 (0-0) | 0 (0-0) | -0.33 (-4.02-3.51) |
| Other sexually transmitted infections | Bosnia and Herzegovina | 0 (0-0) | 0 (0-0) | 0 (0-0) | 0 (0-0) | 0.06 (-4.35-4.67) |
| Other sexually transmitted infections | Botswana | 0 (0-0) | 0 (0-0) | 0 (0-0) | 0 (0-0) | -1.25 (-4.85-2.49) |
| Other sexually transmitted infections | Brazil | 0 (0-0) | 0 (0-0) | 0 (0-0) | 0 (0-0) | 0.06 (-3.86-4.14) |
| Other sexually transmitted infections | Brunei | 0 (0-0) | 0 (0-0) | 0 (0-0) | 0 (0-0) | 0.32 (-3.39-4.18) |
| Other sexually transmitted infections | Bulgaria | 0 (0-0) | 0 (0-0) | 0 (0-0) | 0 (0-0) | 0.4 (-3.8-4.78) |
| Other sexually transmitted infections | Burkina Faso | 0 (0-0) | 0 (0-0) | 0 (0-0) | 0 (0-0) | -1.47 (-5.19-2.41) |
| Other sexually transmitted infections | Burundi | 0 (0-0) | 0 (0-0) | 0 (0-0) | 0 (0-0) | -1.89 (-5.64-2.01) |
| Other sexually transmitted infections | Cambodia | 0 (0-0) | 0 (0-0) | 0 (0-0) | 0 (0-0) | -0.55 (-4.4-3.45) |
| Other sexually transmitted infections | Cameroon | 0 (0-0) | 0 (0-0) | 0 (0-0) | 0 (0-0) | -0.82 (-4.42-2.91) |
| Other sexually transmitted infections | Canada | 0 (0-0) | 0 (0-0) | 0 (0-0) | 0 (0-0) | 0.1 (-3.42-3.74) |
| Other sexually transmitted infections | Cape Verde | 0 (0-0) | 0 (0-0) | 0 (0-0) | 0 (0-0) | -0.72 (-4.4-3.11) |
| Other sexually transmitted infections | Central African Republic | 0 (0-0) | 0 (0-0) | 0 (0-0) | 0 (0-0) | -0.86 (-4.39-2.79) |
| Other sexually transmitted infections | Chad | 0 (0-0) | 0 (0-0) | 0 (0-0) | 0 (0-0) | -0.59 (-4.2-3.15) |
| Other sexually transmitted infections | Chile | 0 (0-0) | 0 (0-0) | 0 (0-0) | 0 (0-0) | 0.38 (-3.11-4.01) |
| Other sexually transmitted infections | China | 0 (0-0) | 0 (0-0) | 0 (0-0) | 0 (0-0) | -0.08 (-4.2-4.23) |
| Other sexually transmitted infections | Colombia | 0 (0-0) | 0 (0-0) | 0 (0-0) | 0 (0-0) | 0.24 (-3.66-4.29) |
| Other sexually transmitted infections | Comoros | 0 (0-0) | 0 (0-0) | 0 (0-0) | 0 (0-0) | -0.17 (-4.66-4.53) |
| Other sexually transmitted infections | Congo | 0 (0-0) | 0 (0-0) | 0 (0-0) | 0 (0-0) | -0.53 (-4.06-3.13) |
| Other sexually transmitted infections | Cook Islands | 0 (0-0) | 0 (0-0) | 0 (0-0) | 0 (0-0) | 1.05 (-3.11-5.39) |
| Other sexually transmitted infections | Costa Rica | 0 (0-0) | 0 (0-0) | 0 (0-0) | 0 (0-0) | 0.07 (-3.98-4.28) |
| Other sexually transmitted infections | Cote d'Ivoire | 0 (0-0) | 0 (0-0) | 0 (0-0) | 0 (0-0) | -1.18 (-4.71-2.49) |
| Other sexually transmitted infections | Croatia | 0 (0-0) | 0 (0-0) | 0 (0-0) | 0 (0-0) | 0.08 (-4.16-4.5) |
| Other sexually transmitted infections | Cuba | 0 (0-0) | 0 (0-0) | 0 (0-0) | 0 (0-0) | 0.92 (-3.19-5.22) |
| Other sexually transmitted infections | Cyprus | 0 (0-0) | 0 (0-0) | 0 (0-0) | 0 (0-0) | 0.04 (-3.63-3.85) |
| Other sexually transmitted infections | Czech Republic | 0 (0-0) | 0 (0-0) | 0 (0-0) | 0 (0-0) | 0.39 (-3.87-4.83) |
| Other sexually transmitted infections | Democratic Republic of the Congo | 0 (0-0) | 0 (0-0) | 0 (0-0) | 0 (0-0) | -1.3 (-4.94-2.49) |
| Other sexually transmitted infections | Denmark | 0 (0-0) | 0 (0-0) | 0 (0-0) | 0 (0-0) | -0.26 (-3.73-3.34) |
| Other sexually transmitted infections | Djibouti | 0 (0-0) | 0 (0-0) | 0 (0-0) | 0 (0-0) | -0.19 (-3.94-3.7) |
| Other sexually transmitted infections | Dominica | 0 (0-0) | 0 (0-0) | 0 (0-0) | 0 (0-0) | -0.21 (-4.16-3.9) |
| Other sexually transmitted infections | Dominican Republic | 0 (0-0) | 0 (0-0) | 0 (0-0) | 0 (0-0) | -0.71 (-4.46-3.19) |
| Other sexually transmitted infections | Ecuador | 0 (0-0) | 0 (0-0) | 0 (0-0) | 0 (0-0) | 0.54 (-3.22-4.44) |
| Other sexually transmitted infections | Egypt | 0 (0-0) | 0 (0-0) | 0 (0-0) | 0 (0-0) | 0 (-4.33-4.53) |
| Other sexually transmitted infections | El Salvador | 0 (0-0) | 0 (0-0) | 0 (0-0) | 0 (0-0) | -0.23 (-4.16-3.86) |
| Other sexually transmitted infections | Equatorial Guinea | 0 (0-0) | 0 (0-0) | 0 (0-0) | 0 (0-0) | 0.57 (-2.97-4.24) |
| Other sexually transmitted infections | Eritrea | 0 (0-0) | 0 (0-0) | 0 (0-0) | 0 (0-0) | -1.3 (-5.1-2.65) |
| Other sexually transmitted infections | Estonia | 0 (0-0) | 0 (0-0) | 0 (0-0) | 0 (0-0) | 0.65 (-3.26-4.73) |
| Other sexually transmitted infections | Ethiopia | 0 (0-0) | 0 (0-0) | 0 (0-0) | 0 (0-0) | -1.38 (-5.11-2.5) |
| Other sexually transmitted infections | Federated States of Micronesia | 0 (0-0) | 0 (0-0) | 0 (0-0) | 0 (0-0) | 0.99 (-3.14-5.3) |
| Other sexually transmitted infections | Fiji | 0 (0-0) | 0 (0-0) | 0 (0-0) | 0 (0-0) | 0.08 (-4.15-4.5) |
| Other sexually transmitted infections | Finland | 0 (0-0) | 0 (0-0) | 0 (0-0) | 0 (0-0) | -0.17 (-3.68-3.47) |
| Other sexually transmitted infections | France | 0 (0-0) | 0 (0-0) | 0 (0-0) | 0 (0-0) | 0.07 (-3.21-3.47) |
| Other sexually transmitted infections | Gabon | 0 (0-0) | 0 (0-0) | 0 (0-0) | 0 (0-0) | -0.52 (-4.04-3.14) |
| Other sexually transmitted infections | Georgia | 0 (0-0) | 0 (0-0) | 0 (0-0) | 0 (0-0) | 0.67 (-3.52-5.04) |
| Other sexually transmitted infections | Germany | 0 (0-0) | 0 (0-0) | 0 (0-0) | 0 (0-0) | -0.12 (-3.64-3.52) |
| Other sexually transmitted infections | Ghana | 0 (0-0) | 0 (0-0) | 0 (0-0) | 0 (0-0) | -0.48 (-4.16-3.34) |
| Other sexually transmitted infections | Greece | 0 (0-0) | 0 (0-0) | 0 (0-0) | 0 (0-0) | 0.58 (-2.98-4.27) |
| Other sexually transmitted infections | Greenland | 0 (0-0) | 0 (0-0) | 0 (0-0) | 0 (0-0) | 0.04 (-3.4-3.61) |
| Other sexually transmitted infections | Grenada | 0 (0-0) | 0 (0-0) | 0 (0-0) | 0 (0-0) | -0.4 (-4.39-3.75) |
| Other sexually transmitted infections | Guam | 0 (0-0) | 0 (0-0) | 0 (0-0) | 0 (0-0) | 0.1 (-4.06-4.44) |
| Other sexually transmitted infections | Guatemala | 0 (0-0) | 0 (0-0) | 0 (0-0) | 0 (0-0) | -0.51 (-4.52-3.67) |
| Other sexually transmitted infections | Guinea | 0 (0-0) | 0 (0-0) | 0 (0-0) | 0 (0-0) | -0.32 (-3.93-3.42) |
| Other sexually transmitted infections | Guinea-Bissau | 0 (0-0) | 0 (0-0) | 0 (0-0) | 0 (0-0) | 0.28 (-3.3-3.99) |
| Other sexually transmitted infections | Guyana | 0 (0-0) | 0 (0-0) | 0 (0-0) | 0 (0-0) | -0.3 (-4.05-3.59) |
| Other sexually transmitted infections | Haiti | 0 (0-0) | 0 (0-0) | 0 (0-0) | 0 (0-0) | -0.6 (-4.18-3.11) |
| Other sexually transmitted infections | Honduras | 0 (0-0) | 0 (0-0) | 0 (0-0) | 0 (0-0) | -0.35 (-4.55-4.04) |
| Other sexually transmitted infections | Hungary | 0 (0-0) | 0 (0-0) | 0 (0-0) | 0 (0-0) | 0.36 (-3.86-4.77) |
| Other sexually transmitted infections | Iceland | 0 (0-0) | 0 (0-0) | 0 (0-0) | 0 (0-0) | 0.38 (-3.03-3.91) |
| Other sexually transmitted infections | India | 0 (0-0) | 0 (0-0) | 0 (0-0) | 0 (0-0) | -0.44 (-4.05-3.31) |
| Other sexually transmitted infections | Indonesia | 0 (0-0) | 0 (0-0) | 0 (0-0) | 0 (0-0) | 0.56 (-3.67-4.97) |
| Other sexually transmitted infections | Iran | 0 (0-0) | 0 (0-0) | 0 (0-0) | 0 (0-0) | 0.47 (-3.83-4.96) |
| Other sexually transmitted infections | Iraq | 0 (0-0) | 0 (0-0) | 0 (0-0) | 0 (0-0) | -0.11 (-4.46-4.44) |
| Other sexually transmitted infections | Ireland | 0 (0-0) | 0 (0-0) | 0 (0-0) | 0 (0-0) | -0.07 (-3.39-3.35) |
| Other sexually transmitted infections | Israel | 0 (0-0) | 0 (0-0) | 0 (0-0) | 0 (0-0) | 0.11 (-3.29-3.62) |
| Other sexually transmitted infections | Italy | 0 (0-0) | 0 (0-0) | 0 (0-0) | 0 (0-0) | -0.26 (-3.6-3.19) |
| Other sexually transmitted infections | Jamaica | 0 (0-0) | 0 (0-0) | 0 (0-0) | 0 (0-0) | -0.1 (-3.89-3.85) |
| Other sexually transmitted infections | Japan | 0 (0-0) | 0 (0-0) | 0 (0-0) | 0 (0-0) | 0.26 (-3.62-4.31) |
| Other sexually transmitted infections | Jordan | 0 (0-0) | 0 (0-0) | 0 (0-0) | 0 (0-0) | -0.02 (-4.11-4.25) |
| Other sexually transmitted infections | Kazakhstan | 0 (0-0) | 0 (0-0) | 0 (0-0) | 0 (0-0) | 0.39 (-3.64-4.58) |
| Other sexually transmitted infections | Kenya | 0 (0-0) | 0 (0-0) | 0 (0-0) | 0 (0-0) | -1.43 (-5.05-2.32) |
| Other sexually transmitted infections | Kiribati | 0 (0-0) | 0 (0-0) | 0 (0-0) | 0 (0-0) | -0.14 (-4.48-4.4) |
| Other sexually transmitted infections | Kuwait | 0 (0-0) | 0 (0-0) | 0 (0-0) | 0 (0-0) | -0.27 (-4.48-4.13) |
| Other sexually transmitted infections | Kyrgyzstan | 0 (0-0) | 0 (0-0) | 0 (0-0) | 0 (0-0) | 0.86 (-3.29-5.19) |
| Other sexually transmitted infections | Laos | 0 (0-0) | 0 (0-0) | 0 (0-0) | 0 (0-0) | 0.67 (-3.38-4.9) |
| Other sexually transmitted infections | Latvia | 0 (0-0) | 0 (0-0) | 0 (0-0) | 0 (0-0) | 0.27 (-3.61-4.31) |
| Other sexually transmitted infections | Lebanon | 0 (0-0) | 0 (0-0) | 0 (0-0) | 0 (0-0) | 0.32 (-3.65-4.46) |
| Other sexually transmitted infections | Lesotho | 0 (0-0) | 0 (0-0) | 0 (0-0) | 0 (0-0) | -0.33 (-3.99-3.46) |
| Other sexually transmitted infections | Liberia | 0 (0-0) | 0 (0-0) | 0 (0-0) | 0 (0-0) | -0.48 (-4.09-3.26) |
| Other sexually transmitted infections | Libya | 0 (0-0) | 0 (0-0) | 0 (0-0) | 0 (0-0) | 0.15 (-3.89-4.36) |
| Other sexually transmitted infections | Lithuania | 0 (0-0) | 0 (0-0) | 0 (0-0) | 0 (0-0) | 0.45 (-3.61-4.68) |
| Other sexually transmitted infections | Luxembourg | 0 (0-0) | 0 (0-0) | 0 (0-0) | 0 (0-0) | -0.24 (-3.47-3.11) |
| Other sexually transmitted infections | Macedonia | 0 (0-0) | 0 (0-0) | 0 (0-0) | 0 (0-0) | 0.06 (-4.35-4.68) |
| Other sexually transmitted infections | Madagascar | 0 (0-0) | 0 (0-0) | 0 (0-0) | 0 (0-0) | 0.59 (-3.5-4.85) |
| Other sexually transmitted infections | Malawi | 0 (0-0) | 0 (0-0) | 0 (0-0) | 0 (0-0) | -0.93 (-4.53-2.81) |
| Other sexually transmitted infections | Malaysia | 0 (0-0) | 0 (0-0) | 0 (0-0) | 0 (0-0) | -0.14 (-4.07-3.94) |
| Other sexually transmitted infections | Maldives | 0 (0-0) | 0 (0-0) | 0 (0-0) | 0 (0-0) | 0.08 (-4.31-4.67) |
| Other sexually transmitted infections | Mali | 0 (0-0) | 0 (0-0) | 0 (0-0) | 0 (0-0) | -0.76 (-4.46-3.08) |
| Other sexually transmitted infections | Malta | 0 (0-0) | 0 (0-0) | 0 (0-0) | 0 (0-0) | 0.67 (-2.67-4.13) |
| Other sexually transmitted infections | Marshall Islands | 0 (0-0) | 0 (0-0) | 0 (0-0) | 0 (0-0) | 0.87 (-3.11-5.02) |
| Other sexually transmitted infections | Mauritania | 0 (0-0) | 0 (0-0) | 0 (0-0) | 0 (0-0) | -0.14 (-4.38-4.29) |
| Other sexually transmitted infections | Mauritius | 0 (0-0) | 0 (0-0) | 0 (0-0) | 0 (0-0) | 0.69 (-3.22-4.77) |
| Other sexually transmitted infections | Mexico | 0 (0-0) | 0 (0-0) | 0 (0-0) | 0 (0-0) | 0.09 (-3.94-4.29) |
| Other sexually transmitted infections | Moldova | 0 (0-0) | 0 (0-0) | 0 (0-0) | 0 (0-0) | 0.22 (-3.64-4.23) |
| Other sexually transmitted infections | Monaco | 0 (0-0) | 0 (0-0) | 0 (0-0) | 0 (0-0) | -0.03 (-3.59-3.66) |
| Other sexually transmitted infections | Mongolia | 0 (0-0) | 0 (0-0) | 0 (0-0) | 0 (0-0) | 0.37 (-4.08-5.03) |
| Other sexually transmitted infections | Montenegro | 0 (0-0) | 0 (0-0) | 0 (0-0) | 0 (0-0) | 0 (-4.31-4.51) |
| Other sexually transmitted infections | Morocco | 0 (0-0) | 0 (0-0) | 0 (0-0) | 0 (0-0) | -0.27 (-4.32-3.94) |
| Other sexually transmitted infections | Mozambique | 0 (0-0) | 0 (0-0) | 0 (0-0) | 0 (0-0) | -0.21 (-3.9-3.63) |
| Other sexually transmitted infections | Myanmar | 0 (0-0) | 0 (0-0) | 0 (0-0) | 0 (0-0) | -0.3 (-4.05-3.6) |
| Other sexually transmitted infections | Namibia | 0 (0-0) | 0 (0-0) | 0 (0-0) | 0 (0-0) | -0.75 (-4.39-3.03) |
| Other sexually transmitted infections | Nauru | 0 (0-0) | 0 (0-0) | 0 (0-0) | 0 (0-0) | 1.02 (-3.14-5.35) |
| Other sexually transmitted infections | Nepal | 0 (0-0) | 0 (0-0) | 0 (0-0) | 0 (0-0) | 0.14 (-3.56-3.98) |
| Other sexually transmitted infections | Netherlands | 0 (0-0) | 0 (0-0) | 0 (0-0) | 0 (0-0) | -0.06 (-3.48-3.47) |
| Other sexually transmitted infections | New Zealand | 0 (0-0) | 0 (0-0) | 0 (0-0) | 0 (0-0) | 0.13 (-3.66-4.07) |
| Other sexually transmitted infections | Nicaragua | 0 (0-0) | 0 (0-0) | 0 (0-0) | 0 (0-0) | 0.45 (-3.69-4.78) |
| Other sexually transmitted infections | Niger | 0 (0-0) | 0 (0-0) | 0 (0-0) | 0 (0-0) | -1.21 (-4.97-2.7) |
| Other sexually transmitted infections | Nigeria | 0 (0-0) | 0 (0-0) | 0 (0-0) | 0 (0-0) | -0.41 (-4.07-3.4) |
| Other sexually transmitted infections | Niue | 0 (0-0) | 0 (0-0) | 0 (0-0) | 0 (0-0) | 0.96 (-3.19-5.28) |
| Other sexually transmitted infections | North Korea | 0 (0-0) | 0 (0-0) | 0 (0-0) | 0 (0-0) | 0.61 (-3.52-4.92) |
| Other sexually transmitted infections | Northern Mariana Islands | 0 (0-0) | 0 (0-0) | 0 (0-0) | 0 (0-0) | 0.11 (-4.12-4.52) |
| Other sexually transmitted infections | Norway | 0 (0-0) | 0 (0-0) | 0 (0-0) | 0 (0-0) | -0.12 (-3.57-3.45) |
| Other sexually transmitted infections | Oman | 0 (0-0) | 0 (0-0) | 0 (0-0) | 0 (0-0) | 0.21 (-3.9-4.5) |
| Other sexually transmitted infections | Pakistan | 0 (0-0) | 0 (0-0) | 0 (0-0) | 0 (0-0) | 1.1 (-2.86-5.23) |
| Other sexually transmitted infections | Palau | 0 (0-0) | 0 (0-0) | 0 (0-0) | 0 (0-0) | 0.87 (-3.28-5.19) |
| Other sexually transmitted infections | Palestine | 0 (0-0) | 0 (0-0) | 0 (0-0) | 0 (0-0) | 0.16 (-4.12-4.63) |
| Other sexually transmitted infections | Panama | 0 (0-0) | 0 (0-0) | 0 (0-0) | 0 (0-0) | 0.21 (-3.6-4.17) |
| Other sexually transmitted infections | Papua New Guinea | 0 (0-0) | 0 (0-0) | 0 (0-0) | 0 (0-0) | 0.53 (-3.47-4.7) |
| Other sexually transmitted infections | Paraguay | 0 (0-0) | 0 (0-0) | 0 (0-0) | 0 (0-0) | 0.46 (-3.59-4.68) |
| Other sexually transmitted infections | Peru | 0 (0-0) | 0 (0-0) | 0 (0-0) | 0 (0-0) | 0.01 (-3.81-3.99) |
| Other sexually transmitted infections | Philippines | 0 (0-0) | 0 (0-0) | 0 (0-0) | 0 (0-0) | 1.16 (-2.86-5.36) |
| Other sexually transmitted infections | Poland | 0 (0-0) | 0 (0-0) | 0 (0-0) | 0 (0-0) | 0.18 (-4.01-4.55) |
| Other sexually transmitted infections | Portugal | 0 (0-0) | 0 (0-0) | 0 (0-0) | 0 (0-0) | -0.73 (-3.96-2.61) |
| Other sexually transmitted infections | Puerto Rico | 0 (0-0) | 0 (0-0) | 0 (0-0) | 0 (0-0) | -0.9 (-4.73-3.08) |
| Other sexually transmitted infections | Qatar | 0 (0-0) | 0 (0-0) | 0 (0-0) | 0 (0-0) | -0.23 (-4.5-4.22) |
| Other sexually transmitted infections | Romania | 0 (0-0) | 0 (0-0) | 0 (0-0) | 0 (0-0) | 0.28 (-3.82-4.56) |
| Other sexually transmitted infections | Russian Federation | 0 (0-0) | 0 (0-0) | 0 (0-0) | 0 (0-0) | 1.19 (-2.71-5.26) |
| Other sexually transmitted infections | Rwanda | 0 (0-0) | 0 (0-0) | 0 (0-0) | 0 (0-0) | -0.97 (-4.64-2.84) |
| Other sexually transmitted infections | Saint Kitts and Nevis | 0 (0-0) | 0 (0-0) | 0 (0-0) | 0 (0-0) | -0.03 (-3.79-3.88) |
| Other sexually transmitted infections | Saint Lucia | 0 (0-0) | 0 (0-0) | 0 (0-0) | 0 (0-0) | -0.41 (-4.16-3.49) |
| Other sexually transmitted infections | Saint Vincent and the Grenadines | 0 (0-0) | 0 (0-0) | 0 (0-0) | 0 (0-0) | -0.51 (-4.33-3.47) |
| Other sexually transmitted infections | Samoa | 0 (0-0) | 0 (0-0) | 0 (0-0) | 0 (0-0) | 0.97 (-3.28-5.41) |
| Other sexually transmitted infections | San Marino | 0 (0-0) | 0 (0-0) | 0 (0-0) | 0 (0-0) | -0.07 (-3.62-3.61) |
| Other sexually transmitted infections | Sao Tome and Principe | 0 (0-0) | 0 (0-0) | 0 (0-0) | 0 (0-0) | -0.49 (-4.64-3.84) |
| Other sexually transmitted infections | Saudi Arabia | 0 (0-0) | 0 (0-0) | 0 (0-0) | 0 (0-0) | -0.03 (-4.22-4.34) |
| Other sexually transmitted infections | Senegal | 0 (0-0) | 0 (0-0) | 0 (0-0) | 0 (0-0) | -0.7 (-4.3-3.04) |
| Other sexually transmitted infections | Serbia | 0 (0-0) | 0 (0-0) | 0 (0-0) | 0 (0-0) | 0 (-4.35-4.55) |
| Other sexually transmitted infections | Seychelles | 0 (0-0) | 0 (0-0) | 0 (0-0) | 0 (0-0) | -0.26 (-4.28-3.94) |
| Other sexually transmitted infections | Sierra Leone | 0 (0-0) | 0 (0-0) | 0 (0-0) | 0 (0-0) | -0.21 (-3.84-3.55) |
| Other sexually transmitted infections | Singapore | 0 (0-0) | 0 (0-0) | 0 (0-0) | 0 (0-0) | -0.46 (-3.96-3.18) |
| Other sexually transmitted infections | Slovakia | 0 (0-0) | 0 (0-0) | 0 (0-0) | 0 (0-0) | 0.05 (-4.36-4.66) |
| Other sexually transmitted infections | Slovenia | 0 (0-0) | 0 (0-0) | 0 (0-0) | 0 (0-0) | -0.2 (-4.32-4.1) |
| Other sexually transmitted infections | Solomon Islands | 0 (0-0) | 0 (0-0) | 0 (0-0) | 0 (0-0) | 0.94 (-3.15-5.19) |
| Other sexually transmitted infections | Somalia | 0 (0-0) | 0 (0-0) | 0 (0-0) | 0 (0-0) | -0.55 (-4.47-3.53) |
| Other sexually transmitted infections | South Africa | 0 (0-0) | 0 (0-0) | 0 (0-0) | 0 (0-0) | -0.52 (-4.33-3.44) |
| Other sexually transmitted infections | South Korea | 0 (0-0) | 0 (0-0) | 0 (0-0) | 0 (0-0) | 0.02 (-3.88-4.08) |
| Other sexually transmitted infections | South Sudan | 0 (0-0) | 0 (0-0) | 0 (0-0) | 0 (0-0) | -0.09 (-3.83-3.8) |
| Other sexually transmitted infections | Spain | 0 (0-0) | 0 (0-0) | 0 (0-0) | 0 (0-0) | -0.6 (-3.89-2.82) |
| Other sexually transmitted infections | Sri Lanka | 0 (0-0) | 0 (0-0) | 0 (0-0) | 0 (0-0) | 0.01 (-4.41-4.63) |
| Other sexually transmitted infections | Sudan | 0 (0-0) | 0 (0-0) | 0 (0-0) | 0 (0-0) | 0.13 (-3.67-4.08) |
| Other sexually transmitted infections | Suriname | 0 (0-0) | 0 (0-0) | 0 (0-0) | 0 (0-0) | -0.09 (-3.89-3.85) |
| Other sexually transmitted infections | Swaziland | 0 (0-0) | 0 (0-0) | 0 (0-0) | 0 (0-0) | -0.24 (-3.91-3.57) |
| Other sexually transmitted infections | Sweden | 0 (0-0) | 0 (0-0) | 0 (0-0) | 0 (0-0) | -0.15 (-3.64-3.47) |
| Other sexually transmitted infections | Switzerland | 0 (0-0) | 0 (0-0) | 0 (0-0) | 0 (0-0) | -0.79 (-4.08-2.62) |
| Other sexually transmitted infections | Syria | 0 (0-0) | 0 (0-0) | 0 (0-0) | 0 (0-0) | 0 (-4.28-4.47) |
| Other sexually transmitted infections | Taiwan | 0 (0-0) | 0 (0-0) | 0 (0-0) | 0 (0-0) | 0.43 (-3.63-4.67) |
| Other sexually transmitted infections | Tajikistan | 0 (0-0) | 0 (0-0) | 0 (0-0) | 0 (0-0) | 0.14 (-4.11-4.59) |
| Other sexually transmitted infections | Tanzania | 0 (0-0) | 0 (0-0) | 0 (0-0) | 0 (0-0) | -1.24 (-4.97-2.64) |
| Other sexually transmitted infections | Thailand | 0 (0-0) | 0 (0-0) | 0 (0-0) | 0 (0-0) | -0.72 (-4.45-3.15) |
| Other sexually transmitted infections | The Bahamas | 0 (0-0) | 0 (0-0) | 0 (0-0) | 0 (0-0) | -0.3 (-4.02-3.56) |
| Other sexually transmitted infections | The Gambia | 0 (0-0) | 0 (0-0) | 0 (0-0) | 0 (0-0) | 0.14 (-3.42-3.83) |
| Other sexually transmitted infections | Timor-Leste | 0 (0-0) | 0 (0-0) | 0 (0-0) | 0 (0-0) | -0.2 (-4.01-3.76) |
| Other sexually transmitted infections | Togo | 0 (0-0) | 0 (0-0) | 0 (0-0) | 0 (0-0) | -0.96 (-4.47-2.68) |
| Other sexually transmitted infections | Tokelau | 0 (0-0) | 0 (0-0) | 0 (0-0) | 0 (0-0) | 0.84 (-3.31-5.17) |
| Other sexually transmitted infections | Tonga | 0 (0-0) | 0 (0-0) | 0 (0-0) | 0 (0-0) | 1.02 (-3.16-5.37) |
| Other sexually transmitted infections | Trinidad and Tobago | 0 (0-0) | 0 (0-0) | 0 (0-0) | 0 (0-0) | -0.02 (-3.72-3.83) |
| Other sexually transmitted infections | Tunisia | 0 (0-0) | 0 (0-0) | 0 (0-0) | 0 (0-0) | 0.28 (-3.98-4.72) |
| Other sexually transmitted infections | Turkey | 0 (0-0) | 0 (0-0) | 0 (0-0) | 0 (0-0) | -0.04 (-4.32-4.44) |
| Other sexually transmitted infections | Turkmenistan | 0 (0-0) | 0 (0-0) | 0 (0-0) | 0 (0-0) | -0.39 (-4.59-4) |
| Other sexually transmitted infections | Tuvalu | 0 (0-0) | 0 (0-0) | 0 (0-0) | 0 (0-0) | 0.84 (-3.32-5.17) |
| Other sexually transmitted infections | Uganda | 0 (0-0) | 0 (0-0) | 0 (0-0) | 0 (0-0) | -0.66 (-4.3-3.11) |
| Other sexually transmitted infections | Ukraine | 0 (0-0) | 0 (0-0) | 0 (0-0) | 0 (0-0) | 0.33 (-3.48-4.29) |
| Other sexually transmitted infections | United Arab Emirates | 0 (0-0) | 0 (0-0) | 0 (0-0) | 0 (0-0) | 0.15 (-4.02-4.5) |
| Other sexually transmitted infections | United Kingdom | 0 (0-0) | 0 (0-0) | 0 (0-0) | 0 (0-0) | 0 (-3.29-3.4) |
| Other sexually transmitted infections | United States | 0 (0-0) | 0 (0-0) | 0 (0-0) | 0 (0-0) | -0.15 (-3.67-3.5) |
| Other sexually transmitted infections | Uruguay | 0 (0-0) | 0 (0-0) | 0 (0-0) | 0 (0-0) | 0.16 (-3.27-3.71) |
| Other sexually transmitted infections | Uzbekistan | 0 (0-0) | 0 (0-0) | 0 (0-0) | 0 (0-0) | 0.53 (-3.58-4.81) |
| Other sexually transmitted infections | Vanuatu | 0 (0-0) | 0 (0-0) | 0 (0-0) | 0 (0-0) | 0.92 (-3.27-5.3) |
| Other sexually transmitted infections | Venezuela | 0 (0-0) | 0 (0-0) | 0 (0-0) | 0 (0-0) | 0.25 (-3.64-4.3) |
| Other sexually transmitted infections | Vietnam | 0 (0-0) | 0 (0-0) | 0 (0-0) | 0 (0-0) | 0.32 (-3.54-4.34) |
| Other sexually transmitted infections | Yemen | 0 (0-0) | 0 (0-0) | 0 (0-0) | 0 (0-0) | 0.51 (-3.57-4.77) |
| Other sexually transmitted infections | Zambia | 0 (0-0) | 0 (0-0) | 0 (0-0) | 0 (0-0) | -0.88 (-4.51-2.9) |
| Other sexually transmitted infections | Zimbabwe | 0 (0-0) | 0 (0-0) | 0 (0-0) | 0 (0-0) | -1.3 (-4.89-2.44) |
| Sexually transmitted infections excluding HIV | Afghanistan | 579338.5 (490719.1-690755.7) | 7853.4 (6626.2-9475.4) | 2108508.4 (1758025.3-2517463.4) | 7895.8 (6671.8-9495.8) | 0.27 (-3.89-4.6) |
| Sexually transmitted infections excluding HIV | Albania | 300812.2 (246720.2-361730.7) | 8899.9 (7419.2-10561) | 237357.7 (199195.3-285314) | 8711.3 (7235-10523.3) | -0.11 (-4.53-4.51) |
| Sexually transmitted infections excluding HIV | Algeria | 1689161.6 (1418700.9-2011347.7) | 7834.5 (6605.8-9419.5) | 3401969.2 (2799625.4-4146202.6) | 7335.3 (6120.9-8932.6) | 0.49 (-3.58-4.74) |
| Sexually transmitted infections excluding HIV | American Samoa | 5473.8 (4564.7-6559.8) | 11928.8 (10026.7-14558.7) | 5589.5 (4676.2-6759.2) | 11625.3 (9655-14196.5) | 0.96 (-3.19-5.29) |
| Sexually transmitted infections excluding HIV | Andorra | 2224 (1771.5-2768.2) | 3346.8 (2700.4-4158.7) | 3275.9 (2695.3-4234.6) | 3275.1 (2653.9-4104.7) | -0.13 (-3.69-3.57) |
| Sexually transmitted infections excluding HIV | Angola | 1032836.6 (885825.8-1209922) | 11906.9 (10256.1-14093.9) | 3113085.1 (2652217.5-3632854.2) | 11425.8 (9708.4-13530) | 0.43 (-3.19-4.19) |
| Sexually transmitted infections excluding HIV | Antigua and Barbuda | 7210.5 (6021.4-8698.9) | 11822.5 (9835.4-14403) | 11784.6 (9860.3-14441.3) | 11781.6 (9825.3-14416.4) | -0.21 (-4.12-3.85) |
| Sexually transmitted infections excluding HIV | Argentina | 1568983.7 (1322743.4-1875097.6) | 4915.7 (4133.9-5889.9) | 2396780.3 (2014535.7-2913319.9) | 4944.7 (4165.3-6009.6) | -0.06 (-3.49-3.5) |
| Sexually transmitted infections excluding HIV | Armenia | 424093.6 (349626.6-512850.2) | 11968.3 (9959-14446.4) | 362422.7 (302742.4-443617.3) | 11581.7 (9660.9-14087.9) | 0.92 (-3.39-5.42) |
| Sexually transmitted infections excluding HIV | Australia | 830052.8 (685496.8-1011522.1) | 4549.1 (3786-5513.3) | 1195203.5 (992303.3-1469895.4) | 4442.9 (3687.6-5392.1) | -0.13 (-3.7-3.57) |
| Sexually transmitted infections excluding HIV | Austria | 273049 (220210.3-339325.1) | 3193.9 (2575.7-3980.3) | 310553.4 (253198.2-396396.3) | 3201.8 (2583.1-4045.1) | -0.37 (-3.76-3.13) |
| Sexually transmitted infections excluding HIV | Azerbaijan | 908728.5 (740676.6-1094683.7) | 12176.5 (10186.1-14561.7) | 1388332.8 (1155273.8-1687563.7) | 11758.1 (9803.6-14151.7) | 0 (-4.41-4.62) |
| Sexually transmitted infections excluding HIV | Bahrain | 47910.2 (38924.5-58790.5) | 8111.2 (6738.5-9813.5) | 160390.9 (133469-196504.9) | 8044.2 (6762.5-9738.7) | -0.25 (-4.4-4.07) |
| Sexually transmitted infections excluding HIV | Bangladesh | 5341858.2 (4531775.8-6300249.9) | 5850.9 (4949.3-6994) | 9767671.8 (8198592.9-11744747) | 5628.4 (4735.7-6747.6) | 0.02 (-4.03-4.25) |
| Sexually transmitted infections excluding HIV | Barbados | 35395.7 (29356.4-43199.8) | 13176.6 (10984.6-15980.3) | 40036.4 (33538.4-49409) | 13129.1 (10906-16106.8) | -0.19 (-4.02-3.8) |
| Sexually transmitted infections excluding HIV | Belarus | 974702.5 (814189.4-1179480.4) | 9068.2 (7591.6-10916) | 820341.5 (687458.3-1008272.8) | 8768.9 (7346.9-10597.9) | 0.97 (-3.01-5.1) |
| Sexually transmitted infections excluding HIV | Belgium | 341054.5 (278504.3-426140.4) | 3145.5 (2584.9-3945.7) | 369330.3 (302264.5-463635.7) | 3137.1 (2545.4-3882.8) | -0.14 (-3.44-3.28) |
| Sexually transmitted infections excluding HIV | Belize | 18227.9 (15124.5-21829.5) | 11783.4 (9778.9-14190.3) | 53460.7 (44908.4-65300.9) | 11752.2 (9793.8-14414.8) | -0.21 (-3.99-3.72) |
| Sexually transmitted infections excluding HIV | Benin | 381141 (320392-452029.2) | 10362.5 (8693.9-12539.5) | 1186491.8 (996387.2-1427701.2) | 10497.5 (8769.3-12840.4) | -0.5 (-4.08-3.21) |
| Sexually transmitted infections excluding HIV | Bermuda | 8286.9 (6864.3-10123.4) | 11715.6 (9794.5-14215.9) | 7285 (6139.6-8901.8) | 11643.7 (9739.4-14182.3) | -0.48 (-4.39-3.6) |
| Sexually transmitted infections excluding HIV | Bhutan | 32016.1 (27101.6-37782) | 5759.8 (4897.4-6890.2) | 48768.7 (40898-58097.6) | 5636.1 (4775-6715.2) | 0.16 (-3.49-3.96) |
| Sexually transmitted infections excluding HIV | Bolivia | 454258.1 (376701.7-541462) | 8271.3 (6906-9970.1) | 1004369.1 (832686.5-1221540.7) | 8186.9 (6794.2-9995.8) | -0.33 (-4.02-3.51) |
| Sexually transmitted infections excluding HIV | Bosnia and Herzegovina | 429669.7 (359701.5-517265.6) | 8703.6 (7292.4-10463) | 280554.9 (236628.2-335170.1) | 8620.6 (7168.6-10291.4) | 0.06 (-4.35-4.67) |
| Sexually transmitted infections excluding HIV | Botswana | 182925.3 (157765.6-212759.4) | 16089.1 (13860.6-18802.3) | 405975.4 (342480.1-482189.1) | 15178.8 (12920.8-18109) | -1.25 (-4.85-2.49) |
| Sexually transmitted infections excluding HIV | Brazil | 17009602.5 (14159514.7-20730166.5) | 11782.3 (9795.2-14451.9) | 28667512.4 (23767370-35332261) | 11756.6 (9805.4-14480.7) | 0.06 (-3.86-4.14) |
| Sexually transmitted infections excluding HIV | Brunei | 13660.4 (11165.1-16680.1) | 5022.8 (4191.8-6086.5) | 27601.5 (22896.3-34059.4) | 5009.6 (4188.4-6100.6) | 0.32 (-3.39-4.18) |
| Sexually transmitted infections excluding HIV | Bulgaria | 791409.1 (663945.3-958379.4) | 9065.6 (7450.5-11005.1) | 581751.5 (489236.1-717872.3) | 9005.9 (7483.9-10909.3) | 0.4 (-3.8-4.78) |
| Sexually transmitted infections excluding HIV | Burkina Faso | 1014331.9 (850355.9-1206971) | 14374.6 (12015.8-17263.6) | 2425737.6 (2012769.8-2927275) | 12979.6 (10769.7-15864.9) | -1.47 (-5.19-2.41) |
| Sexually transmitted infections excluding HIV | Burundi | 702595.9 (589988.6-839463.6) | 15842.4 (13301.7-19165.1) | 1793463.9 (1504752.5-2148787.2) | 15912.9 (13294.8-19156.8) | -1.89 (-5.64-2.01) |
| Sexually transmitted infections excluding HIV | Cambodia | 922760.3 (771477.6-1109772.5) | 10792.1 (9019.3-13125.3) | 1925607.3 (1594757.6-2357030.5) | 10672.8 (8954.4-13040.5) | -0.55 (-4.4-3.45) |
| Sexually transmitted infections excluding HIV | Cameroon | 1190056.7 (1012098.9-1406274.2) | 14033.1 (11892.2-16815.4) | 3915132.2 (3298249.6-4622860.2) | 13727.3 (11564.4-16474) | -0.82 (-4.42-2.91) |
| Sexually transmitted infections excluding HIV | Canada | 1548264.1 (1216341-1972986.2) | 5016.2 (3984.1-6362.8) | 1995982.4 (1585438.1-2554164.8) | 5140.2 (4074.4-6522.9) | 0.1 (-3.42-3.74) |
| Sexually transmitted infections excluding HIV | Cape Verde | 31355 (26238.5-37326.4) | 11621.4 (9635.3-14207) | 72702.4 (60208.4-88460.7) | 11637.7 (9793.7-14211.9) | -0.72 (-4.4-3.11) |
| Sexually transmitted infections excluding HIV | Central African Republic | 282089.8 (241282-331846) | 12051.9 (10274.8-14246.1) | 597522.6 (508600.4-703500.1) | 12002.9 (10256.3-14281.8) | -0.86 (-4.39-2.79) |
| Sexually transmitted infections excluding HIV | Chad | 544292.5 (454963.8-649146.3) | 11939.6 (9981.5-14526.1) | 1552387.2 (1302751.2-1864793.8) | 11878.8 (10014.4-14349.2) | -0.59 (-4.2-3.15) |
| Sexually transmitted infections excluding HIV | Chile | 662107.3 (552850.1-798691.9) | 4862 (4084.9-5872.6) | 999737.5 (836899.3-1215700.7) | 4885.1 (4095.2-5903.7) | 0.38 (-3.11-4.01) |
| Sexually transmitted infections excluding HIV | China | 123881005.4 (103500675-153569833.7) | 9824.2 (8165.7-12187.4) | 148273660.5 (122477073.2-184610446.2) | 9637 (7982.5-12045.8) | -0.08 (-4.2-4.23) |
| Sexually transmitted infections excluding HIV | Colombia | 3499671.3 (2880786.5-4220151.8) | 11084.4 (9217.5-13462.6) | 5927359.2 (4925100-7257014.4) | 11047.4 (9174.7-13552.5) | 0.24 (-3.66-4.29) |
| Sexually transmitted infections excluding HIV | Comoros | 58533.7 (49642.5-70269.2) | 15926 (13488.4-19356.4) | 118609.6 (98402.5-144172.3) | 15788.8 (13122.4-19165.6) | -0.17 (-4.66-4.53) |
| Sexually transmitted infections excluding HIV | Congo | 234017.5 (200694-272786.2) | 11485.2 (9835.1-13651.4) | 615341.1 (521555.1-732446.4) | 11442.5 (9682.2-13603.3) | -0.53 (-4.06-3.13) |
| Sexually transmitted infections excluding HIV | Cook Islands | 2154 (1780.6-2612.6) | 11981.7 (9887-14694.9) | 2067.6 (1738-2507.3) | 12063.4 (10021.1-14687.2) | 1.05 (-3.11-5.39) |
| Sexually transmitted infections excluding HIV | Costa Rica | 343522.9 (283091-413590.8) | 11763.3 (9794.6-14290) | 614476.6 (505216-754305.1) | 11722.2 (9649-14329.1) | 0.07 (-3.98-4.28) |
| Sexually transmitted infections excluding HIV | Cote d'Ivoire | 1212650.3 (1019376.5-1453332.2) | 11902.6 (10031.9-14496.4) | 2984535.7 (2495381.3-3624961.8) | 11737.3 (9854.7-14347.8) | -1.18 (-4.71-2.49) |
| Sexually transmitted infections excluding HIV | Croatia | 465228.1 (388760.3-557749.3) | 9010.1 (7537.5-10820.9) | 361719.2 (305490.9-437209.3) | 8958.7 (7460.4-10887.5) | 0.08 (-4.16-4.5) |
| Sexually transmitted infections excluding HIV | Cuba | 1383422.1 (1157976.6-1666677.3) | 11672 (9715.1-14067.7) | 1344030.6 (1124594-1629113.8) | 11618.1 (9627.3-14032.4) | 0.92 (-3.19-5.22) |
| Sexually transmitted infections excluding HIV | Cyprus | 26546.3 (21405.5-32948) | 3237.1 (2613-4019.8) | 51474.7 (41687.7-65353) | 3209.6 (2631.4-3984.4) | 0.04 (-3.63-3.85) |
| Sexually transmitted infections excluding HIV | Czech Republic | 909842.8 (755945.5-1098106.8) | 8617.9 (7196.5-10313.3) | 867401.4 (727220.8-1053615.5) | 8516.6 (7088.9-10253.6) | 0.39 (-3.87-4.83) |
| Sexually transmitted infections excluding HIV | Democratic Republic of the Congo | 3587938.2 (3063864.9-4198686.5) | 11464.7 (9871.1-13602.9) | 9181869.3 (7762833.8-10840953.7) | 11431.1 (9634.1-13704.8) | -1.3 (-4.94-2.49) |
| Sexually transmitted infections excluding HIV | Denmark | 195754.4 (160138.4-243793.4) | 3459.5 (2831.1-4265.1) | 204036.1 (168632.5-252775.8) | 3422 (2802-4209.7) | -0.26 (-3.73-3.34) |
| Sexually transmitted infections excluding HIV | Djibouti | 58248 (48829.1-69685) | 15961.6 (13316.2-19463.7) | 211596.2 (175881.8-258736.7) | 15879.4 (13266.8-19276.5) | -0.19 (-3.94-3.7) |
| Sexually transmitted infections excluding HIV | Dominica | 7893.2 (6670.2-9484.5) | 11874.3 (9931.5-14336.8) | 8380.4 (6995-10147) | 11861.6 (9840.1-14362.4) | -0.21 (-4.16-3.9) |
| Sexually transmitted infections excluding HIV | Dominican Republic | 806073.2 (676846.4-967166.1) | 11979.8 (10045.6-14588.3) | 1391989.4 (1149971.6-1677454) | 11865.4 (9811.1-14272.9) | -0.71 (-4.46-3.19) |
| Sexually transmitted infections excluding HIV | Ecuador | 702772.6 (581486.6-849713.5) | 7785.7 (6429.2-9522.8) | 1438280.8 (1189458.5-1753271.1) | 7713 (6397.5-9392.1) | 0.54 (-3.22-4.44) |
| Sexually transmitted infections excluding HIV | Egypt | 4424466.4 (3721999-5301848.7) | 8743.9 (7402.2-10529.2) | 8670879 (7304046.5-10414833.4) | 8320.1 (7013.5-9978.5) | 0 (-4.33-4.53) |
| Sexually transmitted infections excluding HIV | El Salvador | 540814.6 (452409-654702.7) | 11900.2 (9908.9-14603.8) | 771798.4 (641534.2-934019.5) | 11810 (9789.3-14408.2) | -0.23 (-4.16-3.86) |
| Sexually transmitted infections excluding HIV | Equatorial Guinea | 39710.8 (34326.7-46528) | 11671.2 (10089.4-13769.8) | 172023.6 (146356.5-203775.4) | 11568.6 (9820.3-13819.3) | 0.57 (-2.97-4.24) |
| Sexually transmitted infections excluding HIV | Eritrea | 436033.5 (367824.2-518544.5) | 15840.3 (13287.3-19256.7) | 982981.8 (812155.3-1186654.8) | 15568.6 (12996.8-18950.6) | -1.3 (-5.1-2.65) |
| Sexually transmitted infections excluding HIV | Estonia | 145449.3 (121670.4-175852.7) | 9104.7 (7607.1-11024.4) | 109970.3 (92479.4-134410.9) | 8739.9 (7318.2-10653.7) | 0.65 (-3.26-4.73) |
| Sexually transmitted infections excluding HIV | Ethiopia | 6179747 (5140403.4-7428650.7) | 16153.6 (13524.6-19781.2) | 14584110.3 (11999311-17758840.9) | 15427.9 (12753.6-19053.3) | -1.38 (-5.11-2.5) |
| Sexually transmitted infections excluding HIV | Federated States of Micronesia | 10417.7 (8693.6-12502.1) | 11953.8 (9995.4-14431.9) | 12165.1 (10141.8-14654.2) | 11646.8 (9660.1-14122.6) | 0.99 (-3.14-5.3) |
| Sexually transmitted infections excluding HIV | Fiji | 109046.4 (94309.4-127390.1) | 14296.6 (12384.8-16746.8) | 123472.9 (103768-148931.4) | 13050.2 (10979.6-15719) | 0.08 (-4.15-4.5) |
| Sexually transmitted infections excluding HIV | Finland | 188182.3 (151895.3-234561.7) | 3366.6 (2743.1-4135.5) | 183839.9 (151487.7-228520.8) | 3298 (2681.3-4095.3) | -0.17 (-3.68-3.47) |
| Sexually transmitted infections excluding HIV | France | 2123521.7 (1744714.7-2638517.9) | 3436.8 (2812.5-4269.7) | 2229922.8 (1848976.2-2850893.6) | 3379.5 (2728.4-4215.5) | 0.07 (-3.21-3.47) |
| Sexually transmitted infections excluding HIV | Gabon | 98713.4 (84901.8-115493.4) | 11664.5 (9996.7-13766.6) | 201935 (170700.8-239882.2) | 11363.3 (9635.7-13684.3) | -0.52 (-4.04-3.14) |
| Sexually transmitted infections excluding HIV | Georgia | 666981.6 (556531.6-803998.3) | 11808.9 (9882.4-14309.4) | 400888.6 (337752-481662.6) | 11560.8 (9614.9-13913.8) | 0.67 (-3.52-5.04) |
| Sexually transmitted infections excluding HIV | Germany | 3192913.4 (2628630.4-3955834.5) | 3585 (2965.2-4428.9) | 3088248.5 (2559019.1-3815818.2) | 3527.1 (2897-4328.6) | -0.12 (-3.64-3.52) |
| Sexually transmitted infections excluding HIV | Ghana | 1779813.4 (1508465.1-2134886.9) | 13938.3 (11872.7-16728.1) | 4613338.3 (3863362.6-5520592.5) | 13845.9 (11664.4-16650.7) | -0.48 (-4.16-3.34) |
| Sexually transmitted infections excluding HIV | Greece | 429243.1 (354328-532003.2) | 3917.7 (3192.2-4867) | 410752.8 (336113.4-511841.8) | 3898.9 (3193.9-4792.5) | 0.58 (-2.98-4.27) |
| Sexually transmitted infections excluding HIV | Greenland | 3405.5 (2710.2-4240.1) | 5177 (4165-6342.7) | 3202.1 (2578.4-3996.4) | 5334.7 (4320.4-6623) | 0.04 (-3.4-3.61) |
| Sexually transmitted infections excluding HIV | Grenada | 8888.5 (7487.3-10642.2) | 11940.3 (9987-14373.6) | 13110.5 (11016.5-15868) | 11859.1 (9922.6-14452.7) | -0.4 (-4.39-3.75) |
| Sexually transmitted infections excluding HIV | Guam | 17180.6 (14160.6-20923.1) | 11435.7 (9498.3-13904.5) | 18082.1 (15092.9-21927.6) | 11449 (9507.3-13969.3) | 0.1 (-4.06-4.44) |
| Sexually transmitted infections excluding HIV | Guatemala | 786431 (662675.5-932464.9) | 12064.6 (10179.3-14516.5) | 1878239.4 (1552038.7-2244015.8) | 11832.3 (9770.3-14361.2) | -0.51 (-4.52-3.67) |
| Sexually transmitted infections excluding HIV | Guinea | 570619.5 (477901.9-680406.8) | 11960.2 (9955.2-14363.5) | 1328900.7 (1111632.3-1598041) | 11895.4 (9932-14509.9) | -0.32 (-3.93-3.42) |
| Sexually transmitted infections excluding HIV | Guinea-Bissau | 93325.6 (77707.3-112239.6) | 11772.9 (9795.3-14293.1) | 213524.5 (177080.8-259213.7) | 11731 (9796-14299.7) | 0.28 (-3.3-3.99) |
| Sexually transmitted infections excluding HIV | Guyana | 89060.7 (73789.8-107364.1) | 11950.3 (9908-14484) | 94030.7 (78352.6-114043.5) | 11853.2 (9868.4-14427.5) | -0.3 (-4.05-3.59) |
| Sexually transmitted infections excluding HIV | Haiti | 629130.8 (531270.4-755818.5) | 11358.5 (9526.9-13652.9) | 1469466.5 (1219602.3-1792080.5) | 11125.4 (9308.4-13587.6) | -0.6 (-4.18-3.11) |
| Sexually transmitted infections excluding HIV | Honduras | 451209.2 (373463.6-538390.4) | 12131.9 (10056.5-14765.9) | 1225117.4 (1021002.9-1482467.9) | 11998.9 (10022.3-14735.9) | -0.35 (-4.55-4.04) |
| Sexually transmitted infections excluding HIV | Hungary | 912857.2 (772131-1096133.6) | 8644.6 (7181.7-10351.3) | 795095.7 (666870.1-962822.7) | 8497.4 (7111.6-10238.7) | 0.36 (-3.86-4.77) |
| Sexually transmitted infections excluding HIV | Iceland | 8674 (7013.5-10785.3) | 3269.7 (2648.2-4078.1) | 12126.3 (9886.3-15291.1) | 3289.9 (2682.8-4125.9) | 0.38 (-3.03-3.91) |
| Sexually transmitted infections excluding HIV | India | 49931838.4 (42037330.3-60332041.3) | 6264.7 (5345.8-7528.6) | 94354730.8 (80187521.9-113567790.9) | 6138.2 (5237.8-7378.6) | -0.44 (-4.05-3.31) |
| Sexually transmitted infections excluding HIV | Indonesia | 19541628.3 (16344356.9-24011304.1) | 11079.3 (9288.6-13614.3) | 34113154.1 (28567852.1-42303207.2) | 10883.9 (9120.5-13378.9) | 0.56 (-3.67-4.97) |
| Sexually transmitted infections excluding HIV | Iran | 4137711.6 (3460626.6-5032226.6) | 8951.9 (7505.3-10983.9) | 8715344.4 (7278616.3-10800985.4) | 8696.3 (7336.7-10670) | 0.47 (-3.83-4.96) |
| Sexually transmitted infections excluding HIV | Iraq | 1525575.5 (1299766.1-1795002.3) | 9793.3 (8361.3-11622.9) | 3662099 (3056689.9-4447898.9) | 8634.9 (7233.7-10447.9) | -0.11 (-4.46-4.44) |
| Sexually transmitted infections excluding HIV | Ireland | 116256.1 (94972.6-145363) | 3288.3 (2677.7-4136.5) | 169020.8 (138344-215386.3) | 3258.7 (2643.3-4037.1) | -0.07 (-3.39-3.35) |
| Sexually transmitted infections excluding HIV | Israel | 158379.1 (130358-194212.9) | 3341.9 (2760.5-4117.1) | 308241.1 (254757.7-380400.8) | 3314 (2730.5-4066.3) | 0.11 (-3.29-3.62) |
| Sexually transmitted infections excluding HIV | Italy | 2098213.4 (1710855.9-2639307.8) | 3384 (2724.7-4245.5) | 2114829 (1726397.4-2670398.5) | 3384.1 (2718.5-4244.5) | -0.26 (-3.6-3.19) |
| Sexually transmitted infections excluding HIV | Jamaica | 267413.5 (225374.4-318827.1) | 12173 (10180.6-14792.6) | 372653.9 (310984.2-447685.6) | 11929.7 (9938-14369.2) | -0.1 (-3.89-3.85) |
| Sexually transmitted infections excluding HIV | Japan | 6883314.3 (5696617.9-8547159.8) | 4960.2 (4101.4-6110.6) | 6180017 (5119832.7-7638277.1) | 4987.8 (4105.8-6116.6) | 0.26 (-3.62-4.31) |
| Sexually transmitted infections excluding HIV | Jordan | 173136.3 (146662.8-205818.3) | 5377.5 (4591.6-6434.3) | 706210.8 (605391.3-841928.7) | 5353 (4583.2-6370.1) | -0.02 (-4.11-4.25) |
| Sexually transmitted infections excluding HIV | Kazakhstan | 1978017.9 (1622814.5-2396370.3) | 11794 (9779.9-14356.7) | 2259171.7 (1864115.6-2760169.2) | 11640.2 (9667-14176) | 0.39 (-3.64-4.58) |
| Sexually transmitted infections excluding HIV | Kenya | 2532819.8 (2114448.8-3044283.2) | 14668.9 (12225.6-17923.5) | 6955772.8 (5721562.1-8466048.3) | 14958.7 (12341.7-18342.5) | -1.43 (-5.05-2.32) |
| Sexually transmitted infections excluding HIV | Kiribati | 8239.8 (6804.3-9884.7) | 11918.8 (9891.7-14369.5) | 14074.3 (11583.2-16962.4) | 11682.2 (9643.4-14177.8) | -0.14 (-4.48-4.4) |
| Sexually transmitted infections excluding HIV | Kuwait | 159788.4 (131122-194605) | 8063.7 (6736.1-9780.9) | 464760.1 (382932.6-573432) | 7392.1 (6200.7-8864.6) | -0.27 (-4.48-4.13) |
| Sexually transmitted infections excluding HIV | Kyrgyzstan | 491454.3 (401584-591663.5) | 11750.8 (9785.2-14218.7) | 812777.7 (673639-984187.1) | 11663.6 (9696.5-14150.3) | 0.86 (-3.29-5.19) |
| Sexually transmitted infections excluding HIV | Laos | 386580 (319090.4-468308.7) | 11111.9 (9231.5-13606) | 851843.3 (700831.5-1050266.2) | 10863.7 (9021.7-13283.1) | 0.67 (-3.38-4.9) |
| Sexually transmitted infections excluding HIV | Latvia | 246524.7 (206509.9-299412) | 9106.5 (7631-10992.1) | 153840.6 (130118.2-188651.3) | 8806.5 (7373.5-10688.4) | 0.27 (-3.61-4.31) |
| Sexually transmitted infections excluding HIV | Lebanon | 184060.2 (157305.8-218771.3) | 6574.2 (5604-7888.2) | 405784.6 (334009.3-493364.2) | 6518.6 (5478-7910.3) | 0.32 (-3.65-4.46) |
| Sexually transmitted infections excluding HIV | Lesotho | 210834.4 (179884.8-246909.9) | 16573.3 (14075.8-19549.4) | 309711.7 (265924.5-366800.6) | 16086.2 (13768.3-19101.9) | -0.33 (-3.99-3.46) |
| Sexually transmitted infections excluding HIV | Liberia | 242421.9 (202051.8-287974.1) | 12038.3 (10116.9-14424.6) | 608093.7 (509446.8-734732.1) | 11890.4 (10000.1-14352.9) | -0.48 (-4.09-3.26) |
| Sexually transmitted infections excluding HIV | Libya | 253877.8 (210916.3-301225) | 6962.1 (5823.6-8348.9) | 551343.8 (456899.1-666820.8) | 6700.7 (5604.3-8031.1) | 0.15 (-3.89-4.36) |
| Sexually transmitted infections excluding HIV | Lithuania | 343522.7 (288740.5-415369.6) | 9019.8 (7562.9-10870) | 227483.4 (191998.4-279199.5) | 8825.3 (7434.6-10758.3) | 0.45 (-3.61-4.68) |
| Sexually transmitted infections excluding HIV | Luxembourg | 13786.8 (11227.5-17121.6) | 3158.2 (2589.4-3925.6) | 23398.3 (18900.5-29895.4) | 3171.3 (2562.3-3934.3) | -0.24 (-3.47-3.11) |
| Sexually transmitted infections excluding HIV | Macedonia | 181213.7 (151216.3-220052.8) | 8642.2 (7213.3-10492.3) | 204149.7 (170258-244406.8) | 8619.8 (7229.7-10376.8) | 0.06 (-4.35-4.68) |
| Sexually transmitted infections excluding HIV | Madagascar | 1559787.2 (1328654-1852357.1) | 16201.3 (13738.7-19405.6) | 4101100.4 (3442797.1-4895332.1) | 15997.1 (13458.9-19529.6) | 0.59 (-3.5-4.85) |
| Sexually transmitted infections excluding HIV | Malawi | 1229744.7 (1049194.6-1467273.4) | 15234.3 (12926.6-18425.7) | 2607732.4 (2207643.8-3072267.6) | 15101.8 (12791.4-18093.9) | -0.93 (-4.53-2.81) |
| Sexually transmitted infections excluding HIV | Malaysia | 1853978.1 (1539073.6-2262752.9) | 10784.9 (8959.2-13077.2) | 3847477.7 (3185791.7-4684523.1) | 10675.7 (8900-12923) | -0.14 (-4.07-3.94) |
| Sexually transmitted infections excluding HIV | Maldives | 19104.2 (15857-22985.2) | 10983.4 (9082.2-13393.1) | 82845.9 (66800.8-103113.8) | 11440.8 (9471.4-13979.1) | 0.08 (-4.31-4.67) |
| Sexually transmitted infections excluding HIV | Mali | 937834.5 (785937.3-1112418) | 13826 (11545.2-16671.3) | 2526811.2 (2098091-3016854.8) | 13510.5 (11275.1-16499) | -0.76 (-4.46-3.08) |
| Sexually transmitted infections excluding HIV | Malta | 12965.1 (10518-16317.5) | 3209.8 (2624.6-3980.3) | 15231.5 (12298.1-19235) | 3264.1 (2639.8-4084.1) | 0.67 (-2.67-4.13) |
| Sexually transmitted infections excluding HIV | Marshall Islands | 3556.3 (2913.7-4326.4) | 9781.8 (8041.9-11974) | 5650.5 (4638.7-6985.5) | 9586.3 (7898.9-11898.3) | 0.87 (-3.11-5.02) |
| Sexually transmitted infections excluding HIV | Mauritania | 201945.8 (168271.9-241757.3) | 12020.1 (9968-14471.9) | 444780 (370401.8-531215.5) | 11814.2 (9767-14315.4) | -0.14 (-4.38-4.29) |
| Sexually transmitted infections excluding HIV | Mauritius | 128584.6 (106560.4-156610.5) | 10791.8 (9013.4-13056.6) | 144422.2 (120999.8-177201.1) | 10507.9 (8751.3-12905.3) | 0.69 (-3.22-4.77) |
| Sexually transmitted infections excluding HIV | Mexico | 9497379.6 (7820713.4-11529325.7) | 12618.3 (10456.4-15534) | 17182019.3 (14174835.4-21299720.4) | 12385.2 (10210.8-15314.1) | 0.09 (-3.94-4.29) |
| Sexually transmitted infections excluding HIV | Moldova | 416543 (345362.8-509331.4) | 9139.4 (7600.9-11076) | 345347.1 (289052.8-426283.3) | 8914.9 (7402.3-10803.7) | 0.22 (-3.64-4.23) |
| Sexually transmitted infections excluding HIV | Monaco | 1063.9 (873.4-1361.8) | 3235.4 (2643-4037.6) | 1190.4 (987.9-1513.6) | 3228.1 (2635.1-4029.7) | -0.03 (-3.59-3.66) |
| Sexually transmitted infections excluding HIV | Mongolia | 234479.8 (190729-283294) | 11887.6 (9922.2-14513.7) | 407652.1 (339564.9-497924) | 11803.9 (9897.9-14265.9) | 0.37 (-4.08-5.03) |
| Sexually transmitted infections excluding HIV | Montenegro | 57275.2 (47457.5-68329.2) | 8725.8 (7239.1-10422.5) | 53833.2 (45432-65021.7) | 8649 (7166.2-10370.5) | 0 (-4.31-4.51) |
| Sexually transmitted infections excluding HIV | Morocco | 2559437.9 (2138494.6-3080053.6) | 10802.5 (9123.2-13108.5) | 3801754 (3142746.7-4676752.8) | 9660.6 (7988.5-11867.6) | -0.27 (-4.32-3.94) |
| Sexually transmitted infections excluding HIV | Mozambique | 2085613.3 (1782100.8-2444347.2) | 19264.8 (16423.5-22778.6) | 4582416.9 (3872001.9-5416475.7) | 17887.2 (15130.8-21549) | -0.21 (-3.9-3.63) |
| Sexually transmitted infections excluding HIV | Myanmar | 4313799.5 (3595414.2-5179772.1) | 11160.7 (9312.1-13512.2) | 6349868.5 (5293171-7735396.7) | 10838.4 (9036.9-13235.7) | -0.3 (-4.05-3.6) |
| Sexually transmitted infections excluding HIV | Namibia | 199046.1 (170909.3-234400.9) | 16088.7 (13830.2-18960.2) | 400301 (340734-475355.9) | 16011.9 (13600-19027.3) | -0.75 (-4.39-3.03) |
| Sexually transmitted infections excluding HIV | Nauru | 1147.3 (950.3-1399.7) | 12204.5 (10124.9-14889.6) | 1298.3 (1070.1-1562) | 11952.6 (9908.4-14520.8) | 1.02 (-3.14-5.35) |
| Sexually transmitted infections excluding HIV | Nepal | 952684.6 (809437-1124107.1) | 5722.3 (4838.5-6817.8) | 1762446.8 (1496312.9-2112844.4) | 5518.7 (4672.8-6641.7) | 0.14 (-3.56-3.98) |
| Sexually transmitted infections excluding HIV | Netherlands | 548539.3 (449231-675181) | 3261 (2688.3-4012.8) | 556427.2 (461090-694677) | 3179 (2616.1-3943.5) | -0.06 (-3.48-3.47) |
| Sexually transmitted infections excluding HIV | New Zealand | 190511.7 (157469.5-234277) | 5252.7 (4349.3-6449.2) | 284038.2 (236192.6-345543.7) | 5266.1 (4378.8-6436.7) | 0.13 (-3.66-4.07) |
| Sexually transmitted infections excluding HIV | Nicaragua | 386564.5 (325013.2-461807.6) | 12396.2 (10352.4-14898.3) | 843239.7 (696559.2-1014140.8) | 11994.6 (10022.6-14486) | 0.45 (-3.69-4.78) |
| Sexually transmitted infections excluding HIV | Niger | 731822.6 (610205.9-872931.1) | 12028.1 (10074.8-14484.8) | 2151269.8 (1804059.5-2578977.6) | 11848.7 (9912.6-14348.5) | -1.21 (-4.97-2.7) |
| Sexually transmitted infections excluding HIV | Nigeria | 9313559.1 (7740714.1-11136563.7) | 12582.5 (10437.3-15302.8) | 24992437.1 (20606409-30190458.9) | 13126 (10772.5-16047.6) | -0.41 (-4.07-3.4) |
| Sexually transmitted infections excluding HIV | Niue | 243.1 (203-292.3) | 12059.2 (9900.3-14626.4) | 192.4 (160.9-233.7) | 11782.9 (9790.5-14296.6) | 0.96 (-3.19-5.28) |
| Sexually transmitted infections excluding HIV | North Korea | 2149469.2 (1762277.7-2640122.4) | 10034.8 (8217.9-12370.9) | 2977120.4 (2473876.4-3709273.2) | 9984.9 (8254-12372.4) | 0.61 (-3.52-4.92) |
| Sexually transmitted infections excluding HIV | Northern Mariana Islands | 6586.4 (5408-8049.7) | 11608.2 (9709.1-14037.4) | 5716 (4817.4-6892.2) | 11360.8 (9481.8-13765.3) | 0.11 (-4.12-4.52) |
| Sexually transmitted infections excluding HIV | Norway | 167661.4 (136726.2-207755.3) | 3693 (3007-4578.8) | 213502.7 (173805.3-268151.7) | 3703.7 (2996-4620.8) | -0.12 (-3.57-3.45) |
| Sexually transmitted infections excluding HIV | Oman | 154561.5 (126979.2-186673.1) | 8129.3 (6774-9864.9) | 483555.9 (392322.1-594212.5) | 7956.5 (6621.2-9610.2) | 0.21 (-3.9-4.5) |
| Sexually transmitted infections excluding HIV | Pakistan | 5452928.8 (4505417-6679714.5) | 6199.2 (5102.5-7630.1) | 13734093.9 (11279104.1-16851447.3) | 6051.4 (4976.6-7497.8) | 1.1 (-2.86-5.23) |
| Sexually transmitted infections excluding HIV | Palau | 1933.2 (1589.4-2364) | 11874.3 (9823.3-14546.9) | 2166.5 (1816.6-2710.4) | 10857.8 (9077-13269.2) | 0.87 (-3.28-5.19) |
| Sexually transmitted infections excluding HIV | Palestine | 130247.4 (108412.6-153958.7) | 8136.8 (6829.9-9751.3) | 401935.2 (334716.8-480576) | 8027.2 (6699.7-9703.7) | 0.16 (-4.12-4.63) |
| Sexually transmitted infections excluding HIV | Panama | 269320.3 (223823.4-321562) | 11693.5 (9718.1-14138.1) | 509626.5 (424412.8-622267.5) | 11644.6 (9692.8-14265.2) | 0.21 (-3.6-4.17) |
| Sexually transmitted infections excluding HIV | Papua New Guinea | 609529.9 (515784.2-712705.6) | 15858.2 (13615.2-18629.4) | 1567115.7 (1333767.9-1842506.1) | 15058.8 (12943-17706.4) | 0.53 (-3.47-4.7) |
| Sexually transmitted infections excluding HIV | Paraguay | 426295 (356316.1-510497.2) | 11928 (9964.2-14444.5) | 903142.1 (748755.4-1079980.1) | 11830.2 (9828.8-14197.2) | 0.46 (-3.59-4.68) |
| Sexually transmitted infections excluding HIV | Peru | 1711702 (1429249.8-2034383.7) | 8697.1 (7268.5-10523.7) | 3328120.3 (2782676.5-4032152.6) | 8557 (7162.6-10371.1) | 0.01 (-3.81-3.99) |
| Sexually transmitted infections excluding HIV | Philippines | 6503250 (5427169.2-8038466.1) | 11475.9 (9567.7-14251.6) | 13248197.6 (11080080.9-16343659.6) | 11311.2 (9458.8-13970.8) | 1.16 (-2.86-5.36) |
| Sexually transmitted infections excluding HIV | Poland | 3549700.5 (2959070-4365794.6) | 8926.6 (7434.9-10832.6) | 3477953.8 (2904358.5-4276645.4) | 8736.9 (7270.3-10682.3) | 0.18 (-4.01-4.55) |
| Sexually transmitted infections excluding HIV | Portugal | 358611.7 (296904.9-447980.8) | 3403.8 (2796-4242.5) | 375846.2 (308682.1-474227.8) | 3404.6 (2781.4-4214.5) | -0.73 (-3.96-2.61) |
| Sexually transmitted infections excluding HIV | Puerto Rico | 428169.4 (357420.2-521363.9) | 11720.6 (9791.7-14311.7) | 377679.2 (320036.6-459767.6) | 11701.8 (9760.9-14307.1) | -0.9 (-4.73-3.08) |
| Sexually transmitted infections excluding HIV | Qatar | 48909.9 (39677-60015.6) | 8506.2 (7144.2-10263.5) | 382462.9 (307126.8-481051.9) | 8387.6 (7075-10077.5) | -0.23 (-4.5-4.22) |
| Sexually transmitted infections excluding HIV | Romania | 2057413.7 (1722600.4-2483592.9) | 8745.1 (7253-10560.1) | 1565844.7 (1318237.9-1892263.7) | 8644.8 (7176.4-10360.1) | 0.28 (-3.82-4.56) |
| Sexually transmitted infections excluding HIV | Russian Federation | 14642319.2 (12264742.3-18033271.7) | 9161.8 (7651.9-11184.3) | 13339637.2 (11248725.4-16402732.9) | 9020.4 (7509-11036.4) | 1.19 (-2.71-5.26) |
| Sexually transmitted infections excluding HIV | Rwanda | 903616.1 (758150.7-1074681.8) | 15802.6 (13189.4-19128.2) | 1953777.2 (1625900.4-2368119.7) | 15515.5 (12931.4-19104.3) | -0.97 (-4.64-2.84) |
| Sexually transmitted infections excluding HIV | Saint Kitts and Nevis | 4583.6 (3818.3-5526) | 12059.7 (10037.6-14788.2) | 8032.8 (6679.3-9855) | 11911.3 (9918.7-14489.2) | -0.03 (-3.79-3.88) |
| Sexually transmitted infections excluding HIV | Saint Lucia | 14770.9 (12373.5-17712.1) | 11975.1 (9997.6-14543.8) | 23500.8 (19717.6-28618.9) | 11859.3 (9920-14429.7) | -0.41 (-4.16-3.49) |
| Sexually transmitted infections excluding HIV | Saint Vincent and the Grenadines | 11787.5 (9960.5-14034.8) | 11943.6 (10071.9-14521.8) | 13947.4 (11589.6-16815.3) | 11807.1 (9797.2-14301.9) | -0.51 (-4.33-3.47) |
| Sexually transmitted infections excluding HIV | Samoa | 18918.9 (15982.9-22576.8) | 13280.2 (11166.9-16087.8) | 25466.7 (21657.1-30767.2) | 13121.1 (11023.4-15947.9) | 0.97 (-3.28-5.41) |
| Sexually transmitted infections excluding HIV | San Marino | 846.8 (694.6-1056.2) | 3256.7 (2663.2-4066.8) | 1072.7 (889.9-1375.9) | 3196.4 (2620.3-4006.6) | -0.07 (-3.62-3.61) |
| Sexually transmitted infections excluding HIV | Sao Tome and Principe | 10512.3 (8878.1-12448.3) | 11592.7 (9769.1-14083.5) | 24385.4 (20371.3-29297.1) | 11604.2 (9698.5-14040.7) | -0.49 (-4.64-3.84) |
| Sexually transmitted infections excluding HIV | Saudi Arabia | 1584364.1 (1319112.3-1910309.6) | 10805.2 (8983.7-13012.8) | 5101844.5 (4175200.1-6314411.4) | 10005.5 (8370.7-12168.6) | -0.03 (-4.22-4.34) |
| Sexually transmitted infections excluding HIV | Senegal | 614354.8 (507659.2-748259.4) | 10587 (8757.7-13148.2) | 1415553.3 (1163012.4-1719190.6) | 10208 (8346.5-12690.9) | -0.7 (-4.3-3.04) |
| Sexually transmitted infections excluding HIV | Serbia | 870717 (731131.4-1052609.2) | 8721.8 (7288.4-10522.6) | 783692 (657105.4-943749.6) | 8659.6 (7258.9-10375.6) | 0 (-4.35-4.55) |
| Sexually transmitted infections excluding HIV | Seychelles | 7633.3 (6325.2-9086.5) | 10730.4 (8883.2-12834.6) | 12384.5 (10381.8-15336.6) | 10763 (9056.3-13150.6) | -0.26 (-4.28-3.94) |
| Sexually transmitted infections excluding HIV | Sierra Leone | 413729.1 (341888.2-499162.1) | 11707.5 (9738-14196.1) | 942957 (788055.1-1135102.6) | 11679.9 (9811.4-14152.8) | -0.21 (-3.84-3.55) |
| Sexually transmitted infections excluding HIV | Singapore | 181978.8 (149555.3-221071.4) | 4914.4 (4072.8-5930.3) | 338824.3 (278440.9-420394.3) | 4908.1 (4047.5-5964.4) | -0.46 (-3.96-3.18) |
| Sexually transmitted infections excluding HIV | Slovakia | 470940.8 (390020.6-569716.2) | 8637.1 (7145.6-10347.6) | 480074.9 (404408.6-585633.5) | 8640.6 (7278.3-10325.1) | 0.05 (-4.36-4.66) |
| Sexually transmitted infections excluding HIV | Slovenia | 166951.6 (140481.7-200269.1) | 7897.6 (6659.1-9480.1) | 152163.8 (128245.7-184459.8) | 7755.2 (6498.8-9345.5) | -0.2 (-4.32-4.1) |
| Sexually transmitted infections excluding HIV | Solomon Islands | 30345.3 (25363.3-36491.4) | 11161.1 (9294.1-13531) | 70292.6 (58259.2-84213.1) | 10878.3 (9060-13095.2) | 0.94 (-3.15-5.19) |
| Sexually transmitted infections excluding HIV | Somalia | 1156624.2 (985353.5-1374298) | 18108.6 (15405.4-21644.1) | 3092227.9 (2639056.4-3687164.2) | 17580.4 (14833.5-21127) | -0.55 (-4.47-3.53) |
| Sexually transmitted infections excluding HIV | South Africa | 8277146.6 (7037245.2-9858588.9) | 22785.7 (19679.8-27218) | 13371361.9 (11427445.2-16129013.5) | 21216.8 (18177.3-25310.5) | -0.52 (-4.33-3.44) |
| Sexually transmitted infections excluding HIV | South Korea | 2591321.2 (2165915.6-3096687.5) | 5183.1 (4362.1-6197.5) | 2898503.3 (2459031.8-3558233.4) | 5043.7 (4235.5-6147.4) | 0.02 (-3.88-4.08) |
| Sexually transmitted infections excluding HIV | South Sudan | 798834.8 (679402.8-943349.7) | 16680 (14141.4-19964.7) | 1300022.1 (1097055.2-1548904.3) | 16311.2 (13692.7-19704.1) | -0.09 (-3.83-3.8) |
| Sexually transmitted infections excluding HIV | Spain | 1354282 (1115093.5-1676450) | 3352.5 (2739.9-4166) | 1642344.8 (1351681.3-2106005.7) | 3376.2 (2766-4176.5) | -0.6 (-3.89-2.82) |
| Sexually transmitted infections excluding HIV | Sri Lanka | 2038764.1 (1695756.1-2460002) | 11521.6 (9595.3-13842.7) | 2552867 (2147102-3101531.8) | 11159.2 (9304.1-13568.1) | 0.01 (-4.41-4.63) |
| Sexually transmitted infections excluding HIV | Sudan | 1653892.9 (1389021-1955221.4) | 9804.2 (8200.6-11676) | 4034673.5 (3395470.7-4769758.5) | 9629.6 (8143.3-11452) | 0.13 (-3.67-4.08) |
| Sexually transmitted infections excluding HIV | Suriname | 44872 (37387.4-53552.9) | 11983.1 (9940.3-14365.8) | 70684.2 (58801.6-86804.1) | 11872 (9839.1-14521.9) | -0.09 (-3.89-3.85) |
| Sexually transmitted infections excluding HIV | Swaziland | 110098.8 (94306.6-129123.6) | 16573.4 (14225.8-19601.8) | 191168.2 (162581.3-226599.3) | 16169 (13797.4-19256.7) | -0.24 (-3.91-3.57) |
| Sexually transmitted infections excluding HIV | Sweden | 350255.1 (284929.6-436930.7) | 3854.6 (3145.3-4772.1) | 407309.3 (334082-507860.2) | 3866.7 (3150.4-4777.2) | -0.15 (-3.64-3.47) |
| Sexually transmitted infections excluding HIV | Switzerland | 258582.8 (209521.3-324213.9) | 3308.5 (2694.1-4101.8) | 315577.5 (260233.5-397627.5) | 3274.2 (2671.4-4061.8) | -0.79 (-4.08-2.62) |
| Sexually transmitted infections excluding HIV | Syria | 794319.4 (661706.2-949157.8) | 7836.1 (6566.2-9448.6) | 1037520.8 (879311.4-1249723.1) | 7523.4 (6307.6-9005.6) | 0 (-4.28-4.47) |
| Sexually transmitted infections excluding HIV | Taiwan | 2438193.5 (2024084.4-2987064.4) | 10782.9 (8990.8-13036.6) | 2692076.2 (2216215.1-3355887.1) | 10420 (8528.9-12895.3) | 0.43 (-3.63-4.67) |
| Sexually transmitted infections excluding HIV | Tajikistan | 573451 (461979.2-697857.6) | 12150.2 (10087.3-14798) | 1236643.6 (1025356.4-1486206) | 11992.2 (9995.6-14349.7) | 0.14 (-4.11-4.59) |
| Sexually transmitted infections excluding HIV | Tanzania | 3950676.9 (3349578.4-4715058.6) | 19494.4 (16472.4-23656.2) | 9250378.9 (7716511.8-11196800.5) | 18300.6 (15339.6-22392.9) | -1.24 (-4.97-2.64) |
| Sexually transmitted infections excluding HIV | Thailand | 7123259.9 (5982682.4-8542542.8) | 11612.8 (9713.4-13984.4) | 7843971.5 (6562205.2-9634844.7) | 11100.6 (9201.3-13568.4) | -0.72 (-4.45-3.15) |
| Sexually transmitted infections excluding HIV | The Bahamas | 32392.8 (26695-39075.2) | 11943.9 (9967.2-14526.5) | 50867.2 (42064.5-61731) | 11825.7 (9764.9-14344.5) | -0.3 (-4.02-3.56) |
| Sexually transmitted infections excluding HIV | The Gambia | 88484.7 (73204.9-106573.4) | 11107.9 (9195.1-13477.1) | 234375.2 (194374.3-284156.7) | 11030.9 (9135.7-13599.6) | 0.14 (-3.42-3.83) |
| Sexually transmitted infections excluding HIV | Timor-Leste | 81786.9 (67428.5-98920.2) | 11319.9 (9439.1-13540) | 136276.3 (115011.5-163107.1) | 10823.2 (9032.2-13196) | -0.2 (-4.01-3.76) |
| Sexually transmitted infections excluding HIV | Togo | 307667.8 (257555.1-370701.4) | 10646.8 (8912.8-12917.8) | 828513.9 (693167.6-999767.1) | 10668.4 (8954-12897.2) | -0.96 (-4.47-2.68) |
| Sexually transmitted infections excluding HIV | Tokelau | 166.5 (138.8-199.4) | 12413.5 (10358.5-15004.8) | 154.5 (128.9-187.1) | 11723 (9720-14293.6) | 0.84 (-3.31-5.17) |
| Sexually transmitted infections excluding HIV | Tonga | 10246.7 (8631.8-12323) | 12664.7 (10622-15249.8) | 11816.7 (9945.1-14223.5) | 12463 (10483.5-15155.5) | 1.02 (-3.16-5.37) |
| Sexually transmitted infections excluding HIV | Trinidad and Tobago | 141265.5 (117924-171216.8) | 11757.7 (9845-14420.4) | 174775.7 (143445.3-212795.7) | 11711.5 (9632.5-14265.8) | -0.02 (-3.72-3.83) |
| Sexually transmitted infections excluding HIV | Tunisia | 682962.4 (581412.9-804982.6) | 8736.3 (7399.7-10472.2) | 1051424.4 (881290.5-1284113.6) | 8297.7 (6994.2-10009.3) | 0.28 (-3.98-4.72) |
| Sexually transmitted infections excluding HIV | Turkey | 4873848.1 (4130874-5732149.8) | 8605.9 (7347.4-10137.2) | 6993193.2 (5909000.8-8484615.7) | 7737.4 (6516.5-9352.9) | -0.04 (-4.32-4.44) |
| Sexually transmitted infections excluding HIV | Turkmenistan | 409890.2 (332159.8-495847.4) | 11911.8 (9975.5-14338.8) | 616140.8 (511693.9-745998.5) | 11406.3 (9495.9-13812.4) | -0.39 (-4.59-4) |
| Sexually transmitted infections excluding HIV | Tuvalu | 1126 (925.1-1378.9) | 12658.6 (10415.4-15471.6) | 1405.7 (1170-1731) | 11431 (9492.1-14122.4) | 0.84 (-3.32-5.17) |
| Sexually transmitted infections excluding HIV | Uganda | 2210716.2 (1907278.4-2572604) | 15760.4 (13609.7-18438.4) | 5604448.6 (4863016.5-6561459.2) | 15129.4 (12980.2-17937.9) | -0.66 (-4.3-3.11) |
| Sexually transmitted infections excluding HIV | Ukraine | 4931830.4 (4114030.1-6023127.7) | 9202.1 (7638.6-11205.3) | 4039104 (3413903.5-5000409.6) | 8970.3 (7481.8-10987.3) | 0.33 (-3.48-4.29) |
| Sexually transmitted infections excluding HIV | United Arab Emirates | 187695.7 (152777.3-230757.9) | 8153.8 (6844.7-9881.4) | 1186344.7 (955008.3-1530609.1) | 8041.7 (6714.5-9938.5) | 0.15 (-4.02-4.5) |
| Sexually transmitted infections excluding HIV | United Kingdom | 1944537.5 (1577276.6-2450271.6) | 3183.2 (2564.7-3993.3) | 2270686.8 (1843615.9-2898552) | 3176.8 (2566.1-3958.1) | 0 (-3.29-3.4) |
| Sexually transmitted infections excluding HIV | United States | 16974628.6 (13574300.6-21422991.3) | 6087.6 (4909.4-7664.6) | 20333136.5 (16543404.2-25761358.5) | 5973.8 (4790.3-7579.8) | -0.15 (-3.67-3.5) |
| Sexually transmitted infections excluding HIV | Uruguay | 151518.5 (128538.4-181853.5) | 4932.2 (4175.5-5937.3) | 172131.4 (145748.8-209019.2) | 4910 (4144.9-5949.2) | 0.16 (-3.27-3.71) |
| Sexually transmitted infections excluding HIV | Uzbekistan | 2287529.7 (1874223.6-2745630.5) | 11903.2 (9977.7-14270.6) | 4224372.9 (3505652-5123190) | 11565.4 (9644.7-13893.2) | 0.53 (-3.58-4.81) |
| Sexually transmitted infections excluding HIV | Vanuatu | 17690.5 (14827.4-21112) | 13330.4 (11163.9-16039.9) | 37786.5 (31678.7-45303.2) | 12638.8 (10548.6-15258.3) | 0.92 (-3.27-5.3) |
| Sexually transmitted infections excluding HIV | Venezuela | 2120814.9 (1750556.9-2558429.1) | 11860.2 (9799.4-14519.4) | 3267637.1 (2720489.3-3998028.4) | 11869.5 (9814.9-14431.5) | 0.25 (-3.64-4.3) |
| Sexually transmitted infections excluding HIV | Vietnam | 5984642.9 (4977488.6-7231937.1) | 9631.7 (8015.7-11672.1) | 10707104.4 (8860041.2-13160797.7) | 9715 (8112.8-11861.1) | 0.32 (-3.54-4.34) |
| Sexually transmitted infections excluding HIV | Yemen | 803277.6 (670581.8-964884.9) | 8014.7 (6661.1-9609) | 2420523.7 (2040183.7-2904349.7) | 7822.1 (6563.3-9429.6) | 0.51 (-3.57-4.77) |
| Sexually transmitted infections excluding HIV | Zambia | 1045620.8 (888921.4-1236224.3) | 16896.5 (14279.3-20576.9) | 2906387.7 (2452959.9-3478745.9) | 16754.6 (14094.3-20284.4) | -0.88 (-4.51-2.9) |
| Sexually transmitted infections excluding HIV | Zimbabwe | 1267843.1 (1081094.2-1481806.3) | 14972.3 (12812.6-17900.4) | 2164735.1 (1827595.6-2541969.5) | 14926.8 (12643.6-17898.1) | -1.3 (-4.89-2.44) |
| Syphilis | Afghanistan | 6838.6 (5073.2-8911.9) | 81.8 (60.4-107) | 26616.6 (19372.1-35196.1) | 87.9 (64.9-115.3) | 0.27 (-3.89-4.6) |
| Syphilis | Albania | 1643.7 (1215.4-2164.7) | 47.8 (35.7-62.6) | 1240.9 (920-1622.7) | 46.3 (34.1-60.6) | -0.11 (-4.53-4.51) |
| Syphilis | Algeria | 19611.2 (14010.6-25957.1) | 80.6 (59-104.7) | 35959.2 (26729.3-47176.4) | 79.6 (59-104) | 0.49 (-3.58-4.74) |
| Syphilis | American Samoa | 120.4 (89.8-155.2) | 233.6 (177.1-300.2) | 113 (87.6-143.6) | 241.5 (186.8-308.9) | 0.96 (-3.19-5.29) |
| Syphilis | Andorra | 52.2 (39.4-67.7) | 89.2 (65.7-114.9) | 66.9 (50.8-84.5) | 86.4 (65-112.7) | -0.13 (-3.69-3.57) |
| Syphilis | Angola | 133246.9 (100607.3-171833.5) | 1415.2 (1071.6-1814.4) | 363676.8 (269149-475422.5) | 1225.8 (918.4-1589.3) | 0.43 (-3.19-4.19) |
| Syphilis | Antigua and Barbuda | 104.5 (77.7-136.6) | 165.3 (127.6-212.2) | 150.8 (114.8-193.9) | 157.3 (118.8-201.1) | -0.21 (-4.12-3.85) |
| Syphilis | Argentina | 67011.3 (50508.7-85607.2) | 208.5 (156.9-267.4) | 94277.1 (70271.4-122070) | 201.3 (150.4-259.6) | -0.06 (-3.49-3.5) |
| Syphilis | Armenia | 2336.1 (1787.8-3019.3) | 65.7 (50.7-84.7) | 1595.8 (1193.8-2087.6) | 53.1 (38.6-71) | 0.92 (-3.39-5.42) |
| Syphilis | Australia | 16052.1 (11850.8-20961.2) | 89.7 (66.6-116.6) | 21622.6 (16377.4-28013.4) | 86.1 (64.4-112.4) | -0.13 (-3.7-3.57) |
| Syphilis | Austria | 5919.1 (4371-7762.1) | 71.3 (52.7-93.1) | 6002.7 (4556-7722.8) | 68.5 (50.8-89.9) | -0.37 (-3.76-3.13) |
| Syphilis | Azerbaijan | 5266.9 (3917-6877.9) | 68.7 (53.1-87.6) | 7053.8 (5344.2-9217.3) | 61.2 (45.6-79.3) | 0 (-4.41-4.62) |
| Syphilis | Bahrain | 614.2 (446-816) | 100.7 (75.5-130.8) | 1880.6 (1413.6-2455.7) | 100.1 (75.4-129.5) | -0.25 (-4.4-4.07) |
| Syphilis | Bangladesh | 320525.3 (235930.3-417030.4) | 316.2 (238.3-408.1) | 443269.1 (329123.7-584990.2) | 251.2 (189.3-329.6) | 0.02 (-4.03-4.25) |
| Syphilis | Barbados | 342.4 (260.6-446.5) | 127.9 (98.4-163.2) | 358.6 (276.3-458) | 123.8 (93.6-158.9) | -0.19 (-4.02-3.8) |
| Syphilis | Belarus | 5793 (4500.2-7471.5) | 53.4 (41.1-69) | 4433.2 (3375-5672.9) | 48.6 (36.8-62.9) | 0.97 (-3.01-5.1) |
| Syphilis | Belgium | 7514.2 (5719.2-9829.5) | 72.5 (54.8-94.9) | 7310.2 (5521.1-9566.7) | 67.9 (50.8-89.8) | -0.14 (-3.44-3.28) |
| Syphilis | Belize | 246.4 (180.2-322) | 142.3 (107.1-183.8) | 643.6 (477-829.2) | 136 (101.8-174.2) | -0.21 (-3.99-3.72) |
| Syphilis | Benin | 13315.1 (9957.7-17366.1) | 318.9 (242.8-410) | 39337.4 (28921.6-51236.1) | 309.3 (233-399.4) | -0.5 (-4.08-3.21) |
| Syphilis | Bermuda | 112 (83.1-146.8) | 166.6 (124.4-215.2) | 88.8 (67.4-113) | 159.4 (118.3-205.3) | -0.48 (-4.39-3.6) |
| Syphilis | Bhutan | 2500.6 (1803.4-3267.9) | 376.1 (280.7-483.3) | 3106.7 (2295.2-4057.6) | 354.1 (265.2-456.6) | 0.16 (-3.49-3.96) |
| Syphilis | Bolivia | 18327.7 (13580.7-23425.7) | 293.9 (222.9-377.7) | 34478.7 (25660.6-45024.3) | 273.4 (205.3-354) | -0.33 (-4.02-3.51) |
| Syphilis | Bosnia and Herzegovina | 2295.8 (1706-2987.7) | 46.3 (34.5-60) | 1434.3 (1073.5-1863.9) | 46.1 (34.1-60.4) | 0.06 (-4.35-4.67) |
| Syphilis | Botswana | 12184.5 (9856.2-14777.5) | 946.8 (759-1152.5) | 17416 (12850.9-22547.6) | 648.4 (484.5-834.1) | -1.25 (-4.85-2.49) |
| Syphilis | Brazil | 229105.7 (163674.1-303695.6) | 145.9 (106.1-192.6) | 435006.1 (330344-546364.6) | 191 (145.6-239.2) | 0.06 (-3.86-4.14) |
| Syphilis | Brunei | 276.6 (204-361.5) | 98 (74.8-126) | 517.6 (382.3-665.6) | 96.6 (72.3-123.1) | 0.32 (-3.39-4.18) |
| Syphilis | Bulgaria | 3580.8 (2687.8-4697.3) | 41.6 (30.7-55) | 2513.4 (1897.9-3303) | 41 (30.6-53.1) | 0.4 (-3.8-4.78) |
| Syphilis | Burkina Faso | 28292.8 (21332.7-36599.4) | 358.1 (269.8-461.3) | 75228.2 (55738.2-97279) | 361.1 (270.9-466.5) | -1.47 (-5.19-2.41) |
| Syphilis | Burundi | 29616.6 (22418.7-38405.7) | 620.4 (475.6-786.1) | 72743.1 (55036.6-93741.6) | 609.6 (469.8-778.1) | -1.89 (-5.64-2.01) |
| Syphilis | Cambodia | 9198.1 (6758-12121.7) | 99.8 (75.2-128.2) | 19759.2 (14536.6-25690.4) | 107.7 (80.1-138.4) | -0.55 (-4.4-3.45) |
| Syphilis | Cameroon | 85291.7 (65532.1-106594.2) | 855.3 (657.6-1074.9) | 203848 (150801.3-267299) | 646.7 (486.4-852) | -0.82 (-4.42-2.91) |
| Syphilis | Canada | 22488.8 (16800.6-29485.2) | 75.2 (56.8-98.2) | 25828.6 (19698.2-34006.4) | 72 (53.7-94.9) | 0.1 (-3.42-3.74) |
| Syphilis | Cape Verde | 1507.3 (1087.6-2005) | 485.9 (361.5-630.9) | 3284.2 (2416-4319.4) | 521.2 (384.4-687.3) | -0.72 (-4.4-3.11) |
| Syphilis | Central African Republic | 35698 (26577.9-45723.2) | 1408.6 (1053.4-1798.9) | 70793.6 (53712.1-91397.1) | 1319.5 (1008.4-1701.8) | -0.86 (-4.39-2.79) |
| Syphilis | Chad | 28703.7 (21720.2-36697.4) | 532.6 (402.7-683.9) | 77355.8 (57775.5-100499.8) | 490.3 (368.8-630.8) | -0.59 (-4.2-3.15) |
| Syphilis | Chile | 22184.1 (16329.5-28760.7) | 155.3 (115.9-201.8) | 29419.5 (21716.6-38729.6) | 150 (110.6-196.7) | 0.38 (-3.11-4.01) |
| Syphilis | China | 1975402.3 (1412844.9-2649972.5) | 149.4 (109.3-198.8) | 2047248.7 (1540693.6-2677248.8) | 146.4 (106.8-193.5) | -0.08 (-4.2-4.23) |
| Syphilis | Colombia | 54392.5 (40019.8-71882.2) | 159.5 (120-211.1) | 80536.2 (59214.6-106891.2) | 151.2 (111.4-199.5) | 0.24 (-3.66-4.29) |
| Syphilis | Comoros | 3421.5 (2608.2-4365.2) | 814.5 (630-1035.1) | 5328.9 (4010.6-6942.5) | 683.3 (517.9-879.1) | -0.17 (-4.66-4.53) |
| Syphilis | Congo | 28034.7 (21008.7-36154.9) | 1266.3 (966.4-1613.9) | 65856.7 (49450.8-84825.8) | 1178.6 (890.2-1516.1) | -0.53 (-4.06-3.13) |
| Syphilis | Cook Islands | 47.2 (35-61.5) | 241.4 (180.4-312.4) | 41 (31.1-51.9) | 261.7 (198.9-334.6) | 1.05 (-3.11-5.39) |
| Syphilis | Costa Rica | 3964 (2927-5205) | 127.8 (96.5-164.7) | 6251.1 (4712.3-8122.1) | 122.9 (92-159.7) | 0.07 (-3.98-4.28) |
| Syphilis | Cote d'Ivoire | 60790.2 (45956.2-79583.6) | 516.3 (395.3-663.3) | 122173.9 (89646-163427.6) | 441.5 (328.9-579.4) | -1.18 (-4.71-2.49) |
| Syphilis | Croatia | 2385 (1798.8-3133.4) | 46.7 (34.9-61.4) | 1741.4 (1328.7-2254.5) | 45 (33.7-58.7) | 0.08 (-4.16-4.5) |
| Syphilis | Cuba | 18284 (13273.5-24237) | 148.3 (110.3-191.3) | 15882.4 (12295.4-19957.2) | 145.9 (112.5-186) | 0.92 (-3.19-5.22) |
| Syphilis | Cyprus | 580.9 (432-759) | 71.7 (53.7-93) | 1006.2 (751.9-1328) | 70.6 (53.3-92.3) | 0.04 (-3.63-3.85) |
| Syphilis | Czech Republic | 4791 (3639.1-6250) | 46.6 (35.1-61.3) | 4335.5 (3244.8-5648.6) | 45.2 (33.4-59) | 0.39 (-3.87-4.83) |
| Syphilis | Democratic Republic of the Congo | 476263.2 (363854.8-603494.2) | 1368.8 (1052.3-1709.8) | 1118225.9 (833226.9-1451135.5) | 1283 (964.1-1658.1) | -1.3 (-4.94-2.49) |
| Syphilis | Denmark | 3606 (2725-4660.3) | 66.6 (50.3-86.9) | 3646.2 (2726-4813) | 64.9 (48.1-86.2) | -0.26 (-3.73-3.34) |
| Syphilis | Djibouti | 2589.6 (1929.5-3386.6) | 635.9 (479.3-812.9) | 8228.5 (6108.3-10764.7) | 602.6 (451-786.4) | -0.19 (-3.94-3.7) |
| Syphilis | Dominica | 170.4 (126.6-222.3) | 233.6 (177.3-298.8) | 158.6 (122-204) | 236.8 (181.7-304.5) | -0.21 (-4.16-3.9) |
| Syphilis | Dominican Republic | 16583.7 (12282.8-21521.7) | 220.9 (167.9-280) | 25848 (19559.9-33628.2) | 218.9 (167.2-282.7) | -0.71 (-4.46-3.19) |
| Syphilis | Ecuador | 25389.7 (18819.1-32939.3) | 250.1 (189.9-320.1) | 45540.8 (34302.7-58912.4) | 239.8 (181.8-310.3) | 0.54 (-3.22-4.44) |
| Syphilis | Egypt | 33886.5 (24589.9-44751.5) | 62.1 (45.3-81.6) | 66567.8 (48769.8-87457) | 62 (45.7-81.2) | 0 (-4.33-4.53) |
| Syphilis | El Salvador | 5295 (3849.9-6968.7) | 105.2 (79.2-137.5) | 6659.3 (4892.6-8737.1) | 98.9 (73.2-128.3) | -0.23 (-4.16-3.86) |
| Syphilis | Equatorial Guinea | 5302.2 (4041.3-6796.2) | 1418.4 (1076.3-1814.9) | 22322.9 (16375.3-29220.4) | 1358.9 (1025.6-1773.7) | 0.57 (-2.97-4.24) |
| Syphilis | Eritrea | 22095.6 (16796-28331.6) | 733.7 (561.2-926.6) | 42959.9 (32189.8-54981.1) | 642.2 (485.2-819.2) | -1.3 (-5.1-2.65) |
| Syphilis | Estonia | 940.2 (729.5-1194.3) | 58.6 (45.4-75.3) | 634.3 (482.8-842.8) | 52.3 (38.6-69.1) | 0.65 (-3.26-4.73) |
| Syphilis | Ethiopia | 492527.1 (361863.4-647770.4) | 1125.4 (830.9-1477.9) | 780273.3 (564852.2-1039004.4) | 734 (545.5-959.2) | -1.38 (-5.11-2.5) |
| Syphilis | Federated States of Micronesia | 282.2 (213.2-369.5) | 282.1 (216.1-364.5) | 305 (231.7-396.3) | 279.6 (213.5-360.9) | 0.99 (-3.14-5.3) |
| Syphilis | Fiji | 3378 (2488.6-4398) | 407.1 (301.9-525.1) | 3190.5 (2399.7-4105.8) | 337.8 (254.1-434.6) | 0.08 (-4.15-4.5) |
| Syphilis | Finland | 3733.2 (2843.2-4932.5) | 71.3 (53.9-94.5) | 3446.2 (2579.9-4487) | 68.8 (50.5-90.4) | -0.17 (-3.68-3.47) |
| Syphilis | France | 44863 (34058.2-58322.9) | 75.7 (57.2-98.9) | 43266 (32175-56095.7) | 72.2 (53.2-95.4) | 0.07 (-3.21-3.47) |
| Syphilis | Gabon | 11877.8 (9305.4-14938.1) | 1331.3 (1048.4-1638.3) | 22139.5 (16584.4-29133.8) | 1186.2 (892.6-1536.3) | -0.52 (-4.04-3.14) |
| Syphilis | Georgia | 4219.7 (3186.7-5443.1) | 74.7 (56.2-95.9) | 2332.9 (1761-2991.8) | 71.1 (52.4-92.4) | 0.67 (-3.52-5.04) |
| Syphilis | Germany | 60918.1 (45857-80386.3) | 70.9 (53.1-93.5) | 54118.1 (41157.9-70041.1) | 68.3 (50.8-89.2) | -0.12 (-3.64-3.52) |
| Syphilis | Ghana | 69564.1 (52991-89199.9) | 494.1 (378.7-631.4) | 168229.6 (125853.9-217316.8) | 473.2 (357.1-604.7) | -0.48 (-4.16-3.34) |
| Syphilis | Greece | 4634.1 (4015.7-5296.3) | 43.9 (37.9-50.6) | 6302.6 (4732-8130) | 68.3 (50.1-88.7) | 0.58 (-2.98-4.27) |
| Syphilis | Greenland | 64.2 (47.6-83.9) | 95.5 (71.8-123) | 51.7 (40.6-66.7) | 92.1 (71.2-119) | 0.04 (-3.4-3.61) |
| Syphilis | Grenada | 188.8 (139.8-247) | 229.7 (173.8-296.7) | 251.5 (186.8-327.8) | 228 (169.7-294.6) | -0.4 (-4.39-3.75) |
| Syphilis | Guam | 358.4 (262.4-465.8) | 226.7 (169.7-292.7) | 347.8 (266.7-451.1) | 227.7 (174.1-296) | 0.1 (-4.06-4.44) |
| Syphilis | Guatemala | 9222.5 (6895.6-11893.7) | 128.7 (97.7-166.4) | 19948.3 (14728.3-26412.9) | 118.4 (89.6-154.6) | -0.51 (-4.52-3.67) |
| Syphilis | Guinea | 26137.5 (19810.3-33073.8) | 490.6 (370.3-628.7) | 58133.5 (43250.4-75069.2) | 457 (345.2-589.6) | -0.32 (-3.93-3.42) |
| Syphilis | Guinea-Bissau | 4021.3 (3024.2-5163.4) | 441.4 (333.5-567.5) | 8408.6 (6289.1-10953.2) | 413.4 (310.9-534.6) | 0.28 (-3.3-3.99) |
| Syphilis | Guyana | 1075.5 (793.7-1375.8) | 134.5 (101.8-171.5) | 1016.3 (766-1316.4) | 124.2 (94.9-159.1) | -0.3 (-4.05-3.59) |
| Syphilis | Haiti | 18975.1 (14278.6-24273) | 296.8 (225.3-375.3) | 36037.7 (27095.3-46158.5) | 259.2 (196.4-332.8) | -0.6 (-4.18-3.11) |
| Syphilis | Honduras | 4592.3 (3433.3-5936.9) | 111.8 (84-143.7) | 11357 (8377.2-14833.9) | 105.9 (80.2-136.4) | -0.35 (-4.55-4.04) |
| Syphilis | Hungary | 5813.8 (4446.2-7544.1) | 56.6 (42.7-74.1) | 4837.8 (3677.6-6144) | 55.4 (41.8-71.2) | 0.36 (-3.86-4.77) |
| Syphilis | Iceland | 192 (146.5-251.4) | 72.5 (55.2-94.8) | 241 (179.6-314.6) | 69.5 (51.3-90.2) | 0.38 (-3.03-3.91) |
| Syphilis | India | 2418936.1 (1744540.2-3187444.6) | 284.2 (208.6-371.2) | 3955481 (2909501-5191433.3) | 254.6 (189.5-331.6) | -0.44 (-4.05-3.31) |
| Syphilis | Indonesia | 502961.4 (362017-666325.5) | 260.8 (190.9-344.7) | 771419.9 (576211.5-1014314.4) | 253.7 (189.8-331.6) | 0.56 (-3.67-4.97) |
| Syphilis | Iran | 24060.4 (17250.4-32378.6) | 46.2 (33.4-62.3) | 45917.4 (34119.6-60867.8) | 50 (37.3-66.3) | 0.47 (-3.83-4.96) |
| Syphilis | Iraq | 13661.5 (9743-18232.2) | 79.1 (57.9-103.6) | 34615.8 (25048.3-45601.4) | 78.3 (57.2-102.7) | -0.11 (-4.46-4.44) |
| Syphilis | Ireland | 2471.5 (1839-3201) | 70 (52.2-90.6) | 3213.2 (2383.6-4111.9) | 67.4 (50.3-87.3) | -0.07 (-3.39-3.35) |
| Syphilis | Israel | 3319 (2508.9-4338.4) | 69.3 (52.8-90.3) | 6114.5 (4600.5-7901.2) | 67.4 (50.6-87.2) | 0.11 (-3.29-3.62) |
| Syphilis | Italy | 47643.1 (35254.2-62234.1) | 80.1 (59-104.9) | 38418.7 (28621.8-50219.5) | 72.2 (52.4-95.1) | -0.26 (-3.6-3.19) |
| Syphilis | Jamaica | 7381.5 (5727-9167.6) | 295.5 (234.3-363.5) | 6771.4 (5010-8842.8) | 218.3 (163.8-284.5) | -0.1 (-3.89-3.85) |
| Syphilis | Japan | 135964.3 (102464.5-176981.5) | 102.8 (76.3-135.3) | 106313.6 (79596.9-138596.4) | 97.4 (71.8-127.7) | 0.26 (-3.62-4.31) |
| Syphilis | Jordan | 3410.5 (2435-4549.2) | 92.1 (68.3-119) | 12390.4 (9085-16125.8) | 90.8 (67-117) | -0.02 (-4.11-4.25) |
| Syphilis | Kazakhstan | 11074.1 (8270.6-14788.1) | 65.3 (49.2-85.8) | 11290.1 (8404.2-14898.5) | 59.3 (44.1-78.5) | 0.39 (-3.64-4.58) |
| Syphilis | Kenya | 148851.8 (108740-191789.1) | 688.9 (514.4-896.2) | 240619.2 (178778.5-313954.8) | 470.4 (357.2-604.3) | -1.43 (-5.05-2.32) |
| Syphilis | Kiribati | 232.5 (174.3-300.5) | 295.6 (224-378.8) | 370.4 (283.2-481.3) | 289.5 (223-377.8) | -0.14 (-4.48-4.4) |
| Syphilis | Kuwait | 1680.2 (1214.1-2222.7) | 82.7 (61.3-106.8) | 4445 (3294.6-5830.8) | 77 (56.8-99.9) | -0.27 (-4.48-4.13) |
| Syphilis | Kyrgyzstan | 2518.7 (1827.8-3301) | 58.4 (43.2-75.9) | 3859.4 (2812.8-5091.5) | 54.9 (40.3-72.1) | 0.86 (-3.29-5.19) |
| Syphilis | Laos | 5623.9 (4160.2-7315.4) | 150.2 (112.1-191.6) | 12017.9 (8883.5-15785.6) | 149.3 (112.2-194.9) | 0.67 (-3.38-4.9) |
| Syphilis | Latvia | 1626.8 (1262.8-2071.6) | 59.2 (45.5-75.7) | 894.1 (675.7-1164.8) | 52.6 (39.3-68.4) | 0.27 (-3.61-4.31) |
| Syphilis | Lebanon | 2574 (1908.2-3375.9) | 86.8 (64.6-113.4) | 5202.7 (3859.7-6829.5) | 86.6 (64.1-113.8) | 0.32 (-3.65-4.46) |
| Syphilis | Lesotho | 9150.8 (6897.3-11607.8) | 661.6 (497.2-842.8) | 11461.8 (8596-14948.4) | 568.1 (432.4-728.9) | -0.33 (-3.99-3.46) |
| Syphilis | Liberia | 16185.1 (12192.2-20967) | 683.7 (520.6-878.8) | 35689.7 (26441.3-45752.4) | 622.9 (466.3-797.5) | -0.48 (-4.09-3.26) |
| Syphilis | Libya | 3260.1 (2376.7-4316.4) | 81.5 (61-107) | 6333.3 (4688.3-8247.9) | 79.1 (58.6-102.8) | 0.15 (-3.89-4.36) |
| Syphilis | Lithuania | 2108.4 (1617.7-2752.4) | 55 (42-71.7) | 1322.5 (1019.2-1707.9) | 51.4 (39.1-66.7) | 0.45 (-3.61-4.68) |
| Syphilis | Luxembourg | 321 (242.9-419.3) | 77.4 (58.5-100.2) | 473.7 (362.8-612.8) | 70.1 (53.2-91.5) | -0.24 (-3.47-3.11) |
| Syphilis | Macedonia | 959.3 (700.6-1248) | 45.8 (33.4-59.5) | 1043.2 (774.1-1380.8) | 46.5 (34.1-61.1) | 0.06 (-4.35-4.68) |
| Syphilis | Madagascar | 112509.3 (86140.4-140196) | 1011.4 (767.5-1246.4) | 239260.3 (180937.5-314507.3) | 848.2 (651-1091.4) | 0.59 (-3.5-4.85) |
| Syphilis | Malawi | 65451.8 (54625.1-77968.1) | 729 (608.9-875.1) | 118512.1 (92891.9-146164.7) | 647.4 (508.8-789.7) | -0.93 (-4.53-2.81) |
| Syphilis | Malaysia | 24301.7 (17713.2-32012.1) | 135.7 (102.3-176.8) | 49228.1 (36167.4-63753.5) | 136.5 (101.3-175.8) | -0.14 (-4.07-3.94) |
| Syphilis | Maldives | 314.7 (232.9-408.7) | 163.1 (124-207.3) | 1216 (886-1624.9) | 176.4 (131.2-230) | 0.08 (-4.31-4.67) |
| Syphilis | Mali | 44664.6 (34341.9-56735) | 568.1 (435.6-725.7) | 117093.9 (86472.9-152995.5) | 520.9 (388.9-680.3) | -0.76 (-4.46-3.08) |
| Syphilis | Malta | 263.8 (200.6-346.7) | 68.8 (52.2-90.3) | 276.4 (209.4-359.8) | 67.3 (50.4-87.9) | 0.67 (-2.67-4.13) |
| Syphilis | Marshall Islands | 138.4 (103.8-181.2) | 325.8 (248.5-417.8) | 197.8 (149.5-253.9) | 326.1 (248.8-417.3) | 0.87 (-3.11-5.02) |
| Syphilis | Mauritania | 10313.6 (7832.3-13259.1) | 537.8 (411.1-694.8) | 20000.7 (14740.8-26295.8) | 474.3 (354.3-620.7) | -0.14 (-4.38-4.29) |
| Syphilis | Mauritius | 2105.5 (1541.9-2812.5) | 171.4 (129.5-223.3) | 2127.9 (1594.8-2791.8) | 162.3 (121-212.9) | 0.69 (-3.22-4.77) |
| Syphilis | Mexico | 103555.6 (75131.8-137408.5) | 121.8 (90.2-159.5) | 148610.5 (109370.8-195240.9) | 107.9 (79.6-141.2) | 0.09 (-3.94-4.29) |
| Syphilis | Moldova | 3029.4 (2330.6-3930.3) | 67 (51.2-87.5) | 2225.4 (1702.7-2835.1) | 60.9 (45.6-78) | 0.22 (-3.64-4.23) |
| Syphilis | Monaco | 22.1 (16.9-28.5) | 84.5 (62-112.9) | 23.3 (18.1-29.1) | 77.6 (59.4-99.8) | -0.03 (-3.59-3.66) |
| Syphilis | Mongolia | 2218.9 (1619.4-2923.6) | 100.8 (75.1-130.3) | 3694.3 (2788.4-4713) | 111.1 (82.3-142.9) | 0.37 (-4.08-5.03) |
| Syphilis | Montenegro | 314.1 (233.3-409.8) | 47.9 (35.8-62.4) | 287 (221.4-373.3) | 48.6 (36.9-63.1) | 0 (-4.31-4.51) |
| Syphilis | Morocco | 63900.9 (46908-87094.3) | 242.2 (179.7-325.1) | 89567.5 (65282.9-120919) | 231.4 (168.9-313.3) | -0.27 (-4.32-3.94) |
| Syphilis | Mozambique | 180860.3 (143187.6-217283) | 1469.5 (1166.2-1784.6) | 310955.3 (232228.1-402760.7) | 1084.7 (825.5-1382.4) | -0.21 (-3.9-3.63) |
| Syphilis | Myanmar | 123255.4 (90083.2-163053.8) | 296.7 (219-387.3) | 168256.4 (125664.8-219541.6) | 284.1 (212.9-370.5) | -0.3 (-4.05-3.6) |
| Syphilis | Namibia | 8391 (6223.5-10941.1) | 631.7 (473.8-809.8) | 15102.4 (11316.1-19832.7) | 581.3 (443.6-753.2) | -0.75 (-4.39-3.03) |
| Syphilis | Nauru | 33.2 (25.1-43.3) | 311.1 (232.6-406.3) | 40.2 (30.4-51.8) | 333 (258.1-426.5) | 1.02 (-3.14-5.35) |
| Syphilis | Nepal | 53355.2 (39760.8-69130.5) | 287 (216.2-373.2) | 86998.6 (63269.3-113569.7) | 256.8 (190.7-332.3) | 0.14 (-3.56-3.98) |
| Syphilis | Netherlands | 11815.1 (8887.5-15513.2) | 72.3 (54.7-95.1) | 11046.7 (8320.8-14308.4) | 68.2 (50.4-88.8) | -0.06 (-3.48-3.47) |
| Syphilis | New Zealand | 3421.4 (2536.8-4525.7) | 95.3 (70.9-125.5) | 4562 (3349.5-5980.7) | 89.2 (65.1-116.9) | 0.13 (-3.66-4.07) |
| Syphilis | Nicaragua | 4000 (2931.7-5241.6) | 114.6 (87.8-148.1) | 7955 (5841-10378.8) | 110.7 (82.5-143.6) | 0.45 (-3.69-4.78) |
| Syphilis | Niger | 22337 (16572.8-29048.1) | 337.8 (254-430.8) | 65355.8 (47786.3-84260) | 318.4 (239.2-409.6) | -1.21 (-4.97-2.7) |
| Syphilis | Nigeria | 331005.9 (242601.5-434508.8) | 389.7 (284.1-512.3) | 725717.7 (533163.6-948816) | 328.7 (240.7-429.6) | -0.41 (-4.07-3.4) |
| Syphilis | Niue | 5.5 (4.1-7.2) | 259.7 (193.7-334.5) | 4.1 (3.2-5.1) | 272.5 (208.9-347.5) | 0.96 (-3.19-5.28) |
| Syphilis | North Korea | 28926.2 (21409.5-38197.1) | 131.5 (98.6-171) | 39246.6 (29383.6-50759) | 136.6 (102.6-177.8) | 0.61 (-3.52-4.92) |
| Syphilis | Northern Mariana Islands | 144.3 (106.4-188.4) | 250 (191-319.4) | 118.9 (92.4-151) | 259.2 (201.3-330) | 0.11 (-4.12-4.52) |
| Syphilis | Norway | 3291.7 (2407-4321.3) | 75.9 (55.5-99.4) | 4019.8 (3077.5-5209.2) | 75.4 (56.6-98.9) | -0.12 (-3.57-3.45) |
| Syphilis | Oman | 1432.2 (1031.3-1891.5) | 71.6 (53.6-93.2) | 4187.4 (3051.4-5575.6) | 70.6 (53-91.5) | 0.21 (-3.9-4.5) |
| Syphilis | Pakistan | 254289.3 (182370.3-339760.5) | 249.5 (181.8-334.7) | 570962.9 (409759.8-753706.4) | 230.6 (169.3-303.4) | 1.1 (-2.86-5.23) |
| Syphilis | Palau | 30.9 (22.6-40.1) | 182.5 (136.8-234.6) | 32.9 (25.5-42.6) | 193 (149.3-247.4) | 0.87 (-3.28-5.19) |
| Syphilis | Palestine | 1451.8 (1044.7-1927.4) | 78.2 (57.9-101.8) | 4246.5 (3113.3-5630.9) | 78.7 (58.6-102.8) | 0.16 (-4.12-4.63) |
| Syphilis | Panama | 3843.3 (2848.1-5022.3) | 153.9 (115.5-198.9) | 6611.7 (4974.1-8596.3) | 150.5 (113.8-195.3) | 0.21 (-3.6-4.17) |
| Syphilis | Papua New Guinea | 11200.3 (8475.9-14347.7) | 258.2 (199-329.3) | 27133.5 (20313.7-35736.6) | 243.4 (183.4-319.9) | 0.53 (-3.47-4.7) |
| Syphilis | Paraguay | 9850.8 (7222-13008.3) | 243.3 (181.2-319.1) | 18155.6 (13544.9-23682) | 234 (176.8-301.9) | 0.46 (-3.59-4.68) |
| Syphilis | Peru | 50728.9 (38024.7-64985.1) | 233.3 (178.2-297.4) | 84987.2 (63701.8-109407.2) | 218.3 (164.3-279.6) | 0.01 (-3.81-3.99) |
| Syphilis | Philippines | 129543.1 (94305.1-169985.9) | 204.5 (150-268.3) | 253970.9 (185828.8-333017.4) | 209.5 (154.9-273.4) | 1.16 (-2.86-5.36) |
| Syphilis | Poland | 19271.3 (14234.8-25552.5) | 50.3 (37.1-66.2) | 16376.7 (12126.5-21721.7) | 45.2 (32.9-59.7) | 0.18 (-4.01-4.55) |
| Syphilis | Portugal | 7456 (5553.4-9703.3) | 72.5 (53.6-95) | 6628.7 (5032.9-8621.9) | 68.2 (51.5-90.1) | -0.73 (-3.96-2.61) |
| Syphilis | Puerto Rico | 6026.8 (4531.5-7881.7) | 163.5 (123.4-213.9) | 4836 (3658.8-6239.6) | 160.8 (121.2-208.8) | -0.9 (-4.73-3.08) |
| Syphilis | Qatar | 1086.8 (780.8-1472) | 190.1 (141.4-250.9) | 7812.5 (5581.4-10655.2) | 184.6 (137.5-244.9) | -0.23 (-4.5-4.22) |
| Syphilis | Romania | 13202.1 (9985.1-17065.3) | 56.6 (42.2-73.5) | 9159.4 (6956.5-11882.2) | 53.6 (40.2-69.8) | 0.28 (-3.82-4.56) |
| Syphilis | Russian Federation | 94684.2 (71634.3-123637.1) | 60 (44.8-78.5) | 77797.3 (60100.5-101074.6) | 55.2 (41.4-72.2) | 1.19 (-2.71-5.26) |
| Syphilis | Rwanda | 45571.3 (34817.4-58220.8) | 720.4 (556.3-919) | 85892.7 (64333.2-110873.3) | 641.4 (487.1-815.7) | -0.97 (-4.64-2.84) |
| Syphilis | Saint Kitts and Nevis | 109.6 (78.8-142.6) | 261.9 (194.4-340.1) | 146.6 (109.8-191.2) | 234.7 (174.2-306.4) | -0.03 (-3.79-3.88) |
| Syphilis | Saint Lucia | 316.4 (229.4-412.5) | 228.6 (171.3-292.9) | 409.5 (310.7-526.9) | 222.6 (169.7-285.4) | -0.41 (-4.16-3.49) |
| Syphilis | Saint Vincent and the Grenadines | 235.7 (175-307.9) | 216.9 (166.4-279.6) | 200.1 (153.1-257.6) | 174.7 (133-226.9) | -0.51 (-4.33-3.47) |
| Syphilis | Samoa | 210.2 (155.9-270) | 132.3 (100.1-169) | 271.4 (207.2-350.1) | 132.2 (100.9-171.6) | 0.97 (-3.28-5.41) |
| Syphilis | San Marino | 19.1 (14.3-24.6) | 81.8 (60.9-106.5) | 21.4 (16.5-27.4) | 78.8 (59.9-100.7) | -0.07 (-3.62-3.61) |
| Syphilis | Sao Tome and Principe | 403 (299-524.1) | 391 (294.8-505.5) | 876.3 (648.1-1143.2) | 392.4 (294.2-509.2) | -0.49 (-4.64-3.84) |
| Syphilis | Saudi Arabia | 11153.7 (8030.8-14667.1) | 70.6 (51.7-91.4) | 33698.9 (24590.7-45093.7) | 69.1 (51.3-90.4) | -0.03 (-4.22-4.34) |
| Syphilis | Senegal | 36177.8 (27077.2-46869.5) | 551.4 (420-716.3) | 82676.6 (60427.9-108395.8) | 531.9 (391.9-689.3) | -0.7 (-4.3-3.04) |
| Syphilis | Serbia | 4595.5 (3412.6-6018.3) | 46.3 (34.1-60.7) | 3977 (2962.3-5234.6) | 46.1 (33.8-60.3) | 0 (-4.35-4.55) |
| Syphilis | Seychelles | 121.7 (89.3-159.3) | 162.4 (122.9-210.2) | 185.5 (142.9-235.2) | 168.9 (129.1-213) | -0.26 (-4.28-3.94) |
| Syphilis | Sierra Leone | 15444.4 (11580.3-20044.9) | 398.2 (301.7-509.8) | 34870.5 (25866.7-45777.9) | 390.9 (296.3-505.7) | -0.21 (-3.84-3.55) |
| Syphilis | Singapore | 3655.5 (2655.7-4841.5) | 98 (72.9-129) | 5704.3 (4245.6-7527.9) | 91.6 (68.7-119.8) | -0.46 (-3.96-3.18) |
| Syphilis | Slovakia | 2280.3 (1648.7-3008.7) | 42.5 (30.8-56.3) | 2236.3 (1687-2886.2) | 42.5 (31.8-55.1) | 0.05 (-4.36-4.66) |
| Syphilis | Slovenia | 966.8 (725.1-1250.3) | 46.5 (34.7-60.1) | 831.1 (629.2-1083.7) | 45.1 (33-59.4) | -0.2 (-4.32-4.1) |
| Syphilis | Solomon Islands | 1091.7 (820.5-1412.1) | 325.6 (247.9-415.3) | 2254.2 (1678.6-2916.4) | 316.1 (237.1-409.7) | 0.94 (-3.15-5.19) |
| Syphilis | Somalia | 58266.8 (44896.4-73664.2) | 826.1 (637-1050.2) | 147223.9 (112074.6-187643.1) | 761.2 (582.9-963.7) | -0.55 (-4.47-3.53) |
| Syphilis | South Africa | 427446.6 (316928.6-547464.5) | 1083.7 (812.2-1382.5) | 405123 (299428.3-534038.1) | 652.2 (487-850.3) | -0.52 (-4.33-3.44) |
| Syphilis | South Korea | 51340.4 (38283.9-66974.5) | 100.2 (76.1-129.6) | 52678.1 (39285.6-68992.1) | 100.1 (74.3-129.9) | 0.02 (-3.88-4.08) |
| Syphilis | South Sudan | 61906.2 (46786.9-78503.8) | 1149.9 (873.5-1458.8) | 88444.8 (66741.1-114919.2) | 995.6 (754.3-1275.2) | -0.09 (-3.83-3.8) |
| Syphilis | Spain | 30496.7 (23006.5-39809.4) | 76.2 (57.6-99.7) | 29717.5 (22679.6-38622.2) | 69.7 (51.9-91.4) | -0.6 (-3.89-2.82) |
| Syphilis | Sri Lanka | 11847.8 (8611.4-15486.9) | 64.9 (47.9-84.8) | 13818.6 (10245.6-17915.6) | 61.7 (45.7-79.9) | 0.01 (-4.41-4.63) |
| Syphilis | Sudan | 28998.3 (21394.9-37794.1) | 145.4 (108-188.5) | 64465.2 (46964.1-86544.7) | 139.2 (103.4-184) | 0.13 (-3.67-4.08) |
| Syphilis | Suriname | 510.7 (376.8-664.2) | 126.8 (94.9-162) | 716.8 (556-911.6) | 121.5 (93.8-155.1) | -0.09 (-3.89-3.85) |
| Syphilis | Swaziland | 6414.9 (4871.3-8048.2) | 843.1 (641.8-1060.6) | 7481.5 (5521.3-9651.4) | 604.6 (455.9-773.6) | -0.24 (-3.91-3.57) |
| Syphilis | Sweden | 6475.8 (4760.6-8488.3) | 75.9 (55.6-98.6) | 6939.6 (5156.4-9070.7) | 72.5 (53.3-94.5) | -0.15 (-3.64-3.47) |
| Syphilis | Switzerland | 5130.2 (3861-6776.5) | 68.8 (51.6-90.1) | 5845.3 (4515.3-7582.8) | 66.9 (50.6-87.4) | -0.79 (-4.08-2.62) |
| Syphilis | Syria | 9657.6 (7018.7-12585.4) | 83.3 (62.5-106.9) | 10792.6 (8109.1-13920.6) | 77.6 (57.4-100.5) | 0 (-4.28-4.47) |
| Syphilis | Taiwan | 31248.3 (22756.7-41480.3) | 136 (101-177.6) | 30988.6 (23701.9-40540.2) | 131 (98.6-171.1) | 0.43 (-3.63-4.67) |
| Syphilis | Tajikistan | 3540.6 (2660.7-4588.6) | 72.6 (55.7-92.4) | 7256.4 (5513.1-9356.9) | 69.7 (53.4-89) | 0.14 (-4.11-4.59) |
| Syphilis | Tanzania | 268173.8 (230464.3-314688.3) | 1095.8 (939.1-1287.8) | 382621.9 (289864.1-497093.9) | 674.2 (517.1-861.2) | -1.24 (-4.97-2.64) |
| Syphilis | Thailand | 133864.8 (97567.2-177455.5) | 209.7 (156.4-272.7) | 130577.7 (98327.6-171446.9) | 197.2 (145.6-258.6) | -0.72 (-4.45-3.15) |
| Syphilis | The Bahamas | 570.4 (409.5-754.3) | 196.5 (146.4-254.6) | 810.7 (608-1038) | 192.9 (143.8-248.9) | -0.3 (-4.02-3.56) |
| Syphilis | The Gambia | 4049.3 (3035.9-5253.3) | 428.1 (322.8-551.6) | 9761.3 (7193.9-12868.6) | 398.4 (299.5-516.1) | 0.14 (-3.42-3.83) |
| Syphilis | Timor-Leste | 1276.8 (934.7-1668.6) | 163.4 (122.5-211.5) | 2186.9 (1598.8-2854.3) | 158.6 (120.2-204.8) | -0.2 (-4.01-3.76) |
| Syphilis | Togo | 13633.6 (10238.5-17906.7) | 411.3 (312.6-533.8) | 32880 (24563.2-42869.6) | 393.4 (295.9-509.8) | -0.96 (-4.47-2.68) |
| Syphilis | Tokelau | 4.1 (3-5.4) | 275.2 (201.3-359.7) | 3.4 (2.6-4.3) | 275.5 (213.9-347.2) | 0.84 (-3.31-5.17) |
| Syphilis | Tonga | 179.2 (134.2-235) | 193.5 (145.9-249) | 198.9 (151.8-255.8) | 194.8 (147.6-252.8) | 1.02 (-3.16-5.37) |
| Syphilis | Trinidad and Tobago | 1752.7 (1278.7-2281.7) | 141.3 (105.9-181.5) | 1922.5 (1456.3-2505) | 138.3 (103.5-179.8) | -0.02 (-3.72-3.83) |
| Syphilis | Tunisia | 6618.2 (4815.8-8734.6) | 78.5 (58.3-102.4) | 9359.4 (7032.3-12200.1) | 77.8 (57.9-101) | 0.28 (-3.98-4.72) |
| Syphilis | Turkey | 36880.5 (26964.8-48502.8) | 61.6 (45.7-80.6) | 52627.3 (38692.8-68805.7) | 60.8 (44.5-79.6) | -0.04 (-4.32-4.44) |
| Syphilis | Turkmenistan | 2480 (1799.4-3240.1) | 69.3 (52-89.1) | 3379.9 (2518.7-4444.3) | 62.2 (46.5-81.5) | -0.39 (-4.59-4) |
| Syphilis | Tuvalu | 20.1 (14.8-26.6) | 206.7 (152.9-271.3) | 27.2 (20.6-35.2) | 213.9 (162.9-274.8) | 0.84 (-3.32-5.17) |
| Syphilis | Uganda | 113945.7 (85101.8-146204) | 735.4 (561.1-937.2) | 301601.4 (225459.7-393243.5) | 728.6 (554.1-930) | -0.66 (-4.3-3.11) |
| Syphilis | Ukraine | 40139.1 (31282.1-51285.2) | 74.4 (56.4-96.2) | 21085.6 (15894.7-27705.2) | 50.3 (37.2-66.5) | 0.33 (-3.48-4.29) |
| Syphilis | United Arab Emirates | 1760.4 (1263-2378.7) | 75.8 (57-99.5) | 9490.5 (6840-13145.6) | 73.1 (55.3-95.1) | 0.15 (-4.02-4.5) |
| Syphilis | United Kingdom | 47966 (36272.7-62335) | 82.2 (61.9-107.1) | 50184 (38172.5-64702.2) | 78 (58.5-101) | 0 (-3.29-3.4) |
| Syphilis | United States | 314608.6 (232107.4-416589.5) | 116.7 (86.9-153) | 352848.9 (266470-457847.4) | 110.2 (81.9-143.5) | -0.15 (-3.67-3.5) |
| Syphilis | Uruguay | 6394.8 (4846.9-8226.2) | 211.2 (159.9-271.2) | 6294.9 (4680.1-8243.4) | 190.6 (140.6-248.6) | 0.16 (-3.27-3.71) |
| Syphilis | Uzbekistan | 14084.4 (10232.9-18592.3) | 70.7 (53.3-91.4) | 21617.3 (16069.3-28838.3) | 59.7 (44.6-79.2) | 0.53 (-3.58-4.81) |
| Syphilis | Vanuatu | 444 (336.2-573.8) | 291.8 (221.7-373) | 948.2 (716.8-1243.3) | 292.2 (221.8-383.5) | 0.92 (-3.27-5.3) |
| Syphilis | Venezuela | 36965.8 (26705.3-48598.4) | 187.7 (139.6-244) | 47582 (36208.2-61705.3) | 184.4 (138.7-241) | 0.25 (-3.64-4.3) |
| Syphilis | Vietnam | 95454.4 (69825.3-126353.6) | 141.7 (105.5-183.8) | 154351.8 (117128.4-201108.6) | 145.4 (109.6-189) | 0.32 (-3.54-4.34) |
| Syphilis | Yemen | 9987.5 (7392.4-13084.7) | 89 (66.3-115.6) | 29391.4 (21478.1-38741.4) | 88.8 (65.9-116.9) | 0.51 (-3.57-4.77) |
| Syphilis | Zambia | 73179 (58652.7-88773.6) | 1000.4 (789.9-1216.5) | 170730.5 (129082.7-219776.2) | 892.3 (679.3-1136.5) | -0.88 (-4.51-2.9) |
| Syphilis | Zimbabwe | 63757 (46946.5-82119.7) | 668.3 (499.2-840.8) | 82099.9 (60862.1-106345.3) | 533.5 (404.4-683.5) | -1.3 (-4.89-2.44) |
| Trichomoniasis | Afghanistan | 225034.6 (174908.2-302803.7) | 3316.5 (2500.7-4424.3) | 770518.8 (579820.2-1013034.1) | 3365.5 (2539.9-4493.5) | 0.27 (-3.89-4.6) |
| Trichomoniasis | Albania | 101280.7 (74158.1-133832.9) | 3265.3 (2431.2-4308.9) | 93901.2 (71714.5-123782.4) | 3247.8 (2433-4329.8) | -0.11 (-4.53-4.51) |
| Trichomoniasis | Algeria | 645567.2 (480510.4-839317) | 3424.7 (2577.5-4550.6) | 1584062 (1172434.1-2122742.6) | 3379.5 (2547.6-4494.4) | 0.49 (-3.58-4.74) |
| Trichomoniasis | American Samoa | 3035.9 (2252.1-4002.5) | 7057.7 (5307.8-9265.6) | 3316.8 (2529.5-4441.1) | 6854.9 (5145-9106.6) | 0.96 (-3.19-5.29) |
| Trichomoniasis | Andorra | 1639.8 (1197-2200.4) | 2430.4 (1839.9-3236.3) | 2488.1 (1914.4-3431.9) | 2363.5 (1761-3154) | -0.13 (-3.69-3.57) |
| Trichomoniasis | Angola | 448225.5 (330983-589459.2) | 5789.1 (4367.9-7720.3) | 1374376 (1031405.3-1812752.7) | 5652.9 (4266.4-7468.2) | 0.43 (-3.19-4.19) |
| Trichomoniasis | Antigua and Barbuda | 2950 (2146.8-3895.4) | 5124.5 (3787.2-6886.7) | 5389.5 (4040.4-7269.8) | 5185.5 (3878.1-6972.9) | -0.21 (-4.12-3.85) |
| Trichomoniasis | Argentina | 889116.1 (654495.2-1168896.1) | 2806.7 (2057.9-3696.9) | 1399587.5 (1045662.6-1896208.6) | 2837.7 (2118.4-3825) | -0.06 (-3.49-3.5) |
| Trichomoniasis | Armenia | 134665.3 (98803.2-177876.8) | 3940.9 (2924-5256.1) | 133733.4 (99206.6-178835.9) | 3957.9 (2938.7-5315.6) | 0.92 (-3.39-5.42) |
| Trichomoniasis | Australia | 510839 (378205-672303.3) | 2762.1 (2049.5-3624.5) | 784804.3 (593904-1040179.9) | 2788.4 (2085.9-3672.7) | -0.13 (-3.7-3.57) |
| Trichomoniasis | Austria | 201888.5 (151520.8-264310.8) | 2329.5 (1728.2-3088.8) | 235869.7 (179223-325206.2) | 2344.8 (1753.5-3183.8) | -0.37 (-3.76-3.13) |
| Trichomoniasis | Azerbaijan | 271218.2 (199664.4-353056.1) | 3964.4 (2973.6-5215.4) | 484095.5 (358477.2-642604.4) | 3943.7 (2948.5-5228.2) | 0 (-4.41-4.62) |
| Trichomoniasis | Bahrain | 19682.4 (14198.2-26633.8) | 3625 (2729.2-4894.2) | 74614 (55772.4-102591.5) | 3664.5 (2773.6-4939.2) | -0.25 (-4.4-4.07) |
| Trichomoniasis | Bangladesh | 2135998 (1589971.9-2826491.2) | 2610.9 (1959.6-3454.4) | 4395675.6 (3252564.9-5839977.5) | 2590.1 (1935.9-3453.6) | 0.02 (-4.03-4.25) |
| Trichomoniasis | Barbados | 13553.4 (9977.2-17807.3) | 5195.7 (3886.1-6844.5) | 17180.6 (13042.3-23271.7) | 5192.6 (3885.8-6986.3) | -0.19 (-4.02-3.8) |
| Trichomoniasis | Belarus | 312264.2 (230836.3-411064.9) | 2791.3 (2065.4-3696.6) | 298133.8 (224838.4-405026.9) | 2778.1 (2064.3-3729.4) | 0.97 (-3.01-5.1) |
| Trichomoniasis | Belgium | 238279.5 (180839.5-322056) | 2144.9 (1611.5-2913.5) | 264041.8 (201731-353469.1) | 2150 (1610.6-2887.2) | -0.14 (-3.44-3.28) |
| Trichomoniasis | Belize | 6990.6 (5199.1-9336.5) | 5121.2 (3861.4-6934.4) | 22371.5 (16662-29940.2) | 5161.9 (3870.9-6957.7) | -0.21 (-3.99-3.72) |
| Trichomoniasis | Benin | 208200.8 (152633.4-272804.3) | 6197.8 (4674.2-8259.4) | 651926.2 (478134.5-869648.1) | 6323.1 (4762.7-8449.5) | -0.5 (-4.08-3.21) |
| Trichomoniasis | Bermuda | 3692.2 (2714.5-4964.7) | 5143.2 (3824.4-6909.2) | 3687.9 (2845-4912.5) | 5193.8 (3913.2-6875.6) | -0.48 (-4.39-3.6) |
| Trichomoniasis | Bhutan | 12533.1 (9276-16519.9) | 2584.3 (1935.8-3456.1) | 21651.5 (16054.3-28857.8) | 2570.5 (1944.5-3437.3) | 0.16 (-3.49-3.96) |
| Trichomoniasis | Bolivia | 213391 (156799.3-285869.5) | 4212.8 (3128.9-5617.4) | 501136.7 (371713.5-669246.2) | 4201.5 (3168.4-5669.1) | -0.33 (-4.02-3.51) |
| Trichomoniasis | Bosnia and Herzegovina | 156339.1 (115853-204137) | 3189.3 (2382.7-4156.7) | 118903.8 (89505.1-158878.8) | 3222.4 (2375.5-4271.5) | 0.06 (-4.35-4.67) |
| Trichomoniasis | Botswana | 86594.7 (66777.6-110896.1) | 8578 (6677-10985.5) | 214188.7 (158738.6-285162.5) | 8097.4 (6132.6-10768.3) | -1.25 (-4.85-2.49) |
| Trichomoniasis | Brazil | 7570149.9 (5614884-10195942.6) | 5592.9 (4192.2-7662.9) | 13905513.7 (10382821.3-19337117.8) | 5553.2 (4158.7-7677.6) | 0.06 (-3.86-4.14) |
| Trichomoniasis | Brunei | 7602.3 (5416.7-10197.7) | 2930.8 (2179.8-3857.2) | 16225.1 (11788.8-21797.2) | 2915.3 (2161.7-3877.9) | 0.32 (-3.39-4.18) |
| Trichomoniasis | Bulgaria | 301072.2 (225866.1-405991.5) | 3197.9 (2378.6-4288.9) | 244510.3 (185257.3-336119.8) | 3222.7 (2404.6-4340.2) | 0.4 (-3.8-4.78) |
| Trichomoniasis | Burkina Faso | 662409.1 (504976.6-836487.2) | 9952.1 (7647.9-12704.9) | 1518868.5 (1140693.1-1967842.2) | 8685.2 (6586-11407.8) | -1.47 (-5.19-2.41) |
| Trichomoniasis | Burundi | 390894.5 (292012.8-510555.9) | 9634.1 (7370.5-12753) | 1013299.6 (758019.1-1324365.9) | 9772 (7453.3-13020.2) | -1.89 (-5.64-2.01) |
| Trichomoniasis | Cambodia | 333134.3 (247868.8-441046.4) | 4299.6 (3186-5633) | 740231.6 (549360.3-981132.1) | 4234.2 (3193.3-5667.7) | -0.55 (-4.4-3.45) |
| Trichomoniasis | Cameroon | 631321.9 (474796.8-822467.2) | 8177.2 (6231.8-10710.2) | 2144406.4 (1598641.4-2806668.6) | 8152 (6150.2-10628.9) | -0.82 (-4.42-2.91) |
| Trichomoniasis | Canada | 1255995.3 (919291.4-1675622.9) | 4010.4 (2963.9-5353.4) | 1651247.7 (1247490.5-2201287.4) | 4142.5 (3072.7-5532.9) | 0.1 (-3.42-3.74) |
| Trichomoniasis | Cape Verde | 18042.5 (13581.7-23099.4) | 7413 (5584.9-9842.7) | 45820 (33989.4-59997.7) | 7411 (5598-9674.7) | -0.72 (-4.4-3.11) |
| Trichomoniasis | Central African Republic | 118032.4 (87329.5-155822.8) | 5674.5 (4291.8-7482.9) | 256287.8 (189126.2-344334.8) | 5716.6 (4286.3-7723) | -0.86 (-4.39-2.79) |
| Trichomoniasis | Chad | 315533.1 (235694.3-412534.3) | 7503.6 (5602.1-9807) | 896809.3 (676138.5-1194503.2) | 7587.8 (5814.3-10047.1) | -0.59 (-4.2-3.15) |
| Trichomoniasis | Chile | 369455.9 (273775.1-488577.5) | 2804.9 (2105.2-3733.7) | 602521.3 (452378.6-791989) | 2858.2 (2137.9-3741.5) | 0.38 (-3.11-4.01) |
| Trichomoniasis | China | 47890099.6 (35282940.9-64506392.6) | 4021.8 (3005.5-5466.6) | 65088138.4 (49203371.2-90285927.3) | 3886.4 (2908.6-5297.2) | -0.08 (-4.2-4.23) |
| Trichomoniasis | Colombia | 1924846.1 (1423653.1-2541721.2) | 6474.1 (4824.2-8599.7) | 3447651.1 (2588939.2-4603147.3) | 6410.8 (4819.4-8599.3) | 0.24 (-3.66-4.29) |
| Trichomoniasis | Comoros | 31906.8 (24322.3-41547.6) | 9618.3 (7448.8-12878.6) | 69985 (52604.3-92613.6) | 9648 (7254-12929.3) | -0.17 (-4.66-4.53) |
| Trichomoniasis | Congo | 99870.4 (74096-131744.1) | 5616.9 (4193.6-7458.4) | 291264.7 (217490.3-387514.1) | 5701.9 (4306.3-7572.3) | -0.53 (-4.06-3.13) |
| Trichomoniasis | Cook Islands | 1243.2 (922.4-1644.3) | 7248.4 (5369.4-9795.8) | 1247.4 (960.3-1653.1) | 7096.8 (5343.1-9431.9) | 1.05 (-3.11-5.39) |
| Trichomoniasis | Costa Rica | 177180.9 (129363.2-232918.2) | 6441.9 (4797.7-8489.5) | 341944.7 (251681.3-451105.6) | 6429.8 (4737.9-8495.9) | 0.07 (-3.98-4.28) |
| Trichomoniasis | Cote d'Ivoire | 646572.4 (482414.9-860600) | 7012.5 (5293.4-9361.8) | 1692535.1 (1261509.6-2261916.5) | 7073.1 (5334.2-9457.8) | -1.18 (-4.71-2.49) |
| Trichomoniasis | Croatia | 174923.5 (131999.3-229986.2) | 3194.1 (2396.5-4153.2) | 149339.9 (113052.8-201945.3) | 3238.5 (2421.2-4348.1) | 0.08 (-4.16-4.5) |
| Trichomoniasis | Cuba | 582361.6 (438725.2-761851.4) | 5141.2 (3889.7-6843.8) | 651421.3 (495149.2-860914.6) | 5162.4 (3883.7-6866.3) | 0.92 (-3.19-5.22) |
| Trichomoniasis | Cyprus | 19207.5 (14282.2-25304) | 2331.1 (1736.2-3083.3) | 38598.7 (28999.7-51682.9) | 2309.1 (1743.2-3039.8) | 0.04 (-3.63-3.85) |
| Trichomoniasis | Czech Republic | 361740.3 (269998.5-493767.5) | 3226.9 (2371.9-4313.2) | 384738.2 (296545.5-524070.3) | 3240.3 (2416-4320.4) | 0.39 (-3.87-4.83) |
| Trichomoniasis | Democratic Republic of the Congo | 1571466 (1154867.8-2085990) | 5698.1 (4279.5-7697.8) | 4143851.3 (3075469.3-5576497.5) | 5739.1 (4292.7-7819.3) | -1.3 (-4.94-2.49) |
| Trichomoniasis | Denmark | 135614.7 (101406.7-181836.1) | 2336.3 (1742.1-3095.6) | 144384.9 (110085-190359.9) | 2336.4 (1751.9-3097.7) | -0.26 (-3.73-3.34) |
| Trichomoniasis | Djibouti | 32331.4 (24304.8-42235) | 9808.7 (7451.5-12822.6) | 128085.8 (94731.2-170880.7) | 9848.5 (7426.1-13193.5) | -0.19 (-3.94-3.7) |
| Trichomoniasis | Dominica | 3142.4 (2333.2-4140) | 5104.6 (3813.4-6790.7) | 3730.8 (2796.4-4954.5) | 5149.3 (3789.7-6832.6) | -0.21 (-4.16-3.9) |
| Trichomoniasis | Dominican Republic | 309052.4 (227268.9-409764.4) | 5129 (3824.3-6790.5) | 594339.6 (443525.7-792376.9) | 5145.9 (3865.6-6878.3) | -0.71 (-4.46-3.19) |
| Trichomoniasis | Ecuador | 349623.1 (256545.9-464408.8) | 4208.7 (3163.2-5648.4) | 768822.9 (573837.9-1030522) | 4192.8 (3150-5639.1) | 0.54 (-3.22-4.44) |
| Trichomoniasis | Egypt | 1772086 (1308067-2340299.9) | 3846.6 (2910-5104.6) | 3722556.4 (2771997.1-4957679) | 3727.5 (2817.7-4909.1) | 0 (-4.33-4.53) |
| Trichomoniasis | El Salvador | 272281.9 (201076.6-355961.3) | 6475.6 (4821.1-8524.9) | 413858.7 (307704.8-544636.3) | 6478.4 (4804.2-8602.5) | -0.23 (-4.16-3.86) |
| Trichomoniasis | Equatorial Guinea | 17225.9 (12878-22587.4) | 5661.7 (4258.2-7489.2) | 73299.4 (53404.6-98386.2) | 5656.2 (4293.7-7667.5) | 0.57 (-2.97-4.24) |
| Trichomoniasis | Eritrea | 242302.6 (182490.1-314496.8) | 9654.9 (7295.4-12541.2) | 567030.6 (420096.6-748871.9) | 9552.6 (7231.1-12758.8) | -1.3 (-5.1-2.65) |
| Trichomoniasis | Estonia | 47127.8 (35246.3-63546.1) | 2784.7 (2066.9-3745.1) | 40463.5 (30773.2-54952.6) | 2810.4 (2077.3-3756.8) | 0.65 (-3.26-4.73) |
| Trichomoniasis | Ethiopia | 3503104 (2639999.8-4659300.5) | 9925.9 (7489.3-13526.9) | 8547252.3 (6375610.8-11443952.3) | 9775.4 (7321.2-13339.4) | -1.38 (-5.11-2.5) |
| Trichomoniasis | Federated States of Micronesia | 5630.5 (4155-7378.7) | 6967.5 (5221.2-9009.2) | 6845.4 (5100.3-8917.7) | 6772.7 (5059.5-8848.9) | 0.99 (-3.14-5.3) |
| Trichomoniasis | Fiji | 45085.5 (34104.2-58134.4) | 6337.9 (4833.5-8178.1) | 59392.6 (44374.3-79197.2) | 6280.3 (4692.3-8325.6) | 0.08 (-4.15-4.5) |
| Trichomoniasis | Finland | 134884.1 (99979.2-177896.6) | 2330.6 (1742.9-3079.3) | 137177.2 (105010.8-179353) | 2360 (1765.7-3109.8) | -0.17 (-3.68-3.47) |
| Trichomoniasis | France | 1591053.1 (1206840.9-2116355.1) | 2536 (1919.3-3386.5) | 1696803.4 (1322273.5-2252916.9) | 2490.2 (1878.2-3327.1) | 0.07 (-3.21-3.47) |
| Trichomoniasis | Gabon | 43393 (32006.6-57039.4) | 5704.3 (4256.2-7647.1) | 94257.2 (70116.1-126162.3) | 5672.5 (4235.2-7666.8) | -0.52 (-4.04-3.14) |
| Trichomoniasis | Georgia | 224903.4 (168330.8-297023.9) | 3933.6 (2952.2-5253.9) | 150168.6 (113968.2-202216.8) | 3932.5 (2959.1-5243) | 0.67 (-3.52-5.04) |
| Trichomoniasis | Germany | 2143174.5 (1614065.7-2844684.2) | 2335.2 (1738.2-3122.7) | 2180883.7 (1671158.6-2892494.8) | 2364.4 (1763.1-3155) | -0.12 (-3.64-3.52) |
| Trichomoniasis | Ghana | 859059.6 (644794.6-1126660.9) | 7385.1 (5627.7-9700.4) | 2334561.6 (1717506.4-3110991.9) | 7454.5 (5561.7-9992.9) | -0.48 (-4.16-3.34) |
| Trichomoniasis | Greece | 288734.6 (218715.8-381769.1) | 2590.9 (1949-3418.3) | 286478.2 (218995.6-381159.1) | 2580.1 (1912.3-3391.9) | 0.58 (-2.98-4.27) |
| Trichomoniasis | Greenland | 2533.1 (1848.4-3373.8) | 3863.8 (2864.1-5051.3) | 2477 (1853.6-3286.4) | 4048.5 (3015.8-5352) | 0.04 (-3.4-3.61) |
| Trichomoniasis | Grenada | 3507 (2601.9-4609.6) | 5118.9 (3834.5-6814.6) | 5741.1 (4329.6-7715.2) | 5144.4 (3837.2-6891.1) | -0.4 (-4.39-3.75) |
| Trichomoniasis | Guam | 9988.9 (7326.7-13173.9) | 6893 (5156.9-9057.5) | 10990.3 (8260.1-14307.1) | 6832.6 (5115.7-8877.6) | 0.1 (-4.06-4.44) |
| Trichomoniasis | Guatemala | 383137.4 (284326.6-505692.7) | 6450 (4809.9-8614.9) | 962944.6 (708641.7-1272023.2) | 6394.7 (4757.9-8522.4) | -0.51 (-4.52-3.67) |
| Trichomoniasis | Guinea | 339601.1 (251022.4-444695.7) | 7500.2 (5625.1-9813.2) | 783478.7 (579776.8-1015051.3) | 7579.4 (5649.6-10050.7) | -0.32 (-3.93-3.42) |
| Trichomoniasis | Guinea-Bissau | 53345.3 (39669.5-70148.2) | 7356 (5550.5-9679) | 125723.8 (93068.5-164902.8) | 7448 (5652.3-9905.4) | 0.28 (-3.3-3.99) |
| Trichomoniasis | Guyana | 34178.6 (25265-45036.2) | 5113.1 (3866.1-6830.9) | 39634.1 (29762.5-52371.1) | 5143.9 (3849.1-6881.1) | -0.3 (-4.05-3.59) |
| Trichomoniasis | Haiti | 262705.2 (194432.9-342870.6) | 5155.3 (3869.7-6806) | 649086.4 (474129.1-870397.9) | 5154.1 (3853.1-6911.9) | -0.6 (-4.18-3.11) |
| Trichomoniasis | Honduras | 219567.6 (162227.8-286248.5) | 6457 (4798.2-8506.6) | 617688.2 (457386-818570.1) | 6401.8 (4786.7-8522.8) | -0.35 (-4.55-4.04) |
| Trichomoniasis | Hungary | 364515.6 (275519.4-489106.7) | 3205.9 (2406.5-4244.4) | 346773 (266909.6-470659.1) | 3220.4 (2407.4-4279.3) | 0.36 (-3.86-4.77) |
| Trichomoniasis | Iceland | 6222.8 (4600.6-8241.6) | 2343.9 (1748.9-3107.9) | 8993.8 (6769.6-11964.1) | 2376.4 (1758.1-3203.2) | 0.38 (-3.03-3.91) |
| Trichomoniasis | India | 19688291.5 (14617435.9-26660262.8) | 2658 (1991.2-3617.9) | 39515102.2 (29362474.2-53792137.5) | 2633.4 (1961.8-3589) | -0.44 (-4.05-3.31) |
| Trichomoniasis | Indonesia | 7252592.7 (5385316.3-9760137.4) | 4432.7 (3334.9-6067.5) | 13753811.8 (10215398-18990014.7) | 4353.5 (3258.6-5931.1) | 0.56 (-3.67-4.97) |
| Trichomoniasis | Iran | 1529524.2 (1143896.5-2049983.1) | 3758.3 (2842.3-5164) | 3854404.3 (2871237.7-5364970.3) | 3688.7 (2784.6-5021.7) | 0.47 (-3.83-4.96) |
| Trichomoniasis | Iraq | 598620.8 (449726.1-790883.3) | 4424.7 (3360.2-5889.2) | 1568847.4 (1177654.9-2100971.5) | 3912.5 (2948-5195.1) | -0.11 (-4.46-4.44) |
| Trichomoniasis | Ireland | 82229.4 (62054.3-108959.2) | 2333 (1755.9-3088.6) | 124075.7 (94371.3-169987.2) | 2313 (1727.4-3062.5) | -0.07 (-3.39-3.35) |
| Trichomoniasis | Israel | 106909.5 (79833-141818.1) | 2278 (1703.3-3018.1) | 218161.5 (166211.7-288084.7) | 2323.5 (1741.3-3053.9) | 0.11 (-3.29-3.62) |
| Trichomoniasis | Italy | 1524580.2 (1156151.5-2068160.2) | 2424 (1813.8-3314.2) | 1597266.1 (1227415.7-2166003.2) | 2435.6 (1822.3-3275.9) | -0.26 (-3.6-3.19) |
| Trichomoniasis | Jamaica | 102361.3 (75472.4-134144.2) | 5156.4 (3867.4-6853.8) | 160427.9 (120548.5-211549.9) | 5169.6 (3892.6-6837.3) | -0.1 (-3.89-3.85) |
| Trichomoniasis | Japan | 4300285.4 (3241387-6043958.1) | 3002.1 (2251.1-4115) | 4029799 (3111408.4-5579786.4) | 3001.1 (2235.4-4099.1) | 0.26 (-3.62-4.31) |
| Trichomoniasis | Jordan | 67989.1 (50589.2-89182) | 2559.9 (1940.4-3387.2) | 324414.6 (244958-431764.9) | 2587.6 (1977.3-3451.4) | -0.02 (-4.11-4.25) |
| Trichomoniasis | Kazakhstan | 627570.7 (458522.3-824446.5) | 3891.4 (2885-5164.1) | 794637 (588271.6-1065003.1) | 3944.6 (2944.3-5247.1) | 0.39 (-3.64-4.58) |
| Trichomoniasis | Kenya | 1355103.4 (1016914.1-1798899) | 8894.5 (6750-12231.3) | 4089927 (3053805.9-5489351.8) | 9422.2 (7125.2-12916.2) | -1.43 (-5.05-2.32) |
| Trichomoniasis | Kiribati | 4635.9 (3445.9-6137.8) | 7091.7 (5365.2-9320.1) | 8059 (5978.2-10646.3) | 6886.9 (5188.8-9140.4) | -0.14 (-4.48-4.4) |
| Trichomoniasis | Kuwait | 61943.9 (44480.7-82263.4) | 3417.3 (2573.5-4573.3) | 207434.5 (152518.4-286594.2) | 3128.3 (2346.6-4193.9) | -0.27 (-4.48-4.13) |
| Trichomoniasis | Kyrgyzstan | 149156.6 (108023.9-196247.3) | 3884.9 (2900.2-5231.5) | 266538.4 (194107.3-353311.9) | 3920.8 (2884.1-5176.1) | 0.86 (-3.29-5.19) |
| Trichomoniasis | Laos | 139095.4 (102157.1-183403) | 4351.7 (3251.6-5819.6) | 320443.2 (236946.5-423235) | 4277.3 (3199.3-5668.8) | 0.67 (-3.38-4.9) |
| Trichomoniasis | Latvia | 79939.2 (60335.3-107039.6) | 2786.8 (2070.9-3718.6) | 56709.8 (42872.7-76425.3) | 2792.8 (2059.1-3762.2) | 0.27 (-3.61-4.31) |
| Trichomoniasis | Lebanon | 77853.7 (58205-101787.8) | 2944.1 (2208.9-3914.6) | 187583.3 (137924.3-250084.8) | 2962 (2212.4-3969.5) | 0.32 (-3.65-4.46) |
| Trichomoniasis | Lesotho | 103295.7 (78576.6-132765.3) | 8765.3 (6680.3-11228.8) | 149501.1 (111932.3-196292.9) | 8390.5 (6329.3-11144.2) | -0.33 (-3.99-3.46) |
| Trichomoniasis | Liberia | 138232.8 (102003.5-182804.3) | 7421.2 (5592-9786.1) | 358745.7 (265377.1-473635.7) | 7440.3 (5586.8-9779.4) | -0.48 (-4.09-3.26) |
| Trichomoniasis | Libya | 79578.1 (59923.5-103791.3) | 2563.3 (1944.1-3365.9) | 204435.9 (152480.2-273550.1) | 2450.4 (1853.5-3212.6) | 0.15 (-3.89-4.36) |
| Trichomoniasis | Lithuania | 109326 (81844.7-144696.9) | 2790.7 (2076.1-3703) | 82109 (62974.8-110680.3) | 2790 (2094-3728) | 0.45 (-3.61-4.68) |
| Trichomoniasis | Luxembourg | 10448 (7877.6-13837.6) | 2336.6 (1760.2-3093.1) | 18039.2 (13661.4-24403) | 2359.9 (1771.3-3108.2) | -0.24 (-3.47-3.11) |
| Trichomoniasis | Macedonia | 67030.9 (50119.8-89093.2) | 3201.5 (2398.9-4251.9) | 84624.9 (63390.4-114574.7) | 3221.2 (2399.7-4328.8) | 0.06 (-4.35-4.68) |
| Trichomoniasis | Madagascar | 839482 (632229-1097487) | 9653.6 (7330.3-12611.8) | 2277687.2 (1710791.7-2963652.1) | 9667.6 (7381-12803.2) | 0.59 (-3.5-4.85) |
| Trichomoniasis | Malawi | 583602.4 (434154.9-772340) | 8300 (6282.2-11048.9) | 1268866.1 (935881.6-1661304.8) | 8378.4 (6349.8-11199.2) | -0.93 (-4.53-2.81) |
| Trichomoniasis | Malaysia | 696176.6 (512054.7-926466.3) | 4347.7 (3291.7-5688.8) | 1529302.6 (1143329.2-2027559.5) | 4301.5 (3249-5706.1) | -0.14 (-4.07-3.94) |
| Trichomoniasis | Maldives | 6752.2 (4987.6-8843.3) | 4373.3 (3269.2-5786) | 32400.6 (23399.1-43749.9) | 4462.4 (3333.9-5892.6) | 0.08 (-4.31-4.67) |
| Trichomoniasis | Mali | 573498.3 (439569-729906.1) | 9003.4 (6910.3-11622.6) | 1536093.3 (1157383.4-1994169.2) | 8969.9 (6833.7-11804.1) | -0.76 (-4.46-3.08) |
| Trichomoniasis | Malta | 9516.2 (7120.4-12521.4) | 2308.4 (1731.6-3023.9) | 11584.1 (8740.2-15551.3) | 2366.1 (1761.1-3162) | 0.67 (-2.67-4.13) |
| Trichomoniasis | Marshall Islands | 2310.4 (1691.1-3038.4) | 6871.8 (5141.3-9003.2) | 3892.5 (2907-5192.6) | 6720.4 (5086.4-8948.1) | 0.87 (-3.11-5.02) |
| Trichomoniasis | Mauritania | 116989.2 (87265.6-155334.3) | 7518 (5655.1-9902.4) | 263780 (197190.7-343575.2) | 7523.2 (5672.6-9910.3) | -0.14 (-4.38-4.29) |
| Trichomoniasis | Mauritius | 48827.3 (35406.7-64711.4) | 4329.5 (3222.9-5665.1) | 62063.2 (47198-83710) | 4271 (3189.3-5717.4) | 0.69 (-3.22-4.77) |
| Trichomoniasis | Mexico | 4648807 (3402120.1-6197318.2) | 6648.4 (4953.5-9074.4) | 9089056.7 (6737425.5-12567508.5) | 6529.8 (4854.2-8987.3) | 0.09 (-3.94-4.29) |
| Trichomoniasis | Moldova | 128861.5 (96047-170163.2) | 2778.1 (2087.5-3709.2) | 122044.2 (91431.7-165188.9) | 2774 (2068.4-3736.4) | 0.22 (-3.64-4.23) |
| Trichomoniasis | Monaco | 805.2 (620.9-1085.7) | 2328.3 (1739.5-3143.3) | 906.2 (707.2-1219.3) | 2328 (1761.7-3110.8) | -0.03 (-3.59-3.66) |
| Trichomoniasis | Mongolia | 66534.1 (48748.3-88150.6) | 3884.5 (2921.9-5095.5) | 137283.5 (101231.5-185962.8) | 3905.7 (2913.7-5234.6) | 0.37 (-4.08-5.03) |
| Trichomoniasis | Montenegro | 21120.1 (15707.1-28212.7) | 3222.8 (2406.4-4325) | 22144.7 (16719.7-30001.5) | 3244.2 (2398.1-4330.9) | 0 (-4.31-4.51) |
| Trichomoniasis | Morocco | 761157.6 (563011.7-997635.8) | 3626.8 (2717.4-4778.8) | 1409967.5 (1058154.4-1890872.1) | 3563.7 (2673.2-4759.5) | -0.27 (-4.32-3.94) |
| Trichomoniasis | Mozambique | 1181348.4 (913274.6-1492387.7) | 11725.6 (9159.2-14965.2) | 2479209.9 (1887188.1-3199980.6) | 10778.2 (8286.2-14343) | -0.21 (-3.9-3.63) |
| Trichomoniasis | Myanmar | 1540045.3 (1131221.2-2048187.7) | 4338.4 (3233.9-5840) | 2454095.4 (1838628-3308377) | 4231 (3176.7-5680.7) | -0.3 (-4.05-3.6) |
| Trichomoniasis | Namibia | 91086.4 (68690.5-117961.7) | 8307.2 (6388.5-10738.7) | 201035 (149543.1-264028.7) | 8503.3 (6423.4-11206.7) | -0.75 (-4.39-3.03) |
| Trichomoniasis | Nauru | 655 (482.4-872.2) | 7315.7 (5514.2-9782.8) | 730.2 (533.9-977) | 7045.3 (5227.6-9426.8) | 1.02 (-3.14-5.35) |
| Trichomoniasis | Nepal | 398727.1 (295321.2-522867.9) | 2593.3 (1957-3405) | 770405.7 (579558.9-1023331.3) | 2532.2 (1922.3-3398.4) | 0.14 (-3.56-3.98) |
| Trichomoniasis | Netherlands | 371254.4 (275083.1-495188) | 2167.3 (1608.5-2887.3) | 395036.6 (301457.4-526585.2) | 2163.2 (1613.5-2866) | -0.06 (-3.48-3.47) |
| Trichomoniasis | New Zealand | 103289 (77054.6-139720.1) | 2841.2 (2128.6-3868.1) | 159908.1 (121305.1-218762.3) | 2864.2 (2149.9-3898.6) | 0.13 (-3.66-4.07) |
| Trichomoniasis | Nicaragua | 182376.5 (133533.4-241138.7) | 6526.3 (4865.6-8719.9) | 436969.8 (321820.9-579838.9) | 6393.2 (4780-8462.2) | 0.45 (-3.69-4.78) |
| Trichomoniasis | Niger | 417913.9 (311252.1-543870.5) | 7476.7 (5654.2-9731.5) | 1220465.6 (913484.6-1590424.3) | 7538.6 (5687.2-9983.9) | -1.21 (-4.97-2.7) |
| Trichomoniasis | Nigeria | 5259686.9 (3954300.3-6980117.8) | 7642.5 (5806.3-10326) | 14514515.6 (10833474.9-19328437.3) | 8212.6 (6227.7-11080.1) | -0.41 (-4.07-3.4) |
| Trichomoniasis | Niue | 143.7 (108-191.5) | 7277.1 (5413.7-9754.6) | 116.9 (89.6-156.2) | 6994.9 (5269.5-9305.6) | 0.96 (-3.19-5.28) |
| Trichomoniasis | North Korea | 792894.1 (594455.7-1039184) | 3792.9 (2832.3-4967.6) | 1185397 (896531.6-1606560.9) | 3811.9 (2860.7-5060.4) | 0.61 (-3.52-4.92) |
| Trichomoniasis | Northern Mariana Islands | 3826.4 (2759-5129.3) | 6917 (5200.9-9053.2) | 3517.5 (2690-4671.7) | 6755.2 (5096-8891.9) | 0.11 (-4.12-4.52) |
| Trichomoniasis | Norway | 111855.8 (83980.2-153094.4) | 2416.7 (1812.4-3296.4) | 145613.6 (109929.3-200208.8) | 2439 (1821.4-3317.8) | -0.12 (-3.57-3.45) |
| Trichomoniasis | Oman | 64226.3 (46297.8-86254.2) | 3690.4 (2769.1-4947.7) | 220189.3 (158077.1-298636) | 3639 (2752.3-4918.4) | 0.21 (-3.9-4.5) |
| Trichomoniasis | Pakistan | 2257967 (1662519-3034725.5) | 2771.8 (2052.6-3778.6) | 5880468.2 (4331740.2-7926634.9) | 2745.2 (2039-3738) | 1.1 (-2.86-5.23) |
| Trichomoniasis | Palau | 1129.3 (826.6-1517.4) | 7198.2 (5350.3-9718.9) | 1389.3 (1045.6-1928.5) | 6567 (4914.1-8790.6) | 0.87 (-3.28-5.19) |
| Trichomoniasis | Palestine | 51695.6 (38789.1-67166.2) | 3777 (2878.6-4987.4) | 171456.3 (126997.1-226397.6) | 3750.1 (2839.5-5017.7) | 0.16 (-4.12-4.63) |
| Trichomoniasis | Panama | 138524.2 (103196.9-180682.5) | 6377.1 (4776.3-8369.4) | 277437.7 (210388.6-364818.6) | 6350 (4797.7-8368.5) | 0.21 (-3.6-4.17) |
| Trichomoniasis | Papua New Guinea | 260460.3 (194747.4-338344.3) | 7521.4 (5688.9-9768.2) | 716847.6 (534535.7-939454.7) | 7287.1 (5519.9-9536.7) | 0.53 (-3.47-4.7) |
| Trichomoniasis | Paraguay | 179945.3 (134406.6-238302.4) | 5455.4 (4146.9-7278.6) | 402775.2 (295934.6-535568.2) | 5428.1 (4041.3-7299.6) | 0.46 (-3.59-4.68) |
| Trichomoniasis | Peru | 760417.5 (567793.6-998718.2) | 4220.4 (3190-5557.5) | 1616480.2 (1211705.2-2142394.1) | 4196.6 (3166.8-5574) | 0.01 (-3.81-3.99) |
| Trichomoniasis | Philippines | 2405326 (1768668.6-3246220.1) | 4626.2 (3487.1-6314.4) | 5131304.3 (3819997.3-6951345.3) | 4529.1 (3398.7-6186.8) | 1.16 (-2.86-5.36) |
| Trichomoniasis | Poland | 1422144.3 (1058352.4-1950698.5) | 3431.5 (2572.2-4719.7) | 1555083.8 (1171713.6-2153599) | 3464.8 (2580.2-4730.7) | 0.18 (-4.01-4.55) |
| Trichomoniasis | Portugal | 254075.7 (193798.5-338099.2) | 2391.3 (1795.3-3196.8) | 278477.6 (213611.9-376638.7) | 2400.9 (1789.1-3171.3) | -0.73 (-3.96-2.61) |
| Trichomoniasis | Puerto Rico | 186806.1 (139681-249630.6) | 5166.2 (3853-6902.7) | 182132.6 (139708-240415.1) | 5195.8 (3866.5-6822.9) | -0.9 (-4.73-3.08) |
| Trichomoniasis | Qatar | 20941 (14793.6-28194.3) | 3826.7 (2919.8-5059.3) | 176014 (124885.6-239294.6) | 3794 (2901.3-5037.5) | -0.23 (-4.5-4.22) |
| Trichomoniasis | Romania | 779778.6 (589489.8-1043915.5) | 3216.2 (2380.8-4301.5) | 678055.4 (520733.6-936041.3) | 3253.1 (2442.9-4344.6) | 0.28 (-3.82-4.56) |
| Trichomoniasis | Russian Federation | 4762959.7 (3561426.6-6476413.4) | 2858.5 (2125.6-3928.9) | 4770101 (3587280.2-6556182) | 2833.1 (2110.4-3863.1) | 1.19 (-2.71-5.26) |
| Trichomoniasis | Rwanda | 503825.9 (377581-653653.7) | 9696.1 (7355.2-12844.4) | 1135836.2 (846150.1-1480727.4) | 9624.2 (7294.2-12726.7) | -0.97 (-4.64-2.84) |
| Trichomoniasis | Saint Kitts and Nevis | 1853.3 (1352.9-2443.7) | 5298.4 (3913.6-7189.8) | 3720.7 (2805.3-5092.6) | 5250.3 (3924.8-7087.2) | -0.03 (-3.79-3.88) |
| Trichomoniasis | Saint Lucia | 5696.2 (4198.9-7515.9) | 5137.1 (3833.3-6875.1) | 10788.2 (8156.3-14781.6) | 5163.2 (3898.4-6919.9) | -0.41 (-4.16-3.49) |
| Trichomoniasis | Saint Vincent and the Grenadines | 4487.6 (3328.9-5833.7) | 5105.3 (3869.5-6729.6) | 6299.2 (4779.1-8441) | 5147.4 (3846.6-6856.3) | -0.51 (-4.33-3.47) |
| Trichomoniasis | Samoa | 8995.3 (6705.1-11728.7) | 6976.4 (5228.3-9177.5) | 12672.5 (9533.8-16753.1) | 6816.7 (5125.3-9056.2) | 0.97 (-3.28-5.41) |
| Trichomoniasis | San Marino | 618.3 (468.8-831.8) | 2349.9 (1760.6-3173.3) | 805.7 (626.4-1100.4) | 2297.3 (1731.8-3087) | -0.07 (-3.62-3.61) |
| Trichomoniasis | Sao Tome and Principe | 6066.5 (4616.4-7898.4) | 7365.4 (5650.8-9675.2) | 14892.9 (11178.1-19666.8) | 7424.6 (5651.5-9861.7) | -0.49 (-4.64-3.84) |
| Trichomoniasis | Saudi Arabia | 636485.1 (466836.2-845031.1) | 4907.4 (3738.7-6558.1) | 2435472.9 (1776246.6-3380283.3) | 4730.6 (3566.2-6471.4) | -0.03 (-4.22-4.34) |
| Trichomoniasis | Senegal | 405549 (304200.7-526287.1) | 7487.9 (5694-9918.6) | 984222.2 (733521.7-1282321.1) | 7462.8 (5642.4-9911.8) | -0.7 (-4.3-3.04) |
| Trichomoniasis | Serbia | 335910.7 (254419.4-451187.1) | 3220.1 (2417.6-4305) | 324610.6 (244300.4-434074.8) | 3231 (2378.5-4294.4) | 0 (-4.35-4.55) |
| Trichomoniasis | Seychelles | 2869.2 (2124.4-3774.5) | 4337.8 (3273.3-5605.2) | 5236 (3910.9-7079.6) | 4344.4 (3252.7-5780.1) | -0.26 (-4.28-3.94) |
| Trichomoniasis | Sierra Leone | 244912.1 (183023.8-318192.8) | 7397.9 (5630.3-9691.7) | 559265.9 (420276.7-736569.9) | 7464.2 (5725.7-9817.7) | -0.21 (-3.84-3.55) |
| Trichomoniasis | Singapore | 106140 (77536.6-139980.3) | 2909.8 (2147.5-3817.6) | 217695.9 (161425.1-296521.6) | 2939.6 (2192.7-3872.3) | -0.46 (-3.96-3.18) |
| Trichomoniasis | Slovakia | 179501.6 (133189.1-237795.2) | 3207.4 (2390.8-4242.9) | 205833.8 (157724.2-284336.4) | 3245.2 (2430.2-4315.6) | 0.05 (-4.36-4.66) |
| Trichomoniasis | Slovenia | 71144.4 (53413.5-95202.6) | 3231 (2415.5-4316.7) | 74258.4 (56624.8-100738.8) | 3254.4 (2424.8-4317.4) | -0.2 (-4.32-4.1) |
| Trichomoniasis | Solomon Islands | 17378.7 (12973-22507.1) | 6963.6 (5204.4-9041.4) | 41910.8 (31290.4-54812.7) | 6760.1 (5118.1-8815.1) | 0.94 (-3.15-5.19) |
| Trichomoniasis | Somalia | 566287.2 (423390.3-739186.1) | 9729.5 (7455.7-12649) | 1511579.6 (1118438.5-1998751.7) | 9664.1 (7363-12943.1) | -0.55 (-4.47-3.53) |
| Trichomoniasis | South Africa | 3107166.2 (2314660.6-4145192.9) | 9421.2 (7036-12615.6) | 5367237.9 (3933951.1-7299150.5) | 8515.6 (6317.2-11601.7) | -0.52 (-4.33-3.44) |
| Trichomoniasis | South Korea | 1410652.2 (1015516.8-1856540.2) | 2919.8 (2166.8-3827.2) | 1814085.4 (1391789.5-2456372) | 2941 (2223.3-3924.6) | 0.02 (-3.88-4.08) |
| Trichomoniasis | South Sudan | 420170.7 (318552-544099.7) | 9850.3 (7517.1-12979.7) | 717438.4 (544248.7-950522.3) | 9785 (7372.7-13026) | -0.09 (-3.83-3.8) |
| Trichomoniasis | Spain | 939856.4 (708719.7-1241490.1) | 2316.1 (1721.4-3082.4) | 1193871.8 (916373-1654766.8) | 2335.5 (1743.5-3117.3) | -0.6 (-3.89-2.82) |
| Trichomoniasis | Sri Lanka | 846874.8 (632625.4-1121665) | 5027.1 (3812.7-6537.7) | 1146082.2 (880729.9-1521780.4) | 4853.3 (3678.9-6360.7) | 0.01 (-4.41-4.63) |
| Trichomoniasis | Sudan | 617449.6 (466773.1-806502) | 4169.9 (3194.2-5531) | 1519237.7 (1137731.2-2016712.3) | 4065.1 (3079.8-5398.2) | 0.13 (-3.67-4.08) |
| Trichomoniasis | Suriname | 17830.3 (13205.6-23564.4) | 5168.4 (3885.6-6847.1) | 31593.1 (23695-42812) | 5188.6 (3849.1-6962.4) | -0.09 (-3.89-3.85) |
| Trichomoniasis | Swaziland | 50101.8 (37625.5-64633.1) | 8614.7 (6578.6-11185.9) | 93997.9 (68897.2-123124.5) | 8529.1 (6398.9-11205.3) | -0.24 (-3.91-3.57) |
| Trichomoniasis | Sweden | 224291 (169742.8-310539.4) | 2376.6 (1779.5-3255) | 267034.9 (202631.3-363729.1) | 2422.2 (1808.1-3292.2) | -0.15 (-3.64-3.47) |
| Trichomoniasis | Switzerland | 188376.3 (141080.6-249773.6) | 2353.7 (1749.2-3145.2) | 238197.3 (182829-317479.2) | 2365 (1765-3119.4) | -0.79 (-4.08-2.62) |
| Trichomoniasis | Syria | 302676.6 (224148.5-401106.4) | 3481 (2614.3-4728.4) | 470515.5 (365683-661176.7) | 3368 (2533.8-4534.3) | 0 (-4.28-4.47) |
| Trichomoniasis | Taiwan | 827874.5 (630079.7-1077666) | 3808 (2937.4-4938.4) | 1085333.5 (826764.5-1443899.2) | 3817.1 (2853.9-5061.9) | 0.43 (-3.63-4.67) |
| Trichomoniasis | Tajikistan | 161042.2 (119668-212761) | 3918.9 (2936.3-5197.2) | 383452.9 (281237.2-505084.7) | 3914.3 (2956.2-5131.7) | 0.14 (-4.11-4.59) |
| Trichomoniasis | Tanzania | 2162868.2 (1635901.2-2808631.9) | 11924.8 (9102.8-15863.7) | 5391611.9 (4052706.5-7082186.7) | 11535.6 (8749.7-15417.4) | -1.24 (-4.97-2.64) |
| Trichomoniasis | Thailand | 2503637.2 (1848102-3338750.9) | 4364.1 (3261.4-5822.7) | 3309861.7 (2531327.1-4448650.3) | 4262.2 (3177.3-5638.7) | -0.72 (-4.45-3.15) |
| Trichomoniasis | The Bahamas | 13028 (9378.6-17151.1) | 5204.1 (3850.5-7045.8) | 22811.9 (17238.9-30933) | 5197.8 (3931-7012.1) | -0.3 (-4.02-3.56) |
| Trichomoniasis | The Gambia | 54332.5 (40132-71281.6) | 7432.7 (5608.6-9834) | 147410.3 (108578.8-194366.7) | 7488.8 (5564.3-9993.5) | 0.14 (-3.42-3.83) |
| Trichomoniasis | Timor-Leste | 28975.7 (21058.2-38287.2) | 4412.1 (3283.5-5789.4) | 49049.9 (36372.2-65267.2) | 4284 (3172.5-5807.1) | -0.2 (-4.01-3.76) |
| Trichomoniasis | Togo | 164128.3 (121789.9-217730.1) | 6335.7 (4736.9-8377.3) | 474015.2 (347348.7-621782) | 6460.3 (4803.9-8449.1) | -0.96 (-4.47-2.68) |
| Trichomoniasis | Tokelau | 95.9 (72-126.3) | 7442.8 (5592.2-9931.1) | 91.2 (69.6-120.8) | 6924.4 (5238.6-9153.1) | 0.84 (-3.31-5.17) |
| Trichomoniasis | Tonga | 5271.2 (3998.8-6929.7) | 7099.2 (5303.7-9494.2) | 6324 (4820.9-8375.4) | 6929.1 (5267.5-9182.1) | 1.02 (-3.16-5.37) |
| Trichomoniasis | Trinidad and Tobago | 58314.7 (42530.8-78341.8) | 5122 (3804.3-6955.4) | 82243.1 (61788.6-111153.1) | 5174.7 (3882.7-6922) | -0.02 (-3.72-3.83) |
| Trichomoniasis | Tunisia | 277661.9 (208948-360726.2) | 3955 (3005.2-5215) | 511838.6 (392589.2-696257.7) | 3838.1 (2918-5122.3) | 0.28 (-3.98-4.72) |
| Trichomoniasis | Turkey | 1527363.3 (1142159.2-2005794.5) | 3042.9 (2308.5-4025.9) | 2824223.4 (2117327.2-3830486.5) | 2997.6 (2230.8-4016.9) | -0.04 (-4.32-4.44) |
| Trichomoniasis | Turkmenistan | 117741.8 (85386.1-155215.5) | 3875.4 (2869.3-5142.4) | 206355.4 (153094.9-275483.9) | 3881.9 (2901.2-5183.7) | -0.39 (-4.59-4) |
| Trichomoniasis | Tuvalu | 661.6 (493.3-884.5) | 7603.1 (5736.1-10280.5) | 817 (606.2-1083.7) | 6806.3 (5060.9-9133.3) | 0.84 (-3.32-5.17) |
| Trichomoniasis | Uganda | 917864.9 (702813.6-1190309) | 7778.8 (5905.5-10070.3) | 2490884 (1871098.7-3237206.1) | 7834.3 (5987-10360.2) | -0.66 (-4.3-3.11) |
| Trichomoniasis | Ukraine | 1616329.6 (1192689.7-2211879.5) | 2841.6 (2070.4-3912.1) | 1477051.9 (1112291.7-2078917.4) | 2858.5 (2139.5-4002.4) | 0.33 (-3.48-4.29) |
| Trichomoniasis | United Arab Emirates | 83477.3 (58413.2-112832.8) | 3845.3 (2883.8-5186.2) | 638869.5 (458708.7-949554.1) | 3828.3 (2907.6-5239.3) | 0.15 (-4.02-4.5) |
| Trichomoniasis | United Kingdom | 1464549.4 (1107168-1972871.6) | 2360.4 (1761.7-3185.9) | 1733023.6 (1316135.7-2349831.1) | 2352.6 (1764-3175.9) | 0 (-3.29-3.4) |
| Trichomoniasis | United States | 12081316.2 (8986589.5-16448809.7) | 4269.1 (3197.9-5810) | 14662468.3 (11033481.6-20182614.9) | 4183.8 (3124.4-5779.7) | -0.15 (-3.67-3.5) |
| Trichomoniasis | Uruguay | 86839.4 (65890.7-115302.9) | 2803 (2122.2-3741.4) | 102579.3 (77798.2-137759.8) | 2827.6 (2131.6-3759.2) | 0.16 (-3.27-3.71) |
| Trichomoniasis | Uzbekistan | 659639.9 (479780.8-867480.4) | 3880.7 (2888.5-5197) | 1425099.4 (1047780.7-1890110.5) | 3894.6 (2901.7-5128.9) | 0.53 (-3.58-4.81) |
| Trichomoniasis | Vanuatu | 8528.4 (6350-11131.8) | 6989.2 (5223.5-9208.1) | 19448.5 (14461.6-25476.9) | 6826 (5097.6-8976.4) | 0.92 (-3.27-5.3) |
| Trichomoniasis | Venezuela | 1078553.5 (787742.6-1410944.9) | 6442.9 (4800.9-8489.4) | 1842918 (1381883.1-2494288.8) | 6447.6 (4779.5-8473.7) | 0.25 (-3.64-4.3) |
| Trichomoniasis | Vietnam | 2170167 (1592310.6-2856444.9) | 3851.3 (2880.5-5034.9) | 4337365.8 (3230305.3-5848213) | 3817.4 (2868.3-5090.3) | 0.32 (-3.54-4.34) |
| Trichomoniasis | Yemen | 302047.8 (223395.3-396124.2) | 3448.4 (2626.5-4533) | 944623.9 (696373.6-1270363.5) | 3386.8 (2561-4580.3) | 0.51 (-3.57-4.77) |
| Trichomoniasis | Zambia | 609755.8 (464272.1-777081.4) | 11063.3 (8570.4-14356.7) | 1759215.4 (1323131.8-2273063.3) | 11018.3 (8495-14582.6) | -0.88 (-4.51-2.9) |
| Trichomoniasis | Zimbabwe | 663484.1 (498065.9-845593.3) | 8833.5 (6752.6-11487.8) | 1214151.2 (917880.9-1570778) | 8989.3 (6852.6-11649.4) | -1.3 (-4.89-2.44) |
| HIV/AIDS and sexually transmitted infections | Afghanistan | 579394.4 (490763.3-690800.4) | 7854.1 (6627-9476) | 2108940.9 (1758487.7-2517931.3) | 7897.3 (6673.8-9497.5) | 0.04 (0.03-0.05) |
| HIV/AIDS and sexually transmitted infections | Albania | 300816.5 (246724.5-361735.9) | 8900 (7419.3-10561.1) | 237359.9 (199197.6-285316.8) | 8711.4 (7235.1-10523.4) | -0.11 (-0.13--0.09) |
| HIV/AIDS and sexually transmitted infections | Algeria | 1689250.1 (1418793.9-2011436.7) | 7834.9 (6606.1-9419.8) | 3403651.5 (2801333-4147854) | 7339 (6124.4-8936.2) | -0.04 (-0.1-0.03) |
| HIV/AIDS and sexually transmitted infections | American Samoa | 5474.2 (4565-6560.2) | 11929.7 (10027.7-14559.5) | 5598.8 (4687.2-6768.5) | 11645.2 (9676.8-14215.8) | -0.03 (-0.05--0.01) |
| HIV/AIDS and sexually transmitted infections | Andorra | 2225.1 (1772.6-2769.3) | 3348.4 (2702-4160.4) | 3277.2 (2696.6-4235.9) | 3276.4 (2655.4-4106) | -0.09 (-0.12--0.07) |
| HIV/AIDS and sexually transmitted infections | Angola | 1035388.7 (887946.3-1213429.8) | 11934.5 (10290.9-14130.5) | 3172299.1 (2703026.6-3684507.7) | 11634 (9899.6-13709.3) | -0.11 (-0.13--0.09) |
| HIV/AIDS and sexually transmitted infections | Antigua and Barbuda | 7225 (6033-8715.2) | 11847 (9855.4-14424.2) | 11795.6 (9869.9-14451.4) | 11793.6 (9839.6-14427.5) | -0.01 (-0.01-0) |
| HIV/AIDS and sexually transmitted infections | Argentina | 1575147.9 (1329111.7-1881186.7) | 4935 (4153.9-5908.9) | 2403955.3 (2021802.4-2920531.4) | 4959.5 (4180.2-6024.4) | 0.02 (0-0.04) |
| HIV/AIDS and sexually transmitted infections | Armenia | 424100.4 (349628.1-512855.1) | 11968.5 (9959-14446.4) | 362730.4 (303128.5-443965.9) | 11590.7 (9670.4-14098.1) | -0.17 (-0.2--0.15) |
| HIV/AIDS and sexually transmitted infections | Australia | 831222.1 (686524.2-1012677.2) | 4555.6 (3792.2-5519.7) | 1196857 (994034.4-1471161.9) | 4449.3 (3694.9-5398.8) | -0.06 (-0.12--0.01) |
| HIV/AIDS and sexually transmitted infections | Austria | 274397.4 (221562.9-340610.2) | 3209.6 (2594.2-3995.7) | 310838 (253483-396750.4) | 3205.2 (2586-4048.9) | -0.01 (-0.03-0) |
| HIV/AIDS and sexually transmitted infections | Azerbaijan | 908783.9 (740743.3-1094745.7) | 12177.2 (10186.8-14562.3) | 1388592.8 (1155506.5-1687900.1) | 11760.2 (9805.3-14153.7) | -0.17 (-0.19--0.15) |
| HIV/AIDS and sexually transmitted infections | Bahamas | 32698.2 (26986.1-39374.5) | 12055.3 (10077.2-14642.9) | 51110.7 (42293-61962) | 11885.2 (9821.1-14402.5) | -0.02 (-0.03--0.01) |
| HIV/AIDS and sexually transmitted infections | Bahrain | 47923.1 (38936.1-58804.9) | 8113.4 (6740.7-9815.6) | 160408.6 (133484.8-196523.5) | 8045.2 (6763.5-9739.7) | -0.01 (-0.02-0) |
| HIV/AIDS and sexually transmitted infections | Bangladesh | 5341868.9 (4531785.6-6300261.1) | 5850.9 (4949.3-6994.1) | 9769236.8 (8200288.3-11746337.5) | 5629.3 (4736.6-6748.4) | -0.19 (-0.21--0.17) |
| HIV/AIDS and sexually transmitted infections | Barbados | 35546.1 (29506.2-43347.3) | 13231.3 (11036.9-16036.8) | 40193.6 (33689.1-49558) | 13181.5 (10947-16167.1) | -0.02 (-0.08-0.04) |
| HIV/AIDS and sexually transmitted infections | Belarus | 975082.1 (814457-1179786.5) | 9071.7 (7594.2-10919) | 824639.8 (692438.1-1012594.6) | 8812.9 (7385.6-10647.9) | -0.13 (-0.14--0.11) |
| HIV/AIDS and sexually transmitted infections | Belgium | 341876.3 (279242.3-427043.5) | 3153 (2592.7-3952.6) | 370278.3 (303088.9-464596.2) | 3145.2 (2554-3891) | -0.05 (-0.13-0.03) |
| HIV/AIDS and sexually transmitted infections | Belize | 18273.1 (15171-21875.4) | 11814 (9806.7-14223.5) | 53620.5 (45041.8-65475.1) | 11785.9 (9827.2-14452.9) | -0.01 (-0.01-0) |
| HIV/AIDS and sexually transmitted infections | Benin | 382847.1 (322045.6-454115.7) | 10406.9 (8736.4-12599.7) | 1189486.7 (999944.4-1429761.6) | 10523.6 (8795-12873.9) | 0.14 (0.03-0.25) |
| HIV/AIDS and sexually transmitted infections | Bermuda | 8309.4 (6883.2-10165.1) | 11747.8 (9819.6-14275.4) | 7289.5 (6143.6-8906.4) | 11651.6 (9748.1-14189.4) | -0.01 (-0.02--0.01) |
| HIV/AIDS and sexually transmitted infections | Bhutan | 32031.7 (27120-37796.3) | 5762.5 (4900-6892.7) | 48835.8 (40975.2-58170.8) | 5643.9 (4782.1-6721.3) | -0.09 (-0.1--0.08) |
| HIV/AIDS and sexually transmitted infections | Bolivia (Plurinational State of) | 454442.3 (376844.5-541635.2) | 8274.6 (6909.9-9971.8) | 1005484.6 (833917.1-1222427.4) | 8195.9 (6803.6-10002.8) | -0.05 (-0.07--0.04) |
| HIV/AIDS and sexually transmitted infections | Bosnia and Herzegovina | 429674.6 (359706.3-517270.5) | 8703.7 (7292.5-10463.1) | 280565.3 (236637.1-335183.8) | 8620.8 (7168.9-10291.7) | -0.05 (-0.05--0.04) |
| HIV/AIDS and sexually transmitted infections | Botswana | 199612 (173457.1-228925.4) | 17533.4 (15269.9-20239.5) | 411992.6 (349024.7-488920.7) | 15404.9 (13148.2-18336.1) | -0.72 (-0.83--0.61) |
| HIV/AIDS and sexually transmitted infections | Brazil | 17033687.4 (14186494.6-20753078.7) | 11797.8 (9810.6-14467.9) | 28720121.7 (23813466.7-35393228.5) | 11778.7 (9823.8-14504.1) | -0.08 (-0.14--0.03) |
| HIV/AIDS and sexually transmitted infections | Brunei Darussalam | 13664.7 (11168.2-16683.8) | 5024.6 (4193.9-6088.2) | 27628.3 (22922.1-34085.3) | 5014.6 (4193.6-6106) | -0.01 (-0.03-0.01) |
| HIV/AIDS and sexually transmitted infections | Bulgaria | 791507 (664044-958423.7) | 9066.7 (7451.7-11006.3) | 582095.2 (489520.5-718170.8) | 9011.7 (7489.9-10914.2) | -0.1 (-0.13--0.06) |
| HIV/AIDS and sexually transmitted infections | Burkina Faso | 1045147.7 (877710.4-1236617.5) | 14734.1 (12370.5-17636.4) | 2427571.5 (2014290.1-2930271.5) | 12988.3 (10780.6-15874.9) | -0.53 (-0.63--0.43) |
| HIV/AIDS and sexually transmitted infections | Burundi | 737678.6 (616832.8-880603.8) | 16558.8 (14003.8-19893.8) | 1794216.1 (1505457.8-2149350) | 15919 (13300.3-19160.6) | -0.12 (-0.16--0.09) |
| HIV/AIDS and sexually transmitted infections | Cabo Verde | 31600.3 (26434.1-37516.1) | 11706.1 (9738.8-14284.8) | 72792.7 (60248.7-88544.2) | 11652.4 (9801.1-14234.3) | -0.06 (-0.09--0.02) |
| HIV/AIDS and sexually transmitted infections | Cambodia | 922813.8 (771528.8-1109829.2) | 10792.7 (9019.9-13125.9) | 1926791.8 (1596062.2-2358107.8) | 10679.4 (8960.9-13047.6) | -0.06 (-0.09--0.04) |
| HIV/AIDS and sexually transmitted infections | Cameroon | 1208149.5 (1031861.1-1424755.9) | 14235.5 (12095.4-16986.1) | 3944651.1 (3320397.4-4649804.8) | 13830.2 (11656.1-16621.4) | -0.34 (-0.42--0.25) |
| HIV/AIDS and sexually transmitted infections | Canada | 1551182.4 (1218828.3-1975971) | 5026 (3992.4-6372.6) | 1999614.9 (1588443.4-2557373.6) | 5150.9 (4085.7-6535.5) | 0.09 (0.04-0.14) |
| HIV/AIDS and sexually transmitted infections | Central African Republic | 317851.7 (275307.4-367650.4) | 13527.1 (11649.1-15715.1) | 603593.4 (519305.3-711516.6) | 12126.2 (10397.3-14434.1) | -0.23 (-0.28--0.19) |
| HIV/AIDS and sexually transmitted infections | Chad | 550758 (462957.4-655661.6) | 12069.2 (10102.5-14608.2) | 1557744.7 (1310439.3-1871594.1) | 11918.9 (10050.4-14427.3) | -0.09 (-0.12--0.06) |
| HIV/AIDS and sexually transmitted infections | Chile | 662954.3 (553759.2-799695.2) | 4867.8 (4091.1-5878.7) | 1004490.9 (841502.8-1220031) | 4908.9 (4119.2-5924.7) | -0.01 (-0.02-0.01) |
| HIV/AIDS and sexually transmitted infections | China | 123888890.3 (103510224.8-153576543.9) | 9824.9 (8166.3-12188.1) | 148305369.2 (122504280.2-184642864.7) | 9639.4 (7984.4-12048.2) | -0.31 (-0.48--0.15) |
| HIV/AIDS and sexually transmitted infections | Colombia | 3502269.8 (2885901.8-4221964.2) | 11092.5 (9224.9-13469.1) | 5936910.3 (4934712.9-7268261.5) | 11065.3 (9193.6-13571.5) | 0.02 (-0.02-0.06) |
| HIV/AIDS and sexually transmitted infections | Comoros | 58534.9 (49643.2-70271.4) | 15926.3 (13488.9-19356.8) | 118611.6 (98404.6-144173.6) | 15789 (13122.8-19165.8) | -0.03 (-0.06-0) |
| HIV/AIDS and sexually transmitted infections | Congo | 246928.9 (213512.9-284932.8) | 12071.8 (10399.8-14194.4) | 622155.1 (526908.7-738256.2) | 11569.7 (9767.1-13711.2) | -0.1 (-0.12--0.09) |
| HIV/AIDS and sexually transmitted infections | Cook Islands | 2154.2 (1780.8-2612.8) | 11982.5 (9887.9-14695.5) | 2071 (1741.5-2510.9) | 12083.2 (10041.6-14707) | 0.09 (0.07-0.12) |
| HIV/AIDS and sexually transmitted infections | Costa Rica | 343711.2 (283271.2-413788.7) | 11769.5 (9801.2-14296.2) | 614897.3 (505621.2-754761) | 11730.4 (9656.9-14338) | 0.02 (0-0.04) |
| HIV/AIDS and sexually transmitted infections | C么te d'Ivoire | 1312583.4 (1108158.1-1548875.9) | 12804.4 (10847.9-15185.1) | 2997301.5 (2508144.3-3634692.7) | 11786.8 (9916.1-14386) | -0.26 (-0.31--0.2) |
| HIV/AIDS and sexually transmitted infections | Croatia | 465316.9 (388856-557837.9) | 9011.8 (7538.4-10822.7) | 361797 (305575.4-437286.2) | 8960.6 (7462-10889.9) | 0.03 (-0.01-0.06) |
| HIV/AIDS and sexually transmitted infections | Cuba | 1383616.8 (1158148.9-1666880) | 11673.6 (9716.5-14069.2) | 1346005.5 (1127332.2-1630696.5) | 11633.8 (9644.2-14044.1) | 0 (0-0) |
| HIV/AIDS and sexually transmitted infections | Cyprus | 26550.3 (21409.6-32952.1) | 3237.6 (2613.6-4020.3) | 51486.7 (41697.3-65367) | 3210.6 (2632.3-3985.3) | -0.05 (-0.07--0.04) |
| HIV/AIDS and sexually transmitted infections | Czechia | 909874.3 (755976.3-1098150.4) | 8618.2 (7196.8-10313.7) | 867586.4 (727424.3-1053791.9) | 8518.7 (7090.5-10255.6) | -0.04 (-0.04--0.03) |
| HIV/AIDS and sexually transmitted infections | Democratic People's Republic of Korea | 2149504.9 (1762314.4-2640146.5) | 10035 (8218.1-12371) | 2978565.9 (2474687.6-3711446.8) | 9990 (8257.7-12379.6) | 0.01 (0-0.01) |
| HIV/AIDS and sexually transmitted infections | Democratic Republic of the Congo | 3664566.2 (3153828.1-4274144.1) | 11681.4 (10084.5-13778.3) | 9187784.2 (7769863.5-10846619.7) | 11438.3 (9640.2-13711.9) | -0.1 (-0.11--0.09) |
| HIV/AIDS and sexually transmitted infections | Denmark | 196101.6 (160485.4-244140.5) | 3465.7 (2837.3-4271.3) | 204221.6 (168822.4-252957.9) | 3425.1 (2805.2-4212.8) | -0.05 (-0.06--0.03) |
| HIV/AIDS and sexually transmitted infections | Djibouti | 58291.8 (48877.9-69708.9) | 15973.5 (13324.7-19472) | 212632.9 (177005.3-259337.3) | 15956.7 (13326.7-19374.2) | -0.06 (-0.1--0.03) |
| HIV/AIDS and sexually transmitted infections | Dominica | 7906.9 (6684.9-9499.5) | 11894.7 (9949.7-14359.1) | 8387.5 (7000.9-10154.3) | 11872.3 (9849.9-14373.4) | 0.01 (0.01-0.02) |
| HIV/AIDS and sexually transmitted infections | Dominican Republic | 814202.6 (683572.3-976336.6) | 12098.6 (10169.6-14723.4) | 1393794.8 (1152113.5-1679660.6) | 11881 (9825.8-14294.6) | -0.05 (-0.06--0.03) |
| HIV/AIDS and sexually transmitted infections | Ecuador | 703094.9 (582368.3-850063.2) | 7788.9 (6434.5-9525.4) | 1442083.8 (1193814.9-1756547.6) | 7732.9 (6416.7-9411.2) | -0.02 (-0.03--0.01) |
| HIV/AIDS and sexually transmitted infections | Egypt | 4424574.6 (3722099.4-5301954.5) | 8744.2 (7402.4-10529.4) | 8671689.6 (7305059.5-10415740.7) | 8320.8 (7014.3-9979.1) | -0.32 (-0.38--0.26) |
| HIV/AIDS and sexually transmitted infections | El Salvador | 541255.1 (452966-655135.7) | 11909.4 (9917.6-14614.7) | 772465.5 (642282.2-934719.8) | 11819.9 (9799.8-14418.3) | 0 (-0.02-0.03) |
| HIV/AIDS and sexually transmitted infections | Equatorial Guinea | 39998 (34871-46876.7) | 11750 (10184.7-13869.2) | 180310.4 (155015.6-212138.1) | 12140.4 (10398.7-14420.5) | 0.12 (0.1-0.15) |
| HIV/AIDS and sexually transmitted infections | Eritrea | 441486.9 (372222.9-525862.4) | 16024.4 (13505.1-19382.5) | 983862.4 (812849.8-1187204.3) | 15581.2 (13005.2-18960.9) | -0.09 (-0.12--0.06) |
| HIV/AIDS and sexually transmitted infections | Estonia | 145460.7 (121682.4-175865.8) | 9105.5 (7608.2-11025.4) | 110187.5 (92686.6-134616.2) | 8760.1 (7335.2-10678.9) | -0.12 (-0.13--0.11) |
| HIV/AIDS and sexually transmitted infections | Eswatini | 110640.4 (94719.6-129630.8) | 16652.1 (14262.1-19629.6) | 195426.6 (168144.1-231490.3) | 16518.6 (14226.3-19578.1) | -0.24 (-0.39--0.09) |
| HIV/AIDS and sexually transmitted infections | Ethiopia | 6254451.7 (5209222.2-7477395.1) | 16328.9 (13658.9-19899.5) | 14606367 (12019966.8-17783376.8) | 15450.3 (12771.4-19078.1) | -0.25 (-0.3--0.2) |
| HIV/AIDS and sexually transmitted infections | Fiji | 109056.8 (94319-127400.1) | 14298.1 (12385.9-16748) | 123517.8 (103826.4-148973.6) | 13055 (10983.8-15723.5) | -0.18 (-0.26--0.1) |
| HIV/AIDS and sexually transmitted infections | Finland | 188330.5 (152022.4-234729.7) | 3369.3 (2745.6-4138.2) | 183945.7 (151611.6-228662.1) | 3299.9 (2683-4096.8) | -0.06 (-0.09--0.03) |
| HIV/AIDS and sexually transmitted infections | France | 2129506.4 (1751307.6-2644628.7) | 3446.7 (2822.7-4279.3) | 2238961.7 (1858036.1-2860344.7) | 3393.6 (2742-4229.3) | -0.07 (-0.08--0.05) |
| HIV/AIDS and sexually transmitted infections | Gabon | 100432.5 (86785.4-116596.4) | 11856.5 (10180.1-13936.6) | 204075.4 (173422.4-241609.5) | 11484.3 (9789.7-13824) | -0.26 (-0.32--0.21) |
| HIV/AIDS and sexually transmitted infections | Gambia | 88796.6 (73475.5-106864.8) | 11144.8 (9216.8-13518.2) | 237200 (197471.9-286046) | 11164.3 (9277.4-13703.1) | -0.04 (-0.07--0.01) |
| HIV/AIDS and sexually transmitted infections | Georgia | 667061.7 (556649.9-804093.1) | 11810.3 (9883.6-14312.4) | 401173.5 (338029.1-481939.1) | 11568.1 (9622.3-13924.2) | -0.1 (-0.11--0.08) |
| HIV/AIDS and sexually transmitted infections | Germany | 3198968.9 (2634278.5-3961842.7) | 3592.1 (2971.6-4436.6) | 3090664.5 (2561495-3818069.7) | 3530.1 (2899.4-4331.3) | -0.04 (-0.09-0.01) |
| HIV/AIDS and sexually transmitted infections | Ghana | 1800671.7 (1529899-2155375.8) | 14092.1 (12025.7-16856) | 4637016.9 (3885159.3-5544744.7) | 13917.8 (11744.2-16702.4) | -0.04 (-0.06--0.02) |
| HIV/AIDS and sexually transmitted infections | Greece | 429706.6 (354792.6-532471.8) | 3922.2 (3196.9-4871.6) | 411245.3 (336613-512475.4) | 3905.3 (3201-4799.7) | 0.04 (-0.08-0.15) |
| HIV/AIDS and sexually transmitted infections | Greenland | 3418.2 (2722.3-4253.8) | 5196.3 (4184.5-6364.7) | 3212 (2594.1-4005.4) | 5351.1 (4332.3-6638.7) | 0.07 (0.02-0.11) |
| HIV/AIDS and sexually transmitted infections | Grenada | 8901.2 (7496.5-10652.6) | 11957.3 (9999.9-14392.4) | 13115.3 (11021.6-15873.2) | 11863.6 (9926.6-14457) | 0 (-0.01-0) |
| HIV/AIDS and sexually transmitted infections | Guam | 17185.2 (14166.9-20926.8) | 11438.8 (9502.7-13909) | 18089.7 (15098.6-21935.4) | 11454 (9513-13973.6) | 0.02 (0-0.05) |
| HIV/AIDS and sexually transmitted infections | Guatemala | 787160.6 (663423.5-933040) | 12075.7 (10192-14530.1) | 1879292.1 (1553346.2-2245009.8) | 11838.4 (9777-14368.2) | -0.02 (-0.03-0) |
| HIV/AIDS and sexually transmitted infections | Guinea | 574097.3 (480846.6-683154.4) | 12027.6 (10013.2-14424.4) | 1334964.1 (1118536.7-1603119.6) | 11948.8 (9988.1-14573.4) | -0.05 (-0.08--0.02) |
| HIV/AIDS and sexually transmitted infections | Guinea-Bissau | 93821.5 (78300.8-112591.5) | 11831.5 (9857.6-14355.4) | 218356.4 (181439-263290) | 11969.5 (9989.5-14548.1) | -0.01 (-0.04-0.02) |
| HIV/AIDS and sexually transmitted infections | Guyana | 89380.4 (74104.6-107693.6) | 11992.7 (9946.9-14523.1) | 94340.3 (78617.2-114353.2) | 11891.1 (9903.4-14460.9) | -0.02 (-0.03--0.02) |
| HIV/AIDS and sexually transmitted infections | Haiti | 652989.5 (551452.2-781760.6) | 11747.3 (9924.3-14058.8) | 1481262.8 (1235845.9-1801869.9) | 11214.1 (9396.8-13652.2) | -0.09 (-0.11--0.08) |
| HIV/AIDS and sexually transmitted infections | Honduras | 451477.3 (373748.6-538650.1) | 12138.2 (10062.4-14772) | 1225365.2 (1021224-1482670.9) | 12001.2 (10024.3-14739) | 0.01 (-0.01-0.03) |
| HIV/AIDS and sexually transmitted infections | Hungary | 912964.5 (772254.5-1096239.5) | 8645.6 (7182.7-10352.2) | 795454.4 (667170.5-963232.9) | 8500.4 (7114.2-10241.4) | -0.03 (-0.04--0.02) |
| HIV/AIDS and sexually transmitted infections | Iceland | 8683.9 (7021.9-10795.8) | 3273.4 (2652.4-4082) | 12157.5 (9922-15318.7) | 3299 (2692.9-4133.2) | 0.01 (-0.02-0.03) |
| HIV/AIDS and sexually transmitted infections | India | 49951873.1 (42054624.1-60349172.5) | 6267.2 (5347.9-7531) | 94425747.2 (80251291.2-113642338.2) | 6143 (5242.1-7383.2) | -0.1 (-0.12--0.07) |
| HIV/AIDS and sexually transmitted infections | Indonesia | 19542786.9 (16345475.6-24012699.3) | 11079.9 (9289-13615) | 34123344.1 (28577342.2-42313833.6) | 10887.3 (9124.7-13382.1) | -0.02 (-0.04--0.01) |
| HIV/AIDS and sexually transmitted infections | Iran (Islamic Republic of) | 4137811.8 (3460685.7-5032363.6) | 8952.1 (7505.4-10984.1) | 8717576.1 (7281159.5-10803050.3) | 8698.6 (7339-10672.2) | 0.02 (-0.15-0.18) |
| HIV/AIDS and sexually transmitted infections | Iraq | 1525599 (1299792.2-1795018.2) | 9793.5 (8361.4-11623) | 3662372.6 (3056887.2-4448136.6) | 8635.6 (7234.1-10448.5) | -0.53 (-0.63--0.42) |
| HIV/AIDS and sexually transmitted infections | Ireland | 116618.1 (95315.7-145681.2) | 3298.7 (2687.5-4147.4) | 169419.3 (138721.9-215827.6) | 3267.8 (2651.5-4049.3) | -0.04 (-0.07--0.02) |
| HIV/AIDS and sexually transmitted infections | Israel | 158595.3 (130552-194466.8) | 3346.4 (2764.3-4121.5) | 308889.9 (255422.6-381031.9) | 3321 (2736.3-4072.7) | -0.05 (-0.08--0.02) |
| HIV/AIDS and sexually transmitted infections | Italy | 2106488.3 (1718206.4-2644916.5) | 3398.5 (2740.2-4256.1) | 2117696.7 (1729721.7-2673121.7) | 3389.3 (2722.3-4249.1) | 0 (-0.01-0.02) |
| HIV/AIDS and sexually transmitted infections | Jamaica | 268237.8 (226381.4-319772.4) | 12209.8 (10208-14820.7) | 373710.1 (311875.2-448640.8) | 11963.4 (9966.5-14399.4) | -0.03 (-0.05--0.02) |
| HIV/AIDS and sexually transmitted infections | Japan | 6883958.9 (5697401.2-8547671.9) | 4960.7 (4102-6111) | 6182909.2 (5122352.1-7641310.9) | 4989.6 (4108.3-6118.8) | -0.01 (-0.02-0) |
| HIV/AIDS and sexually transmitted infections | Jordan | 173140.7 (146666.3-205825.5) | 5377.6 (4591.7-6434.5) | 706228.7 (605406.8-841949.4) | 5353.1 (4583.4-6370.3) | 0.02 (-0.02-0.06) |
| HIV/AIDS and sexually transmitted infections | Kazakhstan | 1979840.4 (1624751.2-2398226.7) | 11804.3 (9791.1-14365.7) | 2267741 (1872897.2-2771516) | 11681.5 (9712.9-14232.6) | -0.08 (-0.1--0.06) |
| HIV/AIDS and sexually transmitted infections | Kenya | 2777980.8 (2349327.2-3298435.2) | 15946.7 (13459.9-19157.6) | 6991282.8 (5761025.9-8496295.5) | 15031.6 (12402.4-18421.9) | -0.37 (-0.44--0.29) |
| HIV/AIDS and sexually transmitted infections | Kiribati | 8240.9 (6805.4-9885.7) | 11920.3 (9892.9-14371) | 14075.2 (11583.8-16963.7) | 11682.9 (9643.9-14178.3) | -0.04 (-0.08--0.01) |
| HIV/AIDS and sexually transmitted infections | Kuwait | 159806.1 (131141.5-194629.1) | 8065 (6737.4-9782.4) | 464779.6 (382947.4-573450.8) | 7392.6 (6201.2-8865.1) | -0.3 (-0.34--0.25) |
| HIV/AIDS and sexually transmitted infections | Kyrgyzstan | 491540.8 (401672.6-591759.2) | 11752.9 (9787.6-14220.7) | 815481.5 (676311.1-986863.3) | 11703.4 (9741.5-14189.4) | -0.06 (-0.08--0.05) |
| HIV/AIDS and sexually transmitted infections | Lao People's Democratic Republic | 386583.3 (319093.1-468311.4) | 11112 (9231.6-13606.1) | 852350.6 (701362.6-1050711.6) | 10870.2 (9028.8-13288.7) | -0.04 (-0.05--0.02) |
| HIV/AIDS and sexually transmitted infections | Latvia | 246803 (206778.5-299711.7) | 9116.7 (7641-11001.6) | 154400.4 (130565.4-189224.7) | 8837.9 (7402.9-10723.4) | -0.15 (-0.17--0.13) |
| HIV/AIDS and sexually transmitted infections | Lebanon | 184084.1 (157329.7-218793.2) | 6575.1 (5604.8-7888.9) | 405993.9 (334215.5-493572) | 6522.1 (5481.3-7913.7) | -0.03 (-0.19-0.12) |
| HIV/AIDS and sexually transmitted infections | Lesotho | 219766.1 (187875.7-255917.6) | 17265.1 (14674.4-20211.3) | 325675 (281378.9-383126.3) | 16904.6 (14596.1-19952.4) | -0.27 (-0.36--0.19) |
| HIV/AIDS and sexually transmitted infections | Liberia | 244204.7 (205288.6-289987.4) | 12120.6 (10223.2-14499.5) | 610093.4 (511482.3-737270.6) | 11929.8 (10023.6-14398.9) | -0.09 (-0.12--0.06) |
| HIV/AIDS and sexually transmitted infections | Libya | 253901.9 (210926-301234.6) | 6962.7 (5824.2-8350) | 551478.4 (457312.2-666912.4) | 6702.5 (5605.2-8032.6) | -0.07 (-0.18-0.03) |
| HIV/AIDS and sexually transmitted infections | Lithuania | 343597.9 (288826.2-415450) | 9021.8 (7565-10872.2) | 227789.2 (192278.5-279489.9) | 8836.5 (7445.6-10767.8) | -0.07 (-0.08--0.06) |
| HIV/AIDS and sexually transmitted infections | Luxembourg | 13857.4 (11301-17206) | 3175.1 (2606.9-3943.5) | 23449.3 (18931.5-29941.8) | 3179.2 (2568.6-3941.4) | 0 (-0.02-0.02) |
| HIV/AIDS and sexually transmitted infections | Madagascar | 1559840.6 (1328694.3-1852397.7) | 16201.9 (13739.3-19406.5) | 4107170.8 (3446803.9-4901795.5) | 16019.8 (13488-19557.7) | -0.05 (-0.07--0.03) |
| HIV/AIDS and sexually transmitted infections | Malawi | 1341757.3 (1163712.9-1572433.8) | 16496.9 (14205.9-19647.7) | 2636851.7 (2235321.1-3099689) | 15269.1 (13010-18268.4) | -0.28 (-0.33--0.24) |
| HIV/AIDS and sexually transmitted infections | Malaysia | 1856216.6 (1541142.9-2265214.5) | 10798 (8970.1-13090.4) | 3852302.4 (3190753-4690179.1) | 10689.3 (8912.7-12937.1) | 0.01 (-0.01-0.02) |
| HIV/AIDS and sexually transmitted infections | Maldives | 19105 (15857.9-22986.1) | 10983.8 (9082.6-13393.6) | 82848.9 (66803.4-103117) | 11441.3 (9471.9-13979.5) | 0.17 (0.1-0.24) |
| HIV/AIDS and sexually transmitted infections | Mali | 944067.9 (791484.9-1116414.7) | 13910.5 (11636.7-16744.5) | 2534536.3 (2107170.5-3022598.5) | 13542.8 (11306.8-16522.1) | -0.36 (-0.46--0.25) |
| HIV/AIDS and sexually transmitted infections | Malta | 12985.1 (10537.9-16338) | 3215.2 (2629.8-3985.7) | 15326.4 (12387.1-19336.5) | 3287.7 (2660.4-4110.7) | 0.07 (0.06-0.08) |
| HIV/AIDS and sexually transmitted infections | Marshall Islands | 3556.7 (2913.9-4326.8) | 9782.6 (8042.6-11974.8) | 5662.4 (4652-6997.2) | 9606.9 (7919.3-11915.3) | -0.07 (-0.15-0.02) |
| HIV/AIDS and sexually transmitted infections | Mauritania | 201966.1 (168290.9-241770.1) | 12020.8 (9968.7-14472.3) | 444810.4 (370421-531230.7) | 11814.7 (9767.5-14315.5) | -0.1 (-0.13--0.07) |
| HIV/AIDS and sexually transmitted infections | Mauritius | 128604.7 (106579.4-156630.9) | 10793.5 (9015-13058.4) | 144631.3 (121242.1-177434.5) | 10523.6 (8764.7-12922.1) | -0.03 (-0.05--0.02) |
| HIV/AIDS and sexually transmitted infections | Mexico | 9512985.6 (7834364.9-11546257.1) | 12637.6 (10474.5-15558.5) | 17199194.9 (14191134.5-21318359.3) | 12397.7 (10222.8-15328) | 0.05 (0.01-0.1) |
| HIV/AIDS and sexually transmitted infections | Micronesia (Federated States of) | 10418.5 (8694.3-12502.8) | 11954.6 (9996-14433.1) | 12187 (10164.9-14674.4) | 11667.9 (9679.7-14142) | -0.04 (-0.06--0.02) |
| HIV/AIDS and sexually transmitted infections | Monaco | 1064.4 (873.9-1362.4) | 3237 (2644.7-4039.1) | 1191 (988.5-1514.2) | 3229.7 (2636.7-4031.3) | -0.03 (-0.04--0.02) |
| HIV/AIDS and sexually transmitted infections | Mongolia | 234479.9 (190729-283294) | 11887.6 (9922.2-14513.7) | 407685.5 (339618.8-497958.8) | 11804.9 (9899.5-14266.6) | -0.05 (-0.13-0.03) |
| HIV/AIDS and sexually transmitted infections | Montenegro | 57277.9 (47460.6-68331.9) | 8726.3 (7239.6-10422.9) | 53837.6 (45436-65026.7) | 8649.8 (7167.1-10371.3) | -0.04 (-0.05--0.03) |
| HIV/AIDS and sexually transmitted infections | Morocco | 2560023 (2139018.2-3080717.1) | 10804.9 (9124.8-13111.2) | 3802429.9 (3143457.9-4677425) | 9662.3 (7990.4-11869.2) | -0.38 (-0.42--0.34) |
| HIV/AIDS and sexually transmitted infections | Mozambique | 2110935.2 (1807712.8-2464325.7) | 19475.8 (16674.9-22928.7) | 4694229.1 (3959963.1-5496849.4) | 18289.6 (15511-21868.9) | -0.32 (-0.37--0.28) |
| HIV/AIDS and sexually transmitted infections | Myanmar | 4320996.6 (3602220.1-5186643.9) | 11179 (9331.8-13531.1) | 6358683.1 (5301601.8-7743363.1) | 10853.5 (9052.5-13249.8) | -0.06 (-0.07--0.05) |
| HIV/AIDS and sexually transmitted infections | Namibia | 203913.2 (175407.4-239254.6) | 16486.2 (14221.5-19353.6) | 404745 (345158.5-479723) | 16192.4 (13767.9-19241) | -0.25 (-0.32--0.18) |
| HIV/AIDS and sexually transmitted infections | Nauru | 1147.3 (950.4-1399.8) | 12205.4 (10125.6-14890.4) | 1300.5 (1072.6-1563.9) | 11972.7 (9930-14540.1) | -0.02 (-0.04--0.01) |
| HIV/AIDS and sexually transmitted infections | Nepal | 952684.6 (809437-1124107.1) | 5722.3 (4838.5-6817.8) | 1763369.2 (1497155.3-2113729.8) | 5521.5 (4675.6-6644.4) | -0.14 (-0.15--0.13) |
| HIV/AIDS and sexually transmitted infections | Netherlands | 549076.4 (449807.8-675714.5) | 3264.3 (2691.6-4015.9) | 556779.3 (461536-695040.1) | 3181.2 (2619-3945.6) | 0.07 (0.01-0.14) |
| HIV/AIDS and sexually transmitted infections | New Zealand | 190598.4 (157563.9-234355.6) | 5255.1 (4352-6451.3) | 284151.3 (236314.2-345677.7) | 5268.2 (4380.7-6438.6) | 0.05 (-0.14-0.24) |
| HIV/AIDS and sexually transmitted infections | Nicaragua | 386617.7 (325057.8-461860.7) | 12397.7 (10353.9-14899.6) | 844117 (697492-1015009.1) | 12006.8 (10035.4-14498.1) | -0.08 (-0.11--0.05) |
| HIV/AIDS and sexually transmitted infections | Niger | 735250 (612162.7-875799) | 12081.2 (10123.7-14522.1) | 2152105 (1804374.9-2580056.8) | 11853.1 (9914.8-14355.3) | -0.11 (-0.14--0.08) |
| HIV/AIDS and sexually transmitted infections | Nigeria | 9408004.8 (7840730.9-11244878.1) | 12701.7 (10553.1-15421.6) | 25133214.1 (20763376.1-30334490.3) | 13197.7 (10836.5-16110.5) | 0.01 (-0.04-0.06) |
| HIV/AIDS and sexually transmitted infections | Niue | 243.2 (203-292.3) | 12060 (9901.6-14627.5) | 192.8 (161.2-234) | 11802.5 (9813-14316.9) | -0.04 (-0.07-0) |
| HIV/AIDS and sexually transmitted infections | North Macedonia | 181214.7 (151217.2-220053.9) | 8642.3 (7213.4-10492.3) | 204155.6 (170265-244413.6) | 8620 (7230-10377.1) | -0.01 (-0.02-0) |
| HIV/AIDS and sexually transmitted infections | Northern Mariana Islands | 6587.1 (5408.6-8050.3) | 11609.4 (9710.8-14038.9) | 5717.5 (4819.5-6893.9) | 11364.3 (9487.1-13770.1) | -0.07 (-0.13--0.02) |
| HIV/AIDS and sexually transmitted infections | Norway | 168098.2 (137145-208236.2) | 3702.7 (3017.3-4588.6) | 213795.7 (174155.5-268463.8) | 3709.5 (3001.6-4627.9) | 0.02 (-0.03-0.06) |
| HIV/AIDS and sexually transmitted infections | Oman | 154575 (126991-186686.6) | 8130.1 (6774.7-9865.7) | 483894.8 (392723.1-594521.6) | 7962.2 (6624.8-9617) | -0.09 (-0.11--0.06) |
| HIV/AIDS and sexually transmitted infections | Pakistan | 5452933.2 (4505418.2-6679716.4) | 6199.3 (5102.5-7630.1) | 13758912.3 (11299361.5-16868015.9) | 6061.9 (4984.5-7504.9) | -0.12 (-0.17--0.08) |
| HIV/AIDS and sexually transmitted infections | Palau | 1933.3 (1589.6-2364.1) | 11875.1 (9823.9-14547.8) | 2170 (1819.7-2714.5) | 10876.3 (9094.5-13284.7) | -0.21 (-0.26--0.17) |
| HIV/AIDS and sexually transmitted infections | Palestine | 130249.6 (108414.6-153961.3) | 8136.9 (6830-9751.5) | 401969.2 (334753.7-480612.6) | 8027.9 (6700.4-9704.3) | -0.08 (-0.17-0.01) |
| HIV/AIDS and sexually transmitted infections | Panama | 270099.6 (224631.1-322387.9) | 11726.1 (9752.9-14170) | 511689.2 (426517.9-625054.5) | 11690.6 (9739.6-14309.6) | 0.04 (0.02-0.07) |
| HIV/AIDS and sexually transmitted infections | Papua New Guinea | 609571.2 (515816.2-712750.8) | 15859.3 (13616.3-18630.4) | 1570658.5 (1336142.9-1845048.5) | 15093.9 (12972-17733.9) | -0.06 (-0.12-0) |
| HIV/AIDS and sexually transmitted infections | Paraguay | 426398.3 (356406.6-510544.3) | 11930.8 (9965.3-14454.6) | 903939.3 (749305.5-1080850.4) | 11840.5 (9838.9-14210.7) | 0 (0-0.01) |
| HIV/AIDS and sexually transmitted infections | Peru | 1713562.7 (1431392.7-2035945.2) | 8705.8 (7275.3-10530.8) | 3332809.3 (2788574.5-4037683.4) | 8569 (7176.3-10383.8) | -0.04 (-0.08-0) |
| HIV/AIDS and sexually transmitted infections | Philippines | 6503326.4 (5427238.5-8038534.3) | 11476.1 (9567.8-14251.7) | 13295477.1 (11119850.1-16389759.4) | 11350.8 (9496.3-14013.4) | -0.01 (-0.02-0) |
| HIV/AIDS and sexually transmitted infections | Poland | 3549970.9 (2959425.2-4366101.3) | 8927.3 (7435.8-10833.2) | 3478982.7 (2905317.1-4277611.6) | 8739.7 (7272.8-10686.9) | -0.07 (-0.08--0.06) |
| HIV/AIDS and sexually transmitted infections | Portugal | 361389.1 (299774.2-450821.9) | 3430.1 (2822.5-4268.9) | 376578.3 (309374.8-474976.6) | 3411.5 (2787.7-4220.7) | -0.03 (-0.05--0.02) |
| HIV/AIDS and sexually transmitted infections | Puerto Rico | 434052.9 (363585.8-527867.1) | 11882.3 (9961.3-14490.6) | 378140.5 (320537.3-460230.5) | 11716.4 (9774.2-14321.8) | -0.02 (-0.03-0) |
| HIV/AIDS and sexually transmitted infections | Qatar | 48915.4 (39682.7-60020.9) | 8507.2 (7145.5-10264.4) | 382471.2 (307135.2-481060.3) | 8387.8 (7075.2-10077.7) | -0.01 (-0.03-0.01) |
| HIV/AIDS and sexually transmitted infections | Republic of Korea | 2591507.4 (2166146-3096966) | 5183.5 (4362.3-6198) | 2899047.8 (2459411.8-3558933.8) | 5044.8 (4236.8-6148.6) | -0.07 (-0.11--0.04) |
| HIV/AIDS and sexually transmitted infections | Republic of Moldova | 416825.6 (345609.6-509580) | 9145.4 (7606.9-11081.4) | 345999.1 (289657.1-427022.2) | 8931.6 (7420-10822.7) | -0.08 (-0.09--0.07) |
| HIV/AIDS and sexually transmitted infections | Romania | 2057615.9 (1722854.6-2483797.4) | 8745.9 (7253.9-10561.2) | 1566554 (1318974.5-1892938.3) | 8649.9 (7180.6-10365.8) | -0.06 (-0.08--0.04) |
| HIV/AIDS and sexually transmitted infections | Russian Federation | 14648655.1 (12271031.5-18036359.9) | 9165.8 (7655.5-11188.6) | 13438509.4 (11345229.3-16504641.6) | 9090.6 (7576.5-11120.4) | -0.04 (-0.05--0.02) |
| HIV/AIDS and sexually transmitted infections | Rwanda | 925487.8 (778220.7-1098488.8) | 16152.5 (13486.7-19497.8) | 1960800.7 (1632010.2-2374984.8) | 15571.2 (12962.3-19161.7) | -0.17 (-0.2--0.13) |
| HIV/AIDS and sexually transmitted infections | Saint Kitts and Nevis | 4595.7 (3826.4-5540.4) | 12090.2 (10058.9-14832.2) | 8052.9 (6701.5-9872.1) | 11942.6 (9949.6-14517.9) | -0.02 (-0.03--0.01) |
| HIV/AIDS and sexually transmitted infections | Saint Lucia | 14883.6 (12478.7-17828.3) | 12068.5 (10098.7-14636.1) | 23565.9 (19777.2-28682.1) | 11895.5 (9956.1-14468.5) | -0.02 (-0.03--0.01) |
| HIV/AIDS and sexually transmitted infections | Saint Vincent and the Grenadines | 11833.9 (10000.6-14095.9) | 11990.2 (10112.2-14569.8) | 13969.3 (11608.8-16837.2) | 11826.8 (9814.7-14318.8) | -0.02 (-0.03--0.02) |
| HIV/AIDS and sexually transmitted infections | Samoa | 18920.2 (15984-22577.9) | 13281.1 (11167.6-16088.5) | 25509.6 (21702.3-30805) | 13142.1 (11044.2-15969.7) | 0.01 (-0.05-0.08) |
| HIV/AIDS and sexually transmitted infections | San Marino | 847.3 (695.1-1056.7) | 3258.5 (2664.9-4068.5) | 1073.3 (890.4-1376.4) | 3198.1 (2621.9-4008.3) | -0.07 (-0.09--0.05) |
| HIV/AIDS and sexually transmitted infections | Sao Tome and Principe | 10514.8 (8880.7-12452.8) | 11595.1 (9770.4-14085.1) | 24387.1 (20372.9-29298.3) | 11605 (9699.4-14041.5) | -0.04 (-0.07--0.01) |
| HIV/AIDS and sexually transmitted infections | Saudi Arabia | 1584581.4 (1319293.9-1910468.5) | 10806.7 (8985.3-13014) | 5103007.9 (4176058.5-6315475.5) | 10008.2 (8373.3-12170.6) | -0.23 (-0.29--0.18) |
| HIV/AIDS and sexually transmitted infections | Senegal | 616784.2 (509345.7-750415.3) | 10624.6 (8793.2-13191.8) | 1417742.7 (1165540.7-1721932.8) | 10223 (8363.5-12710.9) | -0.17 (-0.28--0.06) |
| HIV/AIDS and sexually transmitted infections | Serbia | 870753.1 (731178.1-1052633.8) | 8722.1 (7288.9-10522.8) | 783725.5 (657132.6-943781.4) | 8660 (7259.2-10375.9) | -0.03 (-0.04--0.02) |
| HIV/AIDS and sexually transmitted infections | Seychelles | 7640.9 (6332.5-9097.3) | 10741.5 (8893.5-12844.5) | 12388.7 (10385.6-15340.7) | 10766.8 (9060.3-13154.7) | 0.07 (0.05-0.09) |
| HIV/AIDS and sexually transmitted infections | Sierra Leone | 415467.3 (342914.5-500537.1) | 11753.4 (9785.2-14224.9) | 946393.1 (791587.3-1138307.2) | 11721.9 (9851.2-14190) | -0.08 (-0.11--0.04) |
| HIV/AIDS and sexually transmitted infections | Singapore | 182819.6 (150427.4-221872.1) | 4937.8 (4094.9-5954.8) | 339371.6 (278972.9-421063.1) | 4916.9 (4055-5971.3) | -0.02 (-0.03-0) |
| HIV/AIDS and sexually transmitted infections | Slovakia | 470945.4 (390024.8-569720.9) | 8637.2 (7145.7-10347.7) | 480090.4 (404421.1-585646) | 8640.9 (7278.5-10325.4) | 0 (-0.01-0) |
| HIV/AIDS and sexually transmitted infections | Slovenia | 166988.5 (140524-200288) | 7899.3 (6661.3-9480.8) | 152192.5 (128275.5-184486.1) | 7756.6 (6500.1-9347.7) | -0.06 (-0.08--0.04) |
| HIV/AIDS and sexually transmitted infections | Solomon Islands | 30348 (25366.7-36493.7) | 11162 (9294.8-13531.8) | 70435.7 (58396.2-84347) | 10899.5 (9080.5-13116.7) | -0.02 (-0.04-0.01) |
| HIV/AIDS and sexually transmitted infections | Somalia | 1157615.4 (987648.6-1375444.4) | 18123.7 (15417.3-21660.4) | 3096000.5 (2641552-3691968.4) | 17598.3 (14870.5-21150.6) | -0.13 (-0.2--0.06) |
| HIV/AIDS and sexually transmitted infections | South Africa | 8331902.1 (7090264.1-9911956.7) | 22935.7 (19833.6-27365.2) | 13604876.2 (11665272.3-16353491.7) | 21590.1 (18519.2-25725.8) | -0.47 (-0.58--0.37) |
| HIV/AIDS and sexually transmitted infections | South Sudan | 801953.7 (682766.4-945998.8) | 16741.4 (14196.3-20023.7) | 1307638.3 (1102925.4-1552214.4) | 16410.2 (13793.8-19768.7) | -0.08 (-0.11--0.06) |
| HIV/AIDS and sexually transmitted infections | Spain | 1371450.2 (1135009.8-1695820.4) | 3394.8 (2783.5-4209.9) | 1645817.3 (1355658.7-2109344.7) | 3383.8 (2773.8-4184.2) | 0.01 (-0.01-0.03) |
| HIV/AIDS and sexually transmitted infections | Sri Lanka | 2038875.7 (1695934.2-2460021.9) | 11522.3 (9595.6-13843.1) | 2553114.8 (2147400-3101964.3) | 11160.3 (9305-13570.3) | -0.05 (-0.07--0.04) |
| HIV/AIDS and sexually transmitted infections | Sudan | 1655980.3 (1390406.5-1956338.5) | 9816.2 (8218.4-11682) | 4051129 (3409340.9-4781816.7) | 9669.1 (8183.2-11494.3) | -0.27 (-0.42--0.12) |
| HIV/AIDS and sexually transmitted infections | Suriname | 44983.7 (37490.9-53652.8) | 12012.9 (9979.1-14391) | 70930.1 (59065.9-87031.2) | 11914.6 (9873.3-14569) | -0.02 (-0.02--0.01) |
| HIV/AIDS and sexually transmitted infections | Sweden | 351438.6 (285941.1-438059.2) | 3868.2 (3156.7-4783.4) | 407839.4 (334467.5-508300) | 3872.3 (3154.5-4782.4) | -0.04 (-0.1-0.03) |
| HIV/AIDS and sexually transmitted infections | Switzerland | 260543.7 (211476.2-326028) | 3334.7 (2718.9-4128.2) | 315964.1 (260651-397985) | 3278.5 (2675.2-4065.7) | -0.07 (-0.1--0.03) |
| HIV/AIDS and sexually transmitted infections | Syrian Arab Republic | 794329.2 (661715.7-949168.7) | 7836.2 (6566.3-9448.7) | 1037568.7 (879357.1-1249774) | 7523.7 (6307.9-9006.1) | -0.1 (-0.11--0.09) |
| HIV/AIDS and sexually transmitted infections | Taiwan (Province of China) | 2438477.9 (2024335.5-2987334.3) | 10784.2 (8991.8-13037.8) | 2693374.9 (2217476.2-3357278.1) | 10425.7 (8533.8-12901.2) | -0.04 (-0.06--0.02) |
| HIV/AIDS and sexually transmitted infections | Tajikistan | 573541.1 (462087.7-697974.2) | 12152.1 (10089.5-14799.8) | 1237262.3 (1026246.7-1486949.2) | 11998.5 (10002.4-14354.7) | -0.07 (-0.08--0.06) |
| HIV/AIDS and sexually transmitted infections | Thailand | 7183101.2 (6039634-8617592.1) | 11706.5 (9799-14093.1) | 7858762.8 (6574356.7-9651829.4) | 11124.5 (9227.4-13592.8) | -0.06 (-0.1--0.02) |
| HIV/AIDS and sexually transmitted infections | Timor-Leste | 81983.3 (67611.6-99114.9) | 11347 (9466-13566.5) | 136524.4 (115261.4-163348) | 10841.5 (9049.9-13213.7) | -0.12 (-0.13--0.1) |
| HIV/AIDS and sexually transmitted infections | Togo | 313944.2 (263425.1-375673) | 10852.4 (9120-13179.9) | 831472.7 (695820.7-1004010.3) | 10706.5 (8991.3-12939.3) | -0.18 (-0.26--0.1) |
| HIV/AIDS and sexually transmitted infections | Tokelau | 166.5 (138.8-199.4) | 12414.3 (10359.2-15005.5) | 154.8 (129.1-187.3) | 11741.8 (9736.4-14311.9) | -0.17 (-0.2--0.14) |
| HIV/AIDS and sexually transmitted infections | Tonga | 10247.5 (8632.6-12323.8) | 12665.6 (10622.8-15250.6) | 11840.8 (9970.2-14247.4) | 12486.9 (10504.4-15175.1) | -0.01 (-0.05-0.02) |
| HIV/AIDS and sexually transmitted infections | Trinidad and Tobago | 141954.5 (118608.1-172082.4) | 11813.8 (9896.6-14488.8) | 175922.2 (144614.3-213899.7) | 11799.3 (9719.9-14367.2) | 0 (-0.01-0) |
| HIV/AIDS and sexually transmitted infections | Tunisia | 682971.9 (581418.5-804988.5) | 8736.4 (7399.8-10472.3) | 1051541.3 (881427.5-1284199.2) | 8298.6 (6995.2-10010.2) | -0.13 (-0.2--0.07) |
| HIV/AIDS and sexually transmitted infections | Turkey | 4873882 (4130911-5732186.7) | 8606 (7347.5-10137.2) | 6993635.2 (5909521.7-8485081.1) | 7737.9 (6517-9353.5) | -0.36 (-0.46--0.27) |
| HIV/AIDS and sexually transmitted infections | Turkmenistan | 410096.9 (332934.8-495999.5) | 11917.4 (9986.8-14343.1) | 616285.2 (511849.9-746159.4) | 11408.9 (9498.7-13814.7) | -0.2 (-0.23--0.18) |
| HIV/AIDS and sexually transmitted infections | Tuvalu | 1126.1 (925.2-1379) | 12659.4 (10416.1-15472.3) | 1408.2 (1172.4-1733.4) | 11450.9 (9512.1-14142.2) | -0.3 (-0.32--0.27) |
| HIV/AIDS and sexually transmitted infections | Uganda | 2334049.5 (2027584.7-2704533.8) | 16444 (14318.8-19088.6) | 5663519.1 (4928157.6-6623434.8) | 15283 (13090.2-18072.4) | -0.31 (-0.42--0.2) |
| HIV/AIDS and sexually transmitted infections | Ukraine | 4935961.6 (4118012.6-6027253.1) | 9210 (7646-11212.2) | 4079759 (3449878.5-5036513.4) | 9057.7 (7563.7-11082.3) | -0.13 (-0.16--0.09) |
| HIV/AIDS and sexually transmitted infections | United Arab Emirates | 187707.1 (152786.6-230770.6) | 8154.3 (6845.1-9881.8) | 1186556 (955205.2-1530811.8) | 8043.4 (6716.4-9940) | -0.01 (-0.03-0) |
| HIV/AIDS and sexually transmitted infections | United Kingdom | 1947493.9 (1580597.8-2453818.2) | 3188.3 (2570.4-3998.5) | 2276561.2 (1849992.4-2904299.5) | 3185.7 (2574.5-3966.8) | -0.04 (-0.08--0.01) |
| HIV/AIDS and sexually transmitted infections | United Republic of Tanzania | 4134959 (3538547.1-4902551.4) | 20292.1 (17251.2-24417.4) | 9282470.7 (7729715.2-11231005) | 18358.1 (15392.4-22432.7) | -0.32 (-0.35--0.29) |
| HIV/AIDS and sexually transmitted infections | United States of America | 17035427.1 (13625465.6-21478036.2) | 6109.8 (4933.7-7684.6) | 20391620.5 (16594297.6-25816675.8) | 5991.5 (4806.2-7595.9) | -0.16 (-0.22--0.11) |
| HIV/AIDS and sexually transmitted infections | United States Virgin Islands | 12908.2 (10769.9-15795.9) | 11908.9 (9942.7-14470.2) | 9249.7 (7823.6-11308.5) | 11815.4 (9799.1-14425) | -0.01 (-0.02--0.01) |
| HIV/AIDS and sexually transmitted infections | Uruguay | 151896.4 (129049.7-182172.8) | 4943.7 (4188-5946.9) | 173074 (146839-209934.2) | 4938.5 (4173.7-5973.4) | -0.02 (-0.04-0) |
| HIV/AIDS and sexually transmitted infections | Uzbekistan | 2288072.4 (1874926.7-2746114.4) | 11906 (9982.1-14274) | 4233050.7 (3513837.8-5136378.9) | 11588.4 (9663.3-13927.3) | -0.12 (-0.14--0.11) |
| HIV/AIDS and sexually transmitted infections | Vanuatu | 17691.7 (14828.3-21113.2) | 13331.2 (11164.6-16040.6) | 37851.1 (31744.5-45370.8) | 12659.7 (10571.1-15281) | -0.16 (-0.18--0.13) |
| HIV/AIDS and sexually transmitted infections | Venezuela (Bolivarian Republic of) | 2122515.6 (1752375.4-2560604.8) | 11869.7 (9809-14531.7) | 3273723.1 (2727384.8-4004081.9) | 11892.4 (9837.2-14454) | 0.02 (0-0.04) |
| HIV/AIDS and sexually transmitted infections | Viet Nam | 5988430 (4981212.8-7235753.8) | 9637.1 (8020.9-11677.6) | 10721133.6 (8872532-13175407) | 9729.2 (8125.7-11875) | 0.08 (0.05-0.1) |
| HIV/AIDS and sexually transmitted infections | Yemen | 803338 (670651.2-964940.9) | 8015.3 (6661.6-9609.6) | 2421960.3 (2041981.6-2905541.3) | 7826.7 (6569.2-9433.1) | -0.06 (-0.06--0.05) |
| HIV/AIDS and sexually transmitted infections | Zambia | 1149125.1 (996512.9-1344551.9) | 18360.4 (15659.3-22006.3) | 2943957.7 (2491590.3-3509576.8) | 16960.2 (14330.6-20490.2) | -0.3 (-0.34--0.27) |
| HIV/AIDS and sexually transmitted infections | Zimbabwe | 1488453.8 (1298341.8-1708895.2) | 17467.9 (15240.6-20479.1) | 2185040.3 (1852601-2562720.8) | 15065.6 (12789.4-18052.5) | -0.71 (-0.78--0.63) |
| Chlamydial infection | African Union | 17226469.48 (12347639.2-23639407.13) | 3175.54 (2326.87-4407.91) | 38144938.96 (27093856.14-52776036.65) | 2966.54 (2164.12-4145.45) | -0.18 (-0.23--0.13) |
| Chlamydial infection | Association of Southeast Asian Nations | 19873130.97 (14176144.99-28320477.45) | 4572.12 (3350.2-6445.16) | 33192143.69 (24236923.13-47400582.22) | 4464.3 (3248.79-6352.47) | 0.01 (-0.01-0.04) |
| Chlamydial infection | Central Europe, Eastern Europe, and Central Asia | 16441996.49 (11895651.67-23517210.32) | 3777.47 (2733.6-5370.57) | 16781271.95 (12393507.59-23780895.98) | 3925.43 (2867.12-5552.97) | 0.13 (0.12-0.14) |
| Chlamydial infection | Commonwealth | 27713732.29 (19771289.95-38816174.91) | 2004.7 (1462.08-2829.81) | 56521287.73 (40647778.33-79711025.94) | 2061.34 (1497.25-2921.43) | 0.06 (-0.01-0.13) |
| Chlamydial infection | European Union | 5382621.48 (3946889.8-7559252.9) | 1220.09 (892.91-1727.53) | 4835914.57 (3601820.56-6763463.87) | 1154.1 (841.72-1627.54) | -0.1 (-0.13--0.06) |
| Chlamydial infection | Four World Regions | 153725623.39 (110807215.21-218706357.76) | 2906.86 (2126.6-4113.48) | 235303997.83 (172600030.43-334159369) | 2900.87 (2119.49-4109.61) | -0.15 (-0.27--0.04) |
| Chlamydial infection | G20 | 103400465.19 (74867373.51-147954232.9) | 2710.51 (1984.21-3874.78) | 142197639.25 (105059565.7-203586385.08) | 2727.12 (1999.16-3914.2) | -0.24 (-0.41--0.06) |
| Chlamydial infection | Gulf Cooperation Council | 954435.65 (690998.61-1315694) | 4031.23 (2981.65-5516.4) | 3053923.47 (2218196.79-4305876.38) | 3552.85 (2575.68-4871.38) | -0.36 (-0.43--0.29) |
| Chlamydial infection | Health System Grouping Levels | 153908861.53 (110939452.71-218966256.83) | 2908.06 (2127.46-4115.04) | 235457819.72 (172712153.58-334367520.19) | 2901.53 (2119.96-4110.48) | -0.15 (-0.27--0.04) |
| Chlamydial infection | High SDI | 12064188.91 (8758722.64-17060597.74) | 1260.19 (919.89-1770.02) | 15902479.33 (11804452.79-22203971.08) | 1455.48 (1064.37-2044.05) | 0.42 (0.35-0.49) |
| Chlamydial infection | High-income | 6530540.93 (4798665.53-9024563.2) | 671.26 (492.49-926.32) | 7287914.82 (5446232.68-10056287.77) | 690.22 (506.3-959.21) | -0.12 (-0.21--0.03) |
| Chlamydial infection | High-middle SDI | 37546059.42 (27070735.99-53974278.44) | 3290.34 (2398.91-4708.56) | 46451135.29 (34225520.57-66733413.8) | 3333.76 (2429.72-4770.99) | -0.25 (-0.45--0.04) |
| Chlamydial infection | Latin America and Caribbean | 15156203.52 (10806301.87-21270309.58) | 4039.31 (2968.82-5661.07) | 25574066.43 (18598492.51-35980204.99) | 3998.89 (2906.93-5613.4) | -0.04 (-0.08-0.01) |
| Chlamydial infection | League of Arab States | 7330890.89 (5319844.4-9964015.32) | 3556.78 (2634.91-4853.05) | 15450776.07 (11167669-21478472.46) | 3205.06 (2326.23-4427.25) | -0.33 (-0.41--0.25) |
| Chlamydial infection | Low SDI | 10456486.85 (7472950.2-14467239.75) | 2498.24 (1817.16-3477.32) | 25329923.38 (17941166-35083339.12) | 2486.88 (1802.49-3499.93) | -0.01 (-0.06-0.04) |
| Chlamydial infection | Low-middle SDI | 27208571.17 (19431742.05-38123491.77) | 2608.66 (1901.76-3670.9) | 51293342.58 (37020820.57-72126017.76) | 2559.4 (1867.42-3600.22) | -0.09 (-0.14--0.04) |
| Chlamydial infection | Middle SDI | 66633555.17 (48044685.15-94666857.62) | 3870.94 (2850.11-5502.67) | 96480939.13 (71127294.93-137849179.71) | 3654.96 (2669.45-5205.89) | -0.33 (-0.46--0.2) |
| Chlamydial infection | Nordic Region | 157782.3 (116907.17-218342.64) | 643.68 (471.62-887.98) | 173205.92 (129611.75-237948.77) | 651.7 (478.75-900.87) | -0.02 (-0.13-0.09) |
| Chlamydial infection | North Africa and Middle East | 10729629.85 (7807982.22-14522003.55) | 3472.05 (2574.35-4752.8) | 21350904.14 (15557811.31-29791251.01) | 3160.92 (2297.02-4380.5) | -0.22 (-0.25--0.19) |
| Chlamydial infection | OECD Countries | 15174825.73 (11085132.63-21147156.96) | 1287.64 (941.44-1788.49) | 19633495.26 (14521888.54-27391281.06) | 1474.56 (1071.99-2061.23) | 0.44 (0.4-0.49) |
| Chlamydial infection | Organization of Islamic Cooperation | 31092703.55 (22305523.87-43135362) | 3357.42 (2463.67-4703.17) | 62428175.73 (45249671-87832173.44) | 3111.22 (2266.09-4370.44) | -0.19 (-0.21--0.16) |
| Chlamydial infection | Sahel Region | 3065728.18 (2169830.72-4205135) | 2579.44 (1870.78-3585.96) | 8363464.76 (5957409.27-11536810.83) | 2526.93 (1849.95-3531.9) | 0.05 (-0.06-0.15) |
| Chlamydial infection | South Asia | 17457249.18 (12445064.89-24833160.14) | 1747.73 (1275.1-2503.96) | 34723127.49 (25044022.89-50002126.62) | 1741.29 (1265.15-2500.83) | -0.1 (-0.25-0.04) |
| Chlamydial infection | Southeast Asia, East Asia, and Oceania | 75225950.34 (54221836.97-107904242.17) | 4233.16 (3104.99-6043.55) | 100232076.62 (73866701.26-143931827.48) | 4303.34 (3139.72-6162.82) | -0.23 (-0.45--0.01) |
| Chlamydial infection | Sub-Saharan Africa | 12548446 (8953346.5-17321932.63) | 3037.49 (2207.95-4214.41) | 29740876.29 (21080174.66-41257583.15) | 2902.18 (2115-4073.39) | -0.11 (-0.17--0.05) |
| Chlamydial infection | WHO region | 152433758.28 (109833198.02-216886562.22) | 2895.32 (2118.01-4098.18) | 233987345.71 (171650900.76-332322405.47) | 2893.66 (2114.5-4099.73) | -0.15 (-0.26--0.04) |
| Chlamydial infection | World Bank Income Levels | 153908231.96 (110938988.64-218965424.8) | 2908.06 (2127.46-4115.04) | 235457223.67 (172711703.82-334366748.64) | 2901.53 (2119.96-4110.48) | -0.15 (-0.27--0.04) |
| Chlamydial infection | World Bank Regions | 153839159.95 (110890040.01-218866398.84) | 2907.93 (2127.4-4114.86) | 235379912.7 (172655891.78-334259530.71) | 2901.43 (2119.91-4110.35) | -0.15 (-0.27--0.04) |
| Genital herpes | African Union | 5196792 (4418303.95-5918179.54) | 820.35 (704.7-927.29) | 12025019.2 (10118693.47-13781047.28) | 827.48 (707.22-941.22) | -0.16 (-0.23--0.1) |
| Genital herpes | Association of Southeast Asian Nations | 2547456.86 (2148946.99-2937576.99) | 538.95 (457.28-615.47) | 3827644.88 (3205353.64-4421206.74) | 528.33 (442.2-609.33) | -0.12 (-0.14--0.1) |
| Genital herpes | Central Europe, Eastern Europe, and Central Asia | 1599586.22 (1334284.69-1865064.69) | 372.84 (311.01-432.7) | 1489483.66 (1245303.97-1741698.75) | 368.2 (307.18-428.41) | -0.04 (-0.04--0.04) |
| Genital herpes | Commonwealth | 6203352.96 (5225958.96-7149818.26) | 421.72 (359.01-482.39) | 12951123.23 (10923906.26-14912926.47) | 459.74 (390.05-528.47) | 0.22 (0.17-0.28) |
| Genital herpes | European Union | 1447368.41 (1219122.93-1682024.64) | 336.74 (283.63-392.6) | 1298230.26 (1079967.97-1525439.13) | 325.73 (273.04-380.48) | -0.07 (-0.12--0.02) |
| Genital herpes | Four World Regions | 26040502.63 (21975121.63-29949839.48) | 471.23 (399.67-538.29) | 40112438.04 (33944070.25-45955433.59) | 504.31 (426.5-579.62) | 0.1 (0.07-0.14) |
| Genital herpes | G20 | 16172753.76 (13590899.19-18736407.72) | 414.63 (350.51-479.26) | 20826553.73 (17513463.3-24075969.87) | 414.94 (348.28-478.88) | -0.06 (-0.08--0.03) |
| Genital herpes | Gulf Cooperation Council | 86860.23 (70475.1-102949.09) | 368.09 (306.85-436.01) | 282480.28 (231098.23-337218.38) | 369.28 (309.62-430.89) | -0.04 (-0.06--0.02) |
| Genital herpes | Health System Grouping Levels | 26073690.69 (22002202.2-29987786.15) | 471.47 (399.88-538.56) | 40138230.77 (33966011.49-45984357.92) | 504.43 (426.59-579.75) | 0.1 (0.07-0.14) |
| Genital herpes | High SDI | 4154661.26 (3534523.68-4773846.8) | 451.37 (385.43-517.24) | 4203138.06 (3536844.95-4884927.14) | 414.08 (348.14-480.21) | -0.27 (-0.31--0.22) |
| Genital herpes | High-income | 4294428.89 (3679995.92-4894752.72) | 457.71 (393.32-521.53) | 4172416.59 (3501923.52-4837520.54) | 426.25 (357.36-493.3) | -0.2 (-0.24--0.15) |
| Genital herpes | High-middle SDI | 4503704.78 (3771751.92-5239345.74) | 391.88 (329.37-453.24) | 5189330.89 (4355268.36-6055871.85) | 399.73 (334.24-463.82) | -0.11 (-0.21--0.01) |
| Genital herpes | Latin America and Caribbean | 3524216.49 (3006424.55-4031098.26) | 845.87 (729.13-957.79) | 5189147.58 (4394309.41-5910241.16) | 830.91 (702.56-944.94) | 0 (-0.05-0.05) |
| Genital herpes | League of Arab States | 947325.06 (798998.87-1102222.95) | 425.93 (363.35-490.88) | 2034768.44 (1706388.07-2383955.17) | 423.91 (356.84-494.58) | -0.02 (-0.05-0.01) |
| Genital herpes | Low SDI | 3390105.54 (2892537.13-3845302.53) | 700.93 (602.41-792.67) | 8192259.77 (6880580.82-9410680.68) | 702.42 (598.25-801.62) | -0.2 (-0.27--0.12) |
| Genital herpes | Low-middle SDI | 4978228.28 (4165242.34-5764445.26) | 442.61 (375.02-508.52) | 9693271.46 (8144595.95-11170549.92) | 471.61 (399.13-540.6) | 0.15 (0.1-0.2) |
| Genital herpes | Middle SDI | 9046990.82 (7663239.07-10421817.85) | 490.99 (418.56-561.34) | 12860230.58 (10892711.01-14747717.59) | 506.79 (427.53-580.54) | -0.01 (-0.05-0.03) |
| Genital herpes | Nordic Region | 90459.53 (77163.39-103616.62) | 395.02 (338.43-455.79) | 90888.34 (75877.26-106007.91) | 363.46 (302.76-424.53) | -0.23 (-0.33--0.13) |
| Genital herpes | North Africa and Middle East | 1332645.92 (1115087.58-1547227.76) | 402.07 (340.6-466.6) | 2619192.91 (2191089.52-3067842.52) | 394.32 (331.43-460.1) | -0.05 (-0.08--0.02) |
| Genital herpes | OECD Countries | 5372705.64 (4592845.1-6132808.05) | 463.62 (397.65-528.73) | 5653739.61 (4763894.71-6545458.86) | 446.64 (375.63-516.29) | -0.06 (-0.09--0.02) |
| Genital herpes | Organization of Islamic Cooperation | 4983819.2 (4196562.38-5739698.04) | 488.04 (414.45-557.11) | 10519856.82 (8872737.24-12167392.9) | 509.37 (430.61-587.48) | 0.07 (0.04-0.1) |
| Genital herpes | Sahel Region | 1054310.71 (883888.96-1211456.12) | 767.67 (651.67-872.42) | 2972788.83 (2479074.38-3436482.44) | 769.7 (652.69-880.95) | -0.14 (-0.19--0.08) |
| Genital herpes | South Asia | 2923554.18 (2433284.35-3431022.04) | 287.87 (239.96-338.3) | 6064671.15 (5033779.69-7099898.91) | 304.1 (253.19-354.62) | 0.22 (0.15-0.29) |
| Genital herpes | Southeast Asia, East Asia, and Oceania | 7809710.06 (6514388.26-9131441.6) | 421.83 (353.82-490.14) | 9636963.34 (8071829.53-11204947.46) | 437.59 (364.75-507.74) | -0.14 (-0.26--0.01) |
| Genital herpes | Sub-Saharan Africa | 4615052.31 (3909070.55-5270793.07) | 940.9 (809.81-1061.75) | 11000863.5 (9251824.06-12630500.64) | 921.02 (787.54-1044.32) | -0.28 (-0.36--0.21) |
| Genital herpes | WHO region | 25953713.58 (21902751.26-29852847.91) | 471.62 (400.04-538.72) | 40023180.96 (33866542.98-45849932.2) | 504.62 (426.77-579.96) | 0.1 (0.07-0.14) |
| Genital herpes | World Bank Income Levels | 26073539.72 (22002076.72-29987606.9) | 471.46 (399.88-538.56) | 40138105.73 (33965905.62-45984212.48) | 504.43 (426.59-579.75) | 0.1 (0.07-0.14) |
| Genital herpes | World Bank Regions | 26068171.08 (21997511.92-29981471.39) | 471.54 (399.95-538.64) | 40132192.29 (33960814.4-45977111.66) | 504.49 (426.65-579.82) | 0.1 (0.07-0.14) |
| Gonococcal infection | African Union | 10160629.3 (7683435.21-13126578.58) | 1587.55 (1248.96-2019.4) | 21170001.38 (16061576.84-27456587.46) | 1448 (1138.33-1854.74) | -0.41 (-0.48--0.33) |
| Gonococcal infection | Association of Southeast Asian Nations | 6222898.67 (4751086.16-7837153.24) | 1263.05 (984.59-1580.47) | 8857720.28 (6863260.15-11154966.24) | 1242.28 (956.12-1566.38) | -0.1 (-0.14--0.06) |
| Gonococcal infection | Central Europe, Eastern Europe, and Central Asia | 8843974.79 (6743271.79-11149262.99) | 2156.41 (1639.2-2738.63) | 7065088.9 (5536318.25-8824404.47) | 2024.26 (1539.16-2569.95) | -0.33 (-0.38--0.29) |
| Gonococcal infection | Commonwealth | 20159736.53 (14675390.75-27499620.52) | 1303.23 (972.07-1744.81) | 35910064.93 (26491490.35-48265040.67) | 1248.89 (930.35-1659.89) | -0.01 (-0.16-0.13) |
| Gonococcal infection | European Union | 2497044.99 (1959924.14-3220088.09) | 601.05 (469.43-774.02) | 1837526.49 (1462274.75-2283864.03) | 506.58 (396.19-646.7) | -0.69 (-0.84--0.54) |
| Gonococcal infection | Four World Regions | 65831550.89 (49026470.77-86819034.39) | 1145.01 (868.81-1488.6) | 86164306.97 (66490389.23-108885572) | 1096.43 (838.28-1385.67) | -0.13 (-0.18--0.08) |
| Gonococcal infection | G20 | 45881824.06 (31639669.38-64938216.32) | 1133.23 (796.34-1589.04) | 52160462.49 (37390802.51-72181183.72) | 1095.41 (767.61-1541.84) | -0.05 (-0.13-0.02) |
| Gonococcal infection | Gulf Cooperation Council | 237450.31 (167001.38-342772.27) | 962.92 (694.05-1377.02) | 628846.08 (456769.38-861998.24) | 926.04 (675.48-1318.65) | -0.07 (-0.1--0.03) |
| Gonococcal infection | Health System Grouping Levels | 65873738.61 (49062569.13-86857861.74) | 1144.9 (868.83-1488.29) | 86194419.22 (66519840.01-108913096.23) | 1096.36 (838.33-1385.45) | -0.13 (-0.18--0.08) |
| Gonococcal infection | High SDI | 4433910.57 (3499942.02-5496709.47) | 493.89 (386.24-611.1) | 4702135.62 (3806559.59-5736724.94) | 480 (377.48-593.37) | -0.17 (-0.22--0.12) |
| Gonococcal infection | High-income | 2826920.88 (2291250.31-3430564.15) | 304.64 (247.29-368.68) | 2996891.95 (2465625.44-3628959.37) | 303.16 (243.28-369.34) | -0.19 (-0.27--0.1) |
| Gonococcal infection | High-middle SDI | 14679711.71 (10971028.04-19384006.83) | 1251.94 (942.23-1638.16) | 13026914.27 (10194829.96-16502495.17) | 1131.22 (853.27-1437.31) | -0.42 (-0.47--0.36) |
| Gonococcal infection | Latin America and Caribbean | 3201499.56 (2346720.87-4201180.67) | 736.26 (554.86-946.49) | 4263264.82 (3255454.79-5464499.17) | 691.37 (526.4-887.07) | -0.24 (-0.32--0.17) |
| Gonococcal infection | League of Arab States | 2776788.74 (1930002.69-4000999.34) | 1158.94 (830.33-1630.29) | 5350393.85 (3849443.45-7599976.58) | 1113.41 (805.19-1575.42) | -0.23 (-0.28--0.18) |
| Gonococcal infection | Low SDI | 6489094.32 (4944360.16-8400310.32) | 1325.74 (1041.89-1691.86) | 15058629.48 (11421606.82-19446673.3) | 1265.73 (990-1621.98) | -0.13 (-0.2--0.06) |
| Gonococcal infection | Low-middle SDI | 14480941.17 (10847843.33-18736229.59) | 1209.38 (931.02-1540.55) | 23820730.46 (18119896.23-30485767.03) | 1133.11 (876.41-1434.11) | -0.11 (-0.21--0.01) |
| Gonococcal infection | Middle SDI | 25790080.84 (18307071.46-35605725.72) | 1303.8 (953.05-1768.78) | 29586009.39 (21943697.73-39423677.52) | 1209.02 (884.46-1633.02) | -0.23 (-0.26--0.2) |
| Gonococcal infection | Nordic Region | 32926.4 (26867.63-39782.92) | 143.07 (116.66-173.15) | 35896.19 (29553.65-43399.94) | 141.21 (114.01-170.58) | -0.09 (-0.11--0.07) |
| Gonococcal infection | North Africa and Middle East | 4589789.53 (3381069.36-6294799.92) | 1262.18 (956.45-1688.64) | 7424860.38 (5610838.57-10228584.46) | 1136.59 (857.25-1567.48) | -0.28 (-0.31--0.25) |
| Gonococcal infection | OECD Countries | 6150213.36 (4833084.39-7607816.79) | 536.63 (421.55-661.98) | 6269598 (5028696.13-7725656.74) | 510.25 (398.49-631.85) | -0.2 (-0.23--0.18) |
| Gonococcal infection | Organization of Islamic Cooperation | 13553794.8 (10316497.57-17410318.84) | 1249.94 (982.33-1600.37) | 23901568.01 (18556864.45-31048292.77) | 1141.65 (896.65-1470.65) | -0.33 (-0.35--0.3) |
| Gonococcal infection | Sahel Region | 1957155.03 (1477010.78-2587687.38) | 1403.04 (1101.89-1814.38) | 5288783.14 (3991190.84-6895075.03) | 1335.81 (1045.64-1720.82) | -0.23 (-0.26--0.19) |
| Gonococcal infection | South Asia | 13787399.41 (9523400.26-19592600.39) | 1223.62 (875.48-1696.57) | 23236791.93 (16292277.82-33035274.64) | 1128.48 (804.77-1588.61) | 0 (-0.19-0.18) |
| Gonococcal infection | Southeast Asia, East Asia, and Oceania | 24309442.3 (17071253.26-34024282.37) | 1221.91 (882.27-1669.74) | 23046241 (17441919.9-30087675.95) | 1138.93 (834.65-1527.21) | -0.3 (-0.35--0.26) |
| Gonococcal infection | Sub-Saharan Africa | 8391403.49 (6457810.69-10663269.52) | 1714.8 (1347.31-2149.66) | 18244134.42 (13946437.59-23412504.24) | 1530.86 (1210.6-1929.8) | -0.47 (-0.55--0.39) |
| Gonococcal infection | WHO region | 65630259.76 (48843646.75-86649630.77) | 1146.17 (869.05-1491.11) | 85993728.68 (66313722-108726730.59) | 1097.15 (838.26-1386.97) | -0.13 (-0.18--0.08) |
| Gonococcal infection | World Bank Income Levels | 65873495.11 (49062357.12-86857628.62) | 1144.9 (868.83-1488.3) | 86194229.76 (66519655.96-108912925.27) | 1096.36 (838.33-1385.45) | -0.13 (-0.18--0.08) |
| Gonococcal infection | World Bank Regions | 65834712.2 (49029827.35-86823385.86) | 1144.65 (868.53-1488.15) | 86158972.59 (66482925.7-108881413.47) | 1096.2 (838.07-1385.4) | -0.13 (-0.18--0.08) |
| HIV/AIDS | African Union | 1672878.4 (1490829.37-1859600.33) | 287.03 (253.76-321.59) | 997530.92 (838609.21-1172170.06) | 77.36 (64.64-91.02) | -4.72 (-5.06--4.37) |
| HIV/AIDS | Association of Southeast Asian Nations | 75200.92 (51628.99-102353.29) | 16.68 (11.56-22.79) | 102195.36 (78135.21-140887.6) | 14.32 (10.87-19.88) | -1.02 (-1.41--0.63) |
| HIV/AIDS | Central Europe, Eastern Europe, and Central Asia | 15288.26 (12079.12-19211.92) | 3.52 (2.76-4.49) | 170005.84 (136592.66-211674.98) | 39.86 (32.05-51.6) | 8.52 (7.76-9.28) |
| HIV/AIDS | Commonwealth | 1069438.4 (969249.21-1186737.29) | 71.92 (64.85-79.93) | 895519.37 (741739.59-1064240.19) | 32.83 (27.18-39) | -3.57 (-4.16--2.97) |
| HIV/AIDS | European Union | 46771.45 (41918.86-53273.92) | 10.6 (9.54-12.05) | 25812.32 (22757.84-29049.82) | 6.02 (5.27-6.85) | -1.11 (-1.37--0.84) |
| HIV/AIDS | Four World Regions | 2000700.82 (1835236.2-2172421.93) | 36.45 (33.43-39.65) | 1643425.35 (1482890.16-1820661.06) | 20.77 (18.77-23.02) | -2.56 (-2.93--2.18) |
| HIV/AIDS | G20 | 251720.49 (220087.45-283750.91) | 6.53 (5.72-7.36) | 622760.41 (548427.07-700499.08) | 12.4 (10.96-13.99) | -0.13 (-1.36-1.12) |
| HIV/AIDS | Gulf Cooperation Council | 278.33 (169.02-549.01) | 1.35 (0.84-2.61) | 1759.15 (906.5-3358.61) | 2.48 (1.24-4.69) | 0.97 (0.39-1.55) |
| HIV/AIDS | Health System Grouping Levels | 2006635.21 (1841329.29-2178859.67) | 36.53 (33.51-39.74) | 1643916.33 (1483412.93-1821097.59) | 20.77 (18.77-23.02) | -2.56 (-2.93--2.19) |
| HIV/AIDS | High SDI | 96116.02 (70094.57-122017.43) | 10.26 (7.48-13.06) | 94568.24 (52818.33-133660.42) | 8.6 (4.86-12.16) | 0.32 (-0.01-0.65) |
| HIV/AIDS | High-income | 125243.89 (99489.6-152135.19) | 13.08 (10.34-15.96) | 110020.6 (67770.5-150703.71) | 10.42 (6.49-14.37) | 0.08 (-0.24-0.4) |
| HIV/AIDS | High-middle SDI | 55469.61 (48965.02-62851.72) | 4.87 (4.31-5.5) | 194552.61 (161398.35-236338.8) | 14.53 (11.8-18.24) | 4.28 (3.83-4.74) |
| HIV/AIDS | Latin America and Caribbean | 91118.25 (81722.9-103150.67) | 23.57 (21.18-26.58) | 121335.54 (94498.34-153574.57) | 19.28 (14.96-24.42) | 0.01 (-0.37-0.38) |
| HIV/AIDS | League of Arab States | 4362.05 (1966.26-10834.67) | 2.13 (0.95-5.23) | 28496.1 (12273.51-77658.73) | 6.07 (2.62-16.53) | 0.91 (0.04-1.79) |
| HIV/AIDS | Low SDI | 920688.02 (776621.48-1066226.24) | 198.34 (164.5-232.32) | 408301.34 (312594.55-536834.6) | 38.77 (29.57-50.91) | -4.83 (-5.08--4.59) |
| HIV/AIDS | Low-middle SDI | 715391.65 (658320.65-782205.66) | 64.33 (59.1-70.51) | 424831.77 (360098.7-512000.71) | 21.38 (18.13-25.68) | -4.38 (-4.69--4.07) |
| HIV/AIDS | Middle SDI | 218969.92 (188592.95-253507.4) | 12.3 (10.68-14.16) | 521662.38 (462244.43-593918.22) | 20.7 (18.28-23.56) | -0.9 (-2.03-0.25) |
| HIV/AIDS | Nordic Region | 2138.32 (1687.23-2804.24) | 8.67 (6.78-11.47) | 1155.56 (738.81-1554.49) | 4.49 (2.8-6.11) | -0.78 (-1.41--0.16) |
| HIV/AIDS | North Africa and Middle East | 3497.36 (1497.11-9028.17) | 1.13 (0.49-2.97) | 26785.56 (11435.28-70370.62) | 4.12 (1.76-10.83) | 3.08 (2.64-3.52) |
| HIV/AIDS | OECD Countries | 137015.85 (110248.83-163887.35) | 11.69 (9.38-14.01) | 131471.55 (87131.47-175401.54) | 9.77 (6.5-13.1) | 0.37 (0.02-0.72) |
| HIV/AIDS | Organization of Islamic Cooperation | 437432.8 (380382.91-498128.21) | 43.17 (37.36-49.68) | 491698.22 (390076.32-647240.06) | 24.32 (19.3-32.16) | -2.31 (-2.58--2.03) |
| HIV/AIDS | Sahel Region | 161622.48 (134067.43-194127.27) | 129.02 (106.57-156.07) | 151596.42 (117200.08-211967.87) | 45.88 (35.2-64.68) | -4.08 (-4.39--3.77) |
| HIV/AIDS | South Asia | 20065.4 (11674.51-31959.36) | 2 (1.16-3.21) | 98389.2 (59785.54-171964.7) | 5.09 (3.1-8.81) | -2.09 (-4.09--0.04) |
| HIV/AIDS | Southeast Asia, East Asia, and Oceania | 83076.09 (57082.64-114770.78) | 4.53 (3.14-6.24) | 141042.06 (108000.79-194814.42) | 6.84 (5.06-9.73) | 0.51 (0.13-0.9) |
| HIV/AIDS | Sub-Saharan Africa | 1670626.32 (1487912.3-1855567.35) | 374.16 (330.39-419.81) | 977753.76 (820303.74-1148133.32) | 95.67 (79.48-112.36) | -4.85 (-5.2--4.5) |
| HIV/AIDS | WHO region | 2000403.71 (1834857.17-2172139.13) | 36.59 (33.56-39.81) | 1642116.56 (1481600.87-1819425.97) | 20.81 (18.8-23.07) | -2.56 (-2.93--2.19) |
| HIV/AIDS | World Bank Income Levels | 2006635.03 (1841329.17-2178859.5) | 36.53 (33.51-39.74) | 1643912.33 (1483409.37-1821095.33) | 20.77 (18.77-23.02) | -2.56 (-2.93--2.19) |
| HIV/AIDS | World Bank Regions | 2006634.06 (1841328.13-2178858.67) | 36.55 (33.53-39.76) | 1643906.42 (1483402.6-1821089.28) | 20.77 (18.77-23.02) | -2.56 (-2.93--2.19) |
| Other sexually transmitted infections | African Union | 0 (0-0) | 0 (0-0) | 0 (0-0) | 0 (0-0) | 0 (0-0) |
| Other sexually transmitted infections | Association of Southeast Asian Nations | 0 (0-0) | 0 (0-0) | 0 (0-0) | 0 (0-0) | 0 (0-0) |
| Other sexually transmitted infections | Central Europe, Eastern Europe, and Central Asia | 0 (0-0) | 0 (0-0) | 0 (0-0) | 0 (0-0) | 0 (0-0) |
| Other sexually transmitted infections | Commonwealth | 0 (0-0) | 0 (0-0) | 0 (0-0) | 0 (0-0) | 0 (0-0) |
| Other sexually transmitted infections | European Union | 0 (0-0) | 0 (0-0) | 0 (0-0) | 0 (0-0) | 0 (0-0) |
| Other sexually transmitted infections | Four World Regions | 0 (0-0) | 0 (0-0) | 0 (0-0) | 0 (0-0) | 0 (0-0) |
| Other sexually transmitted infections | G20 | 0 (0-0) | 0 (0-0) | 0 (0-0) | 0 (0-0) | 0 (0-0) |
| Other sexually transmitted infections | Gulf Cooperation Council | 0 (0-0) | 0 (0-0) | 0 (0-0) | 0 (0-0) | 0 (0-0) |
| Other sexually transmitted infections | Health System Grouping Levels | 0 (0-0) | 0 (0-0) | 0 (0-0) | 0 (0-0) | 0 (0-0) |
| Other sexually transmitted infections | High SDI | 0 (0-0) | 0 (0-0) | 0 (0-0) | 0 (0-0) | 0 (0-0) |
| Other sexually transmitted infections | High-income | 0 (0-0) | 0 (0-0) | 0 (0-0) | 0 (0-0) | 0 (0-0) |
| Other sexually transmitted infections | High-middle SDI | 0 (0-0) | 0 (0-0) | 0 (0-0) | 0 (0-0) | 0 (0-0) |
| Other sexually transmitted infections | Latin America and Caribbean | 0 (0-0) | 0 (0-0) | 0 (0-0) | 0 (0-0) | 0 (0-0) |
| Other sexually transmitted infections | League of Arab States | 0 (0-0) | 0 (0-0) | 0 (0-0) | 0 (0-0) | 0 (0-0) |
| Other sexually transmitted infections | Low SDI | 0 (0-0) | 0 (0-0) | 0 (0-0) | 0 (0-0) | 0 (0-0) |
| Other sexually transmitted infections | Low-middle SDI | 0 (0-0) | 0 (0-0) | 0 (0-0) | 0 (0-0) | 0 (0-0) |
| Other sexually transmitted infections | Middle SDI | 0 (0-0) | 0 (0-0) | 0 (0-0) | 0 (0-0) | 0 (0-0) |
| Other sexually transmitted infections | Nordic Region | 0 (0-0) | 0 (0-0) | 0 (0-0) | 0 (0-0) | 0 (0-0) |
| Other sexually transmitted infections | North Africa and Middle East | 0 (0-0) | 0 (0-0) | 0 (0-0) | 0 (0-0) | 0 (0-0) |
| Other sexually transmitted infections | OECD Countries | 0 (0-0) | 0 (0-0) | 0 (0-0) | 0 (0-0) | 0 (0-0) |
| Other sexually transmitted infections | Organization of Islamic Cooperation | 0 (0-0) | 0 (0-0) | 0 (0-0) | 0 (0-0) | 0 (0-0) |
| Other sexually transmitted infections | Sahel Region | 0 (0-0) | 0 (0-0) | 0 (0-0) | 0 (0-0) | 0 (0-0) |
| Other sexually transmitted infections | South Asia | 0 (0-0) | 0 (0-0) | 0 (0-0) | 0 (0-0) | 0 (0-0) |
| Other sexually transmitted infections | Southeast Asia, East Asia, and Oceania | 0 (0-0) | 0 (0-0) | 0 (0-0) | 0 (0-0) | 0 (0-0) |
| Other sexually transmitted infections | Sub-Saharan Africa | 0 (0-0) | 0 (0-0) | 0 (0-0) | 0 (0-0) | 0 (0-0) |
| Other sexually transmitted infections | WHO region | 0 (0-0) | 0 (0-0) | 0 (0-0) | 0 (0-0) | 0 (0-0) |
| Other sexually transmitted infections | World Bank Income Levels | 0 (0-0) | 0 (0-0) | 0 (0-0) | 0 (0-0) | 0 (0-0) |
| Other sexually transmitted infections | World Bank Regions | 0 (0-0) | 0 (0-0) | 0 (0-0) | 0 (0-0) | 0 (0-0) |
| Sexually transmitted infections excluding HIV | African Union | 71512348.1 (61041681.62-84340692.48) | 13579.41 (11556.37-16308.76) | 164900834.31 (138798905.92-196453781) | 13159.08 (11100.11-15911.8) | -0.19 (-0.23--0.15) |
| Sexually transmitted infections excluding HIV | Association of Southeast Asian Nations | 46825538.17 (39175918.75-56865134.31) | 10935.16 (9202.04-13392.5) | 79253650.06 (66304074.63-97831720.43) | 10706.12 (8993.05-13173.69) | -0.02 (-0.04--0.01) |
| Sexually transmitted infections excluding HIV | Central Europe, Eastern Europe, and Central Asia | 41020985.26 (34684776.6-49876351.74) | 9503.59 (8060.25-11514.77) | 40731083.21 (34200720.06-49687610.97) | 9599.61 (8123.71-11656.75) | 0.01 (-0.01-0.02) |
| Sexually transmitted infections excluding HIV | Commonwealth | 106604404.36 (91338256.29-126696388.17) | 7781.7 (6624.22-9284.21) | 216396144.56 (182904483.45-259818377.03) | 7965 (6725.86-9518.88) | 0.01 (0-0.03) |
| Sexually transmitted infections excluding HIV | European Union | 21683014.5 (18220071.3-26351061.83) | 4835.42 (4068.19-5889.56) | 20925865.2 (17607745.13-25408465.08) | 4658.95 (3894.91-5687.1) | -0.11 (-0.13--0.09) |
| Sexually transmitted infections excluding HIV | Four World Regions | 454978589.48 (384677957.77-542940937.1) | 8688.79 (7313.29-10471.56) | 721667028.89 (607346436.09-871730684.18) | 8869.63 (7456.33-10687.6) | -0.02 (-0.07-0.03) |
| Sexually transmitted infections excluding HIV | G20 | 303263248.92 (255424883.54-366844563.23) | 8015.26 (6724.67-9705.05) | 419593288.9 (352665822.45-512050137.7) | 7981.65 (6689.47-9726.12) | -0.13 (-0.19--0.06) |
| Sexually transmitted infections excluding HIV | Gulf Cooperation Council | 2183229.69 (1816381.68-2628536.44) | 9931.45 (8270.56-11954.87) | 7779359.02 (6393403.9-9677699.15) | 9223.49 (7682.7-11218.37) | -0.25 (-0.29--0.21) |
| Sexually transmitted infections excluding HIV | Health System Grouping Levels | 455457172.12 (385082156.72-543499010.04) | 8691.17 (7315.27-10474.48) | 722090623.63 (607703083.26-872234424.69) | 8870.88 (7457.37-10689.14) | -0.02 (-0.07-0.03) |
| Sexually transmitted infections excluding HIV | High SDI | 54120053.34 (44900400.58-66359849.45) | 5632.89 (4678.13-6871.56) | 67157473.2 (55606011.33-82371964.67) | 5848.46 (4855.31-7167.76) | 0.1 (0.09-0.12) |
| Sexually transmitted infections excluding HIV | High-income | 45777080.04 (37381437.89-56564315.51) | 4651.07 (3802.83-5743.71) | 52117408.06 (43046733.34-65597993.81) | 4651.6 (3796.87-5770.58) | -0.04 (-0.07--0.02) |
| Sexually transmitted infections excluding HIV | High-middle SDI | 95828334.15 (80747019.91-117059744.75) | 8459.73 (7125.7-10336.75) | 118773393.44 (98874561.81-147194243.19) | 8419.66 (7068.73-10380.5) | -0.18 (-0.27--0.08) |
| Sexually transmitted infections excluding HIV | Latin America and Caribbean | 42290588.67 (35268293.88-50777061.47) | 11587.85 (9669.67-14144.24) | 73289767.31 (61037491.77-89822724.15) | 11455.14 (9550.28-14007.49) | -0.03 (-0.05--0.01) |
| Sexually transmitted infections excluding HIV | League of Arab States | 18532996.6 (15657003.23-21878721) | 9343.8 (7869.5-11162.74) | 41792690.8 (35002488.46-50534445.44) | 8861.48 (7445.65-10686.04) | -0.23 (-0.25--0.2) |
| Sexually transmitted infections excluding HIV | Low SDI | 46774613.08 (39825930.29-55154157.77) | 11438.99 (9711.77-13741.79) | 112464362.48 (94586703.82-133837679.94) | 11351.56 (9576.68-13740.47) | -0.07 (-0.1--0.05) |
| Sexually transmitted infections excluding HIV | Low-middle SDI | 85461111.3 (73104348.57-101690503.72) | 8282.07 (6998.78-9899.83) | 165243063.9 (139429850.78-197287480.53) | 8341.38 (7023.68-10037.92) | -0.02 (-0.04--0.01) |
| Sexually transmitted infections excluding HIV | Middle SDI | 173273060.25 (145911380.74-211455680.73) | 10179.18 (8545.1-12380.76) | 258452330.61 (216855460.91-317811895.3) | 9774.15 (8213.03-11946.26) | -0.22 (-0.28--0.16) |
| Sexually transmitted infections excluding HIV | Nordic Region | 913932.69 (745004.26-1133388.99) | 3621.74 (2957.14-4445.79) | 1024016.28 (843032.88-1278819.62) | 3626.55 (2948.51-4480.94) | -0.01 (-0.05-0.03) |
| Sexually transmitted infections excluding HIV | North Africa and Middle East | 26663129.09 (22522209.77-31472980.32) | 8873.42 (7509.19-10587.36) | 55794562.97 (46845899.18-67444108.85) | 8394.01 (7088.72-10131.38) | -0.2 (-0.22--0.18) |
| Sexually transmitted infections excluding HIV | OECD Countries | 68761909.11 (57267763.89-83804006.7) | 5816.54 (4843.13-7093.19) | 86073644.47 (71751733.67-105864523.26) | 6140.99 (5081.94-7554.1) | 0.17 (0.16-0.18) |
| Sexually transmitted infections excluding HIV | Organization of Islamic Cooperation | 89239782.37 (75424307.61-105145558.97) | 9829.59 (8321.27-11752.93) | 189209521.16 (159079641.85-225525171.95) | 9566.99 (8086.95-11460.04) | -0.13 (-0.14--0.11) |
| Sexually transmitted infections excluding HIV | Sahel Region | 14731649.94 (12471261.9-17432247.13) | 12877.97 (10841.95-15512.9) | 40564291.74 (33963418.09-48372726.18) | 12782.45 (10680.59-15450.76) | -0.11 (-0.15--0.07) |
| Sexually transmitted infections excluding HIV | South Asia | 61711326.07 (52657363.94-73812932.83) | 6207.74 (5319.34-7392.63) | 119667712.03 (101486816.95-143536952.41) | 6066.1 (5158.24-7262.89) | -0.1 (-0.12--0.09) |
| Sexually transmitted infections excluding HIV | Southeast Asia, East Asia, and Oceania | 178357485.26 (149354718.63-219395426.9) | 10160.19 (8459.61-12555.13) | 237867192.96 (197283043.25-295011032.71) | 10090.09 (8438.67-12499.56) | -0.17 (-0.28--0.07) |
| Sexually transmitted infections excluding HIV | Sub-Saharan Africa | 60130795.37 (51139157.11-70699555.6) | 14990.88 (12735.98-17996.46) | 143284915.79 (120630548.6-170452882.79) | 14422.79 (12151.32-17379.42) | -0.22 (-0.25--0.18) |
| Sexually transmitted infections excluding HIV | WHO region | 452536823.99 (382558696.72-540047055.69) | 8679.79 (7304.51-10460.2) | 718971596.04 (605093370.62-868570298.39) | 8864.76 (7452.49-10681.27) | -0.02 (-0.07-0.03) |
| Sexually transmitted infections excluding HIV | World Bank Income Levels | 455454608.46 (385079990.82-543496147.88) | 8691.15 (7315.26-10474.47) | 722088209.1 (607701041.26-872231413.5) | 8870.87 (7457.36-10689.13) | -0.02 (-0.07-0.03) |
| Sexually transmitted infections excluding HIV | World Bank Regions | 455273394.72 (384921346.76-543288601.07) | 8691.24 (7315.33-10474.56) | 721884059.41 (607530535.71-871986504.57) | 8871.01 (7457.48-10689.32) | -0.02 (-0.07-0.03) |
| Syphilis | African Union | 3867074.4 (2974128.62-4905634.7) | 649.9 (503.04-819.77) | 7352583.67 (5532204.84-9536876.82) | 537.79 (408.59-692.57) | -0.72 (-0.8--0.63) |
| Syphilis | Association of Southeast Asian Nations | 1028134.92 (751405.33-1363509.89) | 221.6 (164.91-290.49) | 1565803.76 (1172992.55-2052959.83) | 216.45 (162.25-282.59) | 0.11 (0.05-0.18) |
| Syphilis | Central Europe, Eastern Europe, and Central Asia | 259169.55 (198832.15-336649.26) | 60.34 (45.96-78.27) | 221225.34 (169278.03-288935.57) | 54.57 (40.9-71.33) | -0.45 (-0.5--0.4) |
| Syphilis | Commonwealth | 5017358.21 (3746709.07-6486275.27) | 337.85 (253.83-433.18) | 8393448.85 (6217561.8-11003513.13) | 300.46 (225.13-390.7) | -0.74 (-0.89--0.59) |
| Syphilis | European Union | 295678.38 (223513.85-384098.45) | 67.74 (51.06-88.6) | 266695.21 (199970.18-345550.68) | 64.77 (47.91-84.79) | -0.12 (-0.15--0.1) |
| Syphilis | Four World Regions | 11959855.5 (8920920.5-15536190.24) | 217.02 (163.33-280.37) | 18678962.21 (14020597.01-24309498.63) | 235.54 (176.44-307.52) | 0.03 (-0.06-0.12) |
| Syphilis | G20 | 6751236.09 (4916502.35-8893211.77) | 172.97 (128.08-227.57) | 8897460.85 (6660021.65-11584479.06) | 180.13 (133.48-235.48) | -0.16 (-0.31--0.01) |
| Syphilis | Gulf Cooperation Council | 17727.5 (12918.68-23317.2) | 75.95 (56.17-99.09) | 61514.89 (45005.43-82017.62) | 77.66 (57.53-102.02) | 0.13 (0.09-0.17) |
| Syphilis | Health System Grouping Levels | 11966811.81 (8926352.89-15545150.67) | 216.99 (163.3-280.32) | 18684597.7 (14024956.87-24316714.66) | 235.51 (176.43-307.48) | 0.03 (-0.06-0.12) |
| Syphilis | High SDI | 911988.28 (689040.8-1199284.16) | 97.62 (73.46-127.92) | 1000727.41 (760621.79-1306083.44) | 94.64 (70.56-123.45) | -0.12 (-0.22--0.02) |
| Syphilis | High-income | 942424.36 (716622.32-1229366.65) | 98.74 (74.75-128.64) | 988714.72 (748661.06-1283913.51) | 96.89 (72.03-126.49) | -0.05 (-0.1-0.01) |
| Syphilis | High-middle SDI | 1345398.57 (987446.58-1770177.9) | 117.31 (87.27-153.1) | 1580600.78 (1199673.81-2052697.51) | 122.16 (90.59-159.87) | 0.03 (-0.11-0.17) |
| Syphilis | Latin America and Caribbean | 634979.57 (466428.05-831385.54) | 157.65 (118.05-205.17) | 1053436.1 (799908.94-1356568.88) | 168.82 (128.33-216.47) | -0.31 (-0.6--0.03) |
| Syphilis | League of Arab States | 289337.12 (216093.17-378153.32) | 131.45 (98.85-170.78) | 611188.65 (455880.79-796109.67) | 127.83 (95.69-166.17) | 0.01 (-0.05-0.06) |
| Syphilis | Low SDI | 2856057.54 (2216303.62-3619998.3) | 621.28 (479.83-783.91) | 5665495.41 (4266423.36-7356971.15) | 519.69 (395.12-667.48) | -0.78 (-0.84--0.71) |
| Syphilis | Low-middle SDI | 3277725.17 (2423416.13-4256194.95) | 290.1 (216.93-376.48) | 5556832.71 (4106335.57-7294019.56) | 271.96 (202.72-354.69) | -0.53 (-0.66--0.4) |
| Syphilis | Middle SDI | 3575642.25 (2595996.79-4739583.9) | 195.34 (144.81-257.14) | 4880941.38 (3650324.44-6407225.48) | 193.33 (143.36-253) | -0.23 (-0.3--0.15) |
| Syphilis | Nordic Region | 17362.91 (12930.91-22742.87) | 72.79 (54-95.53) | 18344.42 (13673.6-23795.33) | 70.78 (52.46-92.44) | -0.12 (-0.13--0.1) |
| Syphilis | North Africa and Middle East | 282679.72 (207820.51-371866.89) | 84.89 (62.68-111.39) | 556086.61 (410662.19-730852.7) | 84.35 (62.01-110.56) | 0.04 (-0.04-0.12) |
| Syphilis | OECD Countries | 1100417.13 (827644.91-1442524.97) | 94.48 (71.01-123.52) | 1199701.45 (902344.64-1557686.8) | 91.45 (67.92-119.47) | -0.13 (-0.21--0.06) |
| Syphilis | Organization of Islamic Cooperation | 2522528.88 (1887685.14-3270773.02) | 249.47 (187.94-322.38) | 4917932.1 (3656997.86-6428711.81) | 239.11 (178.89-312.24) | -0.12 (-0.17--0.07) |
| Syphilis | Sahel Region | 669793.59 (509840.93-856850.15) | 511.12 (389.95-652.1) | 1576490.03 (1171816.62-2038934.83) | 434.46 (328.95-562.47) | -0.42 (-0.46--0.38) |
| Syphilis | South Asia | 3049606.57 (2218898.85-4001539.36) | 284.06 (210.06-370.71) | 5059818.31 (3711516.49-6662314.26) | 251.82 (187.19-328.48) | -0.88 (-1.14--0.63) |
| Syphilis | Southeast Asia, East Asia, and Oceania | 3096094.2 (2236227.55-4127929.3) | 166.92 (123.08-221.26) | 3736078.15 (2799844.14-4885307.61) | 171.5 (126.6-225.55) | 0.07 (-0.05-0.2) |
| Syphilis | Sub-Saharan Africa | 3709799.69 (2859915.36-4683749.33) | 814.61 (630.46-1025.67) | 7080649.92 (5325060.39-9182656.74) | 644.72 (489.69-828.23) | -0.86 (-0.94--0.78) |
| Syphilis | WHO region | 11928538.87 (8897291.36-15497314.97) | 217.35 (163.55-280.79) | 18647918.53 (13996334.69-24271368.42) | 235.81 (176.64-307.9) | 0.03 (-0.06-0.12) |
| Syphilis | World Bank Income Levels | 11966754.96 (8926309.79-15545077.55) | 216.99 (163.3-280.32) | 18684549.19 (14024918.21-24316654.41) | 235.51 (176.42-307.48) | 0.03 (-0.06-0.12) |
| Syphilis | World Bank Regions | 11965795.68 (8925574.02-15543858.99) | 217.05 (163.35-280.41) | 18683506.01 (14024121.66-24315374.68) | 235.56 (176.46-307.55) | 0.03 (-0.06-0.12) |
| Trichomoniasis | African Union | 35061382.91 (26565843.4-45943276.18) | 7346.07 (5616.66-9768.97) | 86208291.1 (64998248.15-113408778.98) | 7379.27 (5611.27-9871.95) | -0.11 (-0.17--0.05) |
| Trichomoniasis | Association of Southeast Asian Nations | 17153916.75 (12663480.79-22922364.05) | 4339.44 (3267.57-5831.66) | 31810337.45 (23868004.88-43453084.02) | 4254.76 (3198.31-5758.32) | -0.03 (-0.06--0.01) |
| Trichomoniasis | Central Europe, Eastern Europe, and Central Asia | 13876258.21 (10354091.05-18709310.94) | 3136.52 (2338.06-4237.68) | 15174013.35 (11460924.93-20704218.03) | 3227.15 (2417.35-4331.69) | 0.1 (0.08-0.12) |
| Trichomoniasis | Commonwealth | 47510224.37 (35721285.11-63013104.17) | 3714.2 (2808.71-4992.45) | 102620219.81 (77121657.88-137564200.88) | 3894.57 (2935.14-5240.65) | 0.03 (-0.02-0.08) |
| Trichomoniasis | European Union | 12060301.25 (9049133.92-16106579.92) | 2609.8 (1950.45-3500.38) | 12687498.67 (9739794.34-17118094.4) | 2607.77 (1954.52-3500.98) | 0.01 (-0.01-0.03) |
| Trichomoniasis | Four World Regions | 197421057.07 (147050888.5-262982238.95) | 3948.67 (2965.4-5315.31) | 341407323.84 (257653183.84-463617931.91) | 4132.49 (3110.61-5582.5) | 0.09 (0.06-0.13) |
| Trichomoniasis | G20 | 131056969.83 (97417448.3-177162071.7) | 3583.93 (2682.51-4871.62) | 195511172.58 (147144122.41-269220391.78) | 3564.05 (2673.63-4877.1) | -0.07 (-0.11--0.03) |
| Trichomoniasis | Gulf Cooperation Council | 886756.01 (646165.39-1177417.21) | 4493.26 (3415.76-6015.5) | 3752594.3 (2747573.57-5266651.88) | 4297.67 (3270.09-5887.54) | -0.22 (-0.31--0.13) |
| Trichomoniasis | Health System Grouping Levels | 197634069.48 (147207157.99-263268937.4) | 3949.76 (2966.25-5316.78) | 341615556.22 (257816987.56-463892022.37) | 4133.06 (3111.09-5583.18) | 0.09 (0.06-0.13) |
| Trichomoniasis | High SDI | 32555304.31 (24253481.26-43953966.92) | 3329.82 (2489.07-4499.36) | 41348992.78 (31407580.1-56584324.17) | 3404.25 (2546.16-4635.24) | 0.07 (0.03-0.11) |
| Trichomoniasis | High-income | 31182764.98 (23341667.61-42052843.92) | 3118.72 (2331.91-4213.91) | 36671469.99 (27900094.09-50000563.98) | 3135.08 (2340.83-4252.6) | 0.01 (-0.03-0.05) |
| Trichomoniasis | High-middle SDI | 37753459.66 (28016340.28-50764887.38) | 3408.26 (2557.88-4605.36) | 52525412.2 (39790307.72-72815727.58) | 3432.79 (2574.18-4650.02) | -0.04 (-0.08-0) |
| Trichomoniasis | Latin America and Caribbean | 19773689.53 (14595419.81-26450122.61) | 5808.76 (4361.54-7865.74) | 37209852.39 (27864896.75-51007853.12) | 5765.16 (4328.28-7872.87) | 0.01 (-0.01-0.03) |
| Trichomoniasis | League of Arab States | 7188654.79 (5356622.03-9464952.61) | 4070.7 (3080.19-5366.79) | 18345563.79 (13699797.96-24697716) | 3991.27 (3018.22-5353.16) | -0.17 (-0.24--0.1) |
| Trichomoniasis | Low SDI | 23582868.82 (17822891.76-30984352.45) | 6292.81 (4797.72-8330.93) | 58218054.43 (43576434.06-76643071.42) | 6376.84 (4831.97-8522.19) | -0.01 (-0.05-0.03) |
| Trichomoniasis | Low-middle SDI | 35515645.52 (26495213.81-47511472.59) | 3731.32 (2809.68-5013.14) | 74878886.68 (55931765.53-100516689.79) | 3905.3 (2943.09-5265.6) | 0.06 (0.03-0.1) |
| Trichomoniasis | Middle SDI | 68226791.17 (50622557.71-90680245.55) | 4318.11 (3246.91-5852.54) | 114644210.13 (86386539.28-157598879.52) | 4210.06 (3165.35-5731.87) | -0.14 (-0.17--0.1) |
| Trichomoniasis | Nordic Region | 615401.55 (462255.46-836433.24) | 2367.18 (1769.34-3185.64) | 705681.41 (535619.48-945565.56) | 2399.41 (1811.31-3217.35) | 0.03 (0.01-0.05) |
| Trichomoniasis | North Africa and Middle East | 9728384.07 (7261174.22-12836657.7) | 3652.23 (2759.8-4848.42) | 23843518.94 (17745655.34-32208708.1) | 3617.82 (2727.47-4861.82) | -0.18 (-0.24--0.12) |
| Trichomoniasis | OECD Countries | 40963747.24 (30542044.1-55036334.1) | 3434.17 (2562.46-4627.11) | 53317110.16 (40472224.51-72707867.88) | 3618.09 (2714.12-4907.32) | 0.15 (0.12-0.18) |
| Trichomoniasis | Organization of Islamic Cooperation | 37086935.93 (27732618.25-49157796.14) | 4484.72 (3390.58-5973.14) | 87441988.5 (65484644.45-116600901.53) | 4565.64 (3453.59-6102) | -0.05 (-0.09--0.01) |
| Trichomoniasis | Sahel Region | 7984662.43 (6021921.88-10433585.37) | 7616.7 (5826.04-10027.77) | 22362764.98 (16663023.57-29438864.3) | 7715.55 (5870.79-10272.16) | -0.13 (-0.19--0.06) |
| Trichomoniasis | South Asia | 24493516.73 (18103756.35-32977582.53) | 2664.45 (2001.7-3624.9) | 50583303.16 (37624320.55-68875010.18) | 2640.4 (1970.03-3591.75) | -0.12 (-0.16--0.07) |
| Trichomoniasis | Southeast Asia, East Asia, and Oceania | 67916288.36 (50077857.67-90850922.85) | 4116.37 (3082.94-5567.32) | 101215833.86 (76501279.9-140100207.58) | 4038.73 (3024.98-5480.51) | -0.09 (-0.14--0.04) |
| Trichomoniasis | Sub-Saharan Africa | 30866093.89 (23365309.53-40383805.55) | 8483.08 (6484.64-11285.69) | 77218391.65 (58010279.19-101652827.96) | 8424.01 (6408.1-11316.02) | -0.14 (-0.21--0.08) |
| Trichomoniasis | WHO region | 196590553.52 (146412999.5-261893740.59) | 3949.34 (2965.63-5317.02) | 340319422.15 (256818486.46-462161071.02) | 4133.52 (3111.4-5584.4) | 0.09 (0.06-0.13) |
| Trichomoniasis | World Bank Income Levels | 197632586.71 (147206053.06-263266998.71) | 3949.74 (2966.24-5316.76) | 341614100.76 (257815871.24-463890099.02) | 4133.05 (3111.09-5583.17) | 0.09 (0.06-0.13) |
| Trichomoniasis | World Bank Regions | 197565555.8 (147155999.47-263177905.52) | 3950.06 (2966.49-5317.23) | 341529475.82 (257751632.04-463776720.68) | 4133.33 (3111.31-5583.6) | 0.09 (0.06-0.13) |

Table S2. The number of STD DALYs cases and age standardized DALYs of STD caused by different locaions from 1990 to 2021.

| **cause** | **location** | **1990** | | **2021** | | **1990-2021** |
| --- | --- | --- | --- | --- | --- | --- |
|  |  | DALYs cases  No. ×10^3^ (95% UI) | DALYs rate per 100,000  No. (95% UI) | DALYs cases  No. ×10^3^ (95% UI) | DALYs  rate per 100,000  No. (95% UI) | EAPC  No. (95% CI) |
| Chlamydial infection | Afghanistan | 90 (53.6-140.4) | 1.3 (0.8-2.1) | 357.6 (222.9-553.2) | 1.4 (0.9-2.1) | 0.27 (-1.62-2.2) |
| Chlamydial infection | Albania | 41.2 (25.7-65.4) | 1.2 (0.8-1.9) | 30.9 (19.6-46.3) | 1.2 (0.7-1.7) | -0.44 (-1.52-0.65) |
| Chlamydial infection | Algeria | 277.8 (168-435) | 1.3 (0.8-2.1) | 607.6 (354.6-931.8) | 1.3 (0.8-2) | -0.05 (-1.71-1.63) |
| Chlamydial infection | American Samoa | 0.3 (0.2-0.5) | 0.8 (0.5-1.2) | 0.4 (0.2-0.6) | 0.8 (0.5-1.2) | 0.22 (-2.25-2.76) |
| Chlamydial infection | Andorra | 0.3 (0.2-0.4) | 0.4 (0.3-0.7) | 0.4 (0.3-0.6) | 0.4 (0.2-0.5) | -0.81 (-2.23-0.63) |
| Chlamydial infection | Angola | 338.3 (211.4-477.3) | 4.2 (2.6-6) | 763.2 (526.2-1116.5) | 3 (2.1-4.4) | 0.25 (-2.86-3.45) |
| Chlamydial infection | Antigua and Barbuda | 1.2 (0.8-1.8) | 2 (1.3-2.9) | 2.2 (1.5-3.1) | 2.2 (1.5-3.2) | -0.3 (-2.48-1.93) |
| Chlamydial infection | Argentina | 503.2 (352.5-707.2) | 1.6 (1.1-2.2) | 754.3 (535.7-1090.3) | 1.5 (1.1-2.2) | -0.36 (-2.29-1.61) |
| Chlamydial infection | Armenia | 93.8 (67.8-126.8) | 2.7 (2-3.7) | 67.1 (45.4-100.9) | 2 (1.4-3.1) | -1.22 (-2.26--0.18) |
| Chlamydial infection | Australia | 353.7 (226.6-545) | 1.9 (1.2-2.9) | 459.1 (277.7-715.9) | 1.8 (1.1-2.7) | -1.33 (-2.67-0.02) |
| Chlamydial infection | Austria | 34.7 (24.6-49.3) | 0.4 (0.3-0.6) | 31.4 (21.4-45.1) | 0.3 (0.2-0.5) | -0.88 (-2.29-0.55) |
| Chlamydial infection | Azerbaijan | 239.2 (178.1-316.9) | 3.5 (2.6-4.6) | 323.2 (221.5-468.8) | 2.7 (1.9-3.9) | -1.22 (-2.53-0.1) |
| Chlamydial infection | Bahrain | 8.7 (5.1-14.3) | 1.4 (0.9-2.2) | 28.2 (16.8-45.7) | 1.4 (0.9-2.2) | -0.19 (-2.07-1.73) |
| Chlamydial infection | Bangladesh | 2396.9 (1712.2-3371.9) | 2.9 (2.1-4) | 3107.9 (2150.8-4461.6) | 1.9 (1.3-2.7) | 0.15 (-2.13-2.49) |
| Chlamydial infection | Barbados | 7.9 (5.4-11.3) | 2.9 (2-4.2) | 8.8 (6.1-12.8) | 2.9 (2-4.3) | -0.79 (-3-1.47) |
| Chlamydial infection | Belarus | 174.7 (124.4-239.2) | 1.6 (1.1-2.2) | 154.6 (110.6-216.5) | 1.5 (1.1-2.1) | -0.45 (-2.15-1.29) |
| Chlamydial infection | Belgium | 51.5 (38.5-70.7) | 0.5 (0.3-0.6) | 46.4 (33.1-67) | 0.4 (0.3-0.6) | -0.7 (-2.22-0.85) |
| Chlamydial infection | Belize | 3 (1.9-4.6) | 2 (1.2-2.9) | 9.7 (6.3-14.3) | 2.1 (1.4-3.1) | -0.05 (-2.66-2.64) |
| Chlamydial infection | Benin | 122.9 (83.3-181.1) | 3.2 (2.2-4.8) | 306.7 (201-457.5) | 2.6 (1.7-3.9) | -0.41 (-3.25-2.52) |
| Chlamydial infection | Bermuda | 1.4 (0.9-2.1) | 1.9 (1.2-2.8) | 1.2 (0.7-1.7) | 1.9 (1.2-2.9) | -0.54 (-2.84-1.81) |
| Chlamydial infection | Bhutan | 15.5 (9.2-23.2) | 3.1 (1.9-4.6) | 19.8 (13.2-29.4) | 2.4 (1.6-3.5) | -0.07 (-2.86-2.8) |
| Chlamydial infection | Bolivia | 135.4 (94.2-191.2) | 2.5 (1.7-3.5) | 262.8 (172.3-382.3) | 2.1 (1.4-3.1) | 0.06 (-2.78-2.98) |
| Chlamydial infection | Bosnia and Herzegovina | 55.8 (35-86.6) | 1.1 (0.7-1.8) | 36.8 (23-56.8) | 1.1 (0.7-1.8) | -0.03 (-1.08-1.04) |
| Chlamydial infection | Botswana | 40.3 (27.8-56.3) | 3.8 (2.6-5.3) | 64.5 (42.6-96.7) | 2.4 (1.6-3.7) | -1.74 (-5.42-2.07) |
| Chlamydial infection | Brazil | 2700.3 (2046.4-3611.5) | 1.9 (1.5-2.6) | 6303 (4634.1-8639.7) | 2.6 (1.9-3.5) | -0.03 (-2.1-2.08) |
| Chlamydial infection | Brunei | 4 (2.5-5.9) | 1.6 (1-2.2) | 7.9 (5.1-12.4) | 1.5 (1-2.3) | 0.3 (-1.23-1.84) |
| Chlamydial infection | Bulgaria | 103.5 (66-157.3) | 1.2 (0.7-1.8) | 76.7 (49.3-115.8) | 1.2 (0.7-1.8) | -0.32 (-1.9-1.28) |
| Chlamydial infection | Burkina Faso | 321.5 (223.3-458.5) | 4.5 (3.1-6.5) | 525.6 (343.5-774.1) | 2.7 (1.8-4.1) | -2.04 (-5.04-1.06) |
| Chlamydial infection | Burundi | 234.6 (144.9-423.7) | 4.8 (3-8.5) | 405.2 (236.1-777.4) | 3.3 (1.9-6.2) | -2.38 (-5.42-0.76) |
| Chlamydial infection | Cambodia | 166.3 (103.5-257.4) | 1.9 (1.2-3) | 362.2 (216.6-565) | 2 (1.2-3.1) | 0.59 (-1.73-2.96) |
| Chlamydial infection | Cameroon | 402.8 (269.2-568.4) | 4.7 (3.2-6.7) | 860 (552.9-1244.9) | 2.9 (1.9-4.3) | -0.88 (-4.24-2.59) |
| Chlamydial infection | Canada | 223 (155.2-315.1) | 0.7 (0.5-1) | 234.3 (158.4-359.1) | 0.6 (0.4-1) | -0.98 (-2.6-0.68) |
| Chlamydial infection | Cape Verde | 7.1 (4.6-10.6) | 2.7 (1.7-4.1) | 15 (9.2-22.7) | 2.3 (1.4-3.5) | -0.94 (-3.55-1.73) |
| Chlamydial infection | Central African Republic | 95.2 (55.8-130.2) | 4.6 (2.7-6.3) | 182.4 (116.3-256.3) | 4.1 (2.6-5.7) | -0.8 (-4.45-2.99) |
| Chlamydial infection | Chad | 173.7 (117-246.6) | 3.7 (2.5-5.3) | 390.1 (260.5-569.3) | 2.9 (1.9-4.4) | -0.82 (-3.97-2.42) |
| Chlamydial infection | Chile | 203.9 (140.7-298.4) | 1.5 (1-2.2) | 288.4 (200.7-409.3) | 1.4 (1-2) | -0.28 (-2-1.48) |
| Chlamydial infection | China | 23597.2 (15631.7-34452.3) | 1.9 (1.3-2.8) | 25981.5 (16990.5-40017.1) | 1.7 (1.1-2.6) | 0.24 (-1.45-1.95) |
| Chlamydial infection | Colombia | 683.9 (500.7-959.3) | 2.2 (1.6-3) | 1080.9 (779.5-1529) | 2 (1.5-2.8) | -0.4 (-2.34-1.57) |
| Chlamydial infection | Comoros | 23.6 (11.4-40.1) | 5.7 (3.1-9.4) | 30.7 (19.4-50.4) | 4 (2.5-6.5) | -1.28 (-3.42-0.91) |
| Chlamydial infection | Congo | 69.7 (44.8-97.3) | 3.9 (2.5-5.5) | 148.8 (96.7-212.8) | 2.9 (1.9-4.1) | -1.1 (-4.52-2.45) |
| Chlamydial infection | Cook Islands | 0.1 (0.1-0.2) | 0.9 (0.6-1.3) | 0.1 (0.1-0.2) | 0.9 (0.5-1.3) | 0.67 (-1.53-2.91) |
| Chlamydial infection | Costa Rica | 54.3 (37.8-78.1) | 1.9 (1.3-2.7) | 103.4 (73.4-145.6) | 2 (1.4-2.8) | -0.32 (-2.16-1.55) |
| Chlamydial infection | Cote d'Ivoire | 367.7 (256.1-509.6) | 3.4 (2.4-4.8) | 700.4 (462.4-1018.9) | 2.7 (1.7-3.9) | -1.4 (-4.91-2.23) |
| Chlamydial infection | Croatia | 64.7 (40.7-97.2) | 1.2 (0.8-1.9) | 53.5 (34.5-82.4) | 1.3 (0.8-2) | -0.16 (-1.26-0.94) |
| Chlamydial infection | Cuba | 271.1 (191.1-386.8) | 2.3 (1.6-3.3) | 339.2 (258.7-456.8) | 2.9 (2.2-4) | 0.59 (-0.97-2.18) |
| Chlamydial infection | Cyprus | 3.4 (2.3-5.2) | 0.4 (0.3-0.7) | 4.8 (3.2-7) | 0.3 (0.2-0.4) | -0.24 (-1.59-1.14) |
| Chlamydial infection | Czech Republic | 138.7 (93-202.4) | 1.3 (0.9-1.9) | 126.5 (84.4-191.7) | 1.2 (0.8-1.8) | -0.29 (-1.18-0.62) |
| Chlamydial infection | Democratic Republic of the Congo | 1085.9 (692.4-1532.4) | 3.8 (2.4-5.4) | 2101.4 (1387.3-3183.4) | 2.8 (1.9-4.4) | -2.08 (-5.34-1.29) |
| Chlamydial infection | Denmark | 40.9 (33.9-50.5) | 0.6 (0.5-0.8) | 33.5 (26.7-41.9) | 0.5 (0.3-0.6) | -1.29 (-2.67-0.11) |
| Chlamydial infection | Djibouti | 13.5 (8.6-25.3) | 3.5 (2.2-6.6) | 42.8 (26.7-72.7) | 3.2 (2-5.5) | 1.17 (-2.08-4.54) |
| Chlamydial infection | Dominica | 1.2 (0.7-1.9) | 1.9 (1.1-2.9) | 1.3 (0.8-2) | 1.9 (1.2-2.9) | -0.12 (-2.68-2.5) |
| Chlamydial infection | Dominican Republic | 221.6 (159.4-299.9) | 3.3 (2.4-4.4) | 370.9 (243.6-539.1) | 3.2 (2.1-4.6) | -0.21 (-2.89-2.53) |
| Chlamydial infection | Ecuador | 171.9 (110.9-258.8) | 1.9 (1.2-2.8) | 377.2 (237-563.4) | 2 (1.3-3) | 0.41 (-2.02-2.91) |
| Chlamydial infection | Egypt | 768.9 (499.2-1155.5) | 1.6 (1.1-2.4) | 1465.2 (915.4-2248.9) | 1.4 (0.9-2.2) | -0.62 (-2.09-0.86) |
| Chlamydial infection | El Salvador | 75.9 (50.8-113) | 1.7 (1.1-2.5) | 106.6 (68.8-160) | 1.6 (1.1-2.4) | -0.37 (-2.5-1.8) |
| Chlamydial infection | Equatorial Guinea | 14.3 (8.2-21.4) | 4.6 (2.6-7) | 37.4 (24.1-54.2) | 2.8 (1.8-4.1) | -0.02 (-3.5-3.59) |
| Chlamydial infection | Eritrea | 147 (85.9-328.2) | 5.1 (3.1-10.8) | 247.4 (152-425.3) | 3.8 (2.4-6.4) | -1.11 (-3.94-1.81) |
| Chlamydial infection | Estonia | 33.6 (26-43.5) | 2 (1.5-2.6) | 20.5 (14.3-28.9) | 1.5 (1-2.2) | -1.5 (-3.11-0.14) |
| Chlamydial infection | Ethiopia | 3914.1 (1895-6992.3) | 9.2 (4.6-16.4) | 3962.8 (2381.8-9268.3) | 3.8 (2.3-8.5) | -2.58 (-5.44-0.36) |
| Chlamydial infection | Federated States of Micronesia | 0.9 (0.6-1.3) | 1 (0.7-1.6) | 1 (0.6-1.4) | 1 (0.6-1.4) | 1.06 (-1.48-3.66) |
| Chlamydial infection | Fiji | 11.5 (7.8-16.3) | 1.6 (1.1-2.4) | 11.4 (7.8-16.9) | 1.2 (0.8-1.8) | -0.09 (-2.68-2.57) |
| Chlamydial infection | Finland | 58.7 (48.8-72.8) | 1 (0.8-1.2) | 44.3 (33.9-58.4) | 0.7 (0.5-1) | -1.21 (-2.17--0.24) |
| Chlamydial infection | France | 278 (204.4-378.5) | 0.4 (0.3-0.6) | 248.2 (178.1-352.1) | 0.3 (0.2-0.5) | -1.25 (-3.08-0.62) |
| Chlamydial infection | Gabon | 25.2 (16.8-36) | 3.2 (2.1-4.6) | 42.7 (28.9-63.7) | 2.5 (1.7-3.7) | -1.25 (-4.56-2.18) |
| Chlamydial infection | Georgia | 128 (88.2-181.9) | 2.3 (1.6-3.2) | 83.8 (54.1-128.5) | 2.4 (1.5-3.7) | -0.74 (-1.97-0.52) |
| Chlamydial infection | Germany | 436.8 (329.7-575.1) | 0.5 (0.3-0.6) | 336.2 (242.7-475) | 0.4 (0.2-0.5) | -1.05 (-2.57-0.48) |
| Chlamydial infection | Ghana | 464.9 (317.3-657.9) | 3.6 (2.4-5) | 1093.7 (722.6-1592.4) | 3.2 (2.1-4.7) | -0.3 (-3.46-2.95) |
| Chlamydial infection | Greece | 47.7 (32.6-70.4) | 0.4 (0.3-0.7) | 58.4 (38.2-88.9) | 0.6 (0.4-0.9) | -0.26 (-1.4-0.9) |
| Chlamydial infection | Greenland | 0.4 (0.2-0.5) | 0.5 (0.3-0.8) | 0.3 (0.2-0.5) | 0.6 (0.3-0.9) | -0.61 (-2.62-1.43) |
| Chlamydial infection | Grenada | 1.6 (1.1-2.4) | 2.2 (1.5-3.3) | 2.7 (1.9-3.8) | 2.5 (1.8-3.5) | -0.33 (-2.33-1.71) |
| Chlamydial infection | Guam | 1.2 (0.8-1.8) | 0.8 (0.6-1.3) | 1.2 (0.7-1.9) | 0.8 (0.5-1.2) | 0.1 (-2.43-2.69) |
| Chlamydial infection | Guatemala | 140.7 (103.8-195.5) | 2.2 (1.6-3.1) | 321.6 (222.1-447.9) | 2 (1.4-2.9) | -0.57 (-2.56-1.47) |
| Chlamydial infection | Guinea | 197.7 (135.1-287) | 4 (2.7-6) | 338.7 (221.5-492.5) | 2.9 (1.9-4.3) | -0.39 (-3.49-2.82) |
| Chlamydial infection | Guinea-Bissau | 37.8 (22.6-57.4) | 4.6 (2.8-6.8) | 61.6 (39.5-90.5) | 3.2 (2.1-4.8) | 0.08 (-2.95-3.2) |
| Chlamydial infection | Guyana | 19.4 (13.9-26.9) | 2.7 (2-3.7) | 25.9 (19.2-34.8) | 3.3 (2.5-4.4) | -0.39 (-2.96-2.25) |
| Chlamydial infection | Haiti | 201.5 (135.6-291.8) | 3.7 (2.5-5.2) | 452.2 (261-670.1) | 3.5 (2-5.1) | -0.93 (-4.4-2.66) |
| Chlamydial infection | Honduras | 123.2 (87.2-166.6) | 3.4 (2.4-4.5) | 252.7 (175.8-374) | 2.6 (1.8-3.8) | -1.22 (-3.01-0.6) |
| Chlamydial infection | Hungary | 187.6 (139.5-254.3) | 1.7 (1.2-2.3) | 146.6 (104-205.2) | 1.4 (1-2) | -0.77 (-2.34-0.81) |
| Chlamydial infection | Iceland | 0.8 (0.6-1.1) | 0.3 (0.2-0.4) | 0.9 (0.7-1.3) | 0.2 (0.2-0.4) | -0.57 (-1.89-0.78) |
| Chlamydial infection | India | 26869 (18873.4-34406.7) | 3.5 (2.5-4.5) | 37677.6 (27306.7-49758.7) | 2.5 (1.8-3.3) | -0.13 (-2.5-2.3) |
| Chlamydial infection | Indonesia | 3277.4 (2100-4977) | 1.9 (1.2-2.9) | 5502.7 (3519.3-8457) | 1.8 (1.1-2.7) | 0.86 (-1.43-3.21) |
| Chlamydial infection | Iran | 559.9 (361.1-840.2) | 1.2 (0.8-1.9) | 1317.5 (830.7-1985.4) | 1.3 (0.8-1.9) | 0.76 (-0.72-2.25) |
| Chlamydial infection | Iraq | 222.9 (140.2-345.5) | 1.5 (1-2.2) | 577.4 (353.6-882.1) | 1.4 (0.9-2.1) | 0.3 (-1.27-1.89) |
| Chlamydial infection | Ireland | 16.7 (12.4-22.1) | 0.5 (0.3-0.6) | 18.2 (12.7-26.7) | 0.3 (0.2-0.5) | -0.74 (-2-0.54) |
| Chlamydial infection | Israel | 17.4 (11.9-25) | 0.4 (0.2-0.5) | 28.1 (17.9-42.3) | 0.3 (0.2-0.5) | -0.75 (-2.19-0.71) |
| Chlamydial infection | Italy | 462.9 (306.3-684.7) | 0.8 (0.5-1.1) | 669.8 (446.5-992.5) | 1.2 (0.8-1.7) | -1 (-2.77-0.8) |
| Chlamydial infection | Jamaica | 46.8 (32-68.1) | 2.2 (1.5-3.2) | 75 (51.3-106.7) | 2.4 (1.7-3.4) | -0.52 (-2.87-1.9) |
| Chlamydial infection | Japan | 1828.2 (1154.9-2848.9) | 1.3 (0.8-2.1) | 1912.4 (1266.6-2857.5) | 1.6 (1-2.4) | 0.26 (-0.63-1.16) |
| Chlamydial infection | Jordan | 24.4 (14.9-36.9) | 0.8 (0.5-1.2) | 100 (61.8-158.1) | 0.8 (0.5-1.2) | 0.37 (-1.37-2.14) |
| Chlamydial infection | Kazakhstan | 446.4 (323.2-622.8) | 2.7 (2-3.8) | 429.4 (285.2-637.3) | 2.2 (1.4-3.2) | -0.99 (-2.48-0.52) |
| Chlamydial infection | Kenya | 693.5 (496.2-1015.4) | 3.5 (2.5-5.2) | 1488.9 (1017.2-2245.5) | 3 (2-4.5) | -1.74 (-5.24-1.9) |
| Chlamydial infection | Kiribati | 0.7 (0.5-1) | 1.1 (0.8-1.6) | 1.2 (0.8-1.9) | 1.1 (0.7-1.6) | -0.21 (-2.84-2.5) |
| Chlamydial infection | Kuwait | 33.3 (18.7-52.8) | 1.5 (0.9-2.4) | 103.4 (61.9-160.1) | 1.6 (1-2.4) | 0.22 (-0.7-1.15) |
| Chlamydial infection | Kyrgyzstan | 112.8 (85-152.2) | 2.9 (2.2-3.9) | 146.4 (93.5-220.3) | 2.1 (1.4-3.2) | -1.59 (-3.22-0.08) |
| Chlamydial infection | Laos | 72.2 (43.1-111.6) | 2.1 (1.2-3.3) | 164.6 (103.1-250.5) | 2.1 (1.3-3.1) | 2.19 (0.18-4.25) |
| Chlamydial infection | Latvia | 64.8 (51.4-82.8) | 2.3 (1.8-2.9) | 33.8 (24.3-47.4) | 1.7 (1.2-2.4) | -1.73 (-3.64-0.23) |
| Chlamydial infection | Lebanon | 38.9 (25.9-57.9) | 1.4 (1-2.1) | 85.1 (52.4-131) | 1.3 (0.8-2) | -0.08 (-1.82-1.69) |
| Chlamydial infection | Lesotho | 40.3 (27.4-59.4) | 3.3 (2.2-4.9) | 59.9 (40.6-86.8) | 3.3 (2.2-4.7) | 0.22 (-3.54-4.13) |
| Chlamydial infection | Liberia | 80.6 (54.4-113.5) | 3.8 (2.5-5.3) | 156.8 (96.5-234.5) | 3 (1.9-4.4) | 0.18 (-3.18-3.66) |
| Chlamydial infection | Libya | 45.5 (27.1-69.9) | 1.3 (0.8-2) | 107.9 (66.4-166.9) | 1.3 (0.8-2) | 0.28 (-1.35-1.94) |
| Chlamydial infection | Lithuania | 57.8 (41.1-81.7) | 1.5 (1.1-2.1) | 36.8 (24.6-53.9) | 1.3 (0.9-2) | -0.28 (-1.96-1.42) |
| Chlamydial infection | Luxembourg | 2.4 (1.8-3.2) | 0.5 (0.4-0.7) | 3.2 (2.1-4.6) | 0.4 (0.3-0.6) | -1.35 (-2.76-0.09) |
| Chlamydial infection | Macedonia | 25.8 (17-38) | 1.2 (0.8-1.8) | 28 (17.3-42.3) | 1.2 (0.7-1.7) | -0.35 (-1.35-0.66) |
| Chlamydial infection | Madagascar | 665.3 (374.1-955.4) | 6.2 (3.7-8.7) | 1403.9 (889.7-2002.3) | 5 (3.3-6.9) | 1.41 (-1.31-4.21) |
| Chlamydial infection | Malawi | 438.4 (269.1-646.8) | 5 (3.2-7.1) | 724.7 (462.8-1127) | 3.9 (2.5-5.7) | -1.51 (-5.09-2.2) |
| Chlamydial infection | Malaysia | 355.8 (219.5-549) | 2.1 (1.3-3.1) | 718.3 (435.8-1108) | 2 (1.2-3.1) | -0.02 (-1.99-1.99) |
| Chlamydial infection | Maldives | 5.3 (3.6-7.3) | 3.1 (2.2-4.3) | 18.2 (10.7-29.1) | 2.5 (1.5-3.8) | 0.19 (-1.28-1.68) |
| Chlamydial infection | Mali | 391.5 (260.9-556.6) | 5.7 (3.8-8.1) | 579.7 (381.2-833.6) | 3 (2-4.3) | -0.61 (-3.63-2.51) |
| Chlamydial infection | Malta | 1.2 (0.9-1.7) | 0.3 (0.2-0.4) | 1.2 (0.9-1.8) | 0.2 (0.2-0.4) | -0.46 (-1.82-0.92) |
| Chlamydial infection | Marshall Islands | 0.3 (0.2-0.5) | 0.9 (0.6-1.4) | 0.5 (0.4-0.8) | 0.9 (0.6-1.4) | 1.27 (-1.19-3.79) |
| Chlamydial infection | Mauritania | 63.2 (42.5-91.1) | 3.7 (2.5-5.3) | 103.4 (68.7-152.8) | 2.7 (1.8-4) | -0.99 (-3.24-1.32) |
| Chlamydial infection | Mauritius | 22.1 (12.2-34.9) | 1.8 (1-2.8) | 26.9 (16.9-41.7) | 2 (1.2-3.1) | 2.09 (0.34-3.86) |
| Chlamydial infection | Mexico | 1633.9 (1240.4-2218) | 2.2 (1.7-3) | 3321.2 (2485-4551.4) | 2.4 (1.8-3.3) | -0.16 (-2.13-1.85) |
| Chlamydial infection | Moldova | 103.4 (79.3-134.5) | 2.3 (1.8-2.9) | 71 (52.1-99.3) | 1.7 (1.2-2.3) | -0.76 (-2.62-1.14) |
| Chlamydial infection | Monaco | 0.1 (0.1-0.2) | 0.3 (0.2-0.5) | 0.1 (0.1-0.2) | 0.3 (0.2-0.5) | -0.69 (-1.99-0.62) |
| Chlamydial infection | Mongolia | 73.2 (45.6-120.9) | 4.2 (2.6-7.2) | 97.3 (66-137.7) | 2.8 (1.9-4) | 0.06 (-2.01-2.17) |
| Chlamydial infection | Montenegro | 7.6 (4.9-11.8) | 1.2 (0.7-1.8) | 7.1 (4.6-11.1) | 1.1 (0.7-1.8) | -0.19 (-1.37-1.01) |
| Chlamydial infection | Morocco | 525.2 (309.4-803.1) | 2.3 (1.4-3.4) | 820.7 (507.1-1275.4) | 2.1 (1.3-3.3) | 0.05 (-2.75-2.94) |
| Chlamydial infection | Mozambique | 992.9 (507.8-1555.6) | 8.5 (4.6-13) | 1416.6 (869.4-2309) | 5 (3.2-7.8) | -0.91 (-4.37-2.67) |
| Chlamydial infection | Myanmar | 862 (540.6-1341.1) | 2.2 (1.4-3.4) | 1226.1 (784.3-1841.2) | 2.1 (1.4-3.2) | 0.78 (-1.77-3.39) |
| Chlamydial infection | Namibia | 38.5 (26.8-54.2) | 3.3 (2.3-4.6) | 63.8 (41.8-92.8) | 2.6 (1.8-3.9) | -0.65 (-4.11-2.94) |
| Chlamydial infection | Nauru | 0.1 (0.1-0.1) | 1 (0.7-1.5) | 0.1 (0.1-0.2) | 1 (0.6-1.4) | 1.14 (-1.44-3.79) |
| Chlamydial infection | Nepal | 520.1 (348.4-757.8) | 3.3 (2.2-4.8) | 791.6 (555.6-1098.1) | 2.6 (1.8-3.6) | 1.4 (-1.18-4.06) |
| Chlamydial infection | Netherlands | 75.1 (55.3-103.5) | 0.4 (0.3-0.6) | 62.1 (43.9-87.4) | 0.3 (0.2-0.5) | -1.07 (-2.58-0.46) |
| Chlamydial infection | New Zealand | 66 (42-102.2) | 1.8 (1.1-2.8) | 111.5 (66.9-170.9) | 2.2 (1.3-3.3) | -0.6 (-1.83-0.66) |
| Chlamydial infection | Nicaragua | 55.5 (37.1-79.7) | 1.8 (1.2-2.5) | 120.4 (78.1-182.8) | 1.7 (1.1-2.6) | -0.57 (-2.42-1.33) |
| Chlamydial infection | Niger | 223.3 (149.3-332.1) | 3.6 (2.4-5.3) | 466 (301.4-694.9) | 2.6 (1.6-3.9) | -1.35 (-3.77-1.12) |
| Chlamydial infection | Nigeria | 2317.4 (1556.8-3338.9) | 3 (2-4.4) | 4658.7 (3177.2-6937.4) | 2.4 (1.6-3.5) | -0.33 (-3.34-2.76) |
| Chlamydial infection | Niue | 0 (0-0) | 0.9 (0.6-1.4) | 0 (0-0) | 0.9 (0.6-1.4) | 0.93 (-1.36-3.28) |
| Chlamydial infection | North Korea | 358.6 (219.6-556.7) | 1.7 (1.1-2.6) | 524.5 (315.9-827) | 1.8 (1.1-2.8) | 1.29 (-0.41-3.01) |
| Chlamydial infection | Northern Mariana Islands | 0.5 (0.4-0.8) | 1 (0.6-1.4) | 0.4 (0.3-0.6) | 0.8 (0.5-1.2) | -0.16 (-2.48-2.21) |
| Chlamydial infection | Norway | 42.8 (26.9-64.6) | 0.9 (0.6-1.4) | 54.1 (35.7-79.4) | 1 (0.6-1.4) | -0.07 (-1.31-1.18) |
| Chlamydial infection | Oman | 27.7 (15.1-44.8) | 1.4 (0.8-2.2) | 89 (50.8-140.9) | 1.3 (0.8-2.1) | 0.1 (-1.3-1.52) |
| Chlamydial infection | Pakistan | 1701.2 (1165.9-2509.7) | 2.1 (1.4-3.2) | 4524.3 (3203.3-6449.4) | 2.1 (1.5-3) | 2.13 (-0.38-4.71) |
| Chlamydial infection | Palau | 0.1 (0.1-0.2) | 0.9 (0.6-1.3) | 0.2 (0.1-0.2) | 0.7 (0.5-1.1) | 0.87 (-1.2-2.98) |
| Chlamydial infection | Palestine | 21.4 (13.6-33.9) | 1.4 (0.9-2.2) | 68 (43.7-107.6) | 1.4 (0.9-2.1) | 0.18 (-1.25-1.62) |
| Chlamydial infection | Panama | 44.6 (31.2-63.4) | 1.9 (1.4-2.7) | 81.4 (56.7-118) | 1.9 (1.3-2.7) | -0.19 (-2.52-2.2) |
| Chlamydial infection | Papua New Guinea | 55.3 (37-79.7) | 1.6 (1.1-2.3) | 100.8 (65.6-149.2) | 1 (0.7-1.5) | 0.95 (-1.92-3.91) |
| Chlamydial infection | Paraguay | 79.1 (57.5-108) | 2.3 (1.7-3.1) | 160.6 (115.6-222.4) | 2.1 (1.5-3) | 0.55 (-2.01-3.19) |
| Chlamydial infection | Peru | 405.3 (281.7-583.1) | 2 (1.4-2.9) | 778.5 (503.8-1136.3) | 2 (1.3-2.9) | -1.14 (-3.45-1.23) |
| Chlamydial infection | Philippines | 1133.9 (734.6-1719.1) | 2 (1.3-3) | 2666.3 (1704-3990.5) | 2.3 (1.5-3.4) | 2.65 (0.47-4.88) |
| Chlamydial infection | Poland | 554.9 (381.7-798) | 1.4 (0.9-1.9) | 507.6 (323.4-758.7) | 1.3 (0.8-1.9) | 0.44 (-0.67-1.57) |
| Chlamydial infection | Portugal | 37.3 (29.8-48.6) | 0.3 (0.3-0.4) | 36.2 (26-51.7) | 0.3 (0.2-0.4) | -0.97 (-3.21-1.33) |
| Chlamydial infection | Puerto Rico | 73 (47.9-109.8) | 2 (1.3-3) | 66.6 (44.8-98) | 2.1 (1.4-3.1) | -1.18 (-3.47-1.17) |
| Chlamydial infection | Qatar | 9.4 (5.4-15.3) | 1.7 (1.1-2.7) | 71.1 (38.9-114) | 1.5 (0.9-2.3) | -0.84 (-2.44-0.79) |
| Chlamydial infection | Romania | 286.3 (186.5-436.5) | 1.2 (0.8-1.9) | 213.2 (138.1-326.5) | 1.2 (0.7-1.8) | -0.64 (-2.33-1.08) |
| Chlamydial infection | Russian Federation | 3029.9 (2348.8-4002) | 1.9 (1.4-2.5) | 2814 (2154-3720.3) | 1.7 (1.3-2.3) | -0.01 (-2.12-2.15) |
| Chlamydial infection | Rwanda | 442.3 (260.6-707) | 6.9 (4.2-10.7) | 446.4 (275.2-767.6) | 3.4 (2.1-5.6) | -2.13 (-5.1-0.94) |
| Chlamydial infection | Saint Kitts and Nevis | 1.1 (0.8-1.5) | 2.8 (2.1-3.8) | 1.8 (1.3-2.5) | 2.7 (1.9-3.7) | -0.04 (-2.46-2.43) |
| Chlamydial infection | Saint Lucia | 3.2 (2.3-4.4) | 2.7 (1.9-3.6) | 5.9 (4.2-7.9) | 2.9 (2.1-4) | -0.33 (-2.21-1.58) |
| Chlamydial infection | Saint Vincent and the Grenadines | 2 (1.3-3) | 2 (1.3-3) | 2.5 (1.7-3.6) | 2.1 (1.4-3.1) | -0.95 (-3.39-1.54) |
| Chlamydial infection | Samoa | 1.2 (0.8-1.8) | 0.9 (0.6-1.4) | 1.6 (1-2.5) | 0.9 (0.6-1.3) | 1 (-0.74-2.77) |
| Chlamydial infection | San Marino | 0.1 (0.1-0.1) | 0.3 (0.2-0.5) | 0.1 (0.1-0.1) | 0.3 (0.2-0.5) | -0.63 (-1.96-0.72) |
| Chlamydial infection | Sao Tome and Principe | 2.4 (1.6-3.5) | 2.7 (1.7-3.9) | 5.3 (3.5-7.9) | 2.5 (1.6-3.7) | -0.96 (-2.73-0.83) |
| Chlamydial infection | Saudi Arabia | 196.6 (117.9-303.6) | 1.3 (0.8-2) | 664.4 (398.9-1077.1) | 1.2 (0.7-1.9) | -0.08 (-1.72-1.6) |
| Chlamydial infection | Senegal | 226.8 (149.9-329.2) | 3.7 (2.5-5.5) | 341.6 (219.7-500) | 2.4 (1.5-3.5) | -1.3 (-3.87-1.34) |
| Chlamydial infection | Serbia | 116.2 (74.7-168) | 1.2 (0.8-1.7) | 104.1 (67.4-159.1) | 1.1 (0.7-1.7) | -0.21 (-1.49-1.08) |
| Chlamydial infection | Seychelles | 2.2 (1.6-3) | 3.2 (2.2-4.3) | 2.6 (1.7-3.9) | 2.3 (1.5-3.5) | 0.46 (-1.38-2.33) |
| Chlamydial infection | Sierra Leone | 114.6 (75.7-167.6) | 3.1 (2-4.5) | 224.5 (149.8-326.7) | 2.7 (1.8-3.9) | -0.02 (-2.85-2.89) |
| Chlamydial infection | Singapore | 54.9 (34.6-85.4) | 1.5 (1-2.3) | 100.8 (62.7-161) | 1.5 (0.9-2.3) | -0.6 (-1.88-0.7) |
| Chlamydial infection | Slovakia | 68.8 (46.8-102.1) | 1.2 (0.8-1.8) | 66.8 (43.2-101) | 1.1 (0.7-1.7) | -0.14 (-1-0.73) |
| Chlamydial infection | Slovenia | 24.6 (16.1-36.8) | 1.1 (0.7-1.7) | 21.3 (13.7-32.3) | 1.1 (0.7-1.6) | -0.55 (-1.52-0.43) |
| Chlamydial infection | Solomon Islands | 2.8 (1.9-3.9) | 1.1 (0.8-1.6) | 6.4 (4.3-9.5) | 1 (0.7-1.5) | 0.76 (-1.95-3.55) |
| Chlamydial infection | Somalia | 424.9 (244-831.3) | 6.4 (3.7-12.9) | 1065.3 (602.4-2054.1) | 5.7 (3.3-10.6) | 0.62 (-2.04-3.36) |
| Chlamydial infection | South Africa | 2171.1 (1503.4-2869.6) | 6.1 (4.3-8.1) | 2310.9 (1632.1-3213.9) | 3.6 (2.6-5) | -0.51 (-3.9-3.01) |
| Chlamydial infection | South Korea | 711.3 (438.3-1067.8) | 1.4 (0.9-2.2) | 761.8 (474.7-1186.8) | 1.4 (0.9-2.2) | -0.2 (-1.6-1.21) |
| Chlamydial infection | South Sudan | 268.2 (166.3-484.7) | 5 (3.3-8.7) | 463.7 (264.7-903.1) | 5.3 (3.2-10.2) | 0.59 (-2.73-4.02) |
| Chlamydial infection | Spain | 177.9 (137.8-231.4) | 0.4 (0.3-0.6) | 257 (181.7-370) | 0.5 (0.3-0.7) | -1.37 (-3.39-0.69) |
| Chlamydial infection | Sri Lanka | 392.3 (256-571.9) | 2.2 (1.5-3.2) | 437.8 (274.6-676.9) | 1.9 (1.2-3) | -0.03 (-1.25-1.21) |
| Chlamydial infection | Sudan | 220.4 (133.1-343.3) | 1.3 (0.8-2.1) | 560.5 (343.3-882.5) | 1.4 (0.9-2.1) | 0.45 (-2.48-3.47) |
| Chlamydial infection | Suriname | 8.2 (5.4-12.1) | 2.2 (1.4-3.2) | 13 (8.7-19.5) | 2.2 (1.5-3.3) | -0.25 (-2.76-2.33) |
| Chlamydial infection | Swaziland | 24.8 (17.7-33) | 4 (2.9-5.2) | 34.2 (22.2-51.6) | 3 (2-4.4) | 0.17 (-3.5-3.98) |
| Chlamydial infection | Sweden | 29.7 (21.3-42.2) | 0.3 (0.2-0.5) | 37.6 (26.7-53.4) | 0.3 (0.2-0.5) | -0.61 (-1.86-0.66) |
| Chlamydial infection | Switzerland | 46.2 (34.7-63.5) | 0.6 (0.4-0.8) | 49.4 (35.3-71.7) | 0.5 (0.3-0.7) | -1.31 (-2.83-0.24) |
| Chlamydial infection | Syria | 220 (160.2-302.1) | 2.3 (1.7-3.3) | 231.1 (162-336.5) | 1.8 (1.2-2.6) | -0.23 (-1.72-1.29) |
| Chlamydial infection | Taiwan | 389.8 (232.4-626.9) | 1.7 (1-2.7) | 428.2 (262.8-671) | 1.7 (1-2.6) | 0.52 (-0.69-1.74) |
| Chlamydial infection | Tajikistan | 136.2 (91.4-191.2) | 3.1 (2.1-4.4) | 241.8 (156.4-369) | 2.4 (1.6-3.6) | -0.83 (-2.36-0.73) |
| Chlamydial infection | Tanzania | 1340.5 (781.6-2025.6) | 5.9 (3.6-8.7) | 2106.9 (1366.3-3228) | 3.9 (2.6-5.8) | -1.93 (-5.41-1.68) |
| Chlamydial infection | Thailand | 1284.3 (829.1-1970.8) | 2.1 (1.4-3.2) | 1308.9 (827.1-2059.7) | 1.9 (1.2-3) | -1.84 (-4.44-0.83) |
| Chlamydial infection | The Bahamas | 5.9 (3.9-8.4) | 2.2 (1.5-3.2) | 10.6 (7.4-14.9) | 2.5 (1.7-3.5) | -0.2 (-3-2.68) |
| Chlamydial infection | The Gambia | 23 (15.4-33.1) | 2.7 (1.8-4) | 58.6 (37.8-87.1) | 2.7 (1.7-4) | 0.52 (-2.59-3.73) |
| Chlamydial infection | Timor-Leste | 14.9 (9-23.6) | 2.1 (1.3-3.2) | 25.5 (16.1-38.3) | 2 (1.3-3.1) | 0.7 (-1.77-3.23) |
| Chlamydial infection | Togo | 107.4 (72.6-154.3) | 3.6 (2.4-5.2) | 216.4 (139.5-321.9) | 2.7 (1.8-4.1) | -1.34 (-4.49-1.91) |
| Chlamydial infection | Tokelau | 0 (0-0) | 1 (0.6-1.5) | 0 (0-0) | 0.9 (0.6-1.4) | 0.88 (-1.44-3.25) |
| Chlamydial infection | Tonga | 0.7 (0.5-1.1) | 1 (0.6-1.5) | 0.8 (0.5-1.2) | 0.9 (0.6-1.4) | -0.18 (-2.35-2.03) |
| Chlamydial infection | Trinidad and Tobago | 26.7 (17.6-39.7) | 2.2 (1.5-3.2) | 33.6 (23-48.8) | 2.2 (1.5-3.2) | -0.61 (-3.06-1.9) |
| Chlamydial infection | Tunisia | 98.6 (60.4-157.5) | 1.3 (0.8-2.1) | 164 (100.4-260.6) | 1.3 (0.8-2) | 0.49 (-1-2) |
| Chlamydial infection | Turkey | 789.2 (484.8-1212.8) | 1.4 (0.9-2.2) | 1298.2 (770.3-1992.9) | 1.4 (0.8-2.2) | 0.12 (-1.61-1.89) |
| Chlamydial infection | Turkmenistan | 94.3 (71.2-128.2) | 3 (2.3-4) | 125.4 (83.4-180.5) | 2.3 (1.6-3.3) | -1.44 (-3.19-0.34) |
| Chlamydial infection | Tuvalu | 0.1 (0.1-0.1) | 1.1 (0.7-1.6) | 0.1 (0.1-0.2) | 0.9 (0.6-1.3) | 0.58 (-1.59-2.81) |
| Chlamydial infection | Uganda | 504 (317-796.4) | 3.5 (2.3-5.5) | 1536.7 (994.5-2183.8) | 3.9 (2.6-5.4) | -0.91 (-4.47-2.78) |
| Chlamydial infection | Ukraine | 840.8 (608.2-1182.5) | 1.5 (1.1-2.1) | 745.8 (515.8-1028.5) | 1.5 (1-2.1) | -0.04 (-2.37-2.33) |
| Chlamydial infection | United Arab Emirates | 40.1 (24-63.1) | 1.9 (1.2-2.8) | 222.1 (131-367.7) | 1.6 (1.1-2.5) | -0.05 (-1.32-1.24) |
| Chlamydial infection | United Kingdom | 655.8 (470.1-912.3) | 1 (0.7-1.5) | 1064.7 (732.6-1551.9) | 1.5 (1-2.3) | -0.05 (-1.53-1.45) |
| Chlamydial infection | United States | 2576.9 (1862-3611.2) | 0.9 (0.6-1.3) | 2285 (1632.8-3161.9) | 0.7 (0.5-0.9) | -1.38 (-3.39-0.67) |
| Chlamydial infection | Uruguay | 50 (36.4-68.4) | 1.6 (1.2-2.2) | 58.1 (41.8-82.4) | 1.6 (1.1-2.3) | -0.43 (-2.37-1.55) |
| Chlamydial infection | Uzbekistan | 462.2 (326.7-641) | 2.6 (1.9-3.5) | 763.9 (483.1-1160) | 2.1 (1.3-3.1) | -0.92 (-2.4-0.58) |
| Chlamydial infection | Vanuatu | 1.2 (0.8-1.8) | 1 (0.7-1.5) | 2.7 (1.8-4.1) | 0.9 (0.6-1.4) | 1.02 (-1.61-3.72) |
| Chlamydial infection | Venezuela | 348.2 (243-498.5) | 2 (1.4-2.8) | 561.8 (400.8-795) | 2 (1.5-2.9) | -0.11 (-2.12-1.94) |
| Chlamydial infection | Vietnam | 1127 (663.5-1732.6) | 1.8 (1.1-2.8) | 2094.7 (1221.9-3293.2) | 1.9 (1.1-2.9) | 0.36 (-1.83-2.61) |
| Chlamydial infection | Yemen | 131 (77.3-204.9) | 1.3 (0.8-2.1) | 414.2 (257.3-633.4) | 1.4 (0.9-2.1) | 0.77 (-0.94-2.51) |
| Chlamydial infection | Zambia | 463.2 (234.4-692.4) | 6.5 (3.6-9.1) | 841.9 (491.4-1563.9) | 4.4 (2.7-7.8) | -1.45 (-5.04-2.27) |
| Chlamydial infection | Zimbabwe | 206.1 (132.3-341.3) | 2.6 (1.7-4.2) | 414.9 (271.8-671.2) | 2.9 (1.9-4.7) | -0.98 (-4.66-2.84) |
| Genital herpes | Afghanistan | 171.6 (75.7-342.8) | 2.4 (1-4.8) | 471.5 (203.7-948.7) | 2.3 (1-4.8) | 0.27 (-1.62-2.2) |
| Genital herpes | Albania | 43.8 (18.7-88.2) | 1.6 (0.7-3.1) | 53.1 (22.2-108.1) | 1.6 (0.7-3.2) | -0.44 (-1.52-0.65) |
| Genital herpes | Algeria | 449.5 (198.6-925.5) | 2.5 (1.1-5.2) | 1106.4 (473-2220.4) | 2.5 (1.1-5) | -0.05 (-1.71-1.63) |
| Genital herpes | American Samoa | 1.6 (0.7-3.3) | 4.3 (1.8-8.7) | 2.2 (0.9-4.3) | 4.3 (1.8-8.6) | 0.22 (-2.25-2.76) |
| Genital herpes | Andorra | 1.4 (0.6-2.7) | 2.1 (0.9-4.3) | 2.5 (1-5.1) | 2.1 (0.9-4.3) | -0.81 (-2.23-0.63) |
| Genital herpes | Angola | 562.4 (247.7-1103.7) | 7.7 (3.3-15.1) | 1838.5 (790.6-3657.2) | 8 (3.4-15.7) | 0.25 (-2.86-3.45) |
| Genital herpes | Antigua and Barbuda | 3.1 (1.3-6.1) | 5.5 (2.3-10.9) | 5.8 (2.4-11.4) | 5.5 (2.3-10.8) | -0.3 (-2.48-1.93) |
| Genital herpes | Argentina | 1394 (604.4-2844.5) | 4.4 (1.9-8.9) | 2086.7 (894.9-4173.3) | 4.1 (1.8-8.3) | -0.36 (-2.29-1.61) |
| Genital herpes | Armenia | 68.6 (28.7-134.2) | 2.1 (0.9-4.2) | 79 (32.3-154.4) | 2.1 (0.9-4.2) | -1.22 (-2.26--0.18) |
| Genital herpes | Australia | 637.4 (271.9-1308.8) | 3.5 (1.5-7.1) | 812.9 (338-1640) | 2.6 (1.1-5.3) | -1.33 (-2.67-0.02) |
| Genital herpes | Austria | 180.3 (76-360.5) | 1.9 (0.8-3.9) | 229.1 (95-460.8) | 1.9 (0.8-3.9) | -0.88 (-2.29-0.55) |
| Genital herpes | Azerbaijan | 133.1 (56.6-261.7) | 2.1 (0.9-4.3) | 255.8 (106.6-512.7) | 2.1 (0.9-4.3) | -1.22 (-2.53-0.1) |
| Genital herpes | Bahrain | 10.4 (4.4-21.1) | 2.3 (1-4.7) | 40 (17.3-80.9) | 2.3 (1-4.6) | -0.19 (-2.07-1.73) |
| Genital herpes | Bangladesh | 1201.5 (512.4-2458.8) | 1.7 (0.7-3.5) | 2772.2 (1163-5491.8) | 1.7 (0.7-3.4) | 0.15 (-2.13-2.49) |
| Genital herpes | Barbados | 14.3 (6-28.4) | 5.5 (2.3-11) | 20.6 (8.6-41.1) | 5.5 (2.3-11.1) | -0.79 (-3-1.47) |
| Genital herpes | Belarus | 364.8 (153.3-721.1) | 3.1 (1.3-6.2) | 370.8 (154.1-744.9) | 3.1 (1.3-6.4) | -0.45 (-2.15-1.29) |
| Genital herpes | Belgium | 261.1 (108.5-523.5) | 2.2 (0.9-4.4) | 315 (128.7-633.4) | 2.2 (0.9-4.4) | -0.7 (-2.22-0.85) |
| Genital herpes | Belize | 6.9 (3-13.8) | 5.4 (2.3-10.8) | 22.3 (9.3-45.2) | 5.5 (2.3-10.9) | -0.05 (-2.66-2.64) |
| Genital herpes | Benin | 156.8 (68.5-314.5) | 5.1 (2.2-10.1) | 471.8 (201.9-948.4) | 5.1 (2.1-10.1) | -0.41 (-3.25-2.52) |
| Genital herpes | Bermuda | 3.8 (1.6-7.5) | 5.5 (2.3-11) | 4.6 (1.9-9.4) | 5.5 (2.3-11.1) | -0.54 (-2.84-1.81) |
| Genital herpes | Bhutan | 7 (3-13.7) | 1.7 (0.7-3.3) | 13 (5.4-26.3) | 1.7 (0.7-3.5) | -0.07 (-2.86-2.8) |
| Genital herpes | Bolivia | 285.7 (123-566.7) | 6 (2.6-11.9) | 674 (293.5-1354.9) | 5.9 (2.6-11.7) | 0.06 (-2.78-2.98) |
| Genital herpes | Bosnia and Herzegovina | 75.5 (32.2-151.7) | 1.6 (0.7-3.2) | 70.6 (29.4-142) | 1.6 (0.7-3.2) | -0.03 (-1.08-1.04) |
| Genital herpes | Botswana | 67.7 (29.6-135.2) | 7.3 (3.1-14.7) | 183.9 (78.4-367.5) | 7.7 (3.2-15.4) | -1.74 (-5.42-2.07) |
| Genital herpes | Brazil | 8034.1 (3402.5-16018.9) | 6.3 (2.7-12.6) | 15724.3 (6564.9-31011.4) | 6.3 (2.6-12.3) | -0.03 (-2.1-2.08) |
| Genital herpes | Brunei | 4.6 (2-9.2) | 2.2 (0.9-4.4) | 11.4 (4.8-23.1) | 2.3 (1-4.6) | 0.3 (-1.23-1.84) |
| Genital herpes | Bulgaria | 222.9 (92.9-439) | 2.1 (0.9-4.2) | 198.6 (82.7-399) | 2.1 (0.9-4.3) | -0.32 (-1.9-1.28) |
| Genital herpes | Burkina Faso | 324.8 (141.5-661.7) | 5.3 (2.3-10.8) | 818.4 (354.5-1626.9) | 5.2 (2.2-10.4) | -2.04 (-5.04-1.06) |
| Genital herpes | Burundi | 266.6 (113.3-543.7) | 6.9 (2.9-14) | 661.8 (291.7-1320) | 6.9 (3-13.9) | -2.38 (-5.42-0.76) |
| Genital herpes | Cambodia | 264.4 (116.1-525.6) | 3.7 (1.6-7.5) | 607.9 (256.4-1221.4) | 3.7 (1.6-7.5) | 0.59 (-1.73-2.96) |
| Genital herpes | Cameroon | 461 (200-921.5) | 6.5 (2.8-13) | 1539.2 (678.9-3059.7) | 6.5 (2.9-12.9) | -0.88 (-4.24-2.59) |
| Genital herpes | Canada | 686.4 (288.8-1373.7) | 2.2 (0.9-4.4) | 1038.2 (432.7-2034.6) | 2.2 (0.9-4.4) | -0.98 (-2.6-0.68) |
| Genital herpes | Cape Verde | 13.2 (5.6-26.3) | 5.5 (2.3-11.1) | 29.8 (12.8-59.6) | 5.2 (2.2-10.3) | -0.94 (-3.55-1.73) |
| Genital herpes | Central African Republic | 157 (69.8-308.7) | 7.9 (3.4-15.5) | 336.2 (147.5-675.5) | 7.9 (3.4-16) | -0.8 (-4.45-2.99) |
| Genital herpes | Chad | 177.2 (77.4-358.6) | 4.5 (1.9-9.1) | 480.5 (209.7-965.6) | 4.5 (1.9-9.1) | -0.82 (-3.97-2.42) |
| Genital herpes | Chile | 513.1 (219.5-1043.7) | 4.1 (1.7-8.3) | 904.5 (374.7-1834.4) | 4.1 (1.7-8.3) | -0.28 (-2-1.48) |
| Genital herpes | China | 25609.8 (10753.2-51458.2) | 2.3 (1-4.7) | 43492.7 (18133.6-86751.9) | 2.3 (1-4.7) | 0.24 (-1.45-1.95) |
| Genital herpes | Colombia | 1847.2 (820.3-3676.8) | 6.8 (3-13.7) | 3207.7 (1340.5-6306.6) | 5.9 (2.5-11.6) | -0.4 (-2.34-1.57) |
| Genital herpes | Comoros | 21.9 (9.7-43.5) | 6.8 (2.9-13.7) | 47.1 (20.2-94.7) | 6.8 (2.9-13.8) | -1.28 (-3.42-0.91) |
| Genital herpes | Congo | 137.5 (61.6-278) | 7.9 (3.4-16.2) | 371.8 (159.8-740.6) | 7.8 (3.3-15.5) | -1.1 (-4.52-2.45) |
| Genital herpes | Cook Islands | 0.7 (0.3-1.4) | 4.3 (1.8-8.6) | 0.9 (0.4-1.7) | 4.3 (1.8-8.7) | 0.67 (-1.53-2.91) |
| Genital herpes | Costa Rica | 146.2 (65.2-293.3) | 5.8 (2.6-11.8) | 296.1 (124-594.5) | 5.5 (2.3-11.1) | -0.32 (-2.16-1.55) |
| Genital herpes | Cote d'Ivoire | 419.6 (182.4-861.1) | 5.1 (2.2-10.3) | 1099.2 (472.5-2181.8) | 5.2 (2.2-10.3) | -1.4 (-4.91-2.23) |
| Genital herpes | Croatia | 103.3 (42.9-206.1) | 1.8 (0.7-3.6) | 101.2 (41.8-203.5) | 1.8 (0.7-3.5) | -0.16 (-1.26-0.94) |
| Genital herpes | Cuba | 598.5 (257-1201.9) | 5.4 (2.3-10.8) | 777.5 (320.4-1559.7) | 5.4 (2.3-10.9) | 0.59 (-0.97-2.18) |
| Genital herpes | Cyprus | 17.7 (7.4-35.7) | 2.2 (0.9-4.4) | 37.7 (15.8-76.4) | 2.2 (0.9-4.4) | -0.24 (-1.59-1.14) |
| Genital herpes | Czech Republic | 142.9 (62.8-287.7) | 1.2 (0.5-2.4) | 200.4 (82.5-400.5) | 1.4 (0.6-2.8) | -0.29 (-1.18-0.62) |
| Genital herpes | Democratic Republic of the Congo | 2083.1 (911.7-4087.3) | 7.8 (3.3-15.4) | 5281.2 (2319.4-10592) | 7.7 (3.3-15.4) | -2.08 (-5.34-1.29) |
| Genital herpes | Denmark | 164.9 (68.9-328.4) | 2.7 (1.1-5.3) | 178.5 (74.4-357.2) | 2.4 (1-4.8) | -1.29 (-2.67-0.11) |
| Genital herpes | Djibouti | 18.6 (8.2-37.3) | 6.1 (2.6-12.2) | 71.9 (30.9-145.9) | 6.1 (2.6-12.4) | 1.17 (-2.08-4.54) |
| Genital herpes | Dominica | 3.3 (1.4-6.6) | 5.5 (2.3-10.7) | 4.1 (1.7-8.3) | 5.4 (2.3-10.9) | -0.12 (-2.68-2.5) |
| Genital herpes | Dominican Republic | 303.5 (131.3-617.8) | 5.4 (2.3-10.9) | 606.9 (257.4-1234.2) | 5.4 (2.3-11) | -0.21 (-2.89-2.53) |
| Genital herpes | Ecuador | 467.8 (203.4-948.1) | 5.9 (2.6-11.8) | 1067 (460.1-2127.7) | 5.9 (2.6-11.8) | 0.41 (-2.02-2.91) |
| Genital herpes | Egypt | 1060.6 (458.8-2106.3) | 2.5 (1.1-5) | 2307.9 (992.8-4587.3) | 2.5 (1.1-4.9) | -0.62 (-2.09-0.86) |
| Genital herpes | El Salvador | 223.3 (96.9-450.6) | 5.6 (2.4-11.3) | 363.2 (155.2-732.4) | 5.7 (2.4-11.5) | -0.37 (-2.5-1.8) |
| Genital herpes | Equatorial Guinea | 23.8 (10.6-46.6) | 8 (3.5-15.8) | 92.1 (40.3-180.6) | 7.8 (3.4-15.5) | -0.02 (-3.5-3.59) |
| Genital herpes | Eritrea | 130 (57.4-255.7) | 5.6 (2.4-11.1) | 310.7 (132.2-623.1) | 5.8 (2.4-11.6) | -1.11 (-3.94-1.81) |
| Genital herpes | Estonia | 55.7 (23.2-113) | 3.1 (1.3-6.4) | 51.4 (21.8-102.2) | 3.1 (1.3-6.1) | -1.5 (-3.11-0.14) |
| Genital herpes | Ethiopia | 1677.4 (726.5-3383.8) | 5.1 (2.2-10.2) | 4082.7 (1767.6-8221.9) | 5.1 (2.2-10.2) | -2.58 (-5.44-0.36) |
| Genital herpes | Federated States of Micronesia | 3.1 (1.3-6.1) | 4.3 (1.8-8.5) | 4.2 (1.8-8.4) | 4.3 (1.8-8.7) | 1.06 (-1.48-3.66) |
| Genital herpes | Fiji | 26.7 (11.3-53.9) | 4.3 (1.8-8.5) | 39.7 (16.8-80.4) | 4.3 (1.8-8.7) | -0.09 (-2.68-2.57) |
| Genital herpes | Finland | 167 (72.1-338.3) | 2.8 (1.2-5.6) | 151.8 (63.4-303.6) | 2.1 (0.9-4.3) | -1.21 (-2.17--0.24) |
| Genital herpes | France | 1456.2 (611.1-2981.9) | 2.2 (0.9-4.5) | 1811.8 (751.9-3596.2) | 2.2 (0.9-4.4) | -1.25 (-3.08-0.62) |
| Genital herpes | Gabon | 58.8 (26-118.2) | 7.8 (3.4-15.5) | 127.8 (55.1-253.4) | 8 (3.4-15.7) | -1.25 (-4.56-2.18) |
| Genital herpes | Georgia | 128.1 (53.5-249.7) | 2.2 (0.9-4.2) | 95.6 (39.5-191.3) | 2.1 (0.9-4.3) | -0.74 (-1.97-0.52) |
| Genital herpes | Germany | 3202.3 (1343.5-6576.1) | 3.2 (1.4-6.7) | 2963 (1235.3-5836.7) | 2.6 (1.1-5.3) | -1.05 (-2.57-0.48) |
| Genital herpes | Ghana | 550.7 (231.3-1102) | 5.2 (2.2-10.5) | 1500.5 (656.9-3037.8) | 5.3 (2.3-10.8) | -0.3 (-3.46-2.95) |
| Genital herpes | Greece | 224 (94.4-441.5) | 1.8 (0.8-3.7) | 250.8 (104.1-510.8) | 1.8 (0.8-3.7) | -0.26 (-1.4-0.9) |
| Genital herpes | Greenland | 1.6 (0.7-3.2) | 2.8 (1.2-5.5) | 1.8 (0.8-3.6) | 2.8 (1.2-5.6) | -0.61 (-2.62-1.43) |
| Genital herpes | Grenada | 3.8 (1.6-7.6) | 5.5 (2.3-10.9) | 6.1 (2.6-12.3) | 5.4 (2.3-10.8) | -0.33 (-2.33-1.71) |
| Genital herpes | Guam | 5.5 (2.3-11) | 4.3 (1.8-8.6) | 7.7 (3.2-15.3) | 4.3 (1.9-8.7) | 0.1 (-2.43-2.69) |
| Genital herpes | Guatemala | 303.8 (133.8-611.8) | 5.5 (2.4-11.1) | 797.6 (351.1-1598.4) | 5.6 (2.5-11.2) | -0.57 (-2.56-1.47) |
| Genital herpes | Guinea | 225.5 (95.9-453) | 5.2 (2.2-10.4) | 501.1 (216-1026.5) | 5.3 (2.2-11) | -0.39 (-3.49-2.82) |
| Genital herpes | Guinea-Bissau | 34.7 (15.2-69.8) | 5.3 (2.2-10.7) | 79.5 (34.7-160.1) | 5.4 (2.3-10.8) | 0.08 (-2.95-3.2) |
| Genital herpes | Guyana | 32.7 (14-66.3) | 5.4 (2.3-10.7) | 41.3 (17.7-81.6) | 5.5 (2.3-10.8) | -0.39 (-2.96-2.25) |
| Genital herpes | Haiti | 227.7 (95-452.9) | 4.8 (2-9.5) | 545.7 (234.1-1091.7) | 4.8 (2-9.5) | -0.93 (-4.4-2.66) |
| Genital herpes | Honduras | 215.9 (93.2-428.4) | 6.8 (2.9-13.5) | 620.2 (267-1251.1) | 6.9 (2.9-13.6) | -1.22 (-3.01-0.6) |
| Genital herpes | Hungary | 154.7 (65.3-305.8) | 1.2 (0.5-2.5) | 163.1 (67.5-325.1) | 1.2 (0.5-2.5) | -0.77 (-2.34-0.81) |
| Genital herpes | Iceland | 6.2 (2.6-12.8) | 2.4 (1-4.8) | 9.8 (4.1-19.8) | 2.3 (1-4.8) | -0.57 (-1.89-0.78) |
| Genital herpes | India | 11568.5 (4922.4-23190) | 1.8 (0.8-3.5) | 27368.3 (11577.5-54826.9) | 1.9 (0.8-3.9) | -0.13 (-2.5-2.3) |
| Genital herpes | Indonesia | 4836.1 (2030-9864.5) | 3.2 (1.3-6.5) | 9816.3 (4106.3-19714.6) | 3.2 (1.4-6.5) | 0.86 (-1.43-3.21) |
| Genital herpes | Iran | 1178.1 (503.5-2382.8) | 3 (1.3-6.1) | 2939.4 (1236.8-5883) | 3 (1.3-6.1) | 0.76 (-0.72-2.25) |
| Genital herpes | Iraq | 306.6 (132.7-614.2) | 2.5 (1-5) | 902.1 (396.3-1831.3) | 2.5 (1.1-5) | 0.3 (-1.27-1.89) |
| Genital herpes | Ireland | 78.9 (33.3-158.9) | 2.2 (0.9-4.4) | 129.1 (53.8-257.8) | 2.2 (0.9-4.4) | -0.74 (-2-0.54) |
| Genital herpes | Israel | 98.7 (41.7-195.7) | 2.1 (0.9-4.2) | 203.6 (85.7-411.8) | 2 (0.9-4.1) | -0.75 (-2.19-0.71) |
| Genital herpes | Italy | 1322.2 (552-2620.2) | 1.9 (0.8-3.8) | 1580.8 (652.4-3136) | 1.9 (0.8-3.8) | -1 (-2.77-0.8) |
| Genital herpes | Jamaica | 138.9 (61.5-283.2) | 7 (3-14.2) | 191.8 (80.3-379.3) | 6.2 (2.6-12.2) | -0.52 (-2.87-1.9) |
| Genital herpes | Japan | 2596.7 (1081.2-5203.3) | 1.7 (0.7-3.4) | 3133.1 (1295.1-6341.2) | 1.7 (0.7-3.5) | 0.26 (-0.63-1.16) |
| Genital herpes | Jordan | 69.6 (30.2-138.8) | 2.8 (1.2-5.7) | 323.6 (137.3-657.9) | 2.8 (1.2-5.6) | 0.37 (-1.37-2.14) |
| Genital herpes | Kazakhstan | 320.8 (135.5-644.2) | 2.1 (0.9-4.3) | 425 (175.3-867.3) | 2.1 (0.9-4.4) | -0.99 (-2.48-0.52) |
| Genital herpes | Kenya | 1018.3 (446.3-2023.8) | 6.9 (2.9-13.7) | 2813.3 (1223.4-5680.1) | 6.9 (2.9-13.8) | -1.74 (-5.24-1.9) |
| Genital herpes | Kiribati | 2.5 (1-4.9) | 4.3 (1.8-8.5) | 4.6 (1.9-9.2) | 4.3 (1.8-8.5) | -0.21 (-2.84-2.5) |
| Genital herpes | Kuwait | 35.6 (15.6-71.7) | 2.3 (1-4.7) | 139.6 (59-279.8) | 2.4 (1-4.9) | 0.22 (-0.7-1.15) |
| Genital herpes | Kyrgyzstan | 75.2 (30.8-151) | 2.1 (0.9-4.3) | 133.9 (57.1-271.9) | 2.1 (0.9-4.3) | -1.59 (-3.22-0.08) |
| Genital herpes | Laos | 110.3 (47.1-220) | 3.7 (1.6-7.4) | 253.1 (106.1-516.2) | 3.7 (1.5-7.5) | 2.19 (0.18-4.25) |
| Genital herpes | Latvia | 95.6 (39.6-194.3) | 3.1 (1.3-6.4) | 74.6 (30.9-149) | 3.1 (1.3-6.3) | -1.73 (-3.64-0.23) |
| Genital herpes | Lebanon | 65.9 (28.3-134.4) | 2.5 (1.1-5.2) | 153.3 (64.7-306.1) | 2.5 (1.1-5) | -0.08 (-1.82-1.69) |
| Genital herpes | Lesotho | 83.2 (36.2-167.7) | 7.4 (3.2-14.9) | 130.9 (56.5-265.3) | 8 (3.4-16) | 0.22 (-3.54-4.13) |
| Genital herpes | Liberia | 86.7 (37.6-172.9) | 5.1 (2.1-10.2) | 216.6 (94.2-435.1) | 5.1 (2.2-10.1) | 0.18 (-3.18-3.66) |
| Genital herpes | Libya | 71.4 (30.4-143.6) | 2.4 (1-5) | 189 (80.7-377.8) | 2.5 (1.1-5) | 0.28 (-1.35-1.94) |
| Genital herpes | Lithuania | 127.3 (53.2-255.7) | 3.1 (1.3-6.3) | 109.3 (44.7-218.6) | 3.1 (1.3-6.3) | -0.28 (-1.96-1.42) |
| Genital herpes | Luxembourg | 10.1 (4.2-20.3) | 2.2 (0.9-4.4) | 17.9 (7.4-35.8) | 2.2 (0.9-4.3) | -1.35 (-2.76-0.09) |
| Genital herpes | Macedonia | 32.6 (13.6-65) | 1.6 (0.7-3.2) | 45.3 (19-90.2) | 1.6 (0.7-3.1) | -0.35 (-1.35-0.66) |
| Genital herpes | Madagascar | 564.5 (246.1-1135.3) | 6.8 (2.9-13.7) | 1508.9 (663.4-2998.5) | 6.9 (2.9-13.7) | 1.41 (-1.31-4.21) |
| Genital herpes | Malawi | 589.5 (260-1165.1) | 8.5 (3.7-16.7) | 1187.1 (515.2-2388.7) | 8.2 (3.5-16.3) | -1.51 (-5.09-2.2) |
| Genital herpes | Malaysia | 531.9 (224.6-1087.2) | 3.7 (1.5-7.5) | 1241.3 (521-2522.2) | 3.7 (1.5-7.5) | -0.02 (-1.99-1.99) |
| Genital herpes | Maldives | 5.3 (2.3-10.5) | 3.6 (1.6-7.3) | 21.6 (8.9-43) | 3.5 (1.5-7.1) | 0.19 (-1.28-1.68) |
| Genital herpes | Mali | 414.9 (186.6-813.5) | 7 (3-13.7) | 922.1 (401.7-1856) | 5.9 (2.5-11.8) | -0.61 (-3.63-2.51) |
| Genital herpes | Malta | 9 (3.8-18.1) | 2.2 (0.9-4.4) | 12.6 (5.2-25.2) | 2.2 (0.9-4.3) | -0.46 (-1.82-0.92) |
| Genital herpes | Marshall Islands | 1.2 (0.5-2.5) | 4.3 (1.8-8.6) | 2.3 (1-4.5) | 4.3 (1.8-8.5) | 1.27 (-1.19-3.79) |
| Genital herpes | Mauritania | 75.1 (32-151.2) | 5.2 (2.2-10.5) | 171.3 (73.2-342.8) | 5.3 (2.2-10.5) | -0.99 (-3.24-1.32) |
| Genital herpes | Mauritius | 37.7 (16.1-77.4) | 3.7 (1.6-7.5) | 57.7 (24.3-116) | 3.7 (1.6-7.4) | 2.09 (0.34-3.86) |
| Genital herpes | Mexico | 2649.4 (1138.7-5320.7) | 4.1 (1.7-8.3) | 5636.6 (2354-11251.4) | 4.1 (1.7-8.2) | -0.16 (-2.13-1.85) |
| Genital herpes | Moldova | 144.1 (60.6-294.1) | 3.1 (1.3-6.4) | 145.9 (61-289.5) | 3.1 (1.3-6.2) | -0.76 (-2.62-1.14) |
| Genital herpes | Monaco | 0.9 (0.4-1.8) | 2.2 (0.9-4.4) | 1.1 (0.5-2.2) | 2.2 (0.9-4.4) | -0.69 (-1.99-0.62) |
| Genital herpes | Mongolia | 31.1 (13.2-61.4) | 2.1 (0.9-4.1) | 68.8 (29.4-139.3) | 2.1 (0.9-4.3) | 0.06 (-2.01-2.17) |
| Genital herpes | Montenegro | 10.4 (4.3-21) | 1.6 (0.7-3.2) | 12.4 (5.2-25) | 1.6 (0.7-3.2) | -0.19 (-1.37-1.01) |
| Genital herpes | Morocco | 733.3 (326.5-1498.1) | 3.6 (1.6-7.3) | 1064.2 (456.3-2164.7) | 2.7 (1.2-5.6) | 0.05 (-2.75-2.94) |
| Genital herpes | Mozambique | 715.8 (318.5-1434.1) | 7.5 (3.2-15) | 1704.6 (733.9-3402.7) | 7.8 (3.4-15.6) | -0.91 (-4.37-2.67) |
| Genital herpes | Myanmar | 1214.2 (519.9-2456.8) | 3.7 (1.6-7.5) | 2109 (920.4-4256.5) | 3.7 (1.6-7.5) | 0.78 (-1.77-3.39) |
| Genital herpes | Namibia | 72.9 (31.3-146.1) | 7.2 (3-14.2) | 165.5 (70.1-337.3) | 7.6 (3.2-15.6) | -0.65 (-4.11-2.94) |
| Genital herpes | Nauru | 0.3 (0.1-0.7) | 4.3 (1.8-8.6) | 0.4 (0.2-0.8) | 4.3 (1.8-8.7) | 1.14 (-1.44-3.79) |
| Genital herpes | Nepal | 234.2 (99.2-470.8) | 1.7 (0.7-3.5) | 494.4 (209.5-1018.1) | 1.7 (0.7-3.6) | 1.4 (-1.18-4.06) |
| Genital herpes | Netherlands | 470.7 (194.4-961.5) | 2.7 (1.1-5.5) | 588 (245.6-1191) | 2.7 (1.1-5.4) | -1.07 (-2.58-0.46) |
| Genital herpes | New Zealand | 65.5 (27.4-131.4) | 1.8 (0.7-3.6) | 112.2 (47-226.5) | 1.8 (0.8-3.7) | -0.6 (-1.83-0.66) |
| Genital herpes | Nicaragua | 142.5 (61.6-287.1) | 5.6 (2.4-11.5) | 356.3 (153.1-707.3) | 5.6 (2.4-11.2) | -0.57 (-2.42-1.33) |
| Genital herpes | Niger | 259.4 (112.9-517.7) | 5.1 (2.2-10.4) | 776.1 (333.3-1530.3) | 5.2 (2.2-10.6) | -1.35 (-3.77-1.12) |
| Genital herpes | Nigeria | 3333.1 (1435.7-6697.4) | 5.1 (2.2-10.2) | 8854 (3794.3-17796.5) | 5.5 (2.3-10.8) | -0.33 (-3.34-2.76) |
| Genital herpes | Niue | 0.1 (0-0.2) | 4.3 (1.8-8.6) | 0.1 (0-0.2) | 4.3 (1.8-8.5) | 0.93 (-1.36-3.28) |
| Genital herpes | North Korea | 488.1 (207.5-986.8) | 2.5 (1-4.9) | 771.1 (323.8-1555.8) | 2.4 (1-4.8) | 1.29 (-0.41-3.01) |
| Genital herpes | Northern Mariana Islands | 1.9 (0.8-3.9) | 4.3 (1.8-8.7) | 2.4 (1-4.8) | 4.3 (1.8-8.7) | -0.16 (-2.48-2.21) |
| Genital herpes | Norway | 112.8 (46.8-224.1) | 2.3 (0.9-4.5) | 142.2 (59.1-283) | 2.1 (0.9-4.2) | -0.07 (-1.31-1.18) |
| Genital herpes | Oman | 33.4 (14-68) | 2.3 (1-4.7) | 111.5 (47.2-224.7) | 2.3 (1-4.6) | 0.1 (-1.3-1.52) |
| Genital herpes | Pakistan | 1050.7 (450.7-2091.7) | 1.4 (0.6-2.8) | 2685.3 (1140.8-5484.8) | 1.4 (0.6-2.9) | 2.13 (-0.38-4.71) |
| Genital herpes | Palau | 0.6 (0.3-1.2) | 4.3 (1.8-8.6) | 1 (0.4-2) | 4.3 (1.8-8.7) | 0.87 (-1.2-2.98) |
| Genital herpes | Palestine | 32.6 (14.5-65.6) | 2.5 (1.1-5.2) | 104.3 (45.6-208.3) | 2.5 (1.1-5.1) | 0.18 (-1.25-1.62) |
| Genital herpes | Panama | 111.1 (47.5-226) | 5.5 (2.3-11.3) | 243.9 (104.5-487.2) | 5.5 (2.4-11.1) | -0.19 (-2.52-2.2) |
| Genital herpes | Papua New Guinea | 144.9 (61-291.9) | 4.7 (2-9.3) | 414.6 (178.4-830.7) | 4.7 (2-9.4) | 0.95 (-1.92-3.91) |
| Genital herpes | Paraguay | 195.6 (85.2-401.5) | 6.4 (2.7-13.1) | 450.2 (192.4-894.1) | 6.4 (2.7-12.6) | 0.55 (-2.01-3.19) |
| Genital herpes | Peru | 1035.3 (459.8-2052.1) | 5.9 (2.6-11.8) | 2221 (948.9-4451) | 5.9 (2.5-11.8) | -1.14 (-3.45-1.23) |
| Genital herpes | Philippines | 1546.4 (657.4-3141.5) | 3.3 (1.4-6.6) | 3563.5 (1504.2-7191.3) | 3.3 (1.4-6.7) | 2.65 (0.47-4.88) |
| Genital herpes | Poland | 564.8 (234.4-1146.3) | 1.3 (0.6-2.7) | 706 (293.9-1418.8) | 1.4 (0.6-2.7) | 0.44 (-0.67-1.57) |
| Genital herpes | Portugal | 251.7 (107.2-506.5) | 2.2 (0.9-4.4) | 313.4 (131.6-630.3) | 2.2 (0.9-4.5) | -0.97 (-3.21-1.33) |
| Genital herpes | Puerto Rico | 198.2 (84.7-389.4) | 5.5 (2.3-10.8) | 230.3 (95.2-448.7) | 5.5 (2.3-10.9) | -1.18 (-3.47-1.17) |
| Genital herpes | Qatar | 9.2 (3.9-19.1) | 2.2 (0.9-4.4) | 77.2 (32.6-155.4) | 2.2 (0.9-4.3) | -0.84 (-2.44-0.79) |
| Genital herpes | Romania | 411.2 (172.1-826.9) | 1.6 (0.7-3.2) | 404.1 (169.3-807.1) | 1.6 (0.7-3.2) | -0.64 (-2.33-1.08) |
| Genital herpes | Russian Federation | 5070.6 (2103.9-10057.1) | 3 (1.2-6) | 5461.5 (2246.9-10734.7) | 3 (1.2-5.9) | -0.01 (-2.12-2.15) |
| Genital herpes | Rwanda | 321.7 (140.6-642.2) | 6.6 (2.8-13.2) | 732.9 (317.5-1464.5) | 6.7 (2.8-13.4) | -2.13 (-5.1-0.94) |
| Genital herpes | Saint Kitts and Nevis | 1.9 (0.8-3.8) | 5.4 (2.3-10.8) | 3.9 (1.6-7.8) | 5.4 (2.3-10.8) | -0.04 (-2.46-2.43) |
| Genital herpes | Saint Lucia | 5.8 (2.5-11.9) | 5.5 (2.3-11.2) | 11.8 (4.9-23.5) | 5.4 (2.3-10.8) | -0.33 (-2.21-1.58) |
| Genital herpes | Saint Vincent and the Grenadines | 4.6 (1.9-9.2) | 5.4 (2.2-10.8) | 6.9 (2.9-13.8) | 5.4 (2.3-10.8) | -0.95 (-3.39-1.54) |
| Genital herpes | Samoa | 5.2 (2.3-10.4) | 4.3 (1.8-8.6) | 7.7 (3.2-15.3) | 4.3 (1.8-8.6) | 1 (-0.74-2.77) |
| Genital herpes | San Marino | 0.6 (0.3-1.2) | 2.2 (0.9-4.3) | 1 (0.4-1.9) | 2.2 (0.9-4.4) | -0.63 (-1.96-0.72) |
| Genital herpes | Sao Tome and Principe | 4.2 (1.9-8.6) | 5.3 (2.3-10.8) | 9.5 (4-19) | 5.2 (2.2-10.4) | -0.96 (-2.73-0.83) |
| Genital herpes | Saudi Arabia | 270.5 (119.2-552.8) | 2.3 (1-4.8) | 1009.7 (431.4-2021.3) | 2.3 (1-4.8) | -0.08 (-1.72-1.6) |
| Genital herpes | Senegal | 227.4 (96.6-462.9) | 4.6 (1.9-9.2) | 549.8 (237.2-1109.1) | 4.6 (1.9-9.2) | -1.3 (-3.87-1.34) |
| Genital herpes | Serbia | 175 (74.3-351.9) | 1.6 (0.7-3.2) | 186.7 (78.5-375.3) | 1.6 (0.7-3.2) | -0.21 (-1.49-1.08) |
| Genital herpes | Seychelles | 2.3 (1-4.8) | 3.7 (1.6-7.4) | 4.4 (1.8-8.9) | 3.6 (1.5-7.4) | 0.46 (-1.38-2.33) |
| Genital herpes | Sierra Leone | 157.2 (67.2-319) | 5.2 (2.2-10.4) | 350 (150.6-705) | 5.2 (2.2-10.5) | -0.02 (-2.85-2.89) |
| Genital herpes | Singapore | 74.4 (31.3-149.2) | 2.3 (1-4.7) | 178.7 (73-355.5) | 2.3 (1-4.6) | -0.6 (-1.88-0.7) |
| Genital herpes | Slovakia | 90.5 (38.1-181.4) | 1.6 (0.7-3.2) | 115.2 (48.2-233.3) | 1.6 (0.7-3.2) | -0.14 (-1-0.73) |
| Genital herpes | Slovenia | 36.4 (15.5-72.9) | 1.6 (0.7-3.2) | 44.8 (18.4-88.3) | 1.6 (0.7-3.1) | -0.55 (-1.52-0.43) |
| Genital herpes | Solomon Islands | 9.7 (4.1-19.6) | 4.3 (1.8-8.6) | 24.1 (10.4-48.8) | 4.3 (1.8-8.8) | 0.76 (-1.95-3.55) |
| Genital herpes | Somalia | 357.6 (155.6-710.2) | 6.8 (2.9-13.4) | 973.6 (430.7-1971.5) | 6.8 (2.9-13.8) | 0.62 (-2.04-3.36) |
| Genital herpes | South Africa | 2145.2 (909.2-4268.8) | 7.1 (3-13.9) | 4388.6 (1831.7-8672.7) | 7.4 (3.1-14.6) | -0.51 (-3.9-3.01) |
| Genital herpes | South Korea | 2074.1 (927.3-4232.7) | 4.7 (2.1-9.7) | 2082.1 (858.5-4152.8) | 2.9 (1.2-5.8) | -0.2 (-1.6-1.21) |
| Genital herpes | South Sudan | 277.4 (119.8-554.1) | 6.7 (2.9-13.4) | 479.1 (209.1-958.5) | 6.9 (2.9-13.8) | 0.59 (-2.73-4.02) |
| Genital herpes | Spain | 809.7 (349.3-1623.7) | 1.8 (0.8-3.7) | 1184.7 (489-2324.7) | 1.9 (0.8-3.9) | -1.37 (-3.39-0.69) |
| Genital herpes | Sri Lanka | 566 (239.8-1138.5) | 3.7 (1.5-7.4) | 915 (381.4-1831.3) | 3.7 (1.6-7.5) | -0.03 (-1.25-1.21) |
| Genital herpes | Sudan | 347.2 (153.6-700.9) | 2.5 (1.1-5) | 849.3 (374.7-1689.7) | 2.5 (1.1-5) | 0.45 (-2.48-3.47) |
| Genital herpes | Suriname | 17.8 (7.7-35.4) | 5.4 (2.3-10.8) | 34.1 (14-69.6) | 5.5 (2.3-11.1) | -0.25 (-2.76-2.33) |
| Genital herpes | Swaziland | 38.3 (16.6-76.6) | 7.3 (3.1-14.5) | 79 (33.8-159.2) | 8 (3.4-15.9) | 0.17 (-3.5-3.98) |
| Genital herpes | Sweden | 278 (115.4-560) | 2.7 (1.1-5.5) | 334.6 (139.3-667.5) | 2.6 (1.1-5.2) | -0.61 (-1.86-0.66) |
| Genital herpes | Switzerland | 223.5 (97.2-452.9) | 2.7 (1.2-5.4) | 277.9 (117.1-562.2) | 2.4 (1-4.8) | -1.31 (-2.83-0.24) |
| Genital herpes | Syria | 167.8 (72.4-344) | 2.1 (0.9-4.2) | 307.1 (131-619.4) | 2.1 (0.9-4.3) | -0.23 (-1.72-1.29) |
| Genital herpes | Taiwan | 472.9 (198.2-952.2) | 2.4 (1-4.7) | 781.3 (320.6-1602.5) | 2.4 (1-4.9) | 0.52 (-0.69-1.74) |
| Genital herpes | Tajikistan | 76.7 (32.3-151.5) | 2.1 (0.9-4.2) | 184 (77.7-377.5) | 2.1 (0.9-4.3) | -0.83 (-2.36-0.73) |
| Genital herpes | Tanzania | 1199.7 (537.4-2389.1) | 6.7 (2.9-13.7) | 2868 (1275.4-5747.5) | 6.5 (2.8-13.1) | -1.93 (-5.41-1.68) |
| Genital herpes | Thailand | 2343.5 (1057.8-4698.8) | 4.4 (2-8.9) | 3470.2 (1448.1-6931) | 4 (1.7-8.2) | -1.84 (-4.44-0.83) |
| Genital herpes | The Bahamas | 12.5 (5.3-25.3) | 5.5 (2.3-11.1) | 24.2 (10.1-47.7) | 5.5 (2.3-10.9) | -0.2 (-3-2.68) |
| Genital herpes | The Gambia | 29.5 (13-59.9) | 4.5 (1.9-9.2) | 82 (36-163.6) | 4.7 (2-9.4) | 0.52 (-2.59-3.73) |
| Genital herpes | Timor-Leste | 23.2 (10-46.8) | 4.1 (1.7-8.2) | 44.7 (19.4-90.3) | 4.1 (1.7-8.3) | 0.7 (-1.77-3.23) |
| Genital herpes | Togo | 123.2 (53.1-244.4) | 5.3 (2.2-10.5) | 355 (151.3-707.9) | 5.4 (2.2-10.9) | -1.34 (-4.49-1.91) |
| Genital herpes | Tokelau | 0.1 (0-0.1) | 4.3 (1.8-8.5) | 0.1 (0-0.1) | 4.3 (1.8-8.8) | 0.88 (-1.44-3.25) |
| Genital herpes | Tonga | 3.1 (1.3-6.2) | 4.3 (1.8-8.6) | 3.9 (1.6-7.8) | 4.3 (1.8-8.7) | -0.18 (-2.35-2.03) |
| Genital herpes | Trinidad and Tobago | 57.5 (24.7-116) | 5.4 (2.3-10.7) | 90.4 (37.8-181) | 5.4 (2.3-11) | -0.61 (-3.06-1.9) |
| Genital herpes | Tunisia | 167.9 (72.5-337) | 2.5 (1.1-5.1) | 337.6 (144-680.3) | 2.5 (1.1-5.1) | 0.49 (-1-2) |
| Genital herpes | Turkey | 1039.3 (447.6-2088) | 2.2 (0.9-4.4) | 2027.5 (861.6-4007.4) | 2.1 (0.9-4.2) | 0.12 (-1.61-1.89) |
| Genital herpes | Turkmenistan | 56.5 (23.8-114.3) | 2.1 (0.9-4.3) | 105.7 (44-212.4) | 2.1 (0.9-4.3) | -1.44 (-3.19-0.34) |
| Genital herpes | Tuvalu | 0.4 (0.1-0.7) | 4.3 (1.8-8.6) | 0.5 (0.2-1) | 4.3 (1.8-8.6) | 0.58 (-1.59-2.81) |
| Genital herpes | Uganda | 1293.7 (581.3-2587.8) | 11.1 (4.8-22.6) | 2385.3 (1069.6-4807.4) | 8 (3.4-15.8) | -0.91 (-4.47-2.78) |
| Genital herpes | Ukraine | 1821.2 (758.1-3625.6) | 3 (1.3-6) | 1704 (709.9-3378) | 3 (1.2-5.9) | -0.04 (-2.37-2.33) |
| Genital herpes | United Arab Emirates | 36.8 (15.8-74.3) | 2.2 (0.9-4.5) | 281.5 (119.6-567.9) | 2.1 (0.9-4.3) | -0.05 (-1.32-1.24) |
| Genital herpes | United Kingdom | 1277.7 (535.8-2554.8) | 1.9 (0.8-3.8) | 1624.3 (677.4-3208) | 1.9 (0.8-3.8) | -0.05 (-1.53-1.45) |
| Genital herpes | United States | 11965.8 (4990-23899.9) | 4.2 (1.8-8.4) | 14424.6 (6023.2-28548.4) | 3.6 (1.5-7.2) | -1.38 (-3.39-0.67) |
| Genital herpes | Uruguay | 136 (58-268.2) | 4.1 (1.8-8.2) | 166.1 (69.4-327.3) | 4.2 (1.8-8.3) | -0.43 (-2.37-1.55) |
| Genital herpes | Uzbekistan | 317.6 (133.1-632.3) | 2.1 (0.9-4.2) | 725 (306.4-1427.9) | 2.1 (0.9-4.2) | -0.92 (-2.4-0.58) |
| Genital herpes | Vanuatu | 4.2 (1.8-8.5) | 3.9 (1.6-7.9) | 10.2 (4.4-20.4) | 3.9 (1.6-7.9) | 1.02 (-1.61-3.72) |
| Genital herpes | Venezuela | 839.9 (362.7-1703.7) | 5.6 (2.4-11.2) | 1644.4 (688-3242.9) | 5.6 (2.4-11.2) | -0.11 (-2.12-1.94) |
| Genital herpes | Vietnam | 1873.2 (803.2-3662.2) | 3.6 (1.5-7) | 3960.1 (1636.3-7991.8) | 3.6 (1.5-7.2) | 0.36 (-1.83-2.61) |
| Genital herpes | Yemen | 203.1 (87.8-410.7) | 2.5 (1.1-5.1) | 627.4 (270.7-1266.3) | 2.5 (1.1-5) | 0.77 (-0.94-2.51) |
| Genital herpes | Zambia | 413.3 (182.3-825.6) | 7.8 (3.4-15.8) | 1185.3 (510.1-2362.9) | 8 (3.5-16) | -1.45 (-5.04-2.27) |
| Genital herpes | Zimbabwe | 527.6 (230.2-1044.2) | 7.7 (3.2-15.5) | 989.4 (423.6-1970.6) | 8.1 (3.4-16.1) | -0.98 (-4.66-2.84) |
| Gonococcal infection | Afghanistan | 63.2 (34.3-112.5) | 0.7 (0.4-1.2) | 233.7 (126.3-386.7) | 0.8 (0.4-1.3) | 0.27 (-1.62-2.2) |
| Gonococcal infection | Albania | 20.9 (13.2-32.2) | 0.6 (0.4-0.9) | 12.6 (7.6-19.8) | 0.5 (0.3-0.8) | -0.44 (-1.52-0.65) |
| Gonococcal infection | Algeria | 165.2 (87.9-291.7) | 0.7 (0.4-1.1) | 274.7 (158.1-441.1) | 0.6 (0.4-1) | -0.05 (-1.71-1.63) |
| Gonococcal infection | American Samoa | 0.3 (0.1-0.4) | 0.5 (0.3-0.9) | 0.2 (0.1-0.4) | 0.5 (0.3-0.8) | 0.22 (-2.25-2.76) |
| Gonococcal infection | Andorra | 0.1 (0.1-0.1) | 0.1 (0.1-0.2) | 0.1 (0.1-0.2) | 0.1 (0.1-0.2) | -0.81 (-2.23-0.63) |
| Gonococcal infection | Angola | 221 (134.3-321) | 2.8 (1.6-4.3) | 475.2 (301.9-741.7) | 1.8 (1.1-2.7) | 0.25 (-2.86-3.45) |
| Gonococcal infection | Antigua and Barbuda | 0.7 (0.4-1) | 1 (0.7-1.5) | 0.8 (0.6-1.2) | 0.8 (0.6-1.3) | -0.3 (-2.48-1.93) |
| Gonococcal infection | Argentina | 299.4 (215.4-414.2) | 0.9 (0.7-1.3) | 389.2 (278.3-565.8) | 0.8 (0.6-1.2) | -0.36 (-2.29-1.61) |
| Gonococcal infection | Armenia | 74.3 (53.7-104.2) | 2.1 (1.5-2.9) | 32.3 (20.1-48.5) | 1.2 (0.7-1.8) | -1.22 (-2.26--0.18) |
| Gonococcal infection | Australia | 151.5 (94.8-226.7) | 0.8 (0.5-1.2) | 166.1 (98.7-255) | 0.6 (0.4-1) | -1.33 (-2.67-0.02) |
| Gonococcal infection | Austria | 13.7 (10.6-18.3) | 0.2 (0.1-0.2) | 8.7 (6.2-12.7) | 0.1 (0.1-0.1) | -0.88 (-2.29-0.55) |
| Gonococcal infection | Azerbaijan | 192.7 (136.1-277.4) | 2.5 (1.8-3.5) | 177.4 (114.3-262.9) | 1.6 (1-2.5) | -1.22 (-2.53-0.1) |
| Gonococcal infection | Bahrain | 4.2 (2.2-7.2) | 0.7 (0.4-1.2) | 13.3 (7.7-21.6) | 0.8 (0.4-1.2) | -0.19 (-2.07-1.73) |
| Gonococcal infection | Bangladesh | 1462.2 (943.8-2182.6) | 1.9 (1.3-2.9) | 1396.2 (955.2-2121) | 0.9 (0.6-1.3) | 0.15 (-2.13-2.49) |
| Gonococcal infection | Barbados | 3.1 (2.1-4.6) | 1.1 (0.8-1.7) | 2.7 (1.8-4) | 1 (0.6-1.4) | -0.79 (-3-1.47) |
| Gonococcal infection | Belarus | 89.3 (66.3-123.6) | 0.8 (0.6-1.2) | 52.9 (38.5-73) | 0.6 (0.4-0.8) | -0.45 (-2.15-1.29) |
| Gonococcal infection | Belgium | 20.8 (16.4-27.2) | 0.2 (0.1-0.2) | 12.4 (9.2-17.4) | 0.1 (0.1-0.1) | -0.7 (-2.22-0.85) |
| Gonococcal infection | Belize | 1.2 (0.7-2.1) | 0.7 (0.4-1.1) | 3.3 (2-5.2) | 0.7 (0.4-1.1) | -0.05 (-2.66-2.64) |
| Gonococcal infection | Benin | 78.6 (47.5-125.5) | 2.1 (1.2-3.7) | 186 (120.2-294.2) | 1.6 (1-2.5) | -0.41 (-3.25-2.52) |
| Gonococcal infection | Bermuda | 0.5 (0.3-0.8) | 0.8 (0.5-1.2) | 0.3 (0.2-0.5) | 0.6 (0.4-1) | -0.54 (-2.84-1.81) |
| Gonococcal infection | Bhutan | 7.5 (4.7-10.8) | 1.4 (0.9-2) | 7.5 (5.2-11.3) | 0.9 (0.6-1.3) | -0.07 (-2.86-2.8) |
| Gonococcal infection | Bolivia | 46.6 (32.6-67.1) | 0.9 (0.6-1.2) | 72 (49.9-102) | 0.6 (0.4-0.8) | 0.06 (-2.78-2.98) |
| Gonococcal infection | Bosnia and Herzegovina | 24 (14.8-39) | 0.5 (0.3-0.8) | 13 (8.1-19.5) | 0.4 (0.3-0.7) | -0.03 (-1.08-1.04) |
| Gonococcal infection | Botswana | 30.3 (19.8-44.9) | 2.8 (1.7-4.2) | 45.5 (29.7-69.7) | 1.7 (1.1-2.7) | -1.74 (-5.42-2.07) |
| Gonococcal infection | Brazil | 1039.6 (777.8-1418.8) | 0.7 (0.5-0.9) | 1891.7 (1420.9-2560.6) | 0.8 (0.6-1.1) | -0.03 (-2.1-2.08) |
| Gonococcal infection | Brunei | 2 (1.3-3.2) | 0.8 (0.5-1.1) | 3.4 (2.2-5.3) | 0.6 (0.4-1) | 0.3 (-1.23-1.84) |
| Gonococcal infection | Bulgaria | 40.5 (25.8-60.9) | 0.5 (0.3-0.7) | 24.9 (15.7-38.5) | 0.4 (0.3-0.7) | -0.32 (-1.9-1.28) |
| Gonococcal infection | Burkina Faso | 176.5 (106.2-307.3) | 2.6 (1.5-4.7) | 269.5 (178.3-421) | 1.4 (0.9-2.3) | -2.04 (-5.04-1.06) |
| Gonococcal infection | Burundi | 123.6 (71.5-228.7) | 2.7 (1.5-5.1) | 199.6 (113.1-374.7) | 1.7 (1-3.5) | -2.38 (-5.42-0.76) |
| Gonococcal infection | Cambodia | 93 (52-160) | 0.9 (0.5-1.5) | 161.8 (94.8-266.8) | 0.9 (0.5-1.5) | 0.59 (-1.73-2.96) |
| Gonococcal infection | Cameroon | 234.8 (147.8-344.8) | 2.8 (1.7-4) | 552.2 (351.7-839.3) | 1.9 (1.2-2.9) | -0.88 (-4.24-2.59) |
| Gonococcal infection | Canada | 138.1 (93.4-199) | 0.4 (0.3-0.6) | 132 (87.6-196.7) | 0.4 (0.2-0.5) | -0.98 (-2.6-0.68) |
| Gonococcal infection | Cape Verde | 3.6 (2.2-6.1) | 1.2 (0.7-2.1) | 6.5 (4-10) | 1 (0.6-1.6) | -0.94 (-3.55-1.73) |
| Gonococcal infection | Central African Republic | 64.7 (37.7-93.8) | 3.1 (1.7-4.5) | 114.6 (72-168.2) | 2.5 (1.5-3.7) | -0.8 (-4.45-2.99) |
| Gonococcal infection | Chad | 106.6 (63.3-219.3) | 2.4 (1.3-5.4) | 219 (136.1-345.6) | 1.7 (1-2.8) | -0.82 (-3.97-2.42) |
| Gonococcal infection | Chile | 120.8 (87.4-170.4) | 0.9 (0.6-1.2) | 136.7 (96.3-198.4) | 0.7 (0.5-1) | -0.28 (-2-1.48) |
| Gonococcal infection | China | 10101.2 (6348.7-15695.3) | 0.8 (0.5-1.1) | 7929.7 (5031-12123) | 0.6 (0.4-0.9) | 0.24 (-1.45-1.95) |
| Gonococcal infection | Colombia | 265.3 (198.8-362.1) | 0.8 (0.6-1.1) | 339.2 (247.8-464) | 0.6 (0.5-0.9) | -0.4 (-2.34-1.57) |
| Gonococcal infection | Comoros | 10.7 (5.6-18.8) | 2.7 (1.4-5) | 13.4 (8.6-22.1) | 1.8 (1.1-2.9) | -1.28 (-3.42-0.91) |
| Gonococcal infection | Congo | 45.2 (29.4-68.1) | 2.3 (1.4-3.5) | 85.6 (56.7-126.7) | 1.6 (1.1-2.4) | -1.1 (-4.52-2.45) |
| Gonococcal infection | Cook Islands | 0.1 (0.1-0.2) | 0.5 (0.3-0.8) | 0.1 (0-0.1) | 0.5 (0.3-0.7) | 0.67 (-1.53-2.91) |
| Gonococcal infection | Costa Rica | 20.3 (14.5-28.6) | 0.7 (0.5-1) | 32.9 (24.5-45.4) | 0.6 (0.5-0.9) | -0.32 (-2.16-1.55) |
| Gonococcal infection | Cote d'Ivoire | 206 (125.6-338.8) | 2.1 (1.2-4.2) | 367.8 (233.9-576.9) | 1.5 (0.9-2.4) | -1.4 (-4.91-2.23) |
| Gonococcal infection | Croatia | 23.6 (14.9-36.6) | 0.5 (0.3-0.8) | 16.3 (10.1-24.4) | 0.4 (0.3-0.7) | -0.16 (-1.26-0.94) |
| Gonococcal infection | Cuba | 100 (66.4-156.5) | 0.8 (0.5-1.2) | 99.4 (75.5-134.4) | 0.9 (0.7-1.3) | 0.59 (-0.97-2.18) |
| Gonococcal infection | Cyprus | 1.4 (0.9-2.1) | 0.2 (0.1-0.3) | 1.6 (1.1-2.3) | 0.1 (0.1-0.1) | -0.24 (-1.59-1.14) |
| Gonococcal infection | Czech Republic | 56.4 (38.2-83) | 0.5 (0.4-0.8) | 41 (27.3-61.3) | 0.5 (0.3-0.7) | -0.29 (-1.18-0.62) |
| Gonococcal infection | Democratic Republic of the Congo | 695 (423.3-1072.5) | 2.4 (1.4-3.7) | 1384.6 (888.7-2342.9) | 1.7 (1.1-3.1) | -2.08 (-5.34-1.29) |
| Gonococcal infection | Denmark | 16.9 (14.1-20.6) | 0.3 (0.2-0.3) | 9.6 (7.8-12) | 0.1 (0.1-0.2) | -1.29 (-2.67-0.11) |
| Gonococcal infection | Djibouti | 7.3 (4.3-13.5) | 2 (1.1-4.1) | 20.3 (12.5-36.7) | 1.6 (1-3) | 1.17 (-2.08-4.54) |
| Gonococcal infection | Dominica | 0.9 (0.6-1.3) | 1.2 (0.8-1.8) | 0.6 (0.4-0.9) | 0.9 (0.6-1.3) | -0.12 (-2.68-2.5) |
| Gonococcal infection | Dominican Republic | 147.4 (101-196.5) | 2.1 (1.5-2.7) | 189.9 (130.9-261.4) | 1.6 (1.1-2.2) | -0.21 (-2.89-2.53) |
| Gonococcal infection | Ecuador | 50.6 (35.5-72) | 0.5 (0.4-0.8) | 92.2 (63-132.7) | 0.5 (0.3-0.7) | 0.41 (-2.02-2.91) |
| Gonococcal infection | Egypt | 440.4 (274.8-684.4) | 0.9 (0.6-1.3) | 742.5 (459.2-1207.5) | 0.7 (0.5-1.1) | -0.62 (-2.09-0.86) |
| Gonococcal infection | El Salvador | 26.6 (17.3-39.2) | 0.5 (0.4-0.8) | 31.8 (20.5-48.5) | 0.5 (0.3-0.7) | -0.37 (-2.5-1.8) |
| Gonococcal infection | Equatorial Guinea | 9.1 (5.3-15.8) | 2.9 (1.6-5.4) | 24 (15-36.3) | 1.6 (1-2.3) | -0.02 (-3.5-3.59) |
| Gonococcal infection | Eritrea | 86.8 (47.2-232.1) | 3.2 (1.6-9.3) | 119.1 (70.4-207.2) | 1.9 (1.1-3.4) | -1.11 (-3.94-1.81) |
| Gonococcal infection | Estonia | 22.8 (18.2-28.9) | 1.4 (1.1-1.8) | 7.4 (5.5-10) | 0.6 (0.4-0.8) | -1.5 (-3.11-0.14) |
| Gonococcal infection | Ethiopia | 1793.1 (818.7-4322.6) | 4.6 (2-12.7) | 1653.1 (981.4-3633.4) | 1.6 (0.9-3.7) | -2.58 (-5.44-0.36) |
| Gonococcal infection | Federated States of Micronesia | 0.6 (0.4-1) | 0.7 (0.4-1) | 0.6 (0.4-1) | 0.6 (0.4-0.9) | 1.06 (-1.48-3.66) |
| Gonococcal infection | Fiji | 5.6 (3.6-8.5) | 0.7 (0.5-1.1) | 5.7 (3.6-8.7) | 0.6 (0.4-0.9) | -0.09 (-2.68-2.57) |
| Gonococcal infection | Finland | 29.4 (24.1-36.8) | 0.5 (0.4-0.6) | 15.5 (11.8-20.3) | 0.3 (0.2-0.4) | -1.21 (-2.17--0.24) |
| Gonococcal infection | France | 115.1 (89.3-149.4) | 0.2 (0.1-0.2) | 70.3 (52.3-96.6) | 0.1 (0.1-0.1) | -1.25 (-3.08-0.62) |
| Gonococcal infection | Gabon | 16.9 (10.4-25.1) | 2 (1.2-3.2) | 25.8 (16.2-38.9) | 1.5 (0.9-2.2) | -1.25 (-4.56-2.18) |
| Gonococcal infection | Georgia | 86 (57.4-130.4) | 1.5 (1-2.3) | 38.7 (24-59.3) | 1.2 (0.7-1.9) | -0.74 (-1.97-0.52) |
| Gonococcal infection | Germany | 159.3 (126.4-201.4) | 0.2 (0.1-0.2) | 88.2 (65.8-120.5) | 0.1 (0.1-0.1) | -1.05 (-2.57-0.48) |
| Gonococcal infection | Ghana | 218.3 (146.1-316.6) | 1.7 (1.1-2.6) | 457.4 (295.4-703.3) | 1.4 (0.9-2) | -0.3 (-3.46-2.95) |
| Gonococcal infection | Greece | 15.2 (11.6-20) | 0.1 (0.1-0.2) | 11.9 (8.3-18.3) | 0.1 (0.1-0.2) | -0.26 (-1.4-0.9) |
| Gonococcal infection | Greenland | 0.3 (0.2-0.4) | 0.4 (0.2-0.6) | 0.2 (0.1-0.3) | 0.3 (0.2-0.5) | -0.61 (-2.62-1.43) |
| Gonococcal infection | Grenada | 1.1 (0.8-1.5) | 1.3 (1-1.8) | 1.2 (0.8-1.6) | 1.1 (0.7-1.5) | -0.33 (-2.33-1.71) |
| Gonococcal infection | Guam | 0.8 (0.4-1.3) | 0.5 (0.3-0.9) | 0.7 (0.4-1.1) | 0.4 (0.2-0.7) | 0.1 (-2.43-2.69) |
| Gonococcal infection | Guatemala | 54.7 (39.4-78) | 0.8 (0.6-1.1) | 102 (73.3-143.6) | 0.6 (0.5-0.9) | -0.57 (-2.56-1.47) |
| Gonococcal infection | Guinea | 106.2 (67.2-169) | 2.2 (1.4-3.8) | 173.5 (109.2-266.1) | 1.5 (0.9-2.5) | -0.39 (-3.49-2.82) |
| Gonococcal infection | Guinea-Bissau | 22.9 (13.5-35.3) | 3 (1.6-5.1) | 33.5 (20.7-50.9) | 1.9 (1.1-2.9) | 0.08 (-2.95-3.2) |
| Gonococcal infection | Guyana | 11.5 (8.4-15.9) | 1.5 (1.2-2) | 9.7 (7.1-13.5) | 1.2 (0.9-1.7) | -0.39 (-2.96-2.25) |
| Gonococcal infection | Haiti | 101.8 (70.7-138.7) | 1.8 (1.3-2.4) | 183.2 (117.3-263.2) | 1.4 (0.9-2) | -0.93 (-4.4-2.66) |
| Gonococcal infection | Honduras | 44.3 (31-62.5) | 1.1 (0.8-1.6) | 83.5 (55.4-123.6) | 0.8 (0.6-1.2) | -1.22 (-3.01-0.6) |
| Gonococcal infection | Hungary | 74.7 (54.1-104) | 0.7 (0.5-1) | 48.1 (33-69.1) | 0.5 (0.4-0.8) | -0.77 (-2.34-0.81) |
| Gonococcal infection | Iceland | 0.4 (0.3-0.5) | 0.1 (0.1-0.2) | 0.3 (0.2-0.4) | 0.1 (0.1-0.1) | -0.57 (-1.89-0.78) |
| Gonococcal infection | India | 14232.1 (9855.6-19597.1) | 1.7 (1.2-2.3) | 20551.4 (13960.2-29739.8) | 1.3 (0.9-1.9) | -0.13 (-2.5-2.3) |
| Gonococcal infection | Indonesia | 2164.8 (1324.4-3324.2) | 1.1 (0.7-1.7) | 3083.8 (1909.6-4739.1) | 1 (0.6-1.6) | 0.86 (-1.43-3.21) |
| Gonococcal infection | Iran | 391.4 (236.3-621.8) | 0.7 (0.4-1.1) | 638.6 (407.4-978.7) | 0.7 (0.5-1.1) | 0.76 (-0.72-2.25) |
| Gonococcal infection | Iraq | 136.8 (80-220.1) | 0.8 (0.5-1.2) | 294.5 (172.8-481.6) | 0.7 (0.4-1.1) | 0.3 (-1.27-1.89) |
| Gonococcal infection | Ireland | 5.9 (4.6-7.8) | 0.2 (0.1-0.2) | 4.8 (3.4-6.8) | 0.1 (0.1-0.1) | -0.74 (-2-0.54) |
| Gonococcal infection | Israel | 7.3 (5.5-9.8) | 0.2 (0.1-0.2) | 7.9 (5.3-11.9) | 0.1 (0.1-0.1) | -0.75 (-2.19-0.71) |
| Gonococcal infection | Italy | 175 (132.2-237) | 0.3 (0.2-0.4) | 132.1 (87.9-199.2) | 0.2 (0.1-0.3) | -1 (-2.77-0.8) |
| Gonococcal infection | Jamaica | 23.4 (15.8-34.4) | 1 (0.7-1.4) | 26.7 (18.5-38.3) | 0.9 (0.6-1.2) | -0.52 (-2.87-1.9) |
| Gonococcal infection | Japan | 717.8 (460-1119.1) | 0.5 (0.3-0.8) | 537.1 (362.7-800) | 0.5 (0.3-0.7) | 0.26 (-0.63-1.16) |
| Gonococcal infection | Jordan | 25.3 (13.4-44.9) | 0.6 (0.4-1.1) | 82.9 (44.2-145.6) | 0.6 (0.3-1) | 0.37 (-1.37-2.14) |
| Gonococcal infection | Kazakhstan | 293.8 (205.9-411.7) | 1.7 (1.2-2.4) | 217.1 (133-337.3) | 1.2 (0.7-1.9) | -0.99 (-2.48-0.52) |
| Gonococcal infection | Kenya | 278.7 (182.7-442.1) | 1.4 (0.9-2.4) | 555.8 (369.3-872.8) | 1.1 (0.7-1.8) | -1.74 (-5.24-1.9) |
| Gonococcal infection | Kiribati | 0.5 (0.3-0.7) | 0.6 (0.4-1) | 0.7 (0.4-1.1) | 0.6 (0.4-0.9) | -0.21 (-2.84-2.5) |
| Gonococcal infection | Kuwait | 13.1 (7.3-21.9) | 0.6 (0.4-1.1) | 31.5 (18.8-49.8) | 0.6 (0.4-1) | 0.22 (-0.7-1.15) |
| Gonococcal infection | Kyrgyzstan | 89.6 (65.1-122.5) | 2.1 (1.5-2.7) | 85.1 (53.9-133.2) | 1.2 (0.8-1.9) | -1.59 (-3.22-0.08) |
| Gonococcal infection | Laos | 40.4 (22.6-68.9) | 1 (0.6-1.7) | 76.5 (44.1-124.7) | 1 (0.6-1.5) | 2.19 (0.18-4.25) |
| Gonococcal infection | Latvia | 43 (34.1-53.3) | 1.5 (1.2-1.9) | 12.6 (9.2-17.3) | 0.7 (0.5-1) | -1.73 (-3.64-0.23) |
| Gonococcal infection | Lebanon | 21 (12.4-34.7) | 0.7 (0.4-1.1) | 38.6 (22.2-63.2) | 0.7 (0.4-1.1) | -0.08 (-1.82-1.69) |
| Gonococcal infection | Lesotho | 33.3 (22.1-49.3) | 2.6 (1.7-3.7) | 51 (33.9-75.5) | 2.7 (1.8-3.9) | 0.22 (-3.54-4.13) |
| Gonococcal infection | Liberia | 43 (26.3-70.7) | 2.1 (1.3-3.6) | 75.8 (48-116.7) | 1.5 (0.9-2.4) | 0.18 (-3.18-3.66) |
| Gonococcal infection | Libya | 28.3 (15.2-48) | 0.7 (0.4-1.1) | 50.2 (30.3-77.3) | 0.6 (0.4-1) | 0.28 (-1.35-1.94) |
| Gonococcal infection | Lithuania | 29.3 (20.8-40.2) | 0.8 (0.5-1.1) | 13.5 (9.1-19.5) | 0.5 (0.4-0.8) | -0.28 (-1.96-1.42) |
| Gonococcal infection | Luxembourg | 1.4 (1.1-1.7) | 0.3 (0.3-0.4) | 0.9 (0.7-1.4) | 0.1 (0.1-0.2) | -1.35 (-2.76-0.09) |
| Gonococcal infection | Macedonia | 10.5 (6.4-15.9) | 0.5 (0.3-0.8) | 9.7 (5.8-15.3) | 0.5 (0.3-0.7) | -0.35 (-1.35-0.66) |
| Gonococcal infection | Madagascar | 296.2 (169.5-427.6) | 2.8 (1.6-4) | 601.4 (406.2-873.9) | 2.2 (1.4-3.1) | 1.41 (-1.31-4.21) |
| Gonococcal infection | Malawi | 190.9 (114.6-285.5) | 2.3 (1.3-3.4) | 298.3 (182.4-462.5) | 1.6 (1-2.7) | -1.51 (-5.09-2.2) |
| Gonococcal infection | Malaysia | 170.8 (103.4-290.2) | 0.9 (0.6-1.5) | 303.7 (173.5-521.3) | 0.9 (0.5-1.5) | -0.02 (-1.99-1.99) |
| Gonococcal infection | Maldives | 2.8 (1.8-4.3) | 1.4 (0.9-2.1) | 6.4 (3.5-10.2) | 1.1 (0.6-1.7) | 0.19 (-1.28-1.68) |
| Gonococcal infection | Mali | 201.9 (125-327.6) | 3.1 (1.8-5.5) | 309.7 (201.2-481.8) | 1.6 (1-2.7) | -0.61 (-3.63-2.51) |
| Gonococcal infection | Malta | 0.4 (0.3-0.6) | 0.1 (0.1-0.2) | 0.4 (0.3-0.5) | 0.1 (0.1-0.1) | -0.46 (-1.82-0.92) |
| Gonococcal infection | Marshall Islands | 0.2 (0.1-0.4) | 0.6 (0.4-1) | 0.3 (0.2-0.5) | 0.6 (0.4-0.9) | 1.27 (-1.19-3.79) |
| Gonococcal infection | Mauritania | 35.9 (22.6-60.5) | 2.2 (1.3-3.9) | 51.3 (33.4-76.1) | 1.3 (0.8-2) | -0.99 (-3.24-1.32) |
| Gonococcal infection | Mauritius | 10.7 (5.9-17.1) | 0.9 (0.5-1.3) | 11 (7-17.2) | 0.8 (0.5-1.4) | 2.09 (0.34-3.86) |
| Gonococcal infection | Mexico | 640.5 (479.7-864.4) | 0.8 (0.6-1) | 972 (729.5-1343.5) | 0.7 (0.5-1) | -0.16 (-2.13-1.85) |
| Gonococcal infection | Moldova | 47.9 (36.7-62.3) | 1.1 (0.8-1.4) | 24.9 (18.3-34.5) | 0.7 (0.5-1) | -0.76 (-2.62-1.14) |
| Gonococcal infection | Monaco | 0 (0-0.1) | 0.1 (0.1-0.2) | 0 (0-0) | 0.1 (0.1-0.1) | -0.69 (-1.99-0.62) |
| Gonococcal infection | Mongolia | 57.7 (33.8-98.4) | 2.9 (1.6-5) | 53.8 (35.5-79.1) | 1.6 (1.1-2.4) | 0.06 (-2.01-2.17) |
| Gonococcal infection | Montenegro | 3.3 (2.1-5.5) | 0.5 (0.3-0.8) | 2.8 (1.8-4.4) | 0.5 (0.3-0.8) | -0.19 (-1.37-1.01) |
| Gonococcal infection | Morocco | 182.8 (102.8-319.3) | 0.7 (0.4-1.2) | 261.5 (151.2-417.4) | 0.7 (0.4-1.1) | 0.05 (-2.75-2.94) |
| Gonococcal infection | Mozambique | 431.7 (221.7-696.5) | 3.9 (2-6.4) | 643.4 (396.7-1093.7) | 2.3 (1.4-4) | -0.91 (-4.37-2.67) |
| Gonococcal infection | Myanmar | 453.3 (275.4-771.7) | 1 (0.7-1.7) | 563.2 (343.7-923.8) | 1 (0.6-1.6) | 0.78 (-1.77-3.39) |
| Gonococcal infection | Namibia | 34.3 (22.4-52.6) | 2.7 (1.8-4.1) | 52 (33.8-75.5) | 2 (1.4-3) | -0.65 (-4.11-2.94) |
| Gonococcal infection | Nauru | 0.1 (0-0.1) | 0.6 (0.4-0.9) | 0.1 (0-0.1) | 0.6 (0.3-0.9) | 1.14 (-1.44-3.79) |
| Gonococcal infection | Nepal | 247.5 (165.4-385) | 1.6 (1.1-2.6) | 288.9 (196.3-424.5) | 0.9 (0.6-1.3) | 1.4 (-1.18-4.06) |
| Gonococcal infection | Netherlands | 31.5 (24.8-40.6) | 0.2 (0.1-0.2) | 18.3 (13.6-24.9) | 0.1 (0.1-0.1) | -1.07 (-2.58-0.46) |
| Gonococcal infection | New Zealand | 22.9 (13.9-34.8) | 0.6 (0.4-0.9) | 29.1 (17.8-44.4) | 0.6 (0.3-0.9) | -0.6 (-1.83-0.66) |
| Gonococcal infection | Nicaragua | 19.6 (12.9-30.1) | 0.6 (0.4-0.8) | 34.4 (22.5-51.2) | 0.5 (0.3-0.7) | -0.57 (-2.42-1.33) |
| Gonococcal infection | Niger | 137.5 (77-287.7) | 2.4 (1.2-5.9) | 265.2 (156.2-460.8) | 1.4 (0.8-2.7) | -1.35 (-3.77-1.12) |
| Gonococcal infection | Nigeria | 1302.8 (776.6-2185.9) | 1.8 (1-3.2) | 2370.6 (1511-3696.2) | 1.2 (0.7-2) | -0.33 (-3.34-2.76) |
| Gonococcal infection | Niue | 0 (0-0) | 0.5 (0.3-0.8) | 0 (0-0) | 0.5 (0.3-0.8) | 0.93 (-1.36-3.28) |
| Gonococcal infection | North Korea | 118 (68.9-193.2) | 0.5 (0.3-0.9) | 154.2 (90.5-246.2) | 0.6 (0.3-0.9) | 1.29 (-0.41-3.01) |
| Gonococcal infection | Northern Mariana Islands | 0.3 (0.2-0.5) | 0.6 (0.4-0.9) | 0.2 (0.1-0.4) | 0.5 (0.3-0.8) | -0.16 (-2.48-2.21) |
| Gonococcal infection | Norway | 9.3 (6-15) | 0.2 (0.1-0.3) | 13.7 (9.4-20.1) | 0.2 (0.2-0.4) | -0.07 (-1.31-1.18) |
| Gonococcal infection | Oman | 16.2 (9.4-27.4) | 0.9 (0.5-1.4) | 38.2 (21.8-63.5) | 0.7 (0.4-1.2) | 0.1 (-1.3-1.52) |
| Gonococcal infection | Pakistan | 759.2 (512.5-1242.6) | 1 (0.6-1.7) | 1353.3 (983.3-1871.9) | 0.6 (0.5-0.9) | 2.13 (-0.38-4.71) |
| Gonococcal infection | Palau | 0.1 (0.1-0.1) | 0.5 (0.3-0.9) | 0.1 (0.1-0.1) | 0.5 (0.3-0.8) | 0.87 (-1.2-2.98) |
| Gonococcal infection | Palestine | 19.5 (11.5-32) | 1.2 (0.8-1.9) | 45.7 (27.8-72.2) | 1 (0.6-1.5) | 0.18 (-1.25-1.62) |
| Gonococcal infection | Panama | 15.2 (10.9-21.9) | 0.6 (0.5-0.9) | 24.6 (17.4-35.3) | 0.6 (0.4-0.8) | -0.19 (-2.52-2.2) |
| Gonococcal infection | Papua New Guinea | 34 (20.9-53) | 0.9 (0.6-1.4) | 74.8 (42.6-120.3) | 0.7 (0.4-1.2) | 0.95 (-1.92-3.91) |
| Gonococcal infection | Paraguay | 28.6 (20.3-40.7) | 0.8 (0.6-1.1) | 53.4 (38.6-75.3) | 0.7 (0.5-1) | 0.55 (-2.01-3.19) |
| Gonococcal infection | Peru | 130.9 (94.7-180.7) | 0.7 (0.5-0.9) | 200.4 (137.3-284.3) | 0.5 (0.4-0.7) | -1.14 (-3.45-1.23) |
| Gonococcal infection | Philippines | 607.7 (353.3-956.2) | 0.9 (0.6-1.4) | 1256.4 (781.2-1890.8) | 1 (0.7-1.5) | 2.65 (0.47-4.88) |
| Gonococcal infection | Poland | 228.9 (156.3-338.4) | 0.6 (0.4-0.9) | 149.7 (95.3-233.6) | 0.4 (0.3-0.7) | 0.44 (-0.67-1.57) |
| Gonococcal infection | Portugal | 21.6 (17.2-27.2) | 0.2 (0.2-0.3) | 11.2 (8.1-15.7) | 0.1 (0.1-0.1) | -0.97 (-3.21-1.33) |
| Gonococcal infection | Puerto Rico | 32.5 (21.8-49) | 0.9 (0.6-1.3) | 23.7 (16.3-34.9) | 0.8 (0.5-1.1) | -1.18 (-3.47-1.17) |
| Gonococcal infection | Qatar | 4.7 (2.8-7.9) | 1.2 (0.7-1.8) | 28.5 (15.7-45.6) | 0.8 (0.5-1.3) | -0.84 (-2.44-0.79) |
| Gonococcal infection | Romania | 121.5 (77.9-184.9) | 0.5 (0.3-0.8) | 72.9 (46.2-115) | 0.4 (0.3-0.7) | -0.64 (-2.33-1.08) |
| Gonococcal infection | Russian Federation | 1405.9 (1093.9-1872.5) | 0.9 (0.7-1.2) | 1039.7 (794.6-1421) | 0.7 (0.5-1) | -0.01 (-2.12-2.15) |
| Gonococcal infection | Rwanda | 208.6 (117.7-348.6) | 3.4 (1.9-5.9) | 205.2 (122.7-346.8) | 1.6 (0.9-2.7) | -2.13 (-5.1-0.94) |
| Gonococcal infection | Saint Kitts and Nevis | 0.6 (0.4-0.7) | 1.4 (1.1-1.8) | 0.6 (0.4-0.9) | 1 (0.7-1.4) | -0.04 (-2.46-2.43) |
| Gonococcal infection | Saint Lucia | 1.8 (1.3-2.6) | 1.4 (1.1-1.9) | 2.2 (1.6-2.9) | 1.1 (0.8-1.5) | -0.33 (-2.21-1.58) |
| Gonococcal infection | Saint Vincent and the Grenadines | 3 (2.3-3.9) | 2.9 (2.3-3.7) | 1.4 (1-1.9) | 1.2 (0.8-1.6) | -0.95 (-3.39-1.54) |
| Gonococcal infection | Samoa | 1 (0.5-1.7) | 0.6 (0.4-1) | 1.1 (0.6-1.7) | 0.5 (0.3-0.8) | 1 (-0.74-2.77) |
| Gonococcal infection | San Marino | 0 (0-0) | 0.1 (0.1-0.1) | 0 (0-0) | 0.1 (0.1-0.1) | -0.63 (-1.96-0.72) |
| Gonococcal infection | Sao Tome and Principe | 1.4 (0.8-2.4) | 1.5 (0.9-2.6) | 2.6 (1.7-4.1) | 1.2 (0.8-2) | -0.96 (-2.73-0.83) |
| Gonococcal infection | Saudi Arabia | 116.3 (61.5-191.1) | 0.7 (0.4-1.1) | 298.4 (172.5-473.7) | 0.7 (0.4-1.1) | -0.08 (-1.72-1.6) |
| Gonococcal infection | Senegal | 113.2 (66.4-208.9) | 2 (1.1-4) | 149.9 (93.5-235.9) | 1.1 (0.7-1.9) | -1.3 (-3.87-1.34) |
| Gonococcal infection | Serbia | 48.6 (31.6-73.7) | 0.5 (0.3-0.8) | 38.6 (24.4-59.1) | 0.5 (0.3-0.7) | -0.21 (-1.49-1.08) |
| Gonococcal infection | Seychelles | 1 (0.7-1.6) | 1.3 (0.9-2) | 1.1 (0.7-1.7) | 1.1 (0.7-1.7) | 0.46 (-1.38-2.33) |
| Gonococcal infection | Sierra Leone | 63.7 (38.7-103.4) | 1.8 (1.1-3.2) | 113.6 (75.5-172.8) | 1.4 (0.9-2.2) | -0.02 (-2.85-2.89) |
| Gonococcal infection | Singapore | 24.5 (16.4-35.1) | 0.7 (0.5-0.9) | 29.7 (18.6-44.7) | 0.4 (0.3-0.7) | -0.6 (-1.88-0.7) |
| Gonococcal infection | Slovakia | 27.8 (17.7-42.5) | 0.5 (0.3-0.8) | 23.6 (15.5-37) | 0.5 (0.3-0.8) | -0.14 (-1-0.73) |
| Gonococcal infection | Slovenia | 10.5 (6.8-15.9) | 0.5 (0.3-0.8) | 6.9 (4.3-10.6) | 0.4 (0.2-0.6) | -0.55 (-1.52-0.43) |
| Gonococcal infection | Solomon Islands | 2.1 (1.2-3.3) | 0.7 (0.4-1.1) | 4.1 (2.4-6.5) | 0.6 (0.4-1) | 0.76 (-1.95-3.55) |
| Gonococcal infection | Somalia | 271.5 (156.9-673.8) | 4.4 (2.3-13.4) | 658.7 (393.3-1372.8) | 3.6 (2-8.9) | 0.62 (-2.04-3.36) |
| Gonococcal infection | South Africa | 1431.9 (995.7-1984.5) | 3.8 (2.7-5.2) | 1605.1 (1071.7-2410.3) | 2.6 (1.7-3.9) | -0.51 (-3.9-3.01) |
| Gonococcal infection | South Korea | 318.2 (208.1-488.3) | 0.6 (0.4-1) | 250 (156.6-374.4) | 0.5 (0.3-0.7) | -0.2 (-1.6-1.21) |
| Gonococcal infection | South Sudan | 141.9 (82.8-281.9) | 2.9 (1.6-6.6) | 213.6 (125.9-432.4) | 2.6 (1.5-5.6) | 0.59 (-2.73-4.02) |
| Gonococcal infection | Spain | 113.6 (95.4-140.2) | 0.3 (0.2-0.3) | 69.9 (50.8-97.4) | 0.1 (0.1-0.2) | -1.37 (-3.39-0.69) |
| Gonococcal infection | Sri Lanka | 180.4 (111.1-282.1) | 1 (0.6-1.5) | 187.6 (114.5-303.1) | 0.9 (0.5-1.4) | -0.03 (-1.25-1.21) |
| Gonococcal infection | Sudan | 138.3 (77.4-223) | 0.7 (0.4-1.2) | 323.7 (175-538) | 0.7 (0.4-1.1) | 0.45 (-2.48-3.47) |
| Gonococcal infection | Suriname | 4.1 (2.5-6.2) | 1 (0.7-1.5) | 4.7 (3-6.9) | 0.8 (0.5-1.2) | -0.25 (-2.76-2.33) |
| Gonococcal infection | Swaziland | 20 (13.3-30.1) | 3.1 (2.1-4.6) | 28 (18.3-41.7) | 2.3 (1.5-3.4) | 0.17 (-3.5-3.98) |
| Gonococcal infection | Sweden | 12.9 (9.3-18.6) | 0.1 (0.1-0.2) | 16.3 (11.3-24.9) | 0.2 (0.1-0.2) | -0.61 (-1.86-0.66) |
| Gonococcal infection | Switzerland | 20.6 (16-27.4) | 0.3 (0.2-0.4) | 14.1 (10.4-19.1) | 0.1 (0.1-0.2) | -1.31 (-2.83-0.24) |
| Gonococcal infection | Syria | 109.9 (70.5-167.9) | 1 (0.7-1.4) | 106.1 (66.6-168.2) | 0.7 (0.5-1.1) | -0.23 (-1.72-1.29) |
| Gonococcal infection | Taiwan | 127.2 (75.9-203.3) | 0.6 (0.3-0.9) | 111.6 (64.1-174.5) | 0.5 (0.3-0.8) | 0.52 (-0.69-1.74) |
| Gonococcal infection | Tajikistan | 108.8 (75.4-157.2) | 2.2 (1.5-3) | 173.7 (107.5-274.8) | 1.6 (1-2.5) | -0.83 (-2.36-0.73) |
| Gonococcal infection | Tanzania | 643.5 (402.7-958) | 2.9 (1.8-4.2) | 1042.5 (680-1620.5) | 1.9 (1.2-2.9) | -1.93 (-5.41-1.68) |
| Gonococcal infection | Thailand | 606.5 (358.3-952.8) | 0.9 (0.6-1.5) | 510.6 (302.1-760.3) | 0.8 (0.5-1.3) | -1.84 (-4.44-0.83) |
| Gonococcal infection | The Bahamas | 2.4 (1.5-3.7) | 0.8 (0.6-1.2) | 3.6 (2.5-5.1) | 0.8 (0.6-1.2) | -0.2 (-3-2.68) |
| Gonococcal infection | The Gambia | 13.9 (8.7-22.6) | 1.7 (1.1-3.1) | 31.4 (19.7-48.5) | 1.4 (0.9-2.2) | 0.52 (-2.59-3.73) |
| Gonococcal infection | Timor-Leste | 7.9 (4.4-12.8) | 1 (0.6-1.6) | 14.2 (8.1-23.3) | 0.9 (0.6-1.5) | 0.7 (-1.77-3.23) |
| Gonococcal infection | Togo | 57.4 (37.6-90.4) | 2 (1.3-3.3) | 109.2 (67.9-169.9) | 1.4 (0.9-2.3) | -1.34 (-4.49-1.91) |
| Gonococcal infection | Tokelau | 0 (0-0) | 0.6 (0.3-0.9) | 0 (0-0) | 0.5 (0.3-0.8) | 0.88 (-1.44-3.25) |
| Gonococcal infection | Tonga | 0.5 (0.3-0.9) | 0.6 (0.3-0.9) | 0.5 (0.3-0.8) | 0.5 (0.3-0.8) | -0.18 (-2.35-2.03) |
| Gonococcal infection | Trinidad and Tobago | 10.5 (6.9-15.1) | 0.9 (0.6-1.2) | 10.1 (6.8-15) | 0.7 (0.5-1.1) | -0.61 (-3.06-1.9) |
| Gonococcal infection | Tunisia | 58.3 (31.8-91) | 0.7 (0.4-1) | 72.6 (44-115.7) | 0.6 (0.4-1) | 0.49 (-1-2) |
| Gonococcal infection | Turkey | 441.3 (247.6-752.7) | 0.7 (0.4-1.2) | 578.7 (338.2-909.2) | 0.7 (0.4-1.1) | 0.12 (-1.61-1.89) |
| Gonococcal infection | Turkmenistan | 77.6 (54.3-107.8) | 2.1 (1.5-2.9) | 66.8 (41.8-109) | 1.2 (0.8-2) | -1.44 (-3.19-0.34) |
| Gonococcal infection | Tuvalu | 0.1 (0-0.1) | 0.6 (0.4-0.9) | 0.1 (0-0.1) | 0.5 (0.3-0.9) | 0.58 (-1.59-2.81) |
| Gonococcal infection | Uganda | 380.8 (241.4-640.5) | 2.6 (1.7-4.2) | 1009.1 (670.2-1623.3) | 2.4 (1.6-3.8) | -0.91 (-4.47-2.78) |
| Gonococcal infection | Ukraine | 486.4 (360.7-658.6) | 0.9 (0.7-1.2) | 269.1 (188.7-372.2) | 0.6 (0.4-0.9) | -0.04 (-2.37-2.33) |
| Gonococcal infection | United Arab Emirates | 17.6 (10.6-28.7) | 0.9 (0.6-1.4) | 71.5 (44.3-106.1) | 0.8 (0.5-1.2) | -0.05 (-1.32-1.24) |
| Gonococcal infection | United Kingdom | 182.6 (138-257.3) | 0.3 (0.2-0.4) | 227.7 (158.5-326.6) | 0.3 (0.2-0.5) | -0.05 (-1.53-1.45) |
| Gonococcal infection | United States | 1474.6 (1019.4-2099) | 0.5 (0.4-0.7) | 1117.4 (791-1617.7) | 0.3 (0.2-0.5) | -1.38 (-3.39-0.67) |
| Gonococcal infection | Uruguay | 31.6 (23.5-44.4) | 1 (0.8-1.5) | 29.1 (21.4-40.3) | 0.8 (0.6-1.2) | -0.43 (-2.37-1.55) |
[truncated: 227,146 more chars]
